# Neutrophil elastase-mediated myoepithelial loss is a therapeutic vulnerability in ductal carcinoma *in situ* progression

**DOI:** 10.1016/j.xcrm.2026.103050

**Published:** 2026-09-11

**Authors:** Tarek Taifour, Yu Gu, Adéline Massé, Xiwei Meng, Clarisse Dane Echavez, Dongmei Zuo, Virginie Sanguin-Gendreau, Anne Blouin, Adda-Lee Graham-Paquin, Miranda Addie, Emilie Solymoss, Bin Xiao, Hailey Proud, Sherif Samer Attalla, Alice Jisoo Nam, Vasilios Papavasiliou, Hellen Kuasne, Peter M. Siegel, Khandan Keyomarsi, Luke McCaffrey, Morag Park, William J. Muller

**Affiliations:** 1 McGill University, Division of Experimental Medicine, Department of Medicine, Faculty of Medicine, Montreal, QC H4A 3J1, Canada; 2 Rosalind and Morris Goodman Cancer Institute, Montreal, QC H3A 1A3, Canada; 3 McGill University, Department of Biochemistry, Faculty of Medicine, Montreal, QC H3A 1A3, Canada; 4 Department of Surgery, Division of Surgical and Interventional Sciences, McGill University, Montreal, QC H3G 1A4, Canada; 5 Department of Experimental Radiation Oncology, University of Texas MD Anderson Cancer Center, Houston, TX 77030, USA

**Keywords:** breast cancer progression, ductal carcinoma *in situ*, DCIS, myoepithelial layer, extracellular matrix, neutrophils, neutrophil elastase, tumor microenvironment, invasive transition, metastasis

## Abstract

Progression from ductal carcinoma *in situ* (DCIS) to invasive ductal carcinoma (IDC) marks a critical step in breast cancer progression, yet the mechanisms governing this transition remain poorly defined. Here, we identify neutrophils as a key immunological driver of DCIS progression. Across two independent breast cancer mouse models, neutrophil depletion or genetic deletion of their tumor-derived chemoattractant chitinase-3 like 1 (Chi3l1) preserves the myoepithelial barrier, halts DCIS-to-IDC transition, and suppresses lung metastasis. Mechanistically, tumor-infiltrating neutrophils release neutrophil elastase (NE) that degrades the basement membrane extracellular matrix, leading to erosion of the myoepithelial layer by anoikis. Pharmacological NE inhibition restores myoepithelial integrity and blocks DCIS progression and metastasis. Consistently, elevated NE levels in human breast cancer samples are associated with myoepithelial disruption, DCIS progression, and metastasis. Together, these findings identify neutrophils as key mediators of myoepithelial barrier disruption and highlight NE as a potential therapeutic target for high-risk DCIS.

## Highlights

• Neutrophils drive the transition from ductal carcinoma *in situ* to invasive breast cancer
• Neutrophil elastase degrades basement membrane and induces myoepithelial anoikis
• Neutrophil elastase inhibition preserves the myoepithelium and limits metastasis
• Human ductal carcinoma *in situ* links neutrophil elastase to myoepithelial loss

## Graphical abstract

**Figure:**
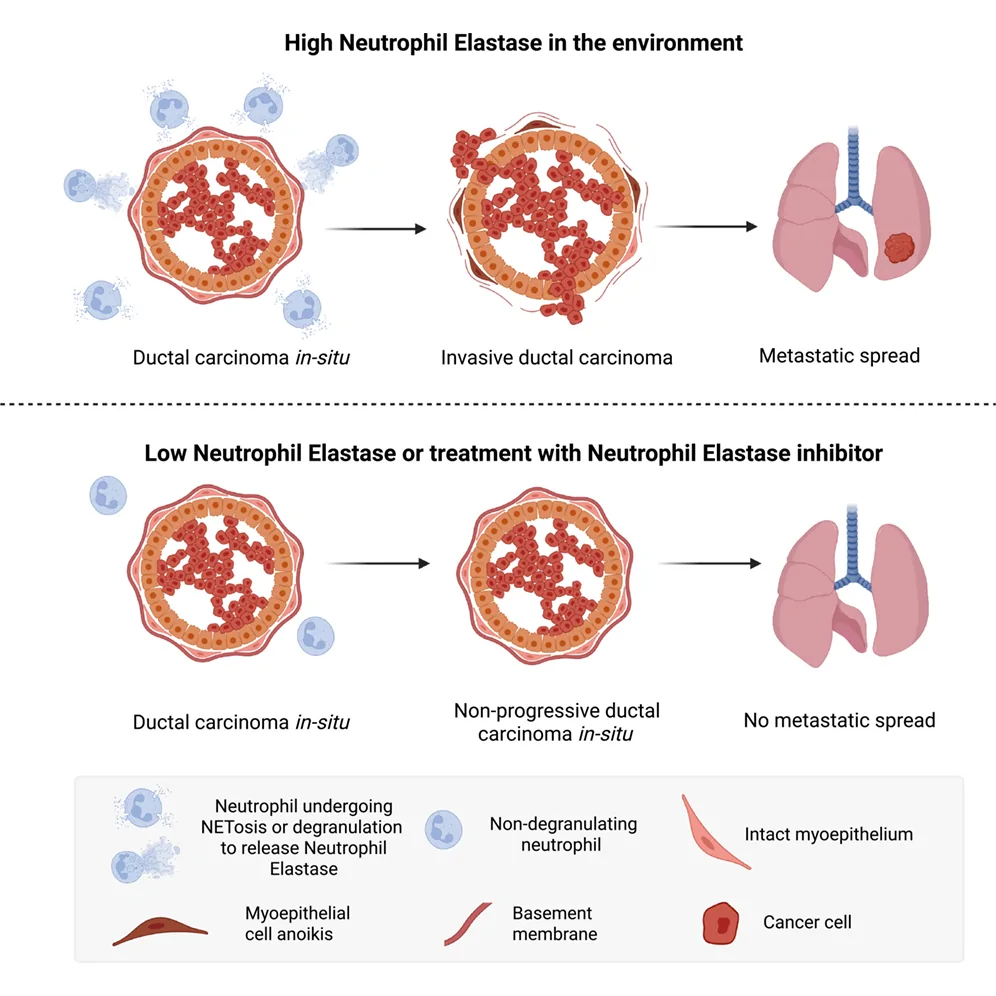

## Teaser

Transition from ductal carcinoma *in situ* (DCIS) to invasive breast cancer remains a key step in disease progression that is poorly understood. Taifour et al. show that neutrophil elastase disrupts the myoepithelial barrier to promote invasive progression, identifying neutrophil elastase as a potential therapeutic target for high-risk DCIS.

## Introduction

The transition from ductal carcinoma *in situ* (DCIS) to invasive ductal carcinoma (IDC) marks a pivotal event in breast cancer progression.^1^ With widespread screening programs, early detection of non-invasive DCIS now accounts for approximately 20% of newly diagnosed breast cancers.^1^^,^^2^ Approximately half of DCIS lesions progress to IDC, yet reliable biomarkers to identify high-risk lesions remain lacking.^1^^,^^2^ DCIS is distinguished from IDC by an intact myoepithelial layer that functions as a physical and biochemical barrier to invasion.^3^ Disruption of this layer is a prerequisite for invasive progression, yet the mechanisms governing myoepithelial breakdown remain poorly understood.^3^ Understanding the mechanisms governing myoepithelial disruption may improve risk stratification and therapeutic intervention for early breast cancer.

Genomic and transcriptomic studies indicate that DCIS and matched IDC share most genetic alterations, suggesting that invasive progression may depend more on changes in the tumor microenvironment than on additional tumor-intrinsic mutations.^4^^,^^5^^,^^6^ Thus, alterations in the tumor immune microenvironment (TIME) have emerged as potential drivers of DCIS progression.^7^^,^^8^^,^^9^^,^^10^^,^^11^^,^^12^ Among immune components, neutrophils are increasingly recognized as critical mediators of immune suppression, metastatic progression, and poor patient outcomes across multiple cancer types.^13^^,^^14^^,^^15^^,^^16^^,^^17^ Importantly, neutrophils release a diverse repertoire of granule-associated proteases capable of degrading extracellular matrix (ECM) and basement membrane components, positioning them as attractive candidates to regulate the integrity of the myoepithelial barrier.^18^^,^^19^ Elevated neutrophil infiltration has been observed in DCIS.^20^ However, whether neutrophils directly contribute to myoepithelial layer disruption and invasive transition during DCIS progression remains unknown.

In this study, we identify neutrophil-derived neutrophil elastase (NE) as a critical mediator of myoepithelial layer disruption and DCIS progression. Neutrophil depletion as well as genetic or pharmacological inhibition of NE across multiple breast cancer genetically engineered mouse models (GEMMs) preserved the myoepithelial barrier, blocked DCIS progression, and reduced lung metastases. Consistently, human DCIS lesions with elevated neutrophil infiltration or NE levels exhibited pronounced myoepithelial disruption. Mechanistically, NE released during neutrophil degranulation or NETosis degraded basement membrane ECM, inducing myoepithelial anoikis. Together, these findings establish neutrophils and NE as key drivers of myoepithelial layer loss and DCIS progression.

## Results

### Neutrophils promote myoepithelial layer disruption

To investigate whether neutrophils contribute to myoepithelial layer disruption during breast cancer progression, we utilized a doxycycline-inducible GEMM co-expressing the oncogenic polyomavirus middle T antigen (PyMT) and Cre recombinase (herein referred to as MIC).^21^ This model recapitulates the stepwise progression of luminal-B breast cancer, developing mammary hyperplasia within 1 week of induction, DCIS-like lesions by 2 weeks, and IDCs by 5–6 weeks, which subsequently metastasize to the lungs.^21^^,^^22^^,^^23^ Using neutrophil depletion via an anti-Ly6G (lymphocyte antigen 6 complex locus G6D) antibody previously established and validated in this model,^24^^,^^25^ we assessed myoepithelial integrity during early tumor progression. As described in these prior studies, 8-week-old MIC mice were treated with anti-Ly6G antibody starting at doxycycline induction and continuing for 2 weeks, which allowed the assessment of neutrophil function at the DCIS stage of disease progression.

Staining for various myoepithelial markers, including cytokeratin 14 (CK14), cytokeratin 5 (CK5), alpha-smooth muscle actin (α-SMA), calponin 1, and P63, revealed significant retention of the myoepithelial layer in anti-Ly6G-treated glands compared with IgG2a-treated controls (Figures 1A–1E; Figures S1A and S1B). Ductal lesions in neutrophil-depleted glands exhibited a continuous and structurally intact myoepithelial layer, whereas control lesions showed marked fragmentation and loss of myoepithelial marker expression (Figures 1A–1E; Figures S1A and S1B). Importantly, neutrophil depletion had no impact on cancer cell proliferation, as assessed through staining for proliferation markers Ki67 and proliferating cell nuclear antigen (PCNA) (Figures S1C–S1E), indicating that preservation of the myoepithelium was not driven by differences in cancer cell proliferation.

**Figure 1:**
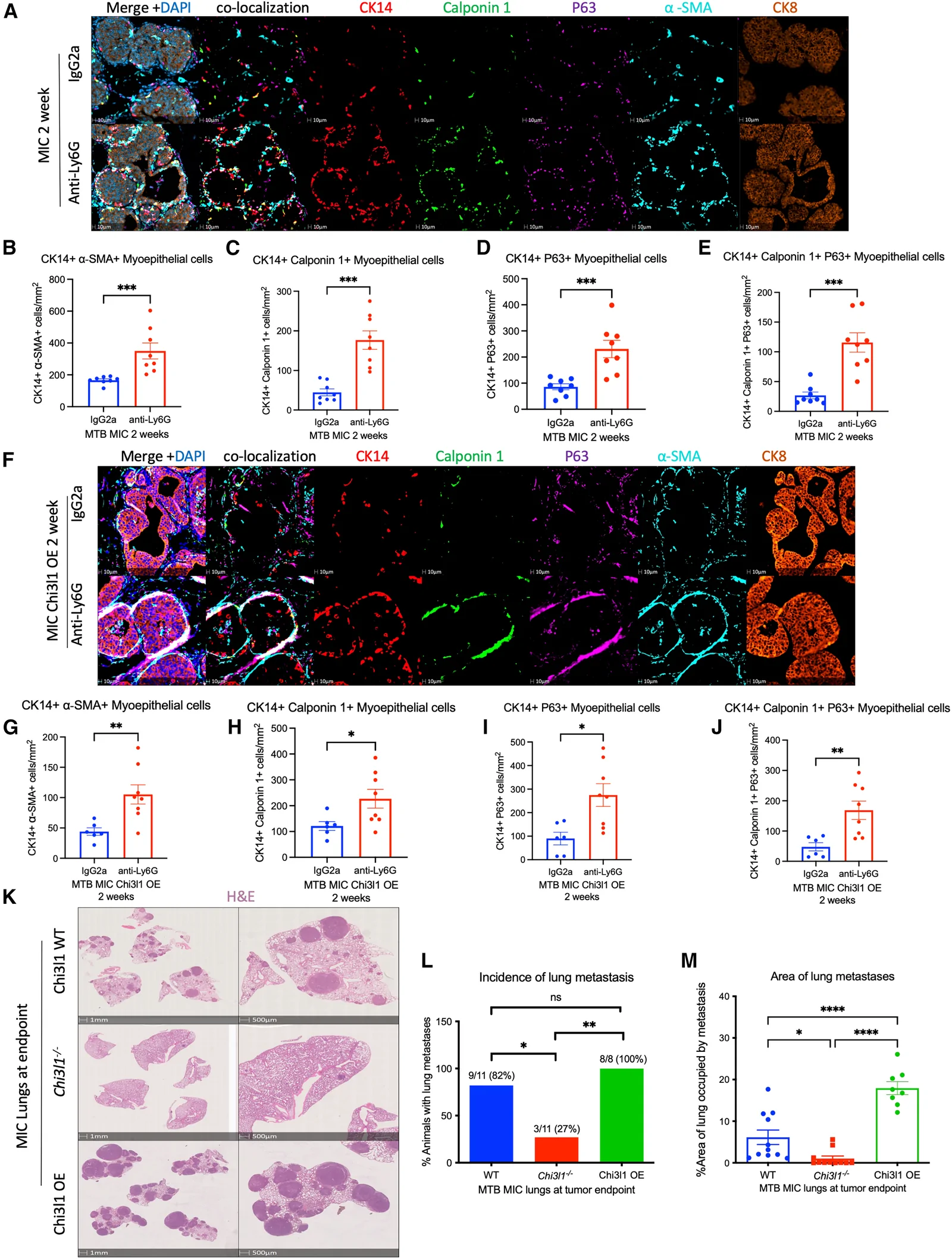
Neutrophils promote DCIS progression (A) Fluorescent immunohistochemistry (IHC) staining of mammary tissue from WT MIC mice treated with anti-Ly6G or IgG2a isotype control for 2 weeks, using antibodies against CK14, α-SMA, calponin 1, P63, and CK8, counterstained with DAPI. (B–E) Quantification of total CK14^+^ α-SMA^+^ (Cohen’s d = −1.8016), CK14^+^ calponin 1^+^ (Cohen’s d = −2.6470), CK14^+^ P63^+^ (Cohen’s d = −2.0707), and CK14^+^ calponin 1^+^ P63^+^ (Cohen’s d = −2.5783) cells normalized to total analyzed area in IgG2a (*n* = 8) and anti-Ly6G (*n* = 8)-treated WT MIC mammary glands at 2 weeks post-induction. Derived from the staining shown in (A). (F) Fluorescent IHC staining of mammary tissue from Chi3l1 OE MIC mice treated with anti-Ly6G or IgG2a isotype control for 2 weeks, using antibodies against CK14, α-SMA, calponin 1, P63, and CK8, counterstained with DAPI. (G–J) Quantification of total CK14^+^ α-SMA^+^ (Cohen’s d = −1.7167), CK14^+^ calponin 1^+^ (Cohen’s d = −1.2640), CK14^+^ P63^+^ (Cohen’s d = −1.6381), and CK14^+^ calponin 1^+^ P63^+^ (Cohen’s d = −1.7400) cells normalized to total analyzed area in IgG2a (*n* = 6) and anti-Ly6G (*n* = 8)-treated Chi3l1 OE MIC mammary glands at 2 weeks post-induction. Derived from the staining shown in (F). (K) Representative H&E staining of WT, Chi3l1^−/−^, and Chi3l1 OE MIC lungs at mammary tumor endpoint. (L) Percentage of WT, Chi3l1^−/−^, and Chi3l1 OE MIC mice with pulmonary metastases. (M) Quantification of total pulmonary metastatic area in WT (*n* = 11), Chi3l1^−/−^ (*n* = 11), and Chi3l1 OE (*n* = 8) MIC lungs at mammary tumor endpoint (average of 59 days since tumor onset for WT, 57 days for Chi3l1^−/−^, and 60 days for Chi3l1 OE) (Eta squared; η2 = 0.73). Represented as percent of total lung area. ^∗^*p* < 0.05, ^∗∗^*p* < 0.01, ^∗∗∗^*p* < 0.001, and ^∗∗∗∗^*p* < 0.0001 calculated by Mann-Whitney non-parametric test (for B–E and G–J), Fisher’s exact test (for L), or one-way ANOVA with Tukey’s post hoc test (for M). Data are presented as mean ± SEM. Each data point is an independent biological sample, and *N* represents the number of independent biological replicates. Analysis was performed by the HALO (Indica Labs) software. Quantification was performed on whole-slide scans of the entire mammary gland section. Scale bars: 10 (A and F) and 500 μm (K).

To validate these findings in a second independent GEMM representing a distinct breast cancer subtype, we depleted neutrophils in the C3(1)-TAg (C3Tag) GEMM (Figures S1F–S1H). This model develops aggressive basal-like mammary tumors driven by SV40 large T-antigen expression.^26^^,^^27^ Consistent with our findings in the MIC GEMM, neutrophil depletion in the C3Tag GEMM led to a significant retention of myoepithelial content without altering cancer cell proliferation (Figures S1I–S1P). Overall, these findings demonstrate that neutrophils promote myoepithelial barrier disruption during DCIS progression in luminal-B and basal breast cancer GEMMs.

### Targeting neutrophil chemoattractants preserves myoepithelial integrity

We next asked whether tumor-intrinsic regulation of neutrophil recruitment similarly influences myoepithelial integrity. We previously demonstrated that the tumor-derived cytokine chitinase-3 like 1 (Chi3l1) promotes neutrophil recruitment to the TIME.^24^^,^^25^ To assess whether Chi3l1-dependent neutrophil accumulation contributes to myoepithelial layer disruption, we crossed Chi3l1-deficient (*Chi3l1*^−/−^) or mammary epithelial-specific Chi3l1-overexpressing (Chi3l1 OE) mice with the MIC model (Figures S2A–S2C). After tumor onset, these models exhibited no differences in disease-bearing survival or tumor volume outgrowth (Figures S2D and S2E).

Staining for neutrophil markers Ly6G, myeloperoxidase (MPO), and NE validated our prior findings. Chi3l1-deficient lesions exhibited reduced neutrophil numbers, whereas Chi3l1 OE glands exhibited a marked increase in neutrophil content relative to wild-type (WT) controls (Figures S2F–S2I). Importantly, no differences in neutrophil numbers were observed in age-matched non-MIC WT, Chi3l1-deficient, or Chi3l1 OE mammary glands (Figures S2J–S2L), indicating that differences in neutrophil levels in these models occur specifically in the context of oncogene-induced tumorigenesis. Thus, these models provide complementary genetic tools to modulate neutrophil levels and investigate their role in DCIS progression. Correspondingly, Chi3l1-deficient MIC lesions demonstrated robust retention of myoepithelial markers, while Chi3l1 OE MIC lesions displayed substantial loss of myoepithelial marker expression and structural integrity compared to controls (Figures S3A–S3H). Analysis of proliferation markers revealed no significant differences between the three groups (Figures S3I–S3K). To determine whether loss of the myoepithelial layer observed in Chi3l1 OE MIC glands was neutrophil dependent, we assessed myoepithelial content in Chi3l1 OE mammary glands following anti-Ly6G treatment as validated in our earlier study.^25^ Anti-Ly6G treatment restored myoepithelial layer continuity in Chi3l1 OE MIC glands without impacting cancer cell proliferation, consistent with observations in WT MIC mice (Figures 1F–1J; Figures S3L, S3M, and S4A–S4C).

These phenotypes persisted at tumor endpoint; Chi3l1-deficient tumors retained significantly higher myoepithelial content, while Chi3l1 OE tumors exhibited myoepithelial loss (Figures S4D–S4I), indicating that neutrophil-driven loss of myoepithelium remains consistent throughout tumor progression. Consistent with the protective barrier function of the myoepithelium against tumor invasion and metastasis, Chi3l1-deficient mice developed significantly fewer and smaller lung metastases, whereas Chi3l1 overexpression increased both metastatic incidence and burden compared to WT controls (Figures 1K–1M). Cancer cell proliferation remained unchanged at mammary tumor endpoint (Figures S4J–S4L), indicating that the metastatic phenotype was independent of cancer cell proliferation. Together, these data demonstrate that Chi3l1-mediated neutrophil recruitment promotes myoepithelial barrier disruption and metastatic progression.

To further validate our findings in a basal GEMM, Chi3l1-deficient mice were crossed with the C3Tag GEMM (Figures S5A and S5B).^26^^,^^27^ Like the MIC model, Chi3l1 ablation in the C3Tag GEMM had no impact on disease-bearing survival, tumor outgrowth, or proliferation (Figures S5C–S5G). Consistent with the role of Chi3l1 in neutrophil recruitment, Chi3l1-deficient C3Tag tumors exhibited significantly reduced neutrophil infiltration compared with Chi3l1-proficient controls (Figures S5H–S5K). Notably, Chi3l1-deficient C3Tag tumors retained robust expression of myoepithelial markers, including α-SMA, calponin 1, and P63, compared to Chi3l1-proficient tumors (Figures S5L–S5P). Because C3Tag tumors exhibit a basal phenotype, CK14 was expressed by both tumor and myoepithelial cells, making it unsuitable for quantifying the myoepithelial compartment in end-stage tumors. These findings demonstrate that neutrophil-associated myoepithelial layer disruption is observed across genetically and molecularly distinct models of breast cancer.

### Neutrophil degranulation and NETosis promote myoepithelial layer disruption

Neutrophils exert effector functions through the release of proteases and granular contents via degranulation and through neutrophil extracellular traps (NETosis). To define which neutrophil function contributes to myoepithelial layer disruption, we selectively inhibited neutrophil degranulation or NETosis in the MIC GEMM and assessed myoepithelial integrity.

To inhibit neutrophil degranulation, MIC mice were treated with Nexinhib20 (Nex20), a selective inhibitor of degranulation.^28^ While Nex20 treatment did not impact overall neutrophil levels in the mammary gland (Figures S6A and S6B), it significantly reduced serum MPO and NE levels measured by ELISA (enzyme-linked immunosorbent assay), confirming effective inhibition of neutrophil degranulation (Figures S6C and S6D). Importantly, Nex20 treatment did not impact the levels of citrullinated H3 (CitH3), a selective NETosis marker, confirming the specificity of degranulation blockade (Figures S6E–S6I).

Nex20-treated glands exhibited significant retention of myoepithelial markers and preservation of myoepithelial architecture compared to vehicle-treated controls (Figures 2A–2E). Similar protection of the myoepithelium was observed in Chi3l1 OE MIC mice treated with Nex20 (Figures S6J–S6O), demonstrating that degranulation-dependent myoepithelial disruption occurred even in the context of elevated neutrophil infiltration. Together, these data indicate that neutrophil degranulation is a key contributor to myoepithelial barrier loss.

**Figure 2:**
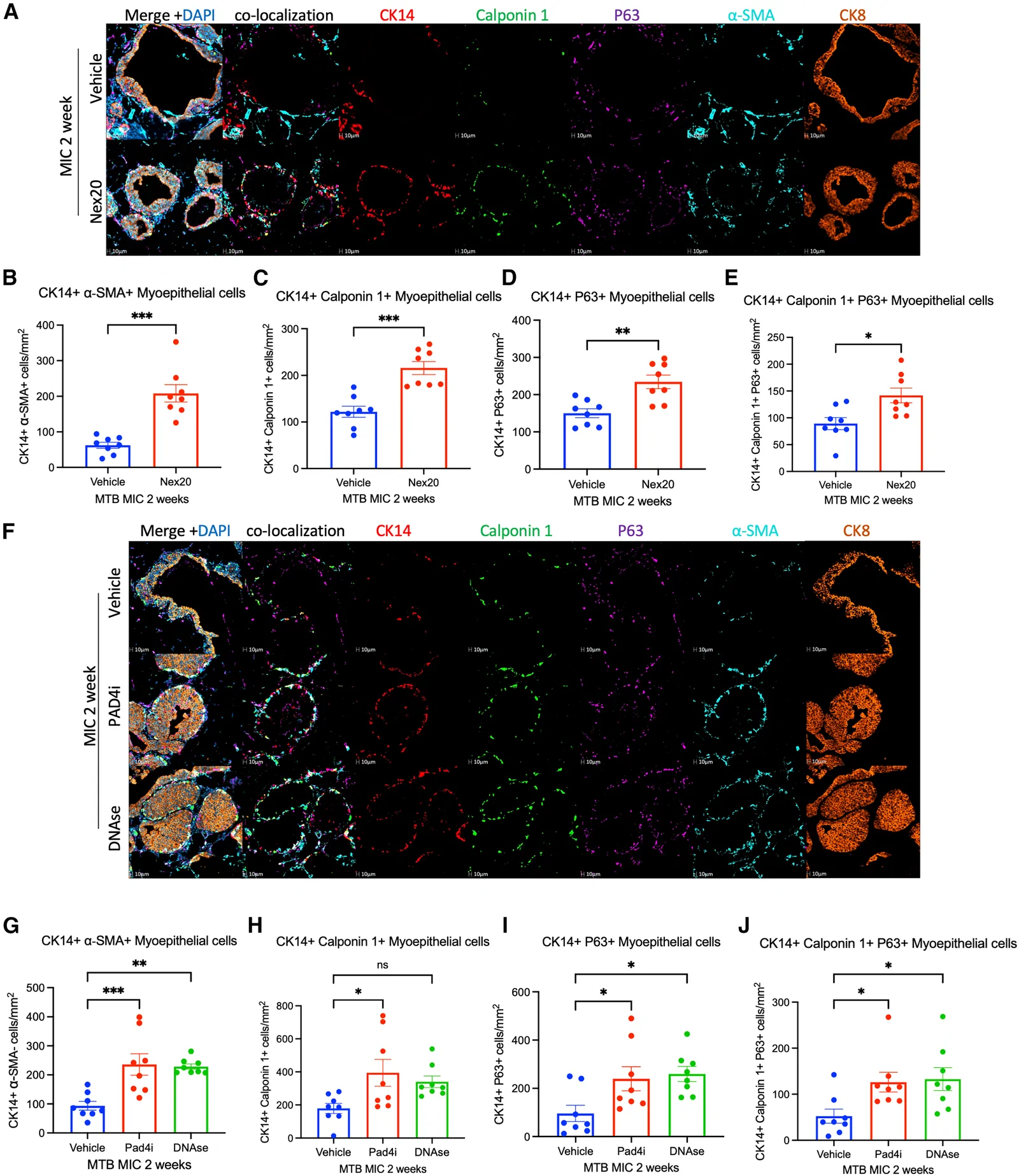
Targeting neutrophil degranulation or NETosis inhibits myoepithelial layer dissolution (A) Fluorescent IHC staining of mammary glands from vehicle or Nex20-treated WT MIC mice at 2 weeks post-induction using antibodies against CK14, α-SMA, calponin 1, P63, and CK8, counterstained with DAPI. (B–E) Quantification of total CK14^+^ α-SMA^+^ (Cohen’s d = −2.8082), CK14^+^ calponin 1^+^ (Cohen’s d = −2.5726), CK14^+^ P63^+^ (Cohen’s d = −1.9333), and CK14^+^ calponin 1^+^ P63^+^ (Cohen’s d = −1.4729) cells normalized to total analyzed area in vehicle (*n* = 8) and Nex20-treated (*n* = 8) MIC mammary glands at 2 weeks post-induction. Derived from the staining shown in (A). (F) Fluorescent IHC staining of mammary glands from vehicle, Pad4i, or DNase-treated WT MIC mice at 2 weeks post-induction using antibodies against CK14, α-SMA, calponin 1, P63, and CK8, counterstained with DAPI. (G–J) Quantification of total CK14^+^ α-SMA^+^ (eta squared: η2 = 0.52), CK14^+^ calponin 1^+^ (Eta squared: η2 = 0.29), CK14^+^ P63^+^ (Eta squared; η2 = 0.33), and CK14^+^ calponin 1^+^ P63^+^ (Eta squared; η2 = 0.3) cells normalized to total analyzed area in vehicle (*n* = 8), Pad4i (*n* = 8), and DNase-treated (*n* = 8) MIC mammary glands at 2 weeks post-induction. Derived from the staining shown in (F). Ns, not significant, ^∗^*p* < 0.05, ^∗∗^*p* < 0.01, ^∗∗∗^*p* < 0.001, and ^∗∗∗∗^*p* < 0.0001 calculated Mann-Whitney non-parametric test (for B–E) or one-way ANOVA with Dunnet’s post hoc test (for G–J). Data are presented as mean ± SEM. Each data point is an independent biological sample, and *N* represents the number of independent biological replicates. Analysis was performed by the HALO (Indica Labs) software. Quantification was performed on whole-slide scans of the entire mammary gland section. Scale bars: 10 μm.

NETosis represents a second neutrophil effector mechanism implicated in tumor progression.^24^^,^^29^ We had previously treated WT MIC mice with a peptidyl arginine deiminase 4 inhibitor (Pad4i, GSK484) to block NET formation, or DNase-1 to disrupt pre-existing NETs.^24^ In both settings, staining revealed a significant retention of myoepithelial markers compared to vehicle-treated glands (Figures 2F–2J). Collectively, these findings demonstrate that both neutrophil degranulation and NETosis independently contribute to myoepithelial layer disruption.

### NE promotes myoepithelial layer disruption

Because pharmacological inhibition of either neutrophil degranulation or NETosis preserved the myoepithelial layer, we hypothesized that a shared neutrophil-derived effector mediates myoepithelial disruption. NE, a serine protease released during both degranulation and NETosis, is a strong candidate mediator given its ability to remodel the TIME and degrade ECM components.^30^^,^^31^^,^^32^ Although elevated NE levels are associated with poor breast cancer patient outcomes,^33^ its role in DCIS progression has not been defined.

To functionally assess the role of NE in DCIS progression, we treated WT MIC mice with alvelestat (Alv), a selective NE inhibitor.^34^ Effective NE inhibition was confirmed by evaluating the levels of NE substrates, thrombospondin-1 (TSP1) and elastin, which are degraded by NE. Indeed, alvelestat-treated mammary glands exhibited significantly increased TSP1 and elastin levels (Figures S7A–S7D). Importantly, alvelestat treatment did not impact overall neutrophil levels or cancer cell proliferation in the mammary gland (Figures S7E–S7I), indicating that its effects were not due to changes in neutrophil recruitment or proliferation.

Furthermore, alvelestat-treated glands exhibited marked retention of myoepithelial markers and preservation of myoepithelial architecture compared to vehicle-treated controls (Figures 3A–3E; Figures S7J and S7K). These findings were also confirmed through fluorescent-activated cell sorting (FACS) where alvelestat treatment had no impact on overall neutrophil levels but significantly increased EPCAM^−^CD49f^+^CK5^+^CK14^+^ myoepithelial cells (Figures S7L–S7O). Similar protection of the myoepithelial layer was observed in Chi3l1 OE MIC mice treated with alvelestat, demonstrating that NE inhibition counteracted myoepithelial disruption even in the setting of elevated neutrophil infiltration (Figures S8A–S8F). To assess whether this phenotype was shared across GEMMs, WT C3Tag mice were treated with alvelestat at the DCIS stage. Consistently, alvelestat-treated C3Tag mammary glands exhibited significantly increased myoepithelial content compared with vehicle-treated controls (Figures S8G–S8J).

**Figure 3:**
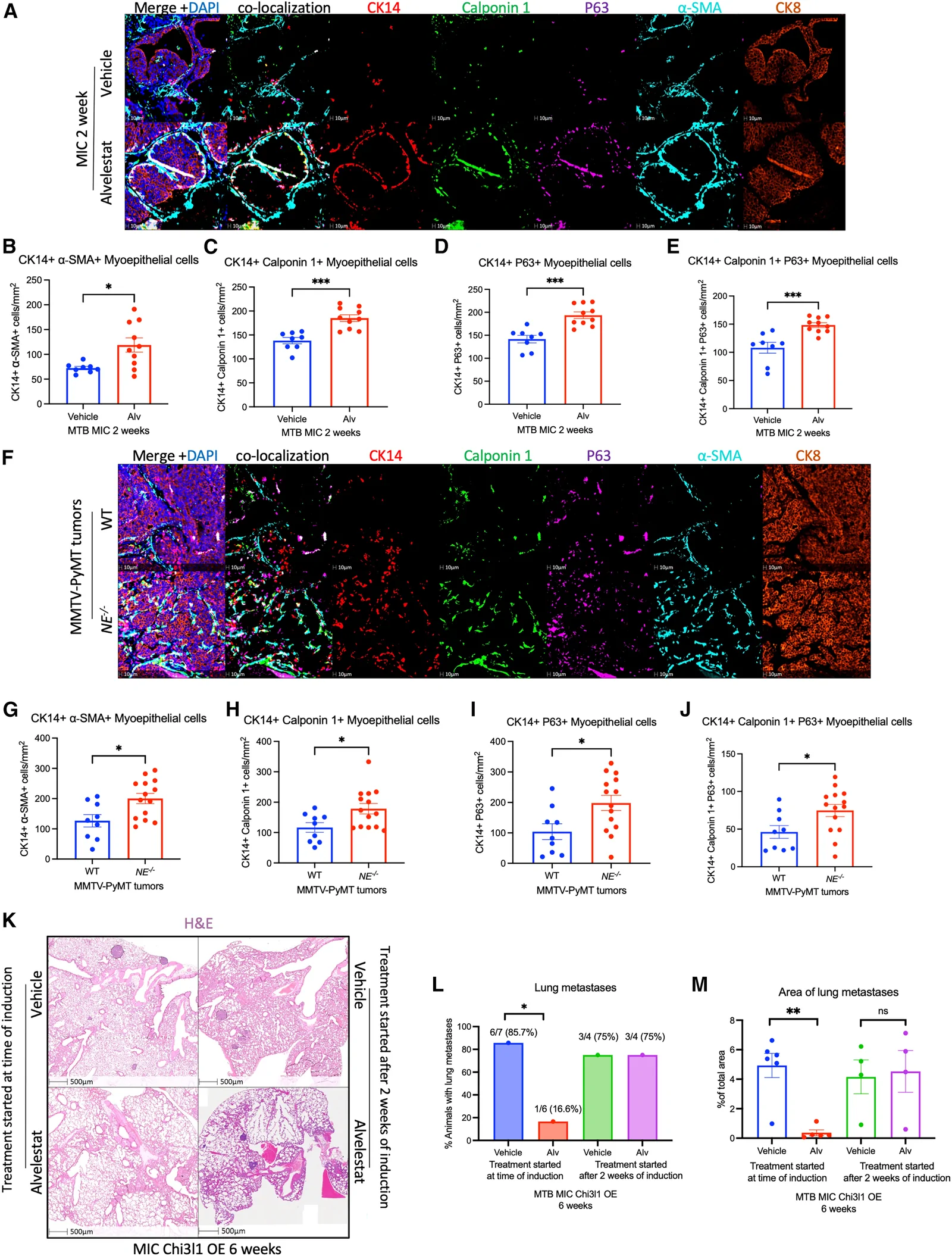
Targeting NE inhibits DCIS progression (A) Fluorescent IHC staining of mammary glands from vehicle or alvelestat-treated WT MIC mice at 2 weeks post-induction using antibodies against CK14, calponin 1, P63 α-SMA, and CK8 counterstained with DAPI. (B–E) Quantification of total CK14^+^ α-SMA^+^ (Cohen’s d = −1.3403), CK14^+^ calponin 1^+^ (Cohen’s d = −2.2712), CK14^+^ P63^+^ (Cohen’s d = −2.2540), and CK14^+^ calponin 1^+^ P63^+^ (Cohen’s d = −1.9551) cells normalized to total analyzed area in vehicle (*n* = 8) and alvelestat-treated (*n* = 10) MIC mammary glands at 2 weeks post-induction. Derived from the staining shown in (A). (F) Fluorescent IHC staining of WT and NE^−/−^ MMTV-PyMT end-stage mammary tumors using antibodies against CK14, calponin 1, P63, α-SMA, and CK8 counterstained with DAPI. (G–J) Quantification of total CK14^+^ α-SMA^+^ (Cohen’s d = −1.1790), CK14^+^ calponin 1^+^ (Cohen’s d = −1.0529), CK14^+^ P63^+^ (Cohen’s d = −1.0780), and CK14^+^ calponin 1^+^ P63^+^ (Cohen’s d = −1.0049) cells normalized to total analyzed area in WT (*n* = 9) and NE^−/−^ (*n* = 14) MMTV-PyMT mammary tumors. Derived from the staining shown in (F). (K) Representative H&E staining of Chi3l1 OE MIC lungs treated with vehicle or alvelestat for 6 weeks. On the left side are mice in which treatment was started from the time of induction (prior to myoepithelial disruption) and on the right are mice in which treatment was started after 2 weeks of induction (after the myoepithelial layer was disrupted). (L) Percentage of Chi3l1 OE MIC mice with pulmonary metastases after treatment with vehicle or alvelestat for 6 weeks. (M) Quantification of total pulmonary metastatic area in Chi3l1 OE MIC lungs after treatment with vehicle (*n* = 7) or alvelestat (*n* = 6) from the time of induction (Cohen’s d = 2.5843) or treated with vehicle (*n* = 4) or alvelestat (*n* = 4) after 2 weeks of induction. Both experiments were completed at 6 weeks of induction. Represented as percent of total lung area. ^∗^*p* < 0.05, ^∗∗^*p* < 0.01, ^∗∗∗^*p* < 0.001, and ^∗∗∗∗^*p* < 0.0001 calculated by Mann-Whitney non-parametric test, Fisher’s exact test (for L). Data are presented as mean ± SEM. Each data point is an independent biological sample, and *N* represents the number of independent biological replicates. Analysis was performed by the HALO (Indica Labs) software. Quantification was performed on whole-slide scans of the entire mammary gland section. Scale bars: 10 (A and F) and 500 μm (K).

Complementing our pharmacological studies, we evaluated myoepithelial content in breast tumors derived from NE-deficient (*NE*^−/−^) MMTV-PyMT mice.^35^ While NE deficiency does not alter primary tumor onset or growth, NE-deficient mice exhibit a marked reduction in lung metastases.^35^ Consistent with our alvelestat-treated mice, staining revealed that NE-deficient tumors retained significantly higher levels of myoepithelial markers, indicating preserved myoepithelial integrity compared to NE-proficient counterparts (Figures 3F–3J). Together, these pharmacologic and genetic approaches identify NE as a critical neutrophil-derived effector driving myoepithelial layer disruption.

We had previously demonstrated that targeting NETosis or neutrophil degranulation inhibits lung metastatic progression.^25^ Given the role of the myoepithelium in restraining invasion and metastatic dissemination, we investigated whether targeting NE would similarly suppress metastasis. Thus, we extended alvelestat treatment in Chi3l1 OE MIC mice to 6 weeks, a time point at which these mice develop lung metastases.^25^ NE inhibition significantly reduced the incidence and burden of lung metastases without impacting mammary tumor onset (Figures 3K–3M; Figures S9A and S9B). To determine whether this anti-metastatic effect was associated with the preservation of the myoepithelial barrier rather than a direct impact on metastatic outgrowth, we performed a second intervention study in which alvelestat treatment was initiated 2 weeks after doxycycline induction, a stage at which myoepithelial disruption had already occurred (Figure S3) and continued until week 6. In contrast to early intervention, delayed NE inhibition failed to reduce metastatic incidence or burden (Figures 3K–3M; Figure S9A). These findings argue that NE activity contributed to early metastatic dissemination and that NE inhibition is less effective once myoepithelial barrier disruption had already occurred.

Consistent with this interpretation, NE inhibition had no impact on anti-tumor immune responses. Staining revealed no differences in total CD8^+^ cytotoxic T cells, CD4^+^ T helper cells, or T cell activation status between vehicle- and alvelestat-treated glands (Figures S9C–S9H), indicating that the metastatic phenotype was not due to changes in the TIME. To further investigate whether NE contributed to tumor progression independently of its effects on the myoepithelial barrier, we treated tumor-bearing MIC mice with alvelestat from the time of palpable tumor onset until humane endpoint. Under these conditions, where the myoepithelial layer is already disrupted and invasive disease is established, NE inhibition had no effect on primary tumor growth, metastatic incidence, or burden (Figures S9I–S9M). Furthermore, we previously demonstrated that blood and metastatic lesions of WT and Chi3l1 OE mice exhibited comparable levels of neutrophil and NE^+^ cell infiltration, indicating that the increased metastatic burden in Chi3l1 OE mice is not associated with increased neutrophil accumulation or NE expression at the metastatic site.^25^

Together, these findings indicate that NE exerts its greatest effects during early disease progression and are consistent with preservation of myoepithelial integrity as major mechanisms underlying the anti-metastatic effects of NE inhibition in this model, rather than direct suppression of established tumors or metastatic lesions.

### NE-mediated ECM degradation induces anoikis of myoepithelial cells

We next sought to determine whether NE-mediated myoepithelial disruption was driven by loss of myoepithelial cells or by alterations in myoepithelial differentiation state. Studies have shown that progression from DCIS to IDC is accompanied by progressive myoepithelial de-differentiation, characterized by the sequential loss of differentiation markers including P63 and calponin 1.^36^^,^^37^ To investigate whether neutrophils regulate myoepithelial differentiation in our model, we re-analyzed our immunofluorescence data and quantified total CK14^+^ myoepithelial cells together with partially differentiated CK14^+^ P63^−^ and CK14^+^ calponin 1^−^ populations as a percentage of total CK14^+^ cells. Consistent with our previous findings, neutrophil depletion or inhibition of neutrophil degranulation significantly increased the abundance of total CK14^+^ myoepithelial cells (Figure S10A). However, the proportion of CK14^+^ P63^−^ and CK14^+^ calponin 1^−^ cells relative to total CK14^+^ cells remained unchanged between treatment groups (Figures S10A–S10F). These data demonstrate that neutrophils drive DCIS progression in our models by promoting myoepithelial cell loss rather than inducing myoepithelial de-differentiation.

Myoepithelial cells exist in a highly specialized physical niche, where their survival and function depend on direct adhesion to ECM components.^38^ Engagement of β1-integrins with laminin and collagen IV activates focal adhesion kinase (FAK) signaling,^39^ which is essential for myoepithelial survival and resistance to anoikis. Because NE is a serine protease known to degrade laminin and collagen IV, we hypothesized that NE-mediated ECM degradation disrupts integrin-dependent survival signaling, leading to myoepithelial cell death by anoikis.

To assess whether neutrophils regulate basement membrane integrity *in vivo*, we examined ECM composition in neutrophil-depleted mammary glands by staining for laminin and collagen IV. Anti-Ly6G-treated glands exhibited a significant increase in ECM components, as measured by the area occupied by laminin or collagen IV, compared to IgG2a-treated controls (Figures 4A–4C). Similar preservation of ECM components was observed following NE inhibition in both WT and Chi3l1 OE MIC mice (Figures 4D–4I).

**Figure 4:**
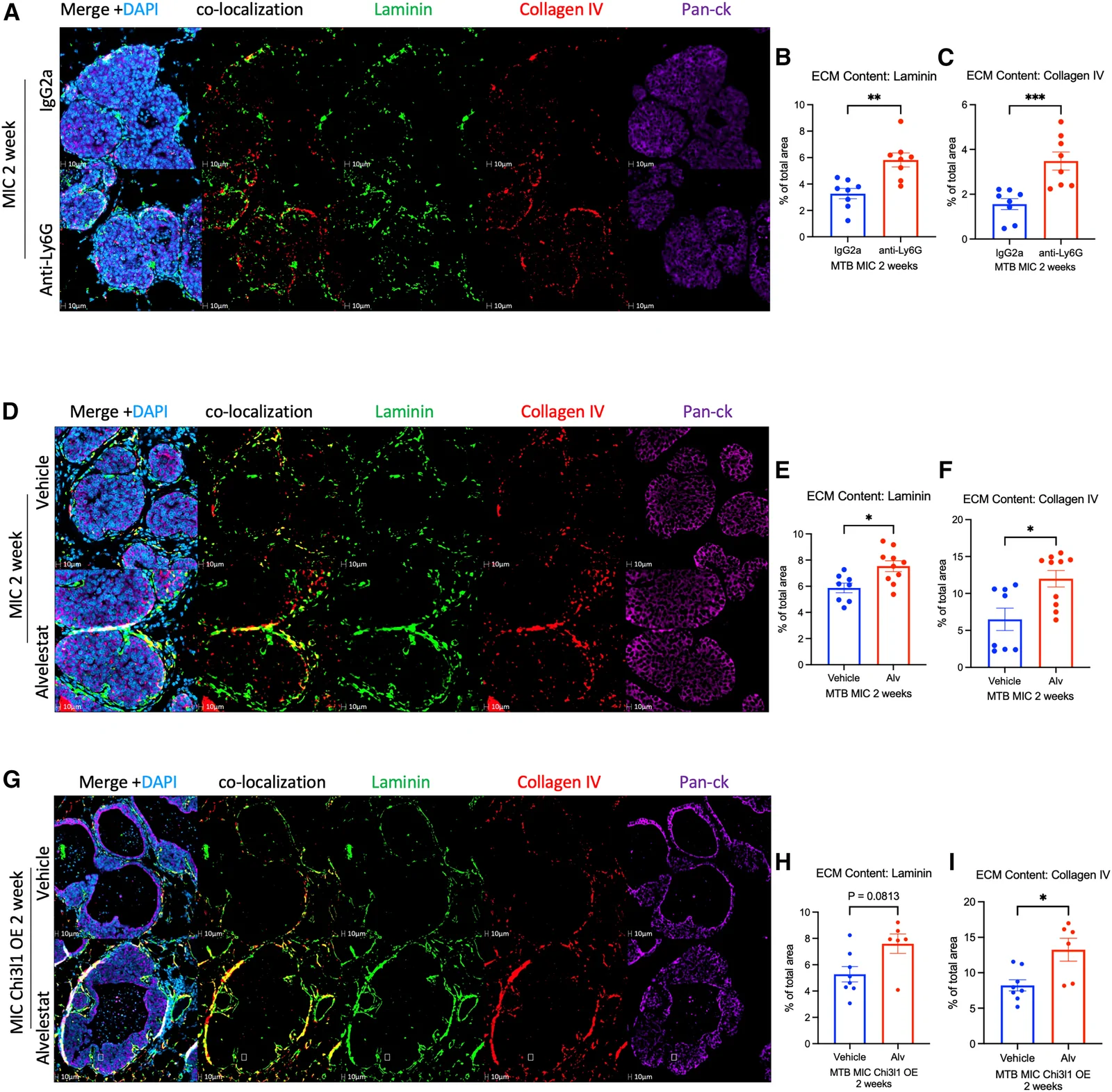
Targeting NE prevents ECM degradation (A) Fluorescent IHC staining of mammary tissue from WT MIC mice treated with anti-Ly6G or IgG2a isotype control for 2 weeks, using antibodies against laminin, collagen IV, and Pan-ck, counterstained with DAPI. (B and C) Quantification of area occupied by laminin (Cohen’s d = −1.9433) and collagen IV (Cohen’s d = −2.0424) in IgG2a (*n* = 8) and anti-Ly6G (*n* = 8)-treated WT MIC mammary glands. Represented as percent of mammary gland area. Derived from the staining shown in (A). (D) Fluorescent IHC staining of mammary tissue from WT MIC mice treated with vehicle or alvelestat for 2 weeks, using antibodies against laminin, collagen IV, and Pan-ck, counterstained with DAPI (blue). (E and F) Quantification of area occupied by laminin (Cohen’s d = −1.3939) and collagen IV (Cohen’s d = −1.4118) in vehicle (*n* = 8) and alvelestat (*n* = 10)-treated WT MIC mammary glands. Represented as percent of mammary gland area. Derived from the staining shown in (D). (G) Fluorescent IHC staining of mammary tissue from Chi3l1 OE MIC mice treated with vehicle or alvelestat for 2 weeks, using antibodies against laminin, collagen IV, and Pan-ck, counterstained with DAPI. (H and I) Quantification of area occupied by laminin (Cohen’s d = −1.3560) and collagen IV (Cohen’s d = −1.6446) in vehicle (*n* = 8) and alvelestat (*n* = 6)-treated Chi3l1 OE MIC mammary glands. All data represented as percent of mammary gland area. Derived from the staining shown in (G). ∗*p* < 0.05 and ∗∗*p* < 0.01 calculated by Mann-Whitney non-parametric test. Data are presented as mean ± SEM. Each data point is an independent biological sample, and *N* represents the number of independent biological replicates. Analysis was performed by the HALO (Indica Labs) software. Quantification was performed on whole-slide scans of the entire mammary gland section. Scale bars: 10 μm.

To independently validate the role of NE in ECM degradation, we performed *in vitro* ECM degradation assays. PyMT^+^ tumor cells were cultured for 12 h on a synthetic ECM layer in conditioned media derived from murine neutrophils treated with alvelestat or vehicle control. Staining revealed significantly increased laminin coverage when tumor cells were cultured in alvelestat-treated neutrophil media compared to vehicle-treated media, indicating loss of laminin degradation after inhibiting NE (Figures S11A and S11B). In parallel, PyMT^+^ cells were cultured on fluorescently labeled collagen IV matrices in conditioned media derived from murine neutrophils treated with alvelestat or vehicle control. This matrix utilizes quenched probes that only fluoresce green when collagen IV is cleaved. Exposure to vehicle-treated neutrophil-conditioned media resulted in marked fluorescence, indicative of collagen IV degradation, whereas alvelestat-treated neutrophil media caused a significant reduction in fluorescence, demonstrating impaired collagen IV degradation (Figures S11C and S11D).

As neutrophils release a variety of factors that remodel the ECM, we sought to validate their role in ECM degradation. Laminin and collagen IV degradation assays were repeated in the presence of either recombinant NE, recombinant Chi3l1, recombinant proteinase 3 (PR3), or recombinant cathepsin G. Consistent with our prior results, recombinant NE caused significant degradation of laminin and collagen IV, like the effects of neutrophil-conditioned media (Figures S11E–S11H). Recombinant cathepsin G and PR3 had no detectable effect on laminin or collagen IV coverage (Figures S11E–S11H), indicating that NE is the primary protease responsible for ECM degradation under these experimental conditions. Recombinant Chi3l1 also had no impact on the ECM, confirming that the phenotypes observed in Chi3l1 OE mice were driven by neutrophils and not due to the effects of Chi3l1 on the ECM (Figures S11E–S11H). To independently confirm the role of NE in ECM rewiring and cancer cell invasion, we performed an *in vitro* transwell invasion assay, culturing PyMT^+^ cells on a synthetic ECM in the presence of conditioned neutrophil medium treated with vehicle or alvelestat. While neutrophil-conditioned medium stimulated cancer cell invasion, this effect was reduced in media treated with alvelestat (Figures S11I and S11J). Collectively, these *in vivo* and *in vitro* data establish NE as a key mediator of basement membrane ECM degradation.

We next evaluated whether ECM degradation leads to myoepithelial cell anoikis. To assess this, we performed staining for cleaved caspase-3 (CCP3), a marker of cell death, and p-FAK (phosphorylated focal adhesion kinase), a readout of β1-integrin-ECM engagement. Both markers were combined with CK14 to assess their levels specifically in the myoepithelial compartment. In neutrophil-depleted glands, we observed a significant reduction in CCP3^+^ myoepithelial cells compared with controls, accompanied by a marked increase in p-FAK^+^ myoepithelial cells, without a change in cancer cell death (Figures S11K–S11O). These findings indicate that neutrophil depletion preserved integrin-mediated survival signaling and protected myoepithelial cells from anoikis.

We next assessed CCP3 and p-FAK expression in alvelestat-treated WT and Chi3l1 OE MIC mammary glands. In both models, NE inhibition caused a significant decrease in CCP3^+^ myoepithelial cells and a concomitant increase in p-FAK^+^ myoepithelial cells compared to vehicle-treated controls, while tumor cell death remained unchanged (Figures 5A–5J). These data demonstrate that NE-mediated ECM degradation disrupts integrin-dependent survival signaling, leading to myoepithelial cell anoikis.

**Figure 5:**
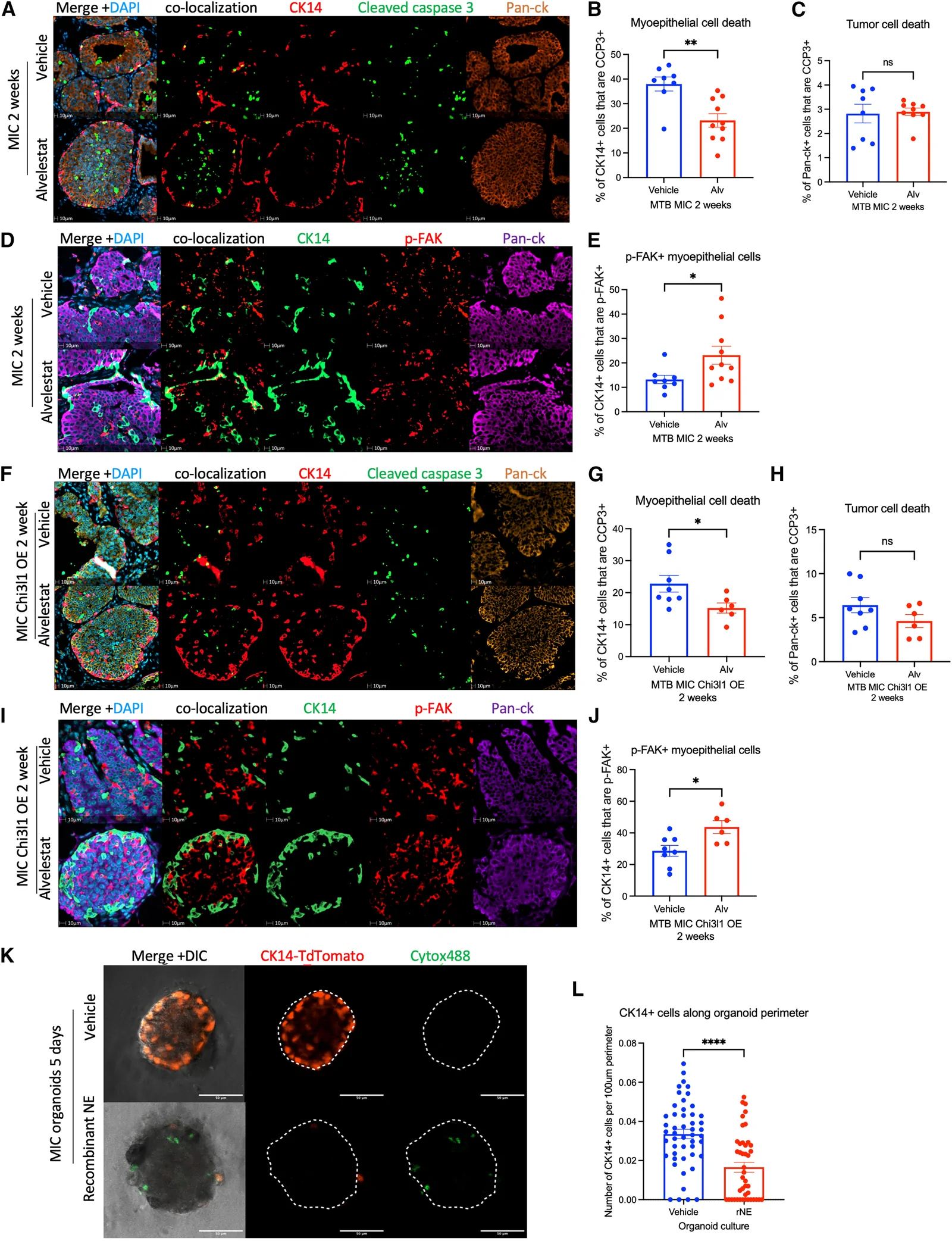
Targeting NE prevents myoepithelial layer anoikis (A) Fluorescent IHC staining of mammary tissue from WT MIC mice treated with vehicle or alvelestat for 2 weeks, using antibodies against CK14, cleaved caspase-3, and Pan-ck, counterstained with DAPI. (B) Quantification of the percentage of CK14^+^ cleaved caspase-3^+^ cells within total CK14^+^ cells in vehicle (*n* = 8) and alvelestat (*n* = 10)-treated WT MIC mammary glands (Cohen’s d = 1.7575). Derived from the staining shown in (A). (C) Quantification of the percentage of Pan-ck^+^ cleaved caspase-3^+^ cells within total Pan-ck^+^ cells in vehicle (*n* = 8) and alvelestat (*n* = 10)-treated WT MIC mammary glands (Cohen’s d = −0.0604). Derived from the staining shown in (A). (D) Fluorescent IHC staining of mammary tissue from WT MIC mice treated with vehicle or alvelestat for 2 weeks, using antibodies against CK14, p-FAK, and Pan-ck, counterstained with DAPI. (E) Quantification of the percentage of CK14^+^ p-FAK^+^ cells within total CK14^+^ cells in vehicle (*n* = 8) and alvelestat (*n* = 10)-treated WT MIC mammary glands (Cohen’s d = −1.0545). Derived from the staining shown in (D). (F) Fluorescent IHC staining of mammary tissue from Chi3l1 OE MIC mice treated with vehicle or alvelestat for 2 weeks, using antibodies against CK14, cleaved caspase-3, and Pan-ck, counterstained with DAPI. (G) Quantification of the percentage of CK14^+^ cleaved caspase-3^+^ cells within total CK14^+^ cells in vehicle (*n* = 8) and alvelestat (*n* = 6)-treated Chi3l1 OE MIC mammary glands (Cohen’s d = 1.2349). Derived from the staining shown in (F). (H) Quantification of the percentage of Pan-ck^+^ cleaved caspase-3^+^ cells within total Pan-ck^+^ cells in vehicle (*n* = 8) and alvelestat (*n* = 6)-treated Chi3l1 OE MIC mammary glands (Cohen’s d = 0.8193). Derived from the staining shown in (F). (I) Fluorescent IHC staining of mammary tissue from Chi3l1 OE MIC mice treated with vehicle or alvelestat for 2 weeks, using antibodies against CK14, p-FAK, and Pan-ck, counterstained with DAPI. (J) Quantification of the percentage of CK14^+^ p-FAK^+^ cells within total CK14^+^ cells in vehicle (*n* = 8) and alvelestat (*n* = 6)-treated Chi3l1 OE MIC mammary glands (Cohen’s d = −1.5316). Derived from the staining shown in (I). (K) Live fluorescent visualization of organoids grown from MIC Ck14-TdTomato mammary glands induced in culture for 5 days and treated with vehicle or recombinant neutrophil elastase for 24 h. Red indicates endogenous K14-driven TdTomato. Green indicates Incucyte Cytotox Dye, which marks dead cells. The white dotted line outlines the organoid seen by DIC. (L) Quantification of total CK14^+^ cells per 100 μm perimeter in NE (*n* = 43 organoids, technical replicate) or vehicle-treated (*n* = 49 technical replicate) organoids (Cohen’s d = 1.0088). Results are pooled from 3 technical replicates. Derived from the staining shown in (K). Ns, not significant, ∗*p* < 0.05, ∗∗*p* < 0.01, ∗∗∗*p* < 0.001, and ∗∗∗∗*p* < 0.0001 calculated by Mann-Whitney non-parametric test. Data are presented as mean ± SEM. Each data point is an independent biological sample, and *N* represents the number of independent biological replicates. Analysis was performed by the HALO (Indica Labs) software. Quantification was performed on whole-slide scans of the entire mammary gland section. Scale bars: 10 (A, D, F, and I) and 50 μm (K).

To further validate these findings, we stained for myoepithelial and neutrophil markers across a larger number of MIC mammary glands. Consistent with our model, NE levels were inversely correlated with the abundance of myoepithelial cells (Figures S12A–S12D). Importantly, NE levels were negatively correlated with laminin content and the number of p-FAK^+^CK14^+^ myoepithelial cells and positively correlated with CCP3^+^ myoepithelial cells (Figures S12E–S12I), which suggested an association between NE accumulation, loss of adhesion signaling, and induction of myoepithelial cell death. Spatial distance analysis further demonstrated that NE^+^ cells were preferentially localized near p-FAK^−^ myoepithelial cells and near CCP3^+^ myoepithelial cells (Figures S12J and S12K).

To validate our findings in an isolated *in vitro* system, we generated organoids from CK14-TdTomato-MIC mammary glands, in which myoepithelial cells are fluorescently labeled with TdTomato.^40^ Organoids were embedded in an ECM-rich three-dimensional matrix that preserves mammary duct-like architecture and myoepithelial organization. Treatment with recombinant NE resulted in significant loss of CK14^+^ myoepithelial cells after 24 h of treatment, whereas PBS-treated control organoids maintained an intact myoepithelial layer (Figures 5K and 5L; Figure S12L). These data demonstrate that NE is sufficient to drive myoepithelial cell loss independently of systemic factors and further support a direct role for NE in disrupting myoepithelial integrity.

Collectively, our *in vivo* and *in vitro* analyses define a mechanism through which neutrophil-derived NE degrades basement membrane ECM, compromises integrin-mediated survival signaling, and induces myoepithelial cell death through anoikis.

### DCIS patient samples with elevated NE exhibit myoepithelial barrier disruption

To validate our findings in a human model of DCIS progression, we analyzed mammary glands from the MCF7-DCIS mouse mammary intraductal (MIND)^41^^,^^42^ model containing either non-invasive DCIS lesions or lesions that had progressed to invasive disease. Staining revealed a marked increase in NE^+^ cells within invasive samples compared to non-invasive DCIS lesions (Figures S13A and S13B). Consistent with our findings in GEMMs, progression to invasive disease was accompanied by a profound loss of myoepithelium and laminin coverage (Figures S13A–S13D). These findings extend our observations to an independent human DCIS progression model and demonstrate that invasive transition is associated with increased NE accumulation, ECM degradation, and myoepithelial loss.

To assess the clinical relevance of our findings, we next examined neutrophil infiltration and myoepithelial integrity in human breast cancer. Staining for MPO as a neutrophil marker and CK14 was performed on breast cancer tissue microarrays encompassing normal breast tissue, DCIS, and IDC (Figure 6A). Consistent with prior reports,^20^ DCIS samples exhibited significantly higher neutrophil infiltration compared to normal breast tissue (Figures 6A and 6B). Notably, neutrophil abundance did not differ between DCIS and IDC samples, indicating that neutrophil accumulation occurs early during disease progression (Figures 6A and 6B). Importantly, quantitative analysis revealed an inverse correlation between neutrophil abundance and myoepithelial content within DCIS lesions (Figures 6A–6C). DCIS samples with elevated neutrophil infiltration exhibited marked disruption of the myoepithelium compared with neutrophil-low lesions (Figures 6A–6C). Importantly, this inverse correlation was observed in both estrogen receptor (ER)^+^ and ER^−^ DCIS lesions, indicating that the relationship is unlikely to be driven solely by ER status (Figures S13E and S13F).

**Figure 6:**
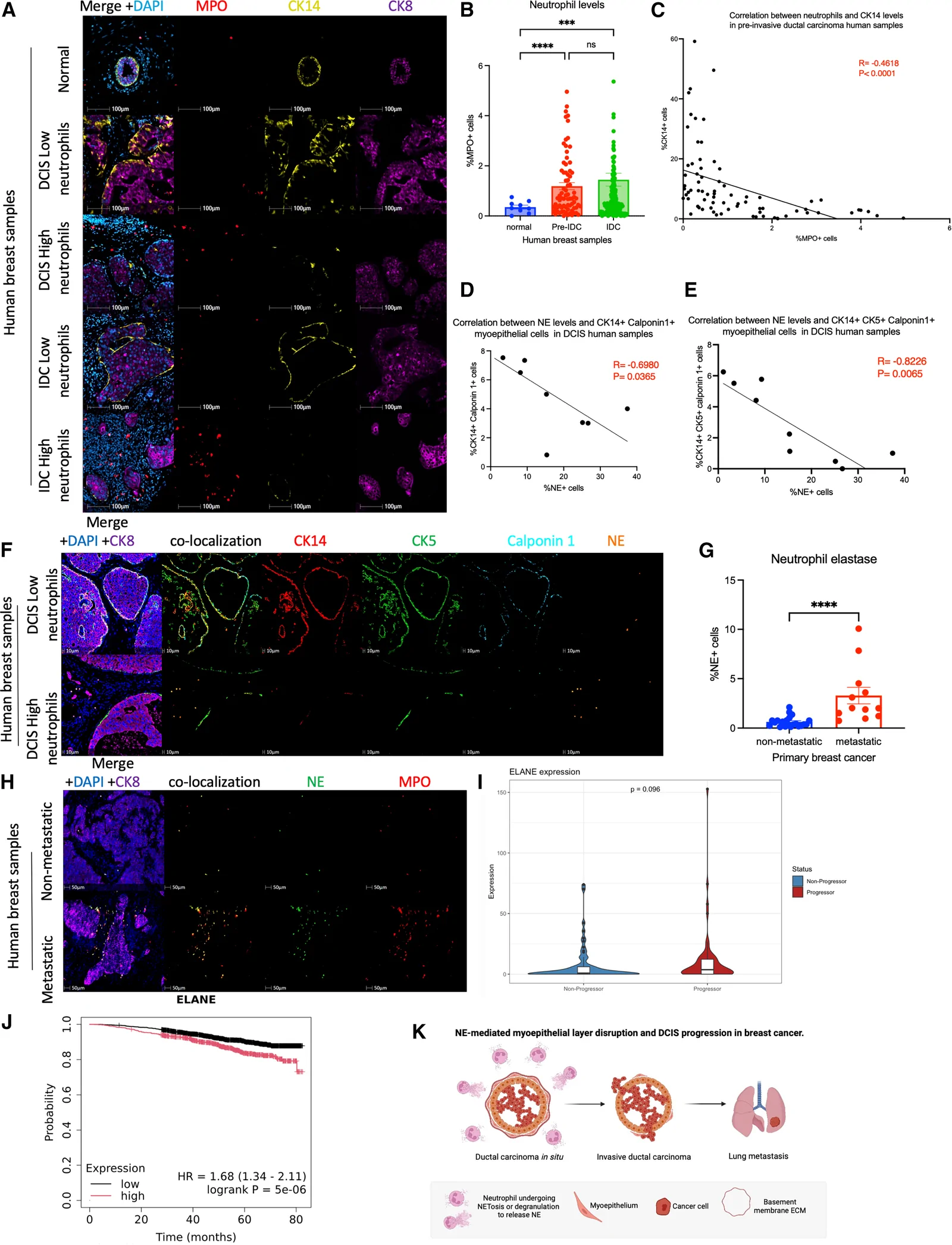
NE correlates with myoepithelial layer loss in human DCIS samples (A) Fluorescent IHC staining of human normal breast tissue, pre-invasive ductal carcinoma, and invasive ductal carcinoma samples using antibodies against MPO, CK14, and CK8, counterstained with DAPI. Pre-IDC samples include hyperplasia, atypia, and DCIS. Micrograph represents areas of high neutrophils and low neutrophils from different DCIS or IDC patient samples. (B) Quantification of total MPO^+^ cells as a percentage of total cells in normal (*n* = 9), DCIS (*n* = 75), and IDC (*n* = 207) human patient samples. Derived from the staining shown in (A). (C) Pearson correlation between neutrophils (MPO^+^ cells) and the total CK14^+^ cells in human DCIS samples (*n* = 75). Both presented as a percentage of total cells. Derived from the staining shown in (A). (D and E) Pearson correlation between NE^+^ cells and total CK14^+^ calponin 1^+^ cells or CK14^+^ CK5^+^ calponin 1^+^ cells in human DCIS samples (*n* = 9). All presented as a percentage of total cells. Derived from the staining shown in (F). (F) Fluorescent IHC staining of human DCIS samples using antibodies against NE, CK14, CK5, calponin 1, and CK8, counterstained with DAPI. Micrograph represents areas of high NE and low NE from different DCIS patient samples. (G) Quantification of total NE^+^ cells as a percentage of total cells in non-metastatic (*n* = 21) or metastatic (*n* = 12) breast cancer samples (Cohen’s d = −1.4931). Derived from the staining shown in (H). (H) Fluorescent IHC staining of human non-metastatic or metastatic breast cancer samples using antibodies against NE, MPO, and CK8, counterstained with DAPI. (I) Expression levels of *ELANE* (NE) in human DCIS samples that progressed to invasive disease compared to DCIS samples that did not progress, analyzed using the Wilcoxon rank-sum test. (J) Elevated levels of *NE* are associated with decreased overall survival in breast cancer patients of all subtypes at 5 years post-diagnosis (*n =* 2,976). (K) Summary of the proposed model for NE-mediated myoepithelial layer disruption and DCIS progression. Neutrophils release NE during NETosis or degranulation, which degrades the ECM, leading to myoepithelial cell death by anoikis. This marks the transition to invasive disease that can metastasize to distant organs. Created with BioRender.com. Ns, not significant, ^∗∗^*p* < 0.01, ^∗∗∗^*p* < 0.001, and ^∗∗∗∗^*p* < 0.0001 calculated by Brown-Forsythe and Welch ANOVA tests with Dunnett’s T3 multiple comparison post hoc test (for B) or Mann-Whitney non-parametric test (for G). Data are presented as mean ± SEM. Pearson correlation was performed using two-tailed *t* test. Each data point is an independent biological sample, and *N* represents the number of independent biological replicates. Analysis was performed by the HALO (Indica Labs) software. Quantification was performed on whole-slide scans of the entire mammary gland section. Scale bars: 100 (A) and 10 μm (F andH).

Given our experimental data identifying NE as a mediator of myoepithelial disruption, we next stained an independent cohort of DCIS samples for NE and myoepithelial markers. Consistent with our mechanistic findings, elevated NE expression strongly correlated with reduced myoepithelial levels and compromised myoepithelial architecture in DCIS lesions (Figures 6D–6F). Importantly, NE levels did not differ between grade II and grade III DCIS samples, indicating that the phenotype was independent of tumor grade (Figure S13G). To determine whether the relationship between NE accumulation, loss of focal adhesion signaling, and myoepithelial cell death was also evident in human disease, we stained for CK14, p-FAK, CCP3, and NE in human DCIS specimens. Consistent with our findings in GEMMs, regions exhibiting elevated NE levels displayed reduced numbers of p-FAK^+^ myoepithelial cells and increased CCP3^+^ myoepithelial cells (Figures S13H–S13O). Quantitative analysis revealed a significant negative correlation between NE abundance and p-FAK^+^ myoepithelial cells and a positive correlation between NE abundance and apoptotic CCP3^+^ myoepithelial cells (Figures S13H–S13O). Spatial analysis further demonstrated that NE^+^ cells were preferentially localized near p-FAK^−^ and CCP3^+^ myoepithelial cells (Figures S13M and S13O). Interestingly, NE^+^ cells were also localized near regions of non-myoepithelial cell death (Figure S13P), suggesting that NE-associated activity may extend beyond myoepithelial disruption and contribute to localized tissue damage. Together, these findings provide independent validation in human DCIS that local NE accumulation is associated with loss of focal adhesion signaling and induction of myoepithelial cell death, supporting a conserved mechanism of NE-mediated myoepithelial barrier disruption during breast cancer progression.

To correlate our findings in the clinical setting, we analyzed independent patient cohorts and publicly available transcriptomic datasets. Immunofluorescence staining of primary breast cancer specimens revealed significantly higher NE levels in tumors from patients who subsequently developed metastatic disease compared with tumors from patients who remained metastasis-free (Figures 6G and 6H). To extend these findings to pre-invasive disease, we interrogated a publicly available DCIS progression dataset^43^ and observed a trend toward increased *NE* expression in DCIS lesions that subsequently progressed to invasive disease compared with non-progressing lesions, although this difference did not reach statistical significance (Figure 6I). Given the low abundance and spatially restricted nature of neutrophils in DCIS lesions, bulk transcriptomic analyses may underestimate localized NE activity within the tumor microenvironment.

Finally, Kaplan-Meier survival analyses demonstrated that elevated *NE* expression was associated with poorer survival outcomes in breast cancer patients (Figure 6J). To determine whether this association reflected a broader neutrophil abundance signature, we examined the prognostic associations of additional neutrophil-associated markers. While some markers, including *MPO*, *CXCR2*, and *S1*00*A8*, were associated with poorer overall survival, *S100A9* was associated with improved survival, whereas *CXCR1* and *PRTN1* did not show significant associations (Figures S14A–S14G). These divergent associations suggest that the prognostic relationship observed for *NE* is not simply attributable to overall neutrophil abundance and may instead reflect distinct neutrophil functional states or programs.

Collectively, analyses of human DCIS samples support our *in vivo* findings and suggest a potential association between neutrophil-derived NE and myoepithelial barrier disruption during DCIS progression. Mechanistically, NE promotes ECM degradation, which leads to myoepithelial cell death through anoikis and breast cancer progression (Figure 6K).

## Discussion

DCIS is a commonly diagnosed early stage of breast cancer that is marked by the presence of an intact myoepithelial layer.^1^^,^^3^ This layer acts as a physical and biochemical barrier that restricts tumor cell invasion.^3^^,^^11^ Loss of myoepithelial integrity is among the strongest histopathological predictors of invasion in DCIS and poor outcomes.^36^^,^^44^^,^^45^ Yet the cellular and molecular mechanisms driving this process remain poorly understood, limiting our ability to identify patients at risk of progression and develop mechanism-based preventive strategies. Using complementary GEMMs and human DCIS samples, we identified neutrophil-derived NE as a key mediator of myoepithelial layer disruption and DCIS progression.

Myoepithelial disruption is likely multifactorial, involving altered differentiation, stromal remodeling, and inflammatory signaling.^9^^,^^10^^,^^46^^,^^47^ Previous studies have associated loss of differentiation markers P63, calponin, and α-SMA with microinvasion.^36^^,^^37^ Our findings complemented these models by identifying a distinct mechanism whereby neutrophils promoted physical loss of myoepithelial cells through protease-mediated ECM degradation and anoikis. Consistent with this interpretation, targeting neutrophils or NE increased total myoepithelial abundance without altering the proportion of differentiated myoepithelial subsets. These findings support a stepwise model in which localized NE degrades the ECM, disrupts β1-integrin signaling, induces myoepithelial anoikis, and facilitates invasive transition.

Emerging evidence argues that tumor-extrinsic factors within the TIME play a decisive role in determining DCIS behavior.^48^^,^^49^ Multiple stromal populations, including macrophages, lymphocytes, and fibroblasts, have been implicated in invasive progression through effects on epithelial behavior, stromal remodeling, and ECM organization.^9^^,^^20^^,^^46^^,^^50^^,^^51^ Immune infiltration patterns have been associated with aggressive DCIS and increased risk of progression.^12^^,^^46^^,^^50^^,^^52^ However, the mechanisms through which immune cells promote myoepithelial barrier disruption have remained poorly defined. Our findings identify neutrophils as active mediators of this process through localized release of NE. Future studies defining the spatial and temporal contributions of other immune populations will further clarify how the TIME regulates invasive progression.

NE is a neutrophil-derived serine protease that modulates the TIME, degrades ECM components, and is associated with poor prognosis.^32^^,^^53^ While neutrophils have been observed in DCIS patient samples,^20^ their functional contribution to myoepithelial disruption has remained largely unexplored. Here, using complementary genetic, pharmacological, and clinical samples, we identified NE as a critical mediator of myoepithelial disruption and invasive progression. Given the availability of selective NE inhibitors with established safety profiles in clinical trials,^54^^,^^55^ these findings provide a rationale for investigating whether NE inhibition may have utility in early-stage DCIS.

Elevated neutrophil abundance is associated with poor patient outcomes and immunosuppression across multiple cancer types.^13^^,^^17^ However, direct therapeutic depletion of neutrophils is not clinically feasible because of their essential role in host defense. Targeting tumor-enriched neutrophil-derived effector molecules, such as NE, or upstream tumor-derived chemoattractants that regulate neutrophil recruitment may represent more selective therapeutic strategies. Importantly, neutrophils release numerous proteases and collagenases capable of degrading the ECM, which may also contribute to myoepithelial disruption. Defining the relative contribution of these proteases during DCIS progression may uncover additional therapeutic opportunities and improve our understanding of neutrophil biology in early breast cancer.

Although neutrophils were relatively sparse within mammary lesions, their biological impact is determined by localized protease release rather than absolute abundance. Unlike many immune populations that act primarily through cell-cell interactions, neutrophils release highly potent proteases capable of sustained ECM degradation following degranulation.^13^^,^^56^ Furthermore, neutrophils are notoriously difficult to capture using conventional transcriptomic approaches due to their short half-life and susceptibility to processing-induced loss.^57^^,^^58^^,^^59^ Our findings indicate that the spatial localization of neutrophils may be more informative than their overall abundance. NE^+^ neutrophils were observed in both intratumoral and peritumoral regions but preferentially localized near sites of myoepithelial disruption, supporting a model in which localized protease release directly compromises barrier integrity. Despite their relatively low abundance, targeting neutrophils or NE preserved myoepithelial integrity and suppressed disease progression. Future studies should determine whether spatial analysis of NE-positive cells in DCIS biopsy samples can improve risk stratification in DCIS.

While Chi3l1 was used in the present study primarily as a genetic platform to modulate neutrophil recruitment, these findings should be viewed within the broader biology of the Chi3l1 pathway. We previously demonstrated that STAT3 (signal transducer and activator of transcription 3) is a direct transcriptional regulator of Chi3l1, with chromatin immunoprecipitation confirming STAT3 binding to the Chi3l1 promoter and genetic Stat3 ablation markedly reducing Chi3l1 expression and neutrophil recruitment.^24^^,^^25^ Furthermore, Chi3l1 deficiency broadly remodels the TIME by enhancing T cell infiltration and activation, a phenotype that was phenocopied by neutrophil depletion, indicating that these immunologic effects are largely neutrophil dependent.^24^ Interestingly, NE inhibition preserved myoepithelial integrity without altering T cell abundance or activation, indicating that neutrophils promote breast cancer progression through at least two distinct mechanisms: an NE-dependent pathway driving ECM degradation and myoepithelial disruption and an NE-independent pathway regulating immune remodeling. Defining the mediators responsible for this second program represents an important area for future investigation.

In summary, our findings identify neutrophils and NE as key regulators of myoepithelial barrier integrity during DCIS progression. Neutrophil degranulation and NE release promote ECM degradation, impair β1-integrin signaling, and trigger myoepithelial anoikis, thereby facilitating invasive transition. These findings establish a mechanistic link between inflammation and myoepithelial layer disruption and support further investigation of neutrophil-derived proteases during early breast cancer progression.

### Limitations of the study

Several limitations of this preclinical study should be considered. Although our data identified NE as a mediator of myoepithelial disruption, neutrophils release numerous additional proteases and inflammatory mediators that may also contribute.^53^^,^^60^ Future studies should define the relative contribution of these pathways, determine the temporal window during which neutrophil activity irreversibly compromises myoepithelial integrity, and investigate how Chi3l1 regulates neutrophil activation, mobilization from the bone marrow, and NE release.

An additional limitation relates to the clinical evaluation of NE as a biomarker of DCIS progression. Although patient cohorts supported an association between elevated NE and disease progression, larger clinically annotated cohorts incorporating spatial proteomics, multiplex imaging, circulating biomarkers, and long-term follow-up will be required to establish the prognostic utility of NE. Functional validation in additional human DCIS models will further strengthen translational relevance. Addressing these questions will clarify the biological and potential clinical significance of NE during DCIS progression.

## Resource availability

### Lead contact

Further information and requests for resources and reagents should be directed to and will be fulfilled by the lead contact, William J. Muller (william.muller@mcgill.ca).

### Materials availability

All unique reagents or mouse models used in this study may be made available upon request from the lead contact with a completed materials transfer agreement.

### Data and code availability

• This paper analyzes existing, publicly available data, accessible at the Gene Expression Omnibus GSE281303.^43^ All data reported in this paper will be shared by the lead contact upon request.
• This paper does not report original code.
• Any additional information required to reanalyze the data reported in this paper is available from the lead contact upon request.

## Acknowledgments

We thank the Histology, Transgenic, Flow Cytometry, Bioinformatics, Microscopy, and Animal Husbandry Core Facilities at the Goodman Cancer Institute, McGill University, and Mitra Cowen from the McGill Integrated Core for Animal Modeling. T.T. is supported by Vanier Canada Graduate Scholarship (10.13039/501100000024CIHR; CGV-192738). Y.G. is supported by 10.13039/501100000024CIHR Canada Graduate Scholarship–Doctoral. A.M. is supported by the Donner Foundation Studentship Award (Goodman Cancer Institute, 10.13039/100008582McGill University), the Rolande and Marcel Gosselin Graduate Fellowship, and the Carole Epstein Fellowship in Health (10.13039/100008644Faculty of Medicine, McGill University). X.M. is supported by a Fonds de Recherche du Québec–Santé Master's Research Scholarship. C.D.E. is supported by a Canada Graduate Scholarship–Master’s. E.S. is supported by a Cancer Research Society Doctoral Research Award. The Breast Cancer Functional Genomics Group (BCFGG) Biobank at the Goodman Cancer Institute/10.13039/100014131MUHC is supported by the Réseau de Recherche en Cancer of the Fonds de Recherche du Québec–Santé and the 10.13039/100016328Quebec Breast Cancer Foundation and is certified by the Canadian Tumor Repository Network (CTRNet). W.J.M. is a Distinguished James McGill Professor and Canada Research Chair in Molecular Oncology. This work was supported by the Terry Fox Research Institute Program Project Grant (P.M.S. and W.J.M.); the CIHR Foundation (148373), Bridge (PLL-185690) and Project (PJT-190027) grants; the Canadian Cancer Society Impact Grant; and the 10.13039/100009326Cancer Research Society (1281449).

## Author contributions

Conceptualization, T.T., Y.G., and W.J.M.; investigation, T.T., Y.G., A.M., X.M., C.D.E., D.Z., V.S.-G., A.B., A.-L.G.-P., M.A., B.X., E.S., H.P., S.S.A., and A.J.N.; analysis, T.T., Y.G., A.M., X.M., C.D.E., V.S.-G., A.B., A.-L.G.-P., and L.M.; resources, M.A., H.K., P.M.S., K.K., L.M., and M.P.; writing – original draft, T.T. and W.J.M.; review and editing, T.T., Y.G., A.M., X.M., C.D.E., D.Z., E.S., B.X., H.P., S.S.A., H.K., P.M.S., K.K., M.P., and W.J.M.; supervision, W.J.M.; and funding acquisition, W.J.M.

## Declaration of interests

At the time of publication, S.S.A. is an employee of Genentech, a member of Roche Group.

## STAR★Methods

### Key resources table

**Table:** 

REAGENT or RESOURCE	SOURCE	IDENTIFIER
**Antibodies**		

Rat IgG2a, 2A3	Bio X cell	Cat# BE0089; RRID: AB_1107769
Anti-Ly6G, 1A8	Bio X cell	Cat# BE0075-1; RRID: AB_1107721
Chi3l1	Invitrogen	Cat# PA5-81356; RRID: AB_2788571
CitH3 (western blot)	Abcam	Cat# Ab5103; RRID: AB_304752
TSP1	Cell Signaling Technology	Cat# 37879S
β -actin	Sigma Aldrich	Cat# A5441; RRID: AB_476744
Vinculin	Millipore	Cat# MAB3574; RRID: AB_2304338
α-tubulin	Cell signaling	Cat# 3873; RRID: AB_1904178
CK14	Ventana	Cat# 760-4805; RRID: AB_2935819
CK5	Biolegend	Cat# 905504; RRID: AB_3095835
Calponin 1	Cell Signaling Technology	Cat# 17819; RRID: AB_2798789
P63	Santa Cruz	Cat# sc25268; RRID: AB_628092
α-SMA	DAKO	Cat# M0851; RRID: AB_2313736
Ly6G	Cell Signaling Technology	Cat#87048; RRID: AB_2909808
MPO	Abcam	Cat# ab208670; RRID: AB_2864724
CitH3 (Staining)	Cell Signaling Technology	Cat# 26111
Mouse NE	Cell Signaling Technology	Cat# 90120; RRID: AB_3096401
Human NE	Cell Signaling Technology	Cat# 28162
Laminin	Abcam	Cat# ab11575; RRID: AB_298179
Collagen IV	Abcam	Cat# ab6586; RRID: AB_305584
Elastin	BIOS USA	Cat# bs-1756R; RRID: AB_10856940
Cleaved caspase 3	Cell Signaling Technology	Cat# 9661; RRID:AB_2341188
*p*-FAK	Invitrogen	Cat# 44-624G
CK8	Biolegend	Cat# 904804; RRID: AB_2616821
CD3	Abcam	Cat# ab16669; RRID: AB_443425
CD8	Cell Signaling Technology	Cat# 98941; RRID: AB_2756376
CD4	Cell Signaling Technology	Cat# 25229; RRID: AB_2798898
PD1	Cell Signaling Technology	Cat# 84651; RRID: AB_2800041
Granzyme B	Cell Signaling Technology	Cat# 44153; RRID: AB_2857976
Ki67	Cell Signaling Technology	Cat# 12202; RRID: AB_2620142
PCNA	Cell Signaling Technology	Cat# 2586; RRID: AB_2160343
Pan-ck	Ventana	Cat# 760-2595; RRID: AB_2941938
CD45	BD Bioscience	Cat# 564225; RRID: AB_2716861
CD11b	Biolegend	Cat# 101201; RRID: AB_312784
Ly6G	Biolegend	Cat# 127606; RRID: AB_1236494
F4/80	Biolegend	Cat# 123114; RRID: AB_893478
Epcam	Biolegend	Cat# 118233; RRID: AB_2632775
CD49f	Biolegend	Cat# 313622; RRID: AB_2561705
CK5 (FACS)	Santa Cruz	Cat# sc-32721; RRID: AB_627852
CK14 (FACS)	Santa Cruz	Cat# sc-53253; RRID: AB_2134820
Viability dye eFluorYM 506	ThermoFisher/eBioscience	Cat# 65-0866-14
ImmPRESS HRP Anti-Mouse IgG Polymer detection kit	Vector Laboratories	Cat# VECTMP745250
ImmPRESS HRP Anti-Rabbit IgG Polymer detection kit	Vector Laboratories	Cat# VECTMP740150
Goat anti-rabbit secondary antibody Alexa Fluor 555	Invitrogen	Cat# A21428
Alexa Fluor plus 555 phalloidin	Invitrogen	Cat# A30106

**Biological samples**		

Fetal Bovine serum (FBS)	Wisent Inc.	Cat#080-150
Bovine pituitary extract	Hammond Cell Tech	Cat#1078-NZ
Breast hyperplasia tissue array	Tissuearray.com (USBiomax)	Cat#BR806
Breast cancer with matched breast tissue array	Tissuearray.com (USBiomax)	Cat# BR251
Advanced stage breast cancer and normal breast tissue array	Tissuearray.com (USBiomax)	Cat#BR1191
Multiple breast disease with NAT breast tissue array	Tissuearray.com (USBiomax)	Cat#1503
Breast cancer tissue array with adjacent normal breast tissue	Tissuearray.com (USBiomax)	Cat#BR8011
Breast tumor tissue array	Tissuearray.com (USBiomax)	Cat#20811
Mid-advanced stage breast cancer and normal tissue array	Tissuearray.com (USBiomax)	Cat#8013
Breast cancer tissue array (mostly ductal carcinoma *in situ*, DCIS)	Tissuearray.com (USBiomax)	Cat#301

**Chemicals, peptides, and recombinant proteins**		

doxycycline	Wisent Inc.	Cat# 450-185
GSK484 PAD4i	Medchem express	Cat# HY-100514
DNAse I	Roche	Cat#4536282001
Nexinhib20	Medchem express	Cat# HY-125792
Alvelestat	Medchem express	Cat# HY-15651
Hydrosortisone	Sigma	Cat#H4001
Recombinant human EGF	Wisent Inc.	Cat#511-110-CU
Recombinant human insulin	Wisent Inc.	Cat#511-016-UG
Recombinant mouse Chi3l1	R&D	Cat#2649-CH-050
Recombinant mouse Cathpesin G	Biomatik	Cat#RPU51859
Recombinant mouse PR3	Biomatik	Cat#RPU43519
Recombinant mouse NE	R&D	Cat#4517-SE
Cultrex Growth Factor Basement Membrane Extract	R&D	Cat# 3433-010-01
DQ collagen, type IV from human placenta, fluorescein conjugate	Invitrogen	Cat# D12052
Geltrex	Geltrex	Cat#A1413202
Critical commercial assays	R&D system	Cat# 4517-SE
Incucyte Cytotox 488 reagent	Sartorius	Cat#4633
DRAQ5 (Thermofisher#)	Thermofisher	Cat#62251
Tyramide signal amplification substrates_ OPAL480	Akoya biosciences	Cat#OP-001000
Tyramide signal amplification substrates_ OPAL520	Akoya biosciences	Cat# OP-001001
Tyramide signal amplification substrates_ OPAL570	Akoya biosciences	Cat#OP-001003
Tyramide signal amplification substrates_ OPAL620	Akoya biosciences	Cat#OP-001004
Tyramide signal amplification substrates_ OPAL690	Akoya biosciences	Cat#OP-001006
Tyramide signal amplification substrates_ OPAL780	Akoya biosciences	Cat#OP-001008
RNAscope 2.5 HD Assay-RED kit	Advanced Cell Diagnostics	Cat#322360
10× Casein Solution-Blocking buffer	Vector Laboratories	Cat#SP-5020-250
Antigen Unmasking Solution, Citrate-Based PH6	Vector Laboratories	Cat#H-3300-250
Antigen Unmasking Solution, Tris-Based PH9	Vector Laboratories	Cat#H-3301-250
BD Cytofix/Cytoperm fixation/permeabilization	BD Bioscience	Cat#554714
NE ELISA	R&D	Cat# DY4517-05
MPO ELISA	R&D	Cat# DY3667
MycoAlert Kit	Lonza	Cat# LT07-118

**Deposited data**		

Published human DCIS RNAseq	Debeljak et al.^43^	GEO: GSE281303

**Experimental models: Cell lines**		

Mouse: MMTV-PyMT	Huck et al.^61^	N/A
Mouse: Primary neutrophils	Hsu et al.^62^	N/A
Mouse organoids: MIC CK14-TdTomato	This paper	N/A
Mouse; 4T1	Hsu et al.^62^	N/A
Human: MCF-7	ATCC	Cat# HTB-22

**Experimental models: Organisms/strains**		

Mouse: MMTV-rtTA (MTB)	Gunther et al.^63^	N/A
Mouse: TetON-MIC	Rao et al.^21^	N/A
Mouse: Chi3l1^-/-^	Taifour et al.^24^^,^^25^	N/A
Mouse: Chi3l1 OE	Taifour et al.^24^^,^^25^	N/A
Mouse: C3Tag	Jackson lab	Cat#013591
Mouse: CK14-TdTomato	Vallmajo-Martin et al.^40^	N/A
Mouse: NOD-scid IL2Rgammanull (NSG)	Jackson lab	Cat#005557
Mouse: Balb/c	Jackson lab	Cat#000651

**Oligonucleotides**		

Chi3l1 RNAscope probe	Advanced Cell Diagnostics	Cat#449621

**Software and algorithms**		

HALO	Indica lab	https://www.indicalab.com/halo/
Prism	Graphpad	https://www.graphpad.com/scientific-software/prism/
Li-Cor Odyssey 3.0	Li-Cor	https://www.licor.com/bio/
Image Studio software	Li-Cor	https://www.licor.com/bio/image-studio/
Flowjo 10.6.2	Treestar	https://www.flowjo.com/
BioRender	BioRender	https://www.BioRender.com
Kaplan Meier plotter	Kaplan Meier plotter	https://kmplot.com/analysis/

### Experimental model and study participant details

#### Animals-mouse models

All procedures and experiments described in this study were performed according to the guidelines of the Canadian Council of Animal Care and approved by the Animal Care Committee of McGill University (Animal Use Protocol #MCGL5518). All animals were maintained in the Goodman Cancer Institute’s SPF facility. The MTB, MIC, germline *Chi3l1*^*-/-*^, Chi3l1 OE and MMTV-PyMT *NE*^*-/-*^ strains have been characterized and described previously.^21^^,^^24^^,^^25^^,^^35^^,^^63^ The MTB strain was generously provided by Dr. Lewis Chodosh.^63^ Briefly, the MTB transgene (MMTV-rtTA) utilizes the mammary gland specific promoter (MMTV) to drive expression of reverse tetracycline transactivator (rtTA) specifically in the mammary epithelial cells. The MIC transgene couples expression of the PyMT oncogene and Cre recombinase through an Internal Ribosomal Entry Site (IRES) element under the control of the TetO promoter.^21^^,^^22^ Doxycycline induction thus leads to expression of the PyMT oncogene as well as Cre recombinase in the mammary epithelium (Figure S2A). PyMT tumors are driven by activation of PI3K/AKT, Src and MAPK signaling pathways.^22^ In the Chi3l1 OE model, doxycycline induction leads to expression of Chi3l1 specifically from mammary epithelial cells. The MTB MIC *Chi3l1*^*-/-*^ as well as MTB MIC Chi3l1 OE crosses were described previously.^24^^,^^25^ Chi3l1 ablation or OE was confirmed through RNA FISH. The C3Tag GEMM was purchased from Jackson lab (Strain # 013591) and has been characterized previously.^26^ MTB MIC mice were crossed with CK14-TdTomato mice to generate organoid culture system where the CK14 myoepithelial layer fluoresces in red. The CK14-TdTomato was described previously^40^ and it was generously provided by Dr. Geoffrey M. Wahl. Formalin fixed paraffin-embedded (FFPE) tissue from MMTV-PyMT *NE*^*-/-*^ breast tumors were provided by Dr. Khandan Keyomarsi.^35^

All GEMM experimental animals were maintained on a uniform FVB/N background. BALB/c mice used for the 4T1 experiments were obtained from Jackson Laboratory (Cat. #000651). All experiments utilized adult virgin female mice. Adult MIC-based experimental animals were induced with doxycycline (2 mg/mL, Wisent, cat# 450–185) in their drinking water at 8 weeks of age. Mice on doxycycline were monitored for tumor formation through weekly physical palpations and caliper measurements. Mice were sacrificed at pre-determined timepoints (2 or 6-week post induction) or when they reached the ethical humane endpoint defined by the animal care guidelines as a single tumor of 2.5 cm^3^ or multiple tumors per mouse totaling 6.0 cm^3^. Each animal experiment was performed using an independent cohort of mice with its own corresponding control group. Animals and tissue samples were not reused across experiments, and all comparisons were made only between experimental groups and their matched litter-mate controls within the same cohort. C3Tag mice were monitored for tumor formation and sacrificed at tumor endpoint or at a predetermined time point (16 weeks of age), when their mammary glands develop DCIS lesions.

Breast cancer is the most common type of cancer affecting women worldwide.^64^ Less than 1% of cases occur among men.^65^ Because women are at a greater risk of developing breast cancer, we use female GEMMs in our research to accurately reflect the epidemiology of the disease. Thus, all experiments described in this study used female mice. Accordingly, the findings may not be generalizable to male breast cancer.

#### Human participants

DCIS and IDC patient samples were collected at the time of biopsy or surgery at the McGill University Health Center (MUHC) after informed written consent, in accordance with institutional ethics approval (MUHC REB protocols SDR-99–780 and SDR-00–966). All DCIS samples were from female patients who were therapy-naïve at the time of surgery (mean age of 82). DCIS diagnoses were confirmed by a pathologist, and only cases classified as pure DCIS with negative lymph node status at diagnosis were included in the study. All DCIS samples (*n=9*) were clinically documented to be ER + PR + grade II or III. FFPE tissue sections were used for immunofluorescent analyses. All DCIS samples were included and they were not allocated into different experimental groups.

A retrospective clinical cohort consisting of metastatic (*n=12*) and non-metastatic (*n=21*) breast cancer samples was used to assess relative levels of NE. The cohort consisted predominantly of HER2-positive grade II–III invasive ductal carcinomas. Clinicopathological characteristics, including age, hormone receptor status, HER2 status, histological grade, and tumor size, were similar between metastatic and non-metastatic groups (Table S1). Samples were split into two groups: metastatic vs. non-metastatic based on clinical outcome data. For both the DCIS and metastasis cohorts: race, ethnicity, and ancestry were not systematically collected and therefore could not be evaluated. Because male breast cancer cases are rare,^65^ all human tissue samples were derived from female patients. Therefore, the potential influence of sex on the observed findings could not be evaluated, and the findings may not be generalizable to male breast cancer.

Human normal (*n=9*), DCIS (*n=75*) and IDC (*n= 207*) breast tissue microarrays (BR806, BR251, BR1191, BR1503, BR8011, BR20811, BR8013 and BR301) were purchased from TissueArray.Com (formerly US Biomax Inc). The TMA included samples from female individuals with normal breast tissue, DCIS, or IDC. Samples were allocated to groups according to the diagnosis provided by the supplier. Patient information and diagnosis are available at https://www.tissuearray.com/

#### Primary cell culture and cell lines

The PyMT+ cell line was established from MMTV-PyMT primary mammary tumors as described previously.^61^ Cells were maintained in DMEM (Wisent, 219–010) media that contained 10% Fetal Bovine Serum (FBS), 5 ng/mL Epidermal Growth Factor (EGF), 35 μg/mL bovine pituitary extract, 5 μg/mL Insulin, 1 μg/mL Hydrocortisone, 5% penicillin/streptomycin and 5% amphotericin. The cells were incubated in a 5% CO2, 37°C incubator at all times. PyMT-derived cell lines were generated in-house from PyMT mammary tumors, and their identity was confirmed based on the expected PyMT genotype and phenotype. All cells were confirmed to be mycoplasma-free using the MycoAlert Kit (Lonza, LT07-118).

In order to extract murine neutrophils, 50K cells of the liver metastatic 4T1 derivative (4T1-2776) were injected into the mammary fat pad of 7-week-old female Balb/c mice (Jackson lab, Cat#000651). The cell line was previously characterized^66^ and generously provided by Dr. Peter Siegel. The cells exhibited the expected 4T1 cellular and tumor phenotype and were confirmed to be mycoplasma-free using the MycoAlert Kit (Lonza, LT07-118). 3 weeks post-injection, the blood was collected by cardiac puncture and extraction of primary mouse low density neutrophils (LDNs) was performed as previously described.^62^ LDNs were cultured at 37°C, 5% CO2 in RPMI medium (Wisent, 350–000-CL) supplemented with 3% FBS, 5% penicillin/streptomycin and 5% amphotericin. In order to generate neutrophil conditioned media, freshly extracted LDNs were cultured in the presence of 10 uM Alvelestat (HY-15651, Medchem express) or vehicle control (10% DMSO, 75% PBS, 15% Tween-80) at 37°C, 5% CO2 for 10 h. Conditioned media was then collected, centrifuged to remove cell debris and stored at -80°C until used for laminin and collagen IV degradation assays.

The human breast cancer MCF-7 cell line (ATCC, cat# HTB-22) was cultured in DMEM supplemented with 10% FBS and was used for the MIND model experiment. MCF7 cells were obtained from ATCC and authenticated by the supplier. All cells were confirmed to be mycoplasma free using the MycoAlert Kit (Lonza, LT07-118).

### Method details

#### Pharmacological treatments

Neutrophil depletion in WT MTB MIC and Chi3l1 OE MTB MIC mice through anti-Ly6G treatment was described previously.^24^^,^^25^ FFPE mammary gland tissue from these experiments was used in this study. Neutrophil depletion was previously confirmed through fluorescent activated cell sorting as well as immunofluorescent staining.^24^^,^^25^ Similarly, Pad4i (GSK484, HY-100514, Medchem express) and DNAse 1 (4536282001, Roche) treatments in WT MTB MIC mice has been described and validated previously.^24^

In order to deplete neutrophils in WT C3Tag mice, mice were randomized to receive 400 μg of purified anti-Ly-6G rat antibody (1A8, BioXcell #BE0075-1) or IgG2a rat isotype control (2A3, Bio X cell #BE0089) twice per week via IP injections. Treatments started when mice reached 12 weeks of age and continued for 4 weeks (16 weeks of age). Neutrophil depletion was confirmed through FACS sorting on the blood for circulating neutrophils as well as through immunofluorescent staining on the mammary glands.

In order to inhibit neutrophil degranulation, cohorts of MTB MIC Chi3l1 OE mice were randomized to receive 30mg/kg Nexinhib20 (HY-125792, Medchem express) via intraperitoneal (IP) injections. Nexinhib20 was dissolved in PBS, DMSO Polyethylene glycol and tween-80. Treatment took place daily starting 5 days before doxycycline induction and continued for 2 weeks post induction. Control mice were given mock (Vehicle) injections at the same time and for the same duration. Immunofluorescent staining and ELISA analyses were undertaken to validate successful inhibition of degranulation without impacting overall neutrophil recruitment.

In order to inhibit NE, cohorts of WT MTB MIC and MTB MIC Chi3l1 OE mice were randomized to receive 50mg/kg of Alvelestat (HY-15651, Medchem express) via IP injections. Alvelestat was dissolved in PBS, DMSO, Polyethylene glycol and tween-80. Treatment took place daily starting 5 days before doxycycline induction and continued for 2 or 6 weeks post induction. Control mice were given mock (Vehicle) injections at the same time and for the same duration. In the second set of experiments, cohorts of MTB MIC Chi3l1 OE were induced for 2 weeks before starting daily IP injections with 50mg/kg of Alvelestat until 6 weeks of induction. Immunofluorescent staining and immunoblots for direct NE-degradation targets was performed to confirm successful inhibition of NE. Similarly, to inhibit NE in C3Tag mice, Cohorts of WT C3Tag mice were randomized to receive 50mg/kg of Alvelestat (HY-15651, Medchem express) or vehicle control via IP injections. Treatments started when mice reached 12 weeks of age and continued daily for 4 weeks (16 weeks of age). Control mice were given mock (Vehicle) injections at the same time and for the same duration.

In order to study the role of NE in established mammary tumors, cohorts of WT MTB MIC mice were induced with doxycycline and monitored for tumor formation (average tumor onset at 35 days post-induction). Once tumors were palpable (measured at 250mm^3^), the mice were randomized to receive 50mg/kg of Alvelestat or vehicle control. Treatment took place daily until reaching humane endpoint (approximately 60 days post tumor onset). The mice were then sacrificed and the lungs were analyzed for metastatic burden.

#### Mammary intraductal engraftment

The human breast cancer MCF-7 cell line (ATCC, cat# HTB-22) was cultured in DMEM supplemented with 10% FBS. 5 × 104 cells were suspended in 10 μL of PBS with 2 μL of trypan blue and injected intraductally into 4th mammary gland of 8- to 12-week-old NOD-scid IL2Rgammanull (NSG) female mice (Jackson lab, Cat# 005557) Mammary glands were collected at 4, 8, or 12 weeks post-injection and fixed overnight in Carnoy’s Fixative. Tissues were embedded in paraffin and sectioned at 4 μm. Sections were first stained with H&E for initial tumor staging, then confirmed by immunofluorescent staining. Tissues with cancer cells confined to the ductal tree were classified as DCIS, as confirmed by the presence of myoepithelial cells on immunofluorescence (CK5+). Tissues with stromal infiltration of cancer cells were classified as invasive. All lesions were of high nuclear grade (prominent pleomorphism and nucleoli; large nuclei (>2.5 times the size of a normal ductal epithelial cell)).

#### Human bioinformatic analysis

Kaplan–Meier curves depicting overall survival based on *ELANE* mRNA expression in breast cancer patients were generated using the web-based tool Kaplan Meier plotter (https://kmplot.com/analysis/). The analysis was for 2976 breast cancer patient sample, stratified according to median *ELANE* expression.

For *ELANE* expression in DCIS datasets, the GSE281303 RNAseq publicly available dataset^43^ was interrogated for *ELANE* expression in DCIS progressors vs. non-progressors and analyzed using Wilcoxon rank-sum test.

#### RNA FISH

RNAscope *in situ* hybridization was performed on paraffin-embedded mammary gland sections using RNAscope 2.5 HD Assay-RED kit (ACD, Cat#322360) according to the manufacturer’s protocol. The following probes were used: mouse *Chi3l1* (Cat# 449621). This protocol was followed with fluorescent IHC for pan-ck as described below.

#### Protein extraction and immunoblots

Flash-frozen mammary gland or breast tumor pieces were crushed in liquid nitrogen using a mortar and a pestle. RIPA buffer (10 mM Tris at PH 8, 1 mM EDTA, 0.5 mM EGTA, 1% Triton X-100, 0.1% sodium deoxycholate, 0.1% SDS, 140 mM NaCl) with protease inhibitor cocktail (1 μg/mL aprotinin, 1 μg/mL leupeptin, 1 mM sodium orthovanadate and 1 μg/mL phenylmethylsulfonyl fluoride) was added to lyse the cells. The samples were left to rotate at 4°C for a minimum of 60 min. Lysates were centrifuged at 13000 RPM for 10 min at 4°C and the supernatant was collected.

Immunoblot was performed as previously described^67^^,^^68^ using the following antibodies: Chi3l1 (1/1000, Invitrogen PA5-81356), CitH3 (1/1000, Abcam Ab5103), TSP1 (1/1000, Cell Signaling 37879S), β-actin (1/5000, Sigma Aldrich A5441), Vinculin (1/2000, Millipore MAB3574) and α-tubulin (1/1000, Cell signaling 3873). The membranes were imaged using the Odyssey CL-C imaging system. Image Studio software (LICOR Biosciences) was used to analyze the images. Each target protein was normalized to its appropriate loading control on the same membrane.

#### Multiplex fluorescent immunohistochemistry

FFPE tissue sections were stained as previously described.^24^ Briefly, the slides were deparaffinized in 3 successive 3-min incubations with xylene followed by dehydration in decreasing concentrations of ethanol. Target retrieval was performed in Citrate buffer (Ph 6, Vector Laboratories #H-3300–250) or EDTA Buffer (Ph 9, Vector Laboratories #H-3301–250) using a pressure cooker for 10 min. In order to block endogenous H2O2, the samples were incubated in 3% hydrogen peroxide for 10 min before blocking with casein-based buffer (Vector Laboratories, #SP-5020) for 5 min. Primary antibody was added for 30 min at room temperature. After 3 washes with tris-buffered saline with tween 20 (TBST), ImmPRESS HRP Polymer secondary antibodies (Vector Laboratories; anti-mouse VECTMP745250 or anti-rabbit VECTMP740150) was added for 30 min at room temperature. 3 additional washes with TBST were performed before adding the fluorophore. Opal working solution containing tyramide signal amplification substrates (OPAL 480: OP-001000, OPAL 520: OP-001001, OPAL 570: OP-001003, OPAL 620: OP-001004, OPAL 690: OP-001006, or OPAL 780; OP-001008 Akoya biosciences) was added and incubated for 10 min at room temperature. Slides were subjected to another round of target retrieval and the protocol was repeated up to 4 colors/antibodies per section. After the last round of staining, DAPI was added and the samples mounted for imaging.

For all staining experiments, the entire mammary gland or breast tumor tissue sections were scanned using the AxioScan Z1 digital slide scanner (Carl Zeiss). For quantifications, we used the HALO (Indica Lab) system sing one of the following algorithms: ‘Fluorescent intensity’ or ‘Area Quantification FL v3.0.1’. The entire tissue section was analyzed for all staining experiments. No ROIs were manually selected; instead, the full tissue area was included in every analysis to avoid sampling bias. All samples were analyzed using identical image acquisition and analysis settings within the same analysis batch. Each sample represents an independent biological replicate. Quantification of immune cell populations is presented as the percentage of total cells within the analyzed region, ECM measurements are presented as the percentage of total analyzed area, and myoepithelial cell measurements are presented as cell density (number of positive cells normalized to the analyzed tissue area). The specific quantification method used for each analysis is indicated in the corresponding figure legend. Representative images throughout the manuscript are cropped, higher-magnification regions selected from the whole-slide scans to allow visualization of the relevant cellular and tissue features at publication resolution. Image selection was performed solely for visualization purposes and had no influence on the quantitative analysis, which was performed on the complete scanned section.

For ECM analyses, positive staining was quantified using the ‘Area Quantification FL v3.0.1’ module in HALO (Indica lab). A supervised, trainable algorithm was optimized to specifically detect fluorophore-associated signals corresponding to individual ECM markers and to calculate area fraction (positive staining as a percentage of total tissue area). Analysis of lung metastatic area was performed using HALO’s ‘Classifier function’ which is teachable algorithm that can distinguish metastatic lesions from surrounding normal lung tissue. The percent of total lung area that is occupied by lung metastatic lesions was quantified. Distance analysis was performed using HALO’s ‘nearest neighbor’ algorithm, which measures the distance from each cell in a target population (e.g., NE + cells) to the nearest neighbor cell of a second specified population (e.g., CK14+ *p*-FAK+ cells) and then averages these distances across the entire dataset.

The following primary antibodies were used: mouse and human CK14 (1/10, Ventana 760–4805), CK5 (1/500, Biolegend 905504), Calponin 1, (1/100, Cell Signaling 17819), P63 (1/300, Santa Cruz sc25268), α-SMA (1/400, DAKO M0851), Ly6G (1/100, Cell signaling 87048), MPO (1/500, Abcam ab208670), CitH3 (1/100, Cell signaling 26111), mouse NE (1/200, Cell signaling 90120), Human NE (1/200, Cell signaling 28162), Laminin (1/200, Abcam ab11575), Collagen IV (1/100, Abcam ab6586), Elastin (1/200, BIOS USA bs-1756R), Cleaved caspase 3 (1/200, Cell signaling 9661), *p*-FAK (1/200, Invitrogen, 44-624G), Ck8 (1/100, Biolegend 904804), CD3 (1/200, Abcam ab16669), CD8 (1/200, Cell signaling 98941), CD4 (1/50, Cell signaling 25229), PD1 (1/200, Cell signaling 84651), Granzyme B (1/200, Cell signaling 44153), Ki67 (1/200, Cell signaling 12202), PCNA (1/400, Cell signaling 2586) and Pan-ck (1/10, Ventana 760–2595). All antibodies were diluted in 2% BSA TBST.

#### FACS analysis

Mammary glands from MTB MIC mice treated with Alvelestat or vehicle control for 2 weeks were excised and the lymph nodes were removed. The mammary glands were then processed as described previously.^24^^,^^25^ BD Cytofix/Cytoperm fixation/permeabilization kit (BD Bioscience, #554714) was used according to manufacturer’s protocol. The following antibodies were used: Viability was assessed using fixable viability dye eFluorYM 506 (ThermoFisher/eBioscience 65–0866–14). Viable cells were then analyzed using the following antibodies: CD45 (BD Bioscience, 564225), CD11b (Biolegend, 101201), F4/80 (Biolegend, 123114), Ly6G (Biolegend, 127606), Epcam (Biolegend, 118233), CD49f (Biolegend, 313622), CK5 (Santa Cruz, sc-32721 PE), and CK14 (Santa Cruz, sc-53253AF488). Flow cytometry was done in the BD LSR Fortessa 4 lasers (405/488/561/633 nm) flow cytometer and the results were analyzed using Flowjo 10.6.2 (Treestar).

#### Enzyme-linked immunosorbent assay

Cardiac puncture was performed to collect blood from Nex20-treated mice at 2-week post induction. The blood was incubated at room temperature for 1 h to allow it to clot before being centrifuged for 20 min at 1000xg at 4°C. The supernatant (serum) was collected and used for enzyme-linked immunosorbent assay ELISA at a 1 in 50 dilution. ELISA for NE (R&D, Cat# DY4517-05) or MPO (R&D, Cat# DY3667) was performed following the manufacturer’s protocol. Each experiment was performed in duplicates and repeated twice.

#### *In vitro* laminin degradation assay

To test for laminin degradation on synthetic ECM, Cultrex Growth Factor Basement Membrane Extract (R&D, Cat# 3433–010–01). Was used to coat a 96-well glass bottom microscopy plate (Thermofisher Scientific, Nunc, Cat#165305). The plate was incubated at 37°C in 5% CO_2_ for 30 min to solidify. PyMT+ cells were plated at 1 × 10^4^ cells per well with neutrophil conditioned media treated with Alvelestat or Vehicle control. The plates were incubated at 37°C in 5% CO_2_ for 12 h. After washing with PBS, the cells were fixed using 2% paraformaldehyde. Cells were then permeabilized with PBS containing 0.5% Triton X-100 for 10 min at room temperature, followed by blocking using PBS with 0.2% Triton X-100 and 0.05% Tween 20 (IF buffer) and 2% BSA for 30 min at room temperature. Primary anti-laminin antibody (1/200, Abcam Ab11575) was added and incubated overnight at 4°C. The cells were washed three times before adding Goat anti-rabbit secondary antibody Alexa Fluor 555 (Invitrogen A21428) for 40 min at room temperature. After three additional washes with IF buffer, DAPI was added for 5 min at room temperature. The cells were then incubated in PBS and imaged using Zeiss AxioObserver fully motorized inverted confocal microscope LSM 710 with a 10× objective. Each experiment was performed in triplicates and repeated twice.

This assay was also repeated in neutrophil conditioned medium or media supplemented with PBS control, 1 μg/mL of recombinant mouse neutrophil elastase (R&D system, 4517-SE), 1 μg/mL of recombinant mouse cathepsin G (Biomatik, RPU51859), 2 μg/mL of recombinant mouse proteinase 3 (Biomatik, RPU43519) or 200ng/mL of recombinant murine Chi3l1(R&D systems, 2649-CH). All recombinant proteins were dissolved in PBS. The assay was ran in triplicates and repeated twice.

#### *In vitro* collagen IV degradation assay

To test for laminin degradation on synthetic ECM, DQ collagen, type IV from human placenta, fluorescein conjugate (Invitrogen, D12052) was used to coat a 96-well glass bottom microscopy plate (Thermofisher Scientific, Nunc, Cat#165305). DQ Collagen was prepared at a 1mg/mL concentration in distilled water. The coating was dissolved by heating it up to 50°C with agitation. The stock was then diluted to a working concentration of 25 μg/mL in 0.1% unconjugated gelatin. PyMT+ cancer cells were culture in neutrophil conditioned media treated with Alvelestat or vehicle control and were incubated at 37°C in 5% CO_2_ for 12 h. After washing with PBS, the cells were fixed using 2% paraformaldehyde. Cells were then permeabilized with PBS containing 0.5% Triton X-100 for 10 min at room temperature, followed by blocking using PBS with 0.2% Triton X-100 and 0.05% Tween 20 (IF buffer) and 2% BSA for 30 min at room temperature. At that point, degraded collagen would fluoresce green under the microscope. For Actin staining, Alexa Fluor plus 555 phalloidin was used (1/500 Invitrogen A30106) and incubated for 45 min at room temperature. After three additional washes with IF buffer, DAPI was added for 5 min at room temperature. The cells were then incubated in PBS and imaged using Zeiss AxioObserver fully motorized inverted confocal microscope LSM 710 with a 10× objective. The experiment was performed in triplicates and repeated twice.

The assay was also repeated in the presence of recombinant NE, recombinant cathepsin G, recombinant Proteinase 3 or recombinant Chi3l1 as described for laminin degradation assay.

#### Trans-well invasion assay

PyMT cancer cell invasion assay through synthetic ECM was performed as described previously.^25^ PyMT cancer cells were placed on top of the inserts in the appropriate conditioned media, and the assay was performed as previously described.^25^ Following 12 h of incubation at 37°C, 5% CO2, the filters were removed, and the non-migrating cells were removed from the top of the membrane. The cells that invaded through the membrane were fixed in 10% neutral buffered formalin for 30 min and filters were counterstained with crystal violet. Image acquisition of stained cells was performed with an EVOS M7000 Invitrogen microscope (objective 10×). Cells were manually counted on HALO. The assay was repeated twice.

#### Organoid culture and NE treatment

Mammary glands (3rd, 4th and 5th) were isolated from MIC CK14-Tdtomato^40^ adult mice, minced with scissors and digested in a pre-warmed digestion media (DMEM/F12 supplemented with 5 μg/mL insulin, 600 U/mL Nystatin, 100 U/mL Penicillin/Streptomycin, 2 mg/mL Collagenase A, 0.00625% Trypsin EDTA) for 30 min with rotation. Following a 5-min 1000rpm spin at room temperature, cells were rinsed in DMEM/F12 and spun for 10 min, at 1000rpm spin at room temperature. Epithelial clumps were purified through a series of five quick spins (1500rpm, 15 s) and washes (PBS 5%FBS). Following this, organoids were resuspended in 100% Geltrex (gibco#A1413202) and plated in domes above a 100% Geltrex pad in ibidi plates (ibidi#80826) and grown with Mammosphere media (EpiCult Mouse media (STEMCELL#05610) supplemented with 5 μg/mL insulin, 100 U/mL Penicillin/Streptomycin, 0.02 μg/mL mEGF (R&D systems#2028-EG-200), 1 μg/mL hydrocortisone, 4% KnockOut serum (gibco#10828010)) with doxycycline (3mg/mL). These were allowed to recover and reorganize into organoids over a 72-h period and were then removed from their Geltrex domes with prewarmed cell recovery solution (Corning#354253). Organoids were then moved to rolling culture (in mammosphere media) where they were treated with either vehicle (PBS) or neutrophil elastase (50mM) for 24 h. Prior to imaging, organoids were stained with Incucyte Cytotox 488 reagent (Sartorius#4633) at 1:2000 dilution to visualize dying cells and DRAQ5 (Thermofisher# 62251) to visualize nuclei. Organoids were carefully moved to a clean ibidi plate and imaged using the LSM-910 confocal laser scanning microscope, equipped with a 20×/NA = 0.40 long distance objective lens. Following imaging of live organoids, organoids were fixed in 4% PFA for 20 min at room temperature and immunostained using antibodies against cytokeratin 5 (Biolegend #905901) at 1/200 dilution. Analysis was performed using ImageJ v1.54p. Organoid perimeter was measured manually by tracing the outline of the organoid structures in the DIC channel. All tdTomato positive cells that came in contact with the organoid perimeter were counted. Statistical analysis on this data was done using JMP Student Edition 19. For each condition, *n* = 3 mice were used to generate 10–15 organoids per mouse.

### Quantification and statistical analysis

All statistical analyses were performed using Prism software (GraphPad, San Diego, CA). The appropriate statistical test was used for different experiments and is indicated in the figure legend. One-way ANOVA with Tukey’s or Dunnett’s post-hoc tests for multiple comparisons were performed as appropriate. Mann-Whitney non-parametric test, two-tailed paired or un-paired Student’s *t* test were used to compare two groups. Effect sizes were determined using Cohen’s d for two-group comparisons or Eta squared for multiple comparison, the values are indicated in the figure legends. For all figures, the data is plotted as average ±standard error of the mean (SEM). Each data point is an independent biological sample. The results of the statistical tests can be found within the figures and figure legends. *p* values less than 0.05 were considered statistically significant. Sample size (*n*) is indicated in the figure legend. (*n)* represents independent biological replicates (separate mice or human breast cancer samples). Sample sizes were determined using a combination of *a priori* power calculations (power = 0.8, α = 0.05) for key genetic mouse cross experiments and estimates derived from pilot studies and prior published work. For *in vivo* drug treatment experiments, sample sizes were based on prior literature and pilot experiments using the same model systems. No samples were excluded.

## Supplemental information

Document S1. Figures S1–S14 and Table S1

Data S1. Source raw data underlying all quantitative analyses and figures presented in this manuscript, related to all figures

Document S2. Article plus supplemental information
