# Supplementary material for: Neutrophil elastase-mediated myoepithelial loss is a therapeutic vulnerability in ductal carcinoma in situ progression
Source: Cell Rep Med. 2026 Sep 11;7(10):103050. doi: 10.1016/j.xcrm.2026.103050 (PMC13710378; doi:10.1016/j.xcrm.2026.103050)
Supplement: Supplementary file 1 [file mmc1.pdf]

Cell Reports Medicine, Volume *7*

Supplemental information

Neutrophil elastase-mediated myoepithelial loss is a therapeutic vulnerability in ductal carcinoma *in situ* progression

Tarek Taifour, Yu Gu, Adéline Massé, Xiwei Meng, Clarisse Dane Echavez, Dongmei Zuo, Virginie Sanguin-Gendreau, Anne Blouin, Adda-Lee Graham-Paquin, Miranda Addie, Emilie Solymoss, Bin Xiao, Hailey Proud, Sherif Samer Attalla, Alice Jisoo Nam, Vasilios Papavasiliou, Hellen Kuasne, Peter M. Siegel, Khandan Keyomarsi, Luke McCaffrey, Morag Park, and William J. Muller

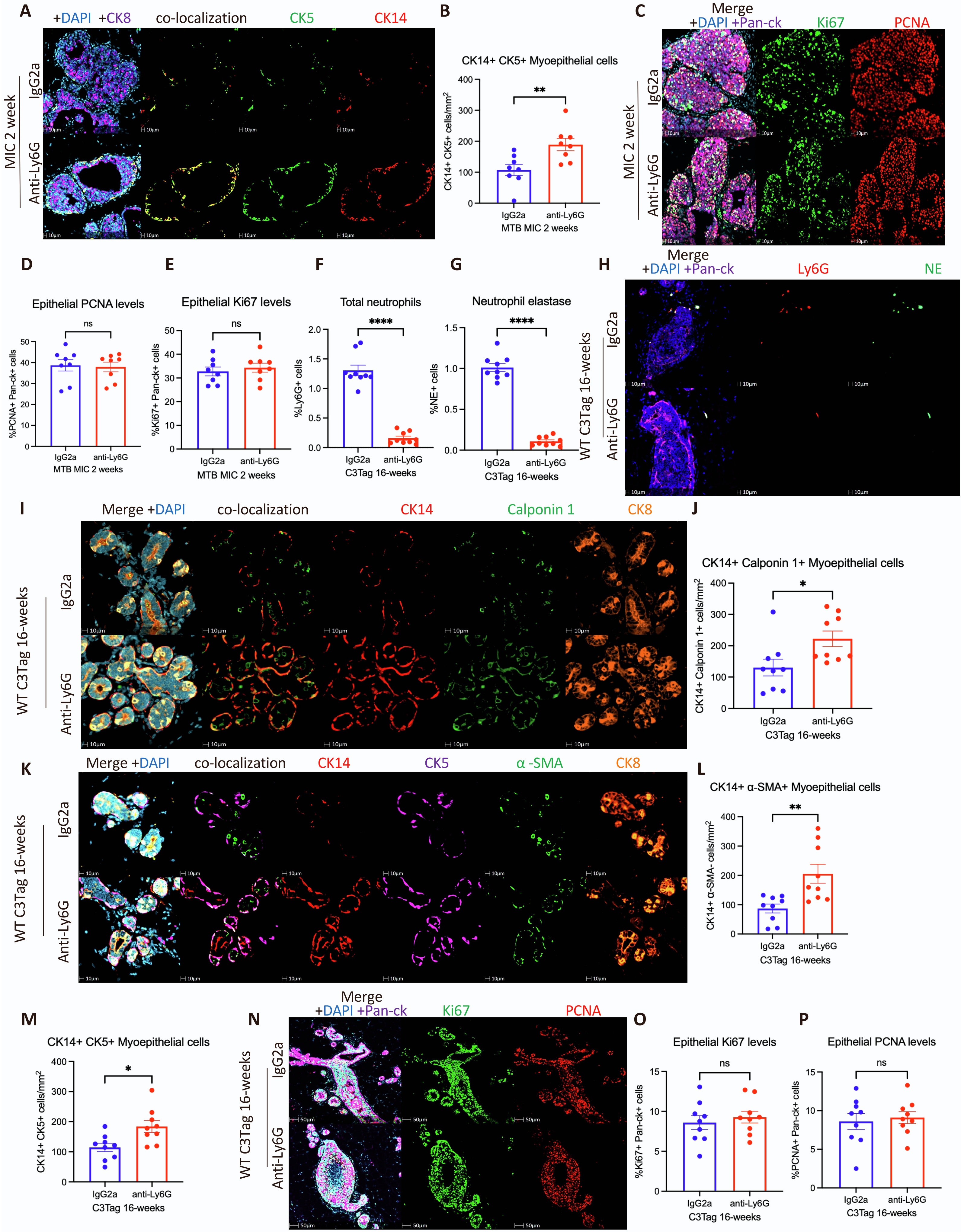

A
+DAPI +CK8
co-localization
CK5
CK14
MIC 2 week
IgG2a
Anti-Ly6G
B
CK14+ CK5+ Myoepithelial cells
CK14+ CK5+ cells/mm2
**
IgG2a
anti-Ly6G
MTB MIC 2 weeks
C
Merge
+DAPI +Pan-ck
Ki67
PCNA
MIC 2 week
IgG2a
Anti-Ly6G
D
Epithelial PCNA levels
%PCNA+ Pan-ck+ cells
ns
IgG2a
anti-Ly6G
MTB MIC 2 weeks
E
Epithelial Ki67 levels
%Ki67+ Pan-ck+ cells
ns
IgG2a
anti-Ly6G
MTB MIC 2 weeks
F
Total neutrophils
%Ly6G+ cells
****
IgG2a
anti-Ly6G
C3Tag 16-weeks
G
Neutrophil elastase
%NE+ cells
****
IgG2a
anti-Ly6G
C3Tag 16-weeks
H
Merge
+DAPI +Pan-ck
Ly6G
NE
WT C3Tag 16-weeks
IgG2a
Anti-Ly6G
I
Merge +DAPI
co-localization
CK14
Calponin 1
CK8
WT C3Tag 16-weeks
IgG2a
Anti-Ly6G
J
CK14+ Calponin 1+ Myoepithelial cells
CK14+ Calponin 1+ cells/mm2
*
IgG2a
anti-Ly6G
C3Tag 16-weeks
K
Merge +DAPI
co-localization
CK14
CK5
α -SMA
CK8
WT C3Tag 16-weeks
IgG2a
Anti-Ly6G
L
CK14+ α-SMA+ Myoepithelial cells
CK14+ α-SMA- cells/mm2
**
IgG2a
anti-Ly6G
C3Tag 16-weeks
M
CK14+ CK5+ Myoepithelial cells
CK14+ CK5+ cells/mm2
*
IgG2a
anti-Ly6G
C3Tag 16-weeks
N
Merge
+DAPI +Pan-ck
Ki67
PCNA
WT C3Tag 16-weeks
IgG2a
Anti-Ly6G
O
Epithelial Ki67 levels
%Ki67+ Pan-ck+ cells
ns
IgG2a
anti-Ly6G
C3Tag 16-weeks
P
Epithelial PCNA levels
%PCNA+ Pan-ck+ cells
ns
IgG2a
anti-Ly6G
C3Tag 16-weeks

Supplementary Figure 1: Neutrophils depletion increases CK5 myoepithelial marker without Impacting cancer proliferation. Related to Figure 1.

(A) Fluorescent IHC staining of mammary tissue from WT MIC mice treated with anti-Ly6G or IgG2a isotype control for 2 weeks, using antibodies against CK14, CK5 and CK8, counterstained with DAPI. **(B)** Quantification of total CK14+ CK5+ cells normalized to total analyzed area in IgG2a (*n* = 8) and anti-Ly6G (*n* = 8) treated WT MIC mammary glands at 2 weeks post induction. Derived from the staining shown in A. **(C)** Fluorescent IHC staining of mammary tissue from WT MIC mice treated with anti-Ly6G or IgG2a isotype control for 2 weeks, using antibodies against Ki67, PCNA and Pan-ck, counterstained with DAPI. **(D,E)** Quantification of total Pan-ck+ Ki67+ and Pan-ck+ PCNA+ cells as a percentage of total cells in IgG2a (*n* = 8) and anti-Ly6G (*n* = 8) treated WT MIC mammary glands at 2 weeks post induction. Derived from the staining shown in C. **(F,G)** Quantification of total Ly6G+ and NE+ cells as a percentage of total cells in IgG2a (*n* = 9), and anti-Ly6G (*n* = 9) treated WT C3Tag mammary glands at 16-weeks. Derived from the staining shown in H. **(H)** Fluorescent IHC staining of IgG2a and anti-Ly6G-treated WT C3Tag at 16-weeks using antibodies against Ly6G, NE, and Pan-ck, counterstained with DAPI. **(I)** Fluorescent IHC staining of IgG2a and anti-Ly6G-treated WT C3Tag at 16-weeks using antibodies against CK14, Calponin 1, and CK8, counterstained with DAPI. **(J)** Quantification of total CK14+ calponin 1+ cells normalized to total analyzed area in IgG2a (*n* = 9), and anti-Ly6G (*n* = 9) treated WT C3Tag mammary glands at 16-weeks. Derived from the staining shown in I. **(K)** Fluorescent IHC staining of IgG2a and anti-Ly6G-treated WT C3Tag at 16-weeks using antibodies against CK14, α-SMA, CK5 and CK8, counterstained with DAPI. **(L,M)** Quantification of total CK14+ α-SMA+ and CK14+ CK5+ cells normalized to total analyzed area in IgG2a (*n* = 9), and anti-Ly6G (*n* = 9) treated WT C3Tag mammary glands at 16-weeks. Derived from the staining shown in K. **(N)** Fluorescent IHC staining of IgG2a and anti-Ly6G-treated WT C3Tag at 16-weeks using antibodies against Ki67, PCNA, and Pan-ck counterstained with DAPI. **(O,P)** Quantification of total Pan-ck+ Ki67+ and Pan-ck+ PCNA+ cells as a percentage of total cells in IgG2a (*n* = 9), and anti-Ly6G (*n* = 9) treated WT C3Tag mammary glands at 16-weeks. Derived from the staining shown in N. Ns: not significant, *p<0.05, **p<0.01, and ****p<0.0001 calculated by Mann-Whitney non-parametric test. Data are presented as mean ± SEM. Each data point is an independent biological sample, *N* represents the number of independent biological replicates. Analysis was performed by the HALO (Indica lab) software. Quantification was performed on whole-slide scans of the entire mammary gland section.

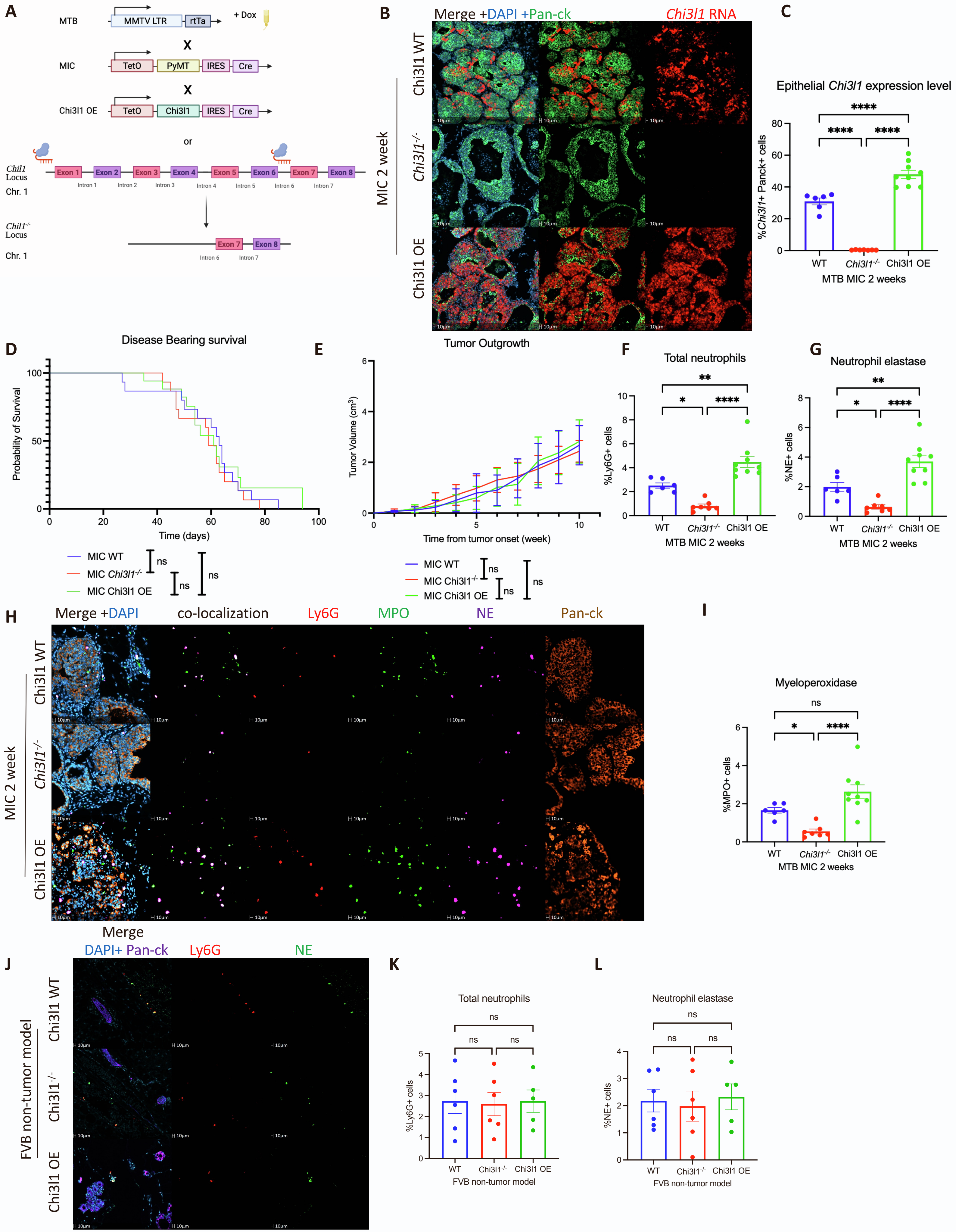

A
MTB
MMTV LTR
rtTa
+ Dox
X
MIC
TetO
PyMT
IRES
Cre
X
Chi3l1 OE
TetO
Chi3l1
IRES
Cre
or
Chil1 Locus
Chr. 1
Exon 1
Exon 2
Exon 3
Exon 4
Exon 5
Exon 6
Exon 7
Exon 8
Intron 1
Intron 2
Intron 3
Intron 4
Intron 5
Intron 6
Intron 7
Chil1-/- Locus
Chr. 1
Exon 7
Exon 8
Intron 6
Intron 7
B
Merge +DAPI +Pan-ck
Chi3l1 RNA
Chi3l1 WT
Chi3l1-/-
Chi3l1 OE
MIC 2 week
C
Epithelial Chi3l1 expression level
%Chi3l1+ Panck+ cells
WT
Chi3l1-/-
Chi3l1 OE
MTB MIC 2 weeks
D
Disease Bearing survival
Probability of Survival
Time (days)
MIC WT
MIC Chi3l1-/-
MIC Chi3l1 OE
ns
E
Tumor Outgrowth
Tumor Volume (cm3)
Time from tumor onset (week)
MIC WT
MIC Chi3l1-/-
MIC Chi3l1 OE
ns
F
Total neutrophils
%Ly6G+ cells
WT
Chi3l1-/-
Chi3l1 OE
MTB MIC 2 weeks
G
Neutrophil elastase
%NE+ cells
WT
Chi3l1-/-
Chi3l1 OE
MTB MIC 2 weeks
H
Merge +DAPI
co-localization
Ly6G
MPO
NE
Pan-ck
Chi3l1 WT
Chi3l1-/-
Chi3l1 OE
MIC 2 week
I
Myeloperoxidase
%MPO+ cells
WT
Chi3l1-/-
Chi3l1 OE
MTB MIC 2 weeks
J
Merge
DAPI+ Pan-ck
Ly6G
NE
Chi3l1 WT
Chi3l1-/-
Chi3l1 OE
FVB non-tumor model
K
Total neutrophils
%Ly6G+ cells
WT
Chi3l1-/-
Chi3l1 OE
FVB non-tumor model
L
Neutrophil elastase
%NE+ cells
WT
Chi3l1-/-
Chi3l1 OE
FVB non-tumor model

Supplementary Figure 2: Characterization of the *Chi3l1*$^{-/-}$ and Chi3l1 OE MIC GEMMs. Related to Figure 1.
(A) Schematic representation of the MTB MIC *Chi3l1*$^{-/-}$ and Chi3l1 OE GEMMs used in this study, created with BioRender.com. **(B)** RNA FISH against *Chi3l1* combined with fluorescent IHC staining for Pan-ck, and DAPI on mammary tissue from WT, *Chi3l1*$^{-/-}$ and Chi3l1 OE MIC mammary glands at 2 weeks post induction. **(C)** Quantification of *Chi3l1*+Pan-ck+ cells as a percentage of total cells in WT (*n* = 6), *Chi3l1*$^{-/-}$ (*n* = 7), and Chi3l1 OE (*n* = 9) MIC mammary glands at 2 weeks post induction. Derived from the staining shown in B. **(D)** Disease-bearing survival of WT (*n* = 14), *Chi3l1*$^{-/-}$ (*n* = 15), and Chi3l1 OE (*n* = 17) MIC mammary tumor-bearing mice measured from day of measurable tumor (onset) to humane endpoint. Statistical analysis by Mantel-Cox test. **(E)** Primary tumors growth in WT (*n* = 14), *Chi3l1*$^{-/-}$ (*n* = 15), and Chi3l1 OE (*n* = 17) MIC mammary tumor-bearing mice. Tumors were measured and tracked weekly by a caliper. **(F,G)** Quantification of total Ly6G+ and NE+ cells as a percentage of total cells in WT (*n* = 6), *Chi3l1*$^{-/-}$ (*n* = 7), and Chi3l1 OE (*n* = 9) MIC mammary glands at 2 weeks post induction. Derived from the staining shown in H. **(H)** Fluorescent IHC staining of WT, *Chi3l1*$^{-/-}$, and Chi3l1 OE MIC mammary glands at 2 weeks post induction using antibodies against Ly6G, MPO, NE, and Pan-ck, counterstained with DAPI. **(I)** Quantification of total MPO+ cells as a percentage of total cells in WT (*n* = 6), *Chi3l1*$^{-/-}$ (*n* = 7), and Chi3l1 OE (*n* = 9) MIC mammary glands at 2 weeks post induction. Derived from the staining shown in H. **(J)** Fluorescent IHC staining of WT, *Chi3l1*$^{-/-}$, and Chi3l1 OE (non-MIC, non-tumor) mammary glands using antibodies against Ly6G, NE, and Pan-ck, counterstained with DAPI. **(K,L)** Quantification of total Ly6G+ and NE+ cells as a percentage of total cells in WT (*n* = 6), *Chi3l1*$^{-/-}$ (*n* = 6), and Chi3l1 OE (*n* = 5) (non-MIC, non-tumor) mammary glands. Derived from the staining shown in J. Ns: not significant, *$p<0.05$, **$p<0.01$, and ****$p<0.0001$ calculated by One-Way Anova with Tukey's post-hoc test. Data are presented as mean ± SEM. Each data point is an independent biological sample, *N* represents the number of independent biological replicates. Analysis was performed by the HALO (Indica lab) software. Quantification was performed on whole-slide scans of the entire mammary gland section.

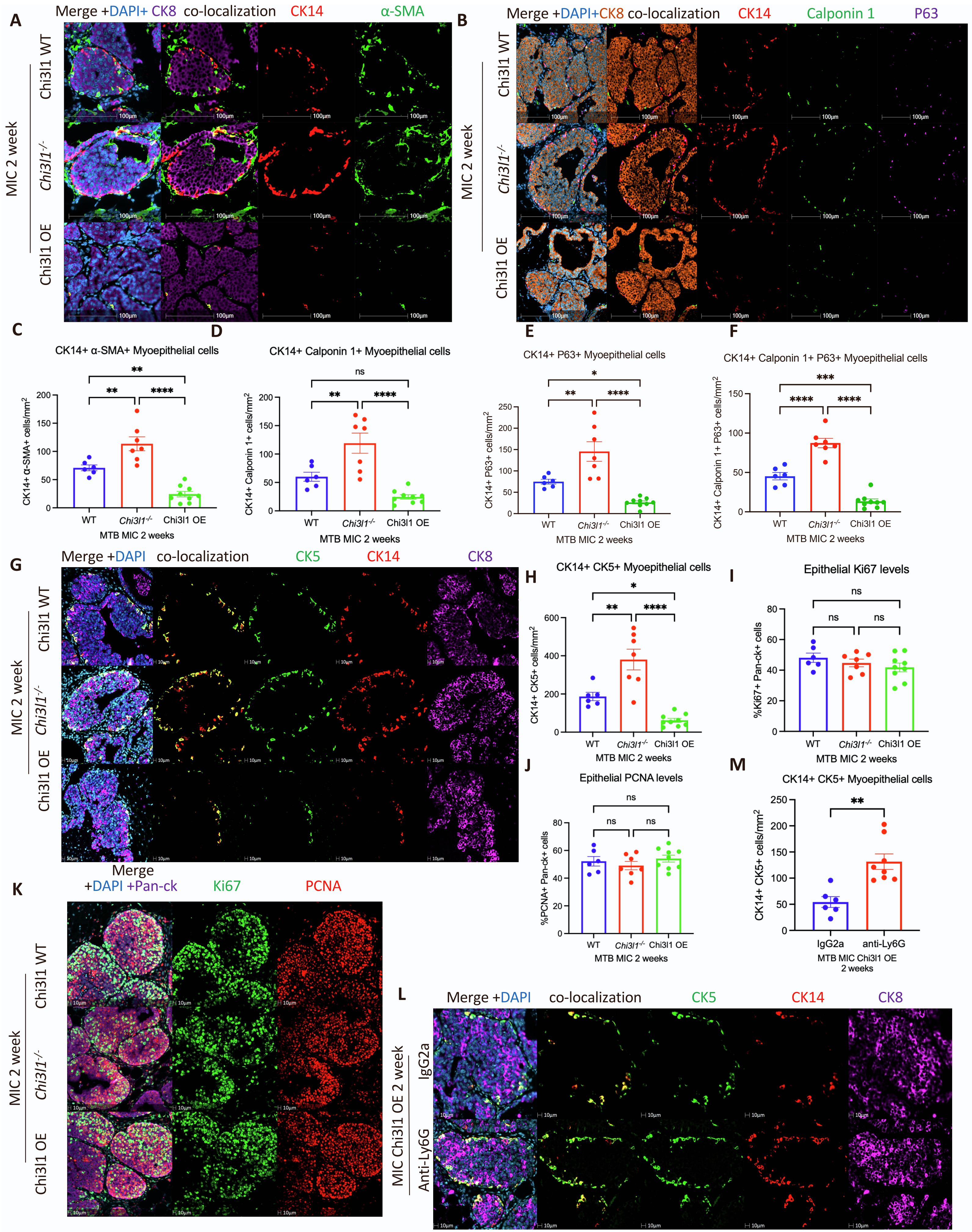

A
Merge +DAPI+ CK8
co-localization
CK14
α-SMA
MIC 2 week
Chi3l1 WT
Chi3l1-/-
Chi3l1 OE
100μm
B
Merge +DAPI+CK8
co-localization
CK14
Calponin 1
P63
MIC 2 week
Chi3l1 WT
Chi3l1-/-
Chi3l1 OE
100μm
C
CK14+ α-SMA+ Myoepithelial cells
CK14+ α-SMA+ cells/mm2
WT
Chi3l1-/-
Chi3l1 OE
MTB MIC 2 weeks
D
CK14+ Calponin 1+ Myoepithelial cells
CK14+ Calponin 1+ cells/mm2
WT
Chi3l1-/-
Chi3l1 OE
MTB MIC 2 weeks
E
CK14+ P63+ Myoepithelial cells
CK14+ P63+ cells/mm2
WT
Chi3l1-/-
Chi3l1 OE
MTB MIC 2 weeks
F
CK14+ Calponin 1+ P63+ Myoepithelial cells
CK14+ Calponin 1+ P63+ cells/mm2
WT
Chi3l1-/-
Chi3l1 OE
MTB MIC 2 weeks
G
Merge +DAPI
co-localization
CK5
CK14
CK8
MIC 2 week
Chi3l1 WT
Chi3l1-/-
Chi3l1 OE
10μm
H
CK14+ CK5+ Myoepithelial cells
CK14+ CK5+ cells/mm2
WT
Chi3l1-/-
Chi3l1 OE
MTB MIC 2 weeks
I
Epithelial Ki67 levels
%Ki67+ Pan-ck+ cells
WT
Chi3l1-/-
Chi3l1 OE
MTB MIC 2 weeks
J
Epithelial PCNA levels
%PCNA+ Pan-ck+ cells
WT
Chi3l1-/-
Chi3l1 OE
MTB MIC 2 weeks
M
CK14+ CK5+ Myoepithelial cells
CK14+ CK5+ cells/mm2
IgG2a
anti-Ly6G
MTB MIC Chi3l1 OE
2 weeks
K
Merge
+DAPI +Pan-ck
Ki67
PCNA
MIC 2 week
Chi3l1 WT
Chi3l1-/-
Chi3l1 OE
10μm
L
Merge +DAPI
co-localization
CK5
CK14
CK8
MIC Chi3l1 OE 2 week
IgG2a
Anti-Ly6G
10μm

Supplementary Figure 3: Genetic ablation of Chi3l1 prevents myoepithelial layer dissolution, whereas Chi3l1 overexpression accelerates it. Related to Figure 1.

(A) Fluorescent IHC staining of WT, *Chi3l1*$^{-/-}$ and Chi3l1 OE MIC mammary glands at 2 weeks post-induction using antibodies against CK14, α-SMA, and CK8, counterstained with DAPI. **(B)** Fluorescent IHC staining of WT, *Chi3l1*$^{-/-}$ and Chi3l1 OE MIC mammary glands at 2 weeks post induction using antibodies against CK14, calponin 1, P63, and CK8, counterstained with DAPI. **(C-F)** Quantification of total CK14+ α-SMA+, CK14+ calponin 1+, CK14+ P63+ and CK14+ calponin 1+ P63+ cells normalized to total analyzed area in WT (n = 6), *Chi3l1*$^{-/-}$ (n = 7), and Chi3l1 OE (n = 9) MIC mammary glands at 2 weeks post induction. Derived from the staining shown in A,B. **(G)** Fluorescent IHC staining of WT, *Chi3l1*$^{-/-}$ and Chi3l1 OE MIC mammary glands at 2 weeks post-induction using antibodies against CK14, CK5, and CK8, counterstained with DAPI. **(H)** Quantification of total CK14+ CK5+ cells normalized to total analyzed area in WT (n = 6), *Chi3l1*$^{-/-}$ (n = 7), and Chi3l1 OE (n = 9) MIC mammary glands at 2 weeks post induction. Derived from the staining shown in G. **(I,J)** Quantification of total Pan-ck+ Ki67+ and Pan-ck+ PCNA+ cells as a percentage of total cells in WT (n = 6), *Chi3l1*$^{-/-}$ (n = 7), and Chi3l1 OE (n = 9) MIC mammary glands at 2 weeks post induction. Derived from the staining shown in K. **(K)** Fluorescent IHC staining of WT, *Chi3l1*$^{-/-}$ and Chi3l1 OE MIC mammary glands at 2 weeks post induction using antibodies against PCNA, Ki67, and Pan-ck, counterstained with DAPI. **(L)** Fluorescent IHC staining of mammary tissue from Chi3l1 OE MIC mice treated with anti-Ly6G or IgG2a isotype control for 2 weeks, using antibodies against CK14, CK5, and CK8, counterstained with DAPI. **(M)** Quantification of total CK14+ CK5+ cells normalized to total analyzed area in IgG2a (n = 6) and anti-Ly6G (n = 8) treated Chi3l1 OE MIC mammary glands at 2 weeks post induction. Derived from the staining shown in L. Ns: not significant, *$p<0.05$, **$p<0.01$, ***$p<0.001$ and ****$p<0.0001$ calculated by One-Way Anova with Tukey's post-hoc test. Mann-Whitney non-parametric test was used for M. Data are presented as mean ± SEM. Each data point is an independent biological sample, N represents the number of independent biological replicates. Analysis was performed by the HALO (Indica lab) software. Quantification was performed on whole-slide scans of the entire mammary gland section.

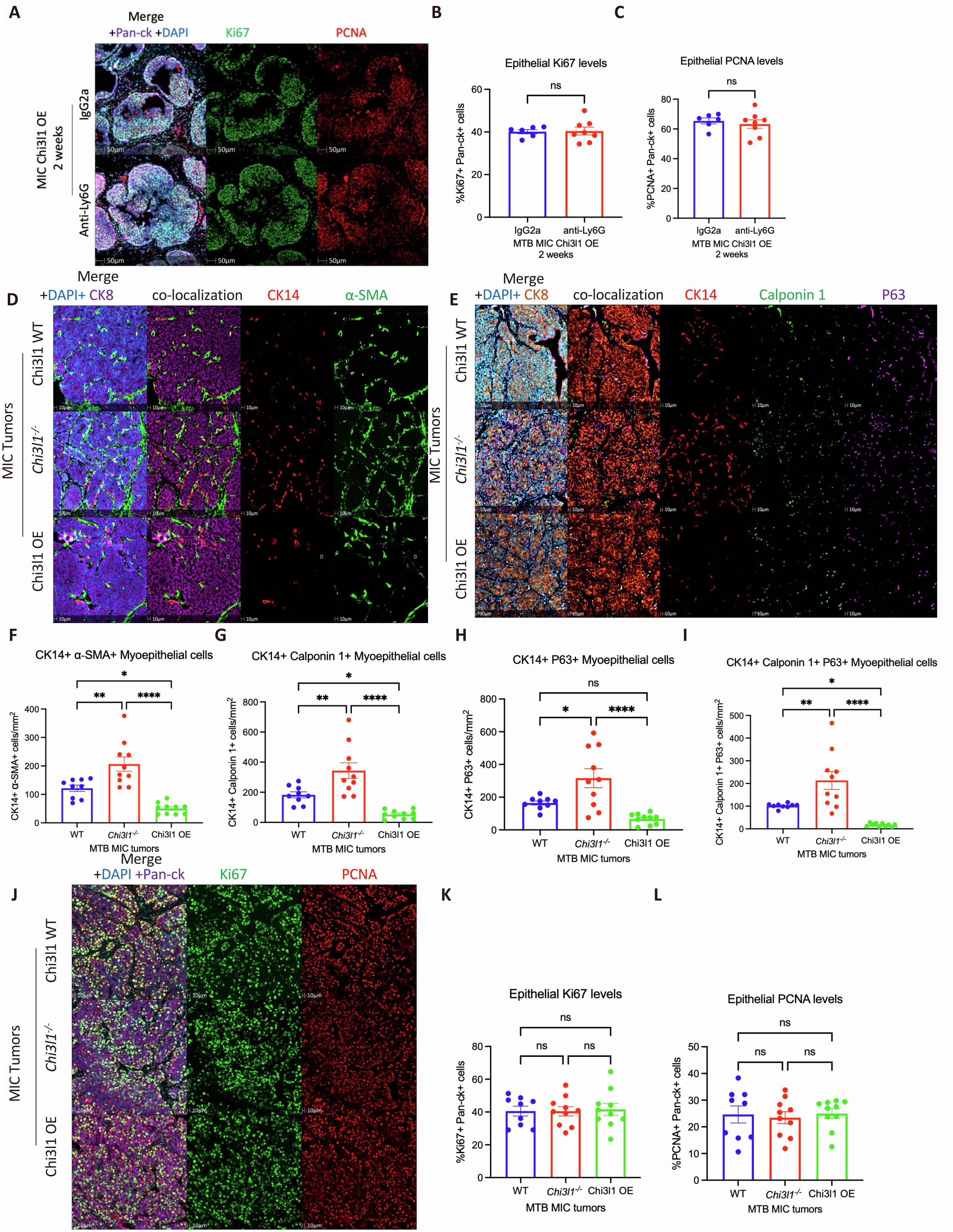
A
Merge
+Pan-ck +DAPI
Ki67
PCNA
MIC Chi3l1 OE
2 weeks
IgG2a
Anti-Ly6G
B
Epithelial Ki67 levels
%Ki67+ Pan-ck+ cells
IgG2a
anti-Ly6G
MTB MIC Chi3l1 OE
2 weeks
C
Epithelial PCNA levels
%PCNA+ Pan-ck+ cells
D
Merge
+DAPI+ CK8
co-localization
CK14
α-SMA
MIC Tumors
Chi3l1 WT
Chi3l1-/-
Chi3l1 OE
E
Calponin 1
P63
F
CK14+ α-SMA+ Myoepithelial cells
CK14+ α-SMA+ cells/mm2
WT
Chi3l1 OE
MTB MIC tumors
G
CK14+ Calponin 1+ Myoepithelial cells
CK14+ Calponin 1+ cells/mm2
H
CK14+ P63+ Myoepithelial cells
CK14+ P63+ cells/mm2
I
CK14+ Calponin 1+ P63+ Myoepithelial cells
CK14+ Calponin 1+ P63+ cells/mm2
J
+DAPI +Pan-ck
K
L

Supplementary Figure 4: Chi3l1 ablation or overexpression does not impact on cancer cell proliferation. Related to Figure 1. **(A)**. Fluorescent IHC staining of mammary tissue from Chi3l1 OE MIC mice treated with anti-Ly6G or IgG2a isotype control for 2 weeks, using antibodies against PCNA, Ki67, and Pan-ck, counterstained with DAPI. **(B,C)** Quantification of total Pan-ck+ Ki67+ and Pan-ck+ PCNA+ cells as a percentage of total cells in IgG2a (n = 6) and anti-Ly6G (n = 8) treated Chi3l1 OE MIC mammary glands at 2 weeks post induction**.** Derived from the staining shown in A. **(D)** Fluorescent IHC staining of WT, *Chi3l1*$^{-/-}$ and Chi3l1 OE MIC end-stage mammary tumors using antibodies against CK14, α-SMA, and CK8, counterstained with DAPI. **(E)** Fluorescent IHC staining of WT, *Chi3l1*$^{-/-}$ and Chi3l1 OE MIC end-stage mammary tumors using antibodies against CK14, calponin 1, P63, and CK8, counterstained with DAPI. **(F-I)** Quantification of total CK14+ α-SMA+, CK14+ calponin 1+, CK14+ P63+ and CK14+ calponin 1+ P63+ cells normalized to total analyzed area in WT (n = 9), *Chi3l1*$^{-/-}$ (n = 10), and Chi3l1 OE (n = 10) MIC mammary tumors. Derived from the staining shown in D,E. **(J)** Fluorescent IHC staining of WT, *Chi3l1*$^{-/-}$ and Chi3l1 OE MIC end-stage mammary tumors using antibodies against PCNA, Ki67, and Pan-ck, counterstained with DAPI. **(K,L)** Quantification of total Pan-ck+ Ki67+ and Pan-ck+ PCNA+ cells as a percentage of total cells in WT (n = 9), *Chi3l1*$^{-/-}$ (n = 10), and Chi3l1 OE (n = 10) MIC mammary tumors. Derived from the staining shown in J. Ns: not significant, *p<0.05, **p<0.01 and ****p<0.0001 calculated by One-Way Anova with Tukey's post-hoc test. Mann-Whitney non-parametric test was used for B,C. Data are presented as mean ± SEM. Each data point is an independent biological sample, *N* represents the number of independent biological replicates. Analysis was performed by the HALO (Indica lab) software. Quantification was performed on whole-slide scans of the entire mammary gland section.

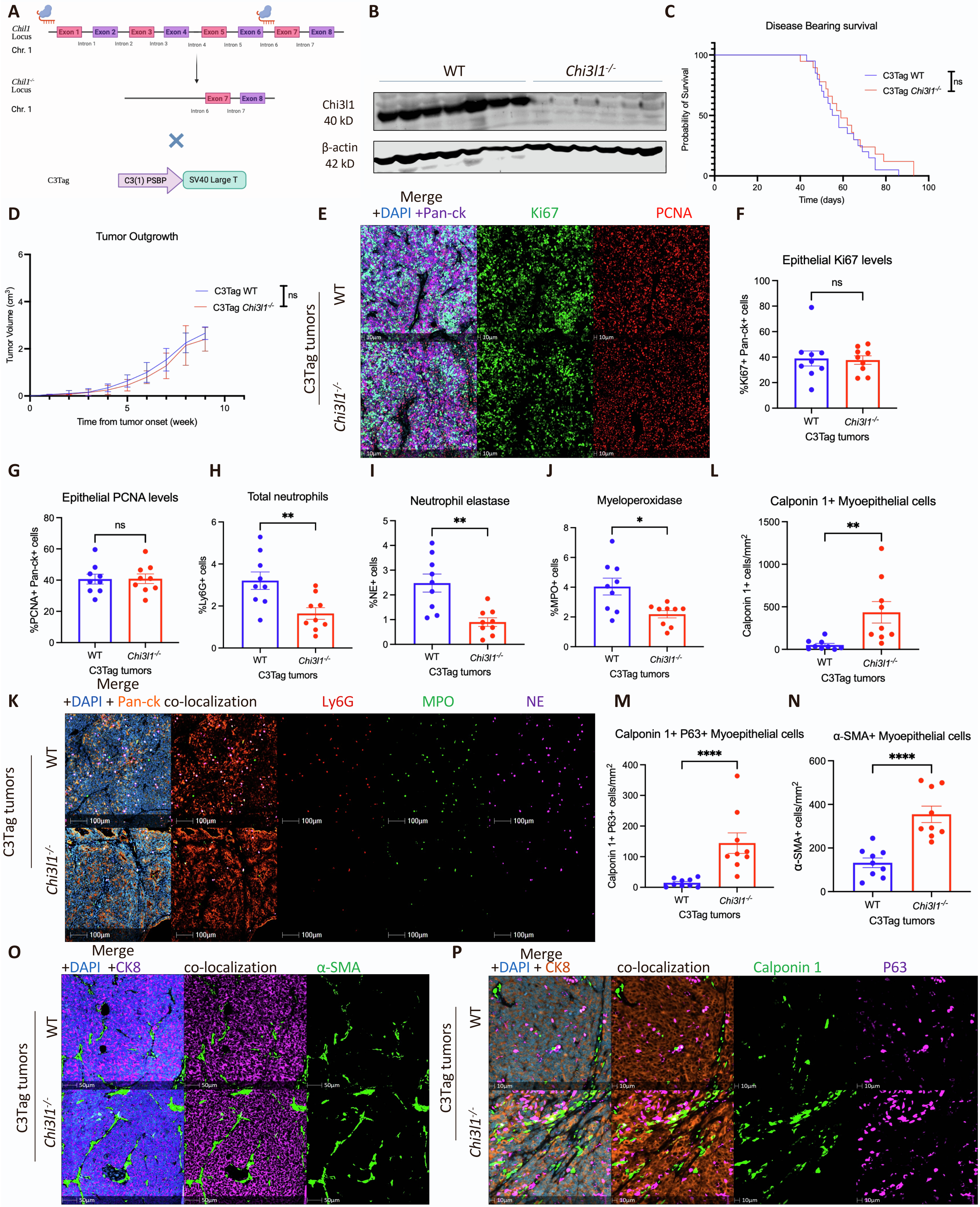
A
Chil1 Locus
Chr. 1
Exon 1
Exon 2
Exon 3
Exon 4
Exon 5
Exon 6
Exon 7
Exon 8
Intron 1
Intron 2
Intron 3
Intron 4
Intron 5
Intron 6
Intron 7
Chil1-/- Locus
C3Tag
C3(1) PSBP
SV40 Large T
B
WT
Chi3l1-/-
Chi3l1
40 kD
β-actin
42 kD
C
Disease Bearing survival
Probability of Survival
Time (days)
C3Tag WT
C3Tag Chi3l1-/-
ns
D
Tumor Outgrowth
Tumor Volume (cm3)
Time from tumor onset (week)
C3Tag WT
C3Tag Chi3l1-/-
ns
E
Merge
+DAPI +Pan-ck
Ki67
PCNA
C3Tag tumors
WT
Chi3l1-/-
F
Epithelial Ki67 levels
%Ki67+ Pan-ck+ cells
ns
C3Tag tumors
G
Epithelial PCNA levels
%PCNA+ Pan-ck+ cells
ns
H
Total neutrophils
%Ly6G+ cells
**
I
Neutrophil elastase
%NE+ cells
**
J
Myeloperoxidase
%MPO+ cells
*
L
Calponin 1+ Myoepithelial cells
Calponin 1+ cells/mm2
**
K
Merge
+DAPI + Pan-ck co-localization
Ly6G
MPO
NE
100μm
M
Calponin 1+ P63+ Myoepithelial cells
Calponin 1+ P63+ cells/mm2
****
N
α-SMA+ Myoepithelial cells
α-SMA+ cells/mm2
****
O
Merge
+DAPI +CK8
co-localization
α-SMA
50μm
P
Merge
+DAPI + CK8
co-localization
Calponin 1
P63
10μm

Supplementary Figure 5: Characterization of Chi3l1-deficient C3Tag GEMM. Related to Figure 1.
(A) Schematic representation of the C3Tag *Chi3l1*$^{-/-}$ GEMM used in this study, created with BioRender.com. **(B)** Immunoblots for Chi3l1 and β-actin on WT ($n = 7$) and *Chi3l1*$^{-/-}$ ($n = 6$) C3Tag mammary endpoint tumors. **(C)** Disease-bearing survival of WT ($n = 20$) and *Chi3l1*$^{-/-}$ ($n = 19$) C3Tag mammary tumor-bearing mice measured from day of measurable tumor (onset) to humane endpoint. Statistical analysis by Mantel–Cox test. **(D)** Primary tumors growth in WT ($n = 7$) and *Chi3l1*$^{-/-}$ ($n = 10$) C3Tag mammary tumor-bearing mice. Tumors were measured and tracked weekly by a caliper. **(E)** Fluorescent IHC staining of WT and *Chi3l1*$^{-/-}$ C3Tag end-stage mammary tumors, using antibodies against Ki67, PCNA and Pan-ck, counterstained with DAPI. **(F,G)** Quantification of total Pan-ck+ Ki67+ and Pan-ck+ PCNA+ cells as a percentage of total cells in WT ($n = 9$) and *Chi3l1*$^{-/-}$ ($n = 9$) C3Tag end-stage mammary tumors. Derived from the staining shown in E. **(H-J)** Quantification of total Ly6G+, NE+ and MPO+ cells as a percentage of total cells in WT ($n = 9$) and *Chi3l1*$^{-/-}$ ($n = 9$) C3Tag end-stage mammary tumors. Derived from the staining shown in K. **(K)** Fluorescent IHC staining of WT and *Chi3l1*$^{-/-}$ C3Tag end-stage mammary tumors using antibodies against Ly6G, MPO, NE and Pan-ck, counterstained with DAPI. **(L-N)** Quantification of total Calponin 1+, calponin 1+ P63+ and α-SMA+ cells normalized to total analyzed area in WT ($n = 9$) and *Chi3l1*$^{-/-}$ ($n = 9$) C3Tag end-stage mammary tumors. Derived from the staining shown in O,P. **(O)** Fluorescent IHC staining of WT and *Chi3l1*$^{-/-}$ C3Tag end-stage mammary tumors using antibodies against α-SMA, and CK8, counterstained with DAPI. **(P)** Fluorescent IHC staining of WT and *Chi3l1*$^{-/-}$ C3Tag end-stage mammary tumors using antibodies against calponin 1, P63 and CK8, counterstained with DAPI. Ns: not significant, *$p<0.05$, **$p<0.01$ and ***$p<0.001$ calculated by Mann-Whitney non-parametric test. Data are presented as mean ± SEM. Each data point is an independent biological sample, *N* represents the number of independent biological replicates. Analysis was performed by the HALO (Indica lab) software. Quantification was performed on whole-slide scans of the entire mammary gland section.

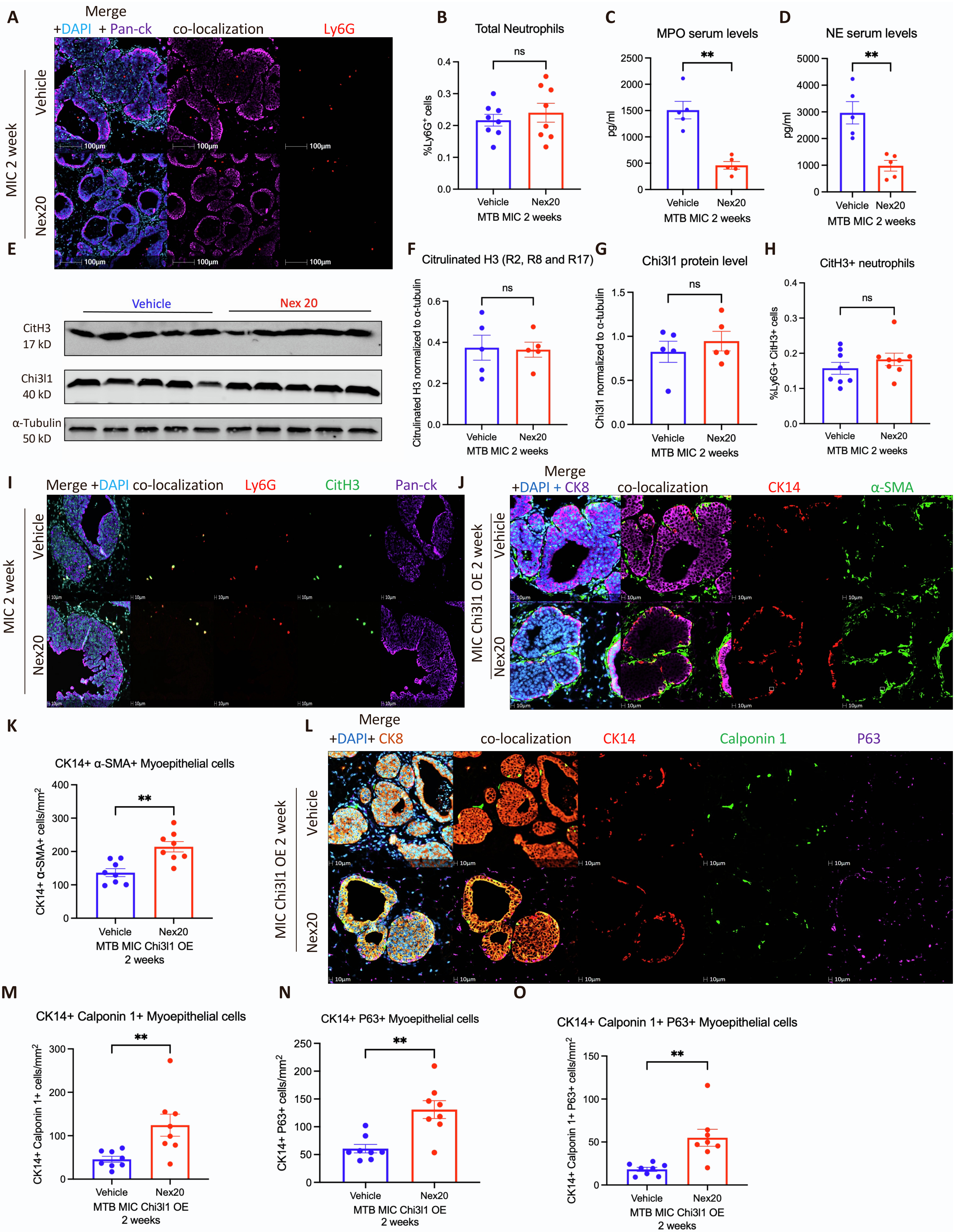
A
Merge
+DAPI + Pan-ck
co-localization
Ly6G
MIC 2 week
Vehicle
Nex20
100µm
B
Total Neutrophils
ns
%Ly6G+ cells
Vehicle
Nex20
MTB MIC 2 weeks
C
MPO serum levels
**
pg/ml
Vehicle
Nex20
MTB MIC 2 weeks
D
NE serum levels
**
pg/ml
Vehicle
Nex20
MTB MIC 2 weeks
E
Vehicle
Nex 20
CitH3
17 kD
Chi3l1
40 kD
α-Tubulin
50 kD
F
Citrulinated H3 (R2, R8 and R17)
ns
Citulinated H3 normalized to α-tubulin
Vehicle
Nex20
MTB MIC 2 weeks
G
Chi3l1 protein level
ns
Chi3l1 normalized to α-tubulin
Vehicle
Nex20
MTB MIC 2 weeks
H
CitH3+ neutrophils
ns
%Ly6G+ CitH3+ cells
Vehicle
Nex20
MTB MIC 2 weeks
I
Merge +DAPI co-localization
Ly6G
CitH3
Pan-ck
MIC 2 week
Vehicle
Nex20
J
Merge
+DAPI + CK8
co-localization
CK14
α-SMA
MIC Chi3l1 OE 2 week
Vehicle
Nex20
K
CK14+ α-SMA+ Myoepithelial cells
**
CK14+ α-SMA+ cells/mm2
Vehicle
Nex20
MTB MIC Chi3l1 OE
2 weeks
L
Merge
+DAPI+ CK8
co-localization
CK14
Calponin 1
P63
MIC Chi3l1 OE 2 week
Vehicle
Nex20
10µm
M
CK14+ Calponin 1+ Myoepithelial cells
**
CK14+ Calponin 1+ cells/mm2
Vehicle
Nex20
MTB MIC Chi3l1 OE
2 weeks
N
CK14+ P63+ Myoepithelial cells
**
CK14+ P63+ cells/mm2
Vehicle
Nex20
MTB MIC Chi3l1 OE
2 weeks
O
CK14+ Calponin 1+ P63+ Myoepithelial cells
**
CK14+ Calponin 1+ P63+ cells/mm2
Vehicle
Nex20
MTB MIC Chi3l1 OE
2 weeks

Supplementary Figure 6: Inhibiting neutrophil degranulation in Chi3l1 OE mice rescues myoepithelial layer loss. Related to Figure 2. **(A)** Fluorescent IHC staining of mammary glands from vehicle or Nex20-treated WT MIC mice at 2 weeks post induction using antibodies against Ly6G, and Pan-ck, counterstained with DAPI. **(B)** Quantification of total Ly6G+ cells as a percentage of total cells in vehicle (*n* = 8), and Nex20-treated (*n* = 8) MIC mammary glands at 2 weeks post induction. Derived from the staining shown in A. **(C, D)** ELISA for serum MPO and NE from vehicle (*n* = 5) and Nex20 (*n* = 5) treated WT MIC mice at 2 weeks post-induction. **(E)** Immunoblots for CitH3, Chi3l1 and α-tubulin on vehicle (*n* = 5) and Nex20 (*n* = 5) treated WT MIC mammary glands at 2 weeks post-induction. Protein was extracted form flash frozen mammary gland and subjected to immunoblot analysis. **(F,G)** Quantification of CitH3 and Chi3l1 immunoblot normalized to α-tubulin. **(H)** Quantification of total Ly6G+ CitH3+ cells as a percentage of total cells in vehicle (*n* = 8), and Nex20-treated (*n* = 8) MIC mammary glands at 2 weeks post induction. Derived from the staining shown in I. **(I)** Fluorescent IHC staining of mammary glands from vehicle or Nex20-treated WT MIC mice at 2 weeks post induction using antibodies against Ly6G, CitH3 and Pan-ck, counterstained with DAPI. **(J)** Fluorescent IHC staining of mammary glands from vehicle or Nex20-treated Chi3l1 OE MIC mice at 2 weeks post induction using antibodies against CK14, α-SMA, and CK8, counterstained with DAPI. **(K)** Quantification of total CK14+ α-SMA+ cells normalized to total analyzed area in vehicle (*n* = 8) or Nex20-treated (*n* = 8) Chi3l1 OE MIC mammary glands at 2 weeks post induction. Derived from the staining shown in J. **(L)** Fluorescent IHC staining of of mammary glands from vehicle or Nex20-treated Chi3l1 OE MIC mice at 2 weeks post induction using antibodies against CK14, calponin 1, P63 and CK8, counterstained with DAPI (blue). **(M-O)** Quantification of total CK14+ α-SMA+, CK14+ calponin 1+, CK14+ P63+ and CK14+ calponin 1+ P63+ cells normalized to total analyzed area in vehicle (*n* = 8) or Nex20-treated (*n* = 8) Chi3l1 OE MIC mammary glands at 2 weeks post induction. Derived from the staining shown in L. Ns: not significant, $*p<0.05$ and $**p<0.01$ calculated by Mann-Whitney non-parametric test. Data are presented as mean ± SEM. Each data point is an independent biological sample, *N* represents the number of independent biological replicates. Analysis was performed by the HALO (Indica lab) software. Quantification was performed on whole-slide scans of the entire mammary gland section.

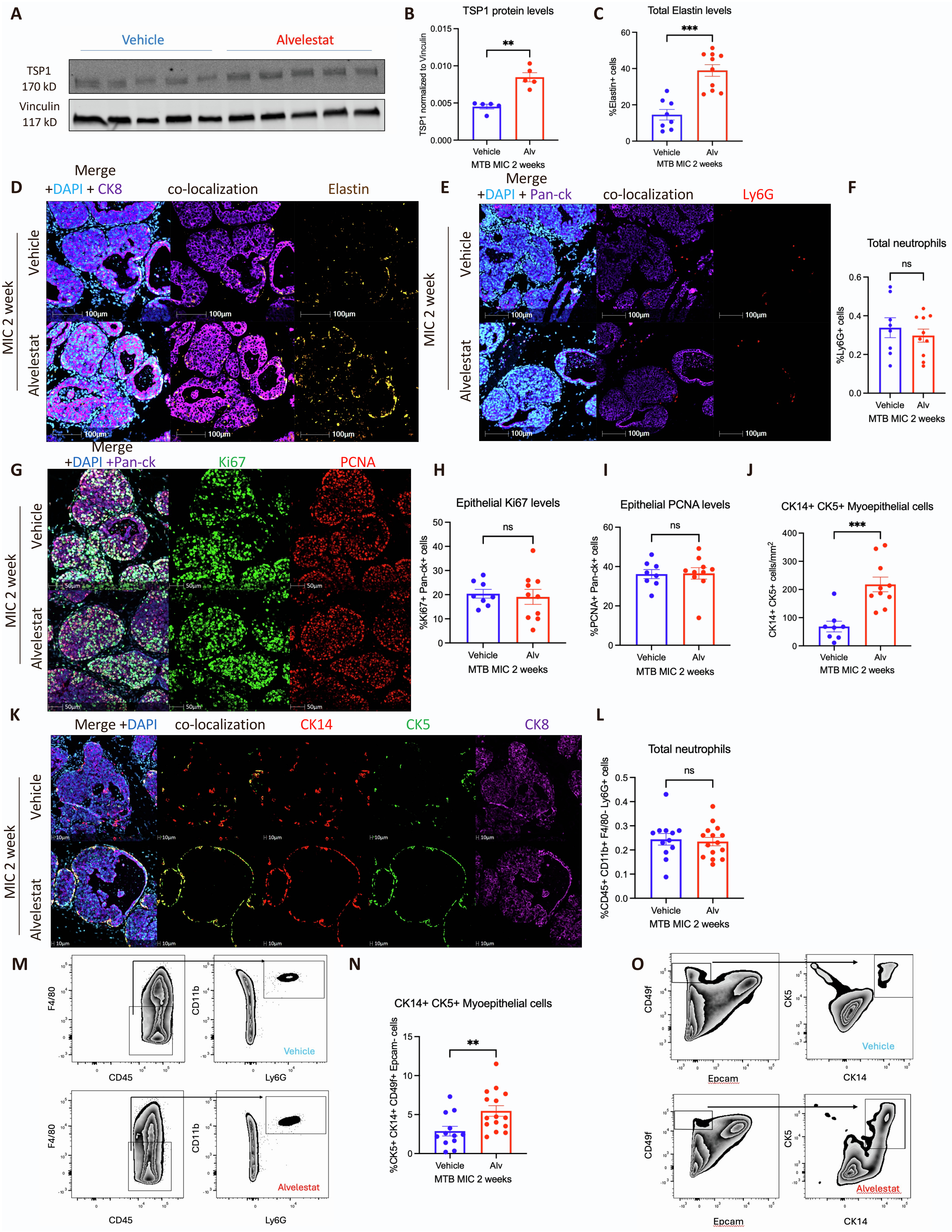
A
Vehicle
Alvelestat
TSP1
170 kD
Vinculin
117 kD
B
TSP1 protein levels
TSP1 normalized to Vinculin
**
Vehicle
Alv
MTB MIC 2 weeks
C
Total Elastin levels
%Elastin+ cells
***
Vehicle
Alv
MTB MIC 2 weeks
D
Merge
+DAPI + CK8
co-localization
Elastin
MIC 2 week
Vehicle
Alvelestat
100µm
E
Merge
+DAPI + Pan-ck
co-localization
Ly6G
MIC 2 week
Vehicle
Alvelestat
100µm
F
Total neutrophils
%Ly6G+ cells
ns
Vehicle
Alv
MTB MIC 2 weeks
G
Merge
+DAPI +Pan-ck
Ki67
PCNA
MIC 2 week
Vehicle
Alvelestat
50µm
H
Epithelial Ki67 levels
%Ki67+ Pan-ck+ cells
ns
Vehicle
Alv
MTB MIC 2 weeks
I
Epithelial PCNA levels
%PCNA+ Pan-ck+ cells
ns
Vehicle
Alv
MTB MIC 2 weeks
J
CK14+ CK5+ Myoepithelial cells
CK14+ CK5+ cells/mm2
***
Vehicle
Alv
MTB MIC 2 weeks
K
Merge +DAPI
co-localization
CK14
CK5
CK8
MIC 2 week
Vehicle
Alvelestat
10µm
L
Total neutrophils
%CD45+ CD11b+ F4/80- Ly6G+ cells
ns
Vehicle
Alv
MTB MIC 2 weeks
M
F4/80
CD45
CD11b
Ly6G
Vehicle
Alvelestat
N
CK14+ CK5+ Myoepithelial cells
%CK5+ CK14+ CD49f+ Epcam- cells
**
Vehicle
Alv
MTB MIC 2 weeks
O
CD49f
Epcam
CK5
CK14
Vehicle
Alvelestat

Supplementary Figure 7: NE inhibition increases myoepithelial content without impacting neutrophil numbers or cancer cell proliferation. Related to Figure 3.

(A) Immunoblots for TSP1 and vinculin on vehicle (*n* = 5) or Alvelestat (*n* = 5) treated WT MIC mammary glands at 2 weeks post-induction. **(B)** Quantification of TSP1 immunoblot normalized to vinculin. **(C)** Quantification of elastin+ cells as a percentage of total cells in vehicle (*n* = 8), and Alvelestat (*n* = 10) treated MIC mammary glands at 2 weeks post induction. Derived from the staining shown in D. **(D)** Fluorescent IHC staining of mammary glands from vehicle or Alvelestat-treated WT MIC mice at 2 weeks post induction using antibodies against elastin and CK8, counterstained with DAPI. **(E)** Fluorescent IHC staining of mammary glands from vehicle or Alvelestat-treated WT MIC mice at 2 weeks post induction using antibodies against Ly6G and Pan-ck, counterstained with DAPI. **(F)** Quantification of Ly6G+ cells as a percentage of total cells in vehicle (*n* = 8), and Alvelesta (*n* = 10) treated MIC mammary glands at 2 weeks post induction. Derived from the staining shown in E. **(G)** Fluorescent IHC staining of mammary glands from vehicle or Alvelestat-treated WT MIC mice at 2 weeks post induction using antibodies against Ki67, PCNA and Pan-ck, counterstained with DAPI. **(H,I)** Quantification of total Pan-ck+ Ki67+ and Pan-ck+ PCNA+ cells as a percentage of total cells in vehicle (*n* = 8), and Alvelestat (*n* = 10) treated MIC mammary glands at 2 weeks post induction. Derived from the staining shown in G. **(J)** Quantification of total CK14+ CK5+ cells normalized to total analyzed area in vehicle (*n* = 8), and Alvelestat (*n* = 10) treated MIC mammary glands at 2 weeks post induction. Derived from the staining shown in K. **(K)** Fluorescent IHC staining of mammary glands from vehicle or Alvelestat-treated WT MIC mice at 2 weeks post induction using antibodies against CK14, CK5 and CK8 counterstained with DAPI (blue). **(L)** Quantification of CD45+ Cd11b+ F4/80- Ly6G cells as percent of total live cells in vehicle (*n* = 12), and Alvelestat (*n* = 15) treated MIC mammary glands at 2 weeks post induction. **(M)** Representative FACS gating strategy of neutrophil populations in WT MIC mammary glands treated with Alvelestat- or vehicle control for 2 weeks. CD45%+ cells were sorted based on expression F4/80 (macrophage marker) and Cd11b (myeloid marker). Neutrophils (Ly6G+) cells were gated out of the CD11b+ F4/80- cells**.** **(N)** Quantification of Epcam- CD49f+ CK14+ CK5+ cells as percent of total live cells in vehicle (*n* = 12), and Alvelestat (*n* = 15) treated MIC mammary glands at 2 weeks post induction. **(O)** Representative FACS gating strategy of myoepithelial populations in WT MIC mammary glands treated with Alvelestat- or vehicle control for 2 weeks. Briefly, viable cells were sorted based on expression of EPCAM (luminal marker) and CD49f (Myoepithelial basal marker). Myoepithelial cells (CK14+ CK5+) were gated out of EPCAM- CD49f+ population. Ns: not significant, $^{**}p<0.01$, $^{***}p<0.001$ and $^{****}p<0.0001$ calculated by Mann-Whitney non-parametric test. Data are presented as mean ± SEM. Each data point is an independent biological sample, *N* represents the number of independent biological replicates. Analysis was performed by the HALO (Indica lab) software. Quantification was performed on whole-slide scans of the entire mammary gland section.

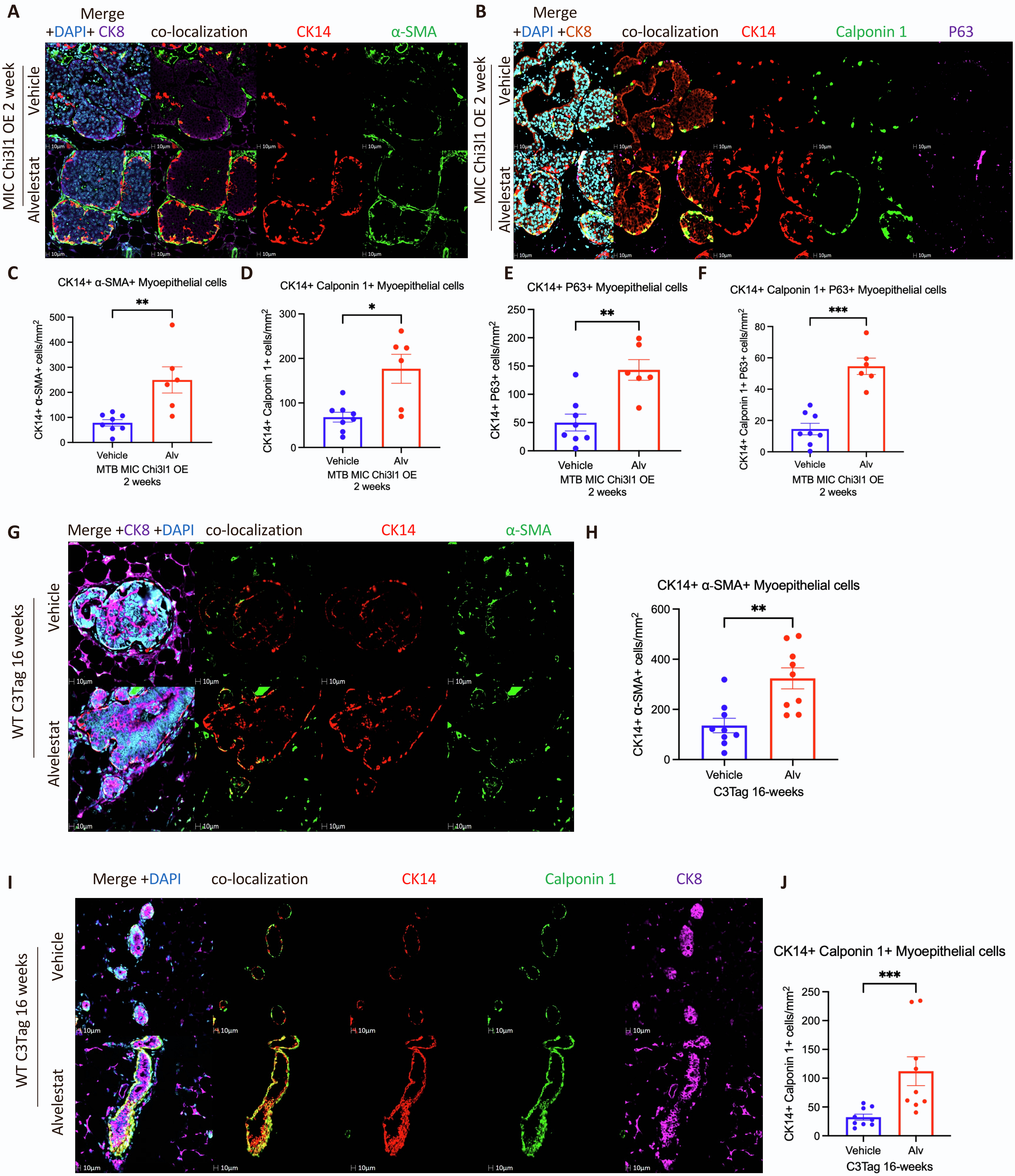

A
Merge +DAPI+ CK8
co-localization
CK14
α-SMA
MIC Chi3l1 OE 2 week
Vehicle
Alvelestat
B
Merge +DAPI +CK8
co-localization
CK14
Calponin 1
P63
MIC Chi3l1 OE 2 week
Vehicle
Alvelestat
C
CK14+ α-SMA+ Myoepithelial cells
CK14+ α-SMA+ cells/mm²
Vehicle
Alv
MTB MIC Chi3l1 OE 2 weeks
D
CK14+ Calponin 1+ Myoepithelial cells
CK14+ Calponin 1+ cells/mm²
Vehicle
Alv
MTB MIC Chi3l1 OE 2 weeks
E
CK14+ P63+ Myoepithelial cells
CK14+ P63+ cells/mm²
Vehicle
Alv
MTB MIC Chi3l1 OE 2 weeks
F
CK14+ Calponin 1+ P63+ Myoepithelial cells
CK14+ Calponin 1+ P63+ cells/mm²
Vehicle
Alv
MTB MIC Chi3l1 OE 2 weeks
G
Merge +CK8 +DAPI
co-localization
CK14
α-SMA
WT C3Tag 16 weeks
Vehicle
Alvelestat
H
CK14+ α-SMA+ Myoepithelial cells
CK14+ α-SMA+ cells/mm²
Vehicle
Alv
C3Tag 16-weeks
I
Merge +DAPI
co-localization
CK14
Calponin 1
CK8
WT C3Tag 16 weeks
Vehicle
Alvelestat
J
CK14+ Calponin 1+ Myoepithelial cells
CK14+ Calponin 1+ cells/mm²
Vehicle
Alv
C3Tag 16-weeks

Supplementary Figure 8: Inhibiting NE in other GEMMs prevents myoepithelial layer dissolution. Related to Figure 3.
(A) Fluorescent IHC staining of mammary glands from vehicle or Alvelestat-treated Chi3l1 OE MIC mice at 2 weeks post induction using antibodies against CK14, α-SMA, and CK8, counterstained with DAPI. **(B)** Fluorescent IHC staining of of mammary glands from vehicle or Alvelestat-treated Chi3l1 OE MIC mice at 2 weeks post induction using antibodies against CK14, calponin 1, P63 and CK8, counterstained with DAPI. **(C-F)** Quantification of total CK14+ α-SMA+, CK14+ calponin 1+, CK14+ P63+ and CK14+ calponin 1+ P63+ cells normalized to total analyzed area in vehicle (*n* = 8) or Alvelestat (*n* = 6) treated Chi3l1 OE MIC mammary glands at 2 weeks post induction. Derived from the staining shown in A,B. **(G)** Fluorescent IHC staining of mammary glands from vehicle or Alvelestat-treated WT C3Tag mice at 16 weeks using antibodies against CK14, α-SMA, and CK8, counterstained with DAPI. **(H)** Quantification of total CK14+ α-SMA+ cells normalized to total analyzed area in vehicle (*n* = 9) or Alvelestat (*n* = 9) treated WT C3Tag mammary glands at 16 weeks. Derived from the staining shown in G. **(I)** Fluorescent IHC staining of mammary glands from vehicle or Alvelestat-treated WT C3Tag mice at 16 weeks using antibodies against calponin 1, CK14 and CK8, counterstained with DAPI. **(J)** Quantification of total CK14+ calponin 1+ cells normalized to total analyzed area in vehicle (*n* = 9) or Alvelestat (*n* = 9) treated WT C3Tag mammary glands at 16 weeks. Derived from the staining shown in I. *$p<0.05$ and **$p<0.01$ calculated by Mann-Whitney non-parametric test. Data are presented as mean ± SEM. Each data point is an independent biological sample, *N* represents the number of independent biological replicates. Analysis was performed by the HALO (Indica lab) software. Quantification was performed on whole-slide scans of the entire mammary gland section.

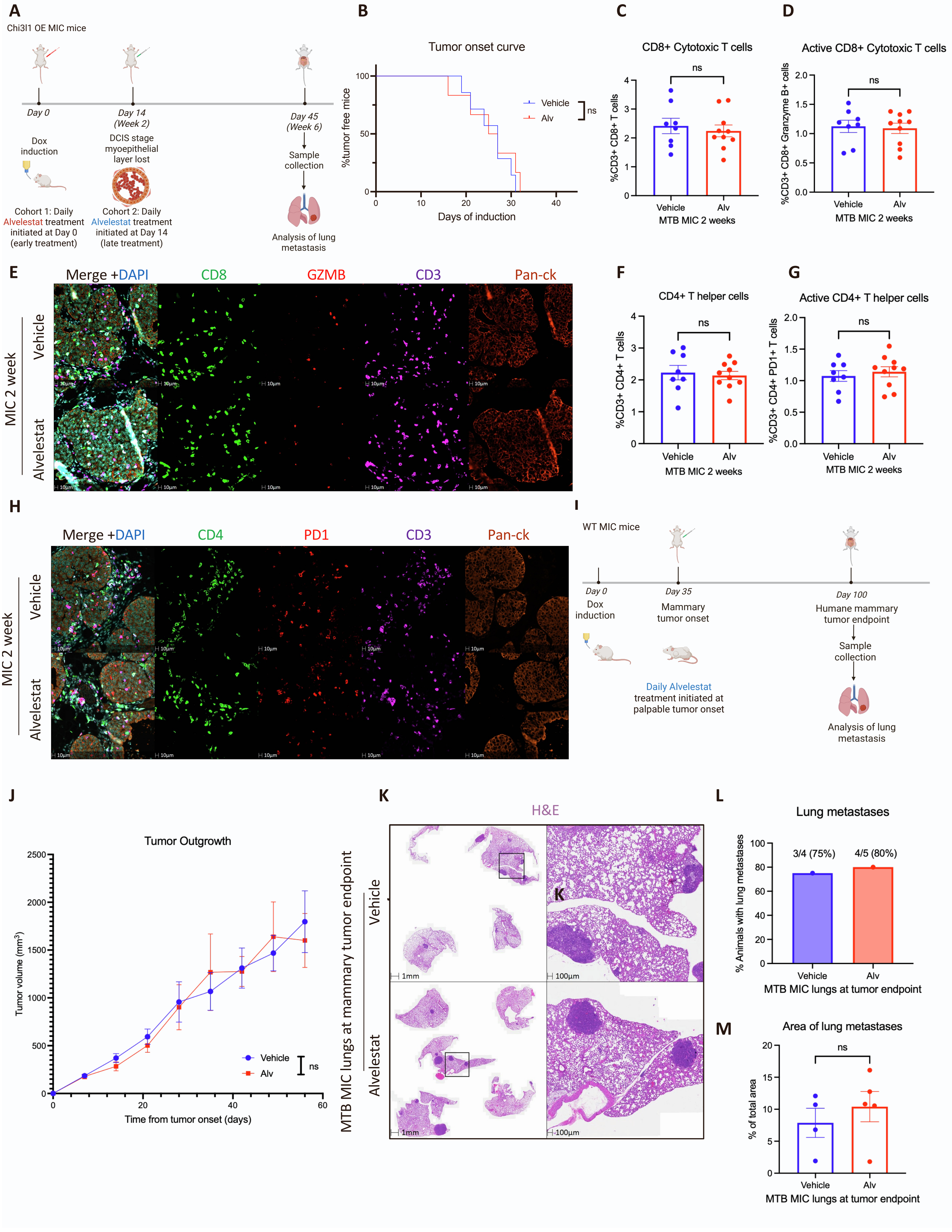

A
Chi3l1 OE MIC mice
Day 0
Day 14 (Week 2)
Day 45 (Week 6)
Dox induction
DCIS stage myoepithelial layer lost
Sample collection
Cohort 1: Daily Alvelestat treatment initiated at Day 0 (early treatment)
Cohort 2: Daily Alvelestat treatment initiated at Day 14 (late treatment)
Analysis of lung metastasis
B
Tumor onset curve
%tumor free mice
Days of induction
Vehicle
Alv
ns
C
CD8+ Cytotoxic T cells
%CD3+ CD8+ T cells
Vehicle
Alv
MTB MIC 2 weeks
D
Active CD8+ Cytotoxic T cells
%CD3+ CD8+ Granzyme B+ cells
MTB MIC 2 weeks
E
Merge +DAPI
CD8
GZMB
CD3
Pan-ck
MIC 2 week
Vehicle
Alvelestat
F
CD4+ T helper cells
%CD3+ CD4+ T cells
MTB MIC 2 weeks
G
Active CD4+ T helper cells
%CD3+ CD4+ PD1+ T cells
MTB MIC 2 weeks
H
Merge +DAPI
CD4
PD1
CD3
Pan-ck
MIC 2 week
Vehicle
Alvelestat
I
WT MIC mice
Day 0
Day 35
Day 100
Dox induction
Mammary tumor onset
Humane mammary tumor endpoint
Sample collection
Daily Alvelestat treatment initiated at palpable tumor onset
Analysis of lung metastasis
J
Tumor Outgrowth
Tumor volume (mm³)
Time from tumor onset (days)
Vehicle
Alv
ns
K
H&E
MTB MIC lungs at mammary tumor endpoint
Vehicle
Alvelestat
1mm
100µm
L
Lung metastases
% Animals with lung metastases
3/4 (75%)
4/5 (80%)
Vehicle
Alv
MTB MIC lungs at tumor endpoint
M
Area of lung metastases
% of total area
ns
Vehicle
Alv
MTB MIC lungs at tumor endpoint

Supplementary Figure 9: Inhibiting NE in established mammary tumor does not impact lung metastasis. Related to Figure 3. **(A)** Schematic representation of the experimental plan for early and delayed Alvelestat treatment in Chi3l1 OE MIC mice, created with BioRender.com. **(B)** Mammary tumor onset in Vehicle (*n* = 7) or Alvelestat (*n* = 6)-treated Chi3l1 OE MIC mice tracked by weekly physical palpation. Treatment started at doxycycline induction and continued daily for 6 weeks. Analysis by Log-rank test. **(C,D)** Quantification of total CD3+ CD8+, CD3+ CD8+ GzmB+ cells as a percentage of total cells in vehicle (*n* = 8), and Alvelestat (*n* = 10) treated MIC mammary glands at 2 weeks post induction. Derived from the staining shown in E. **(E)** Fluorescent IHC staining of mammary glands from vehicle or Alvelestat-treated Chi3l1 OE MIC mice at 2 weeks post induction using antibodies against GzmB, CD8, CD3 and Pan-ck, counterstained with DAPI. **(F,G)** Quantification of total CD3+CD4+ and CD3+ CD4+ PD1+ cells as a percentage of total cells in vehicle (*n* = 8), and Alvelestat (*n* = 10) treated MIC mammary glands at 2 weeks post induction. Derived from the staining shown in H. **(H)** Fluorescent IHC staining of mammary glands from vehicle or Alvelestat-treated Chi3l1 OE MIC mice at 2 weeks post induction using antibodies against PD1, CD4, CD3 and Pan-ck, counterstained with DAPI. **(I)** Schematic representation of the experimental plan for Alvelestat treatment in tumor-bearing WT MIC mice, created with BioRender.com. **(J)** Primary tumors growth in Vehicle (*n* = 4), and Alvelestat-treated (*n* = 5) WT MIC mammary tumor-bearing mice. Tumors were measured and tracked by a caliper. **(K)** Representative H&E staining of WT MIC lungs at mammary tumor endpoint after treatment with vehicle or Alvelestat from tumor onset and until humane end point. **(L)** Percentage of WT MIC mice with pulmonary metastases after treatment with vehicle (*n* = 4) or Alvelestat (*n* = 5) from tumor onset and until humane end point. **(M)** Quantification of total pulmonary metastatic area in WT MIC lungs at mammary tumor endpoint after treatment with vehicle (*n* = 4) or Alvelestat (*n* = 5) from tumor onset and until humane end point. Represented as percent of total lung area. Ns: Not significant; *p<0.05, **p<0.01, ***p<0.001 and ****p<0.0001 calculated by Mann-Whitney non-parametric test. Data are presented as mean ± SEM. Each data point is an independent biological sample, *N* represents the number of independent biological replicates. Analysis was performed by the HALO (Indica lab) software. Quantification was performed on whole-slide scans of the entire mammary gland section.

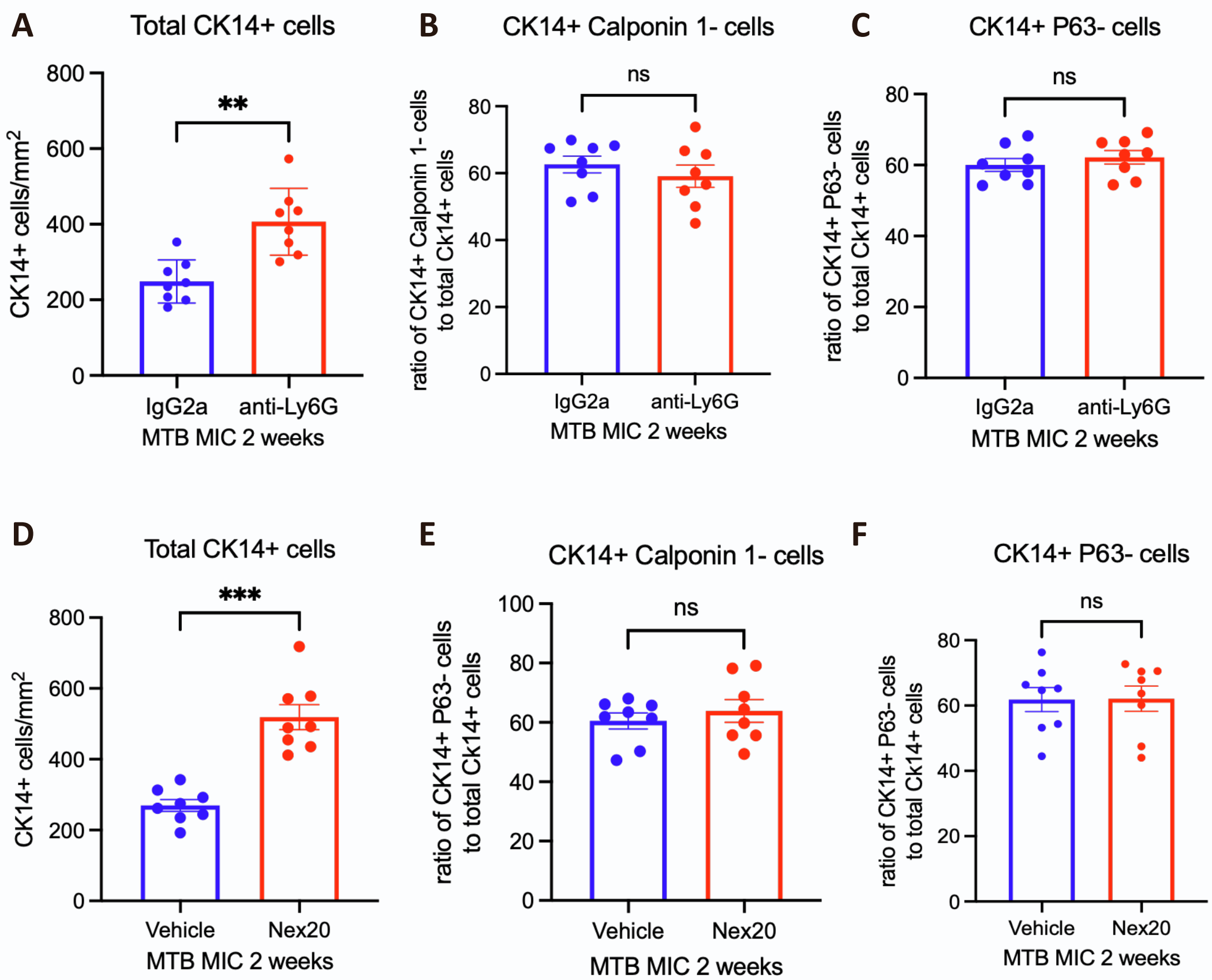
A
Total CK14+ cells
CK14+ cells/mm2
**
IgG2a
anti-Ly6G
MTB MIC 2 weeks
B
CK14+ Calponin 1- cells
ratio of CK14+ Calponin 1- cells to total Ck14+ cells
ns
IgG2a
anti-Ly6G
MTB MIC 2 weeks
C
CK14+ P63- cells
ratio of CK14+ P63- cells to total Ck14+ cells
ns
IgG2a
anti-Ly6G
MTB MIC 2 weeks
D
Total CK14+ cells
CK14+ cells/mm2
***
Vehicle
Nex20
MTB MIC 2 weeks
E
CK14+ Calponin 1- cells
ratio of CK14+ P63- cells to total Ck14+ cells
ns
Vehicle
Nex20
MTB MIC 2 weeks
F
CK14+ P63- cells
ratio of CK14+ P63- cells to total Ck14+ cells
ns
Vehicle
Nex20
MTB MIC 2 weeks

Supplementary Figure 10: Targeting neutrophils and their effector function does not affect myoepithelial cell differentiation. Related to Figure 3.

(A-C) Quantification of total CK14+ and the percentage of CK14+ calponin 1-, CK14+ P63- within total CK14+ in IgG2a (*n* = 8) and anti-Ly6G (*n* = 8) treated WT MIC mammary glands at 2 weeks post induction. Analyzed from staining performed in Figure 1A. **(D-E)** Quantification of total CK14+, and the percentage of CK14+ calponin 1-, CK14+ P63- within total CK14+ in vehicle (*n* = 8), and Nex20-treated (*n* = 8) MIC mammary glands at 2 weeks post induction. Analyzed from staining performed in Figure 2A. Ns: Not significant; **$p<0.01$ and ***$p<0.001$ calculated by Mann-Whitney non-parametric test. Data are presented as mean ± SEM. Each data point is an independent biological sample, *N* represents the number of independent biological replicates. Analysis was performed by the HALO (Indica lab) software. Quantification was performed on whole-slide scans of the entire mammary gland section.

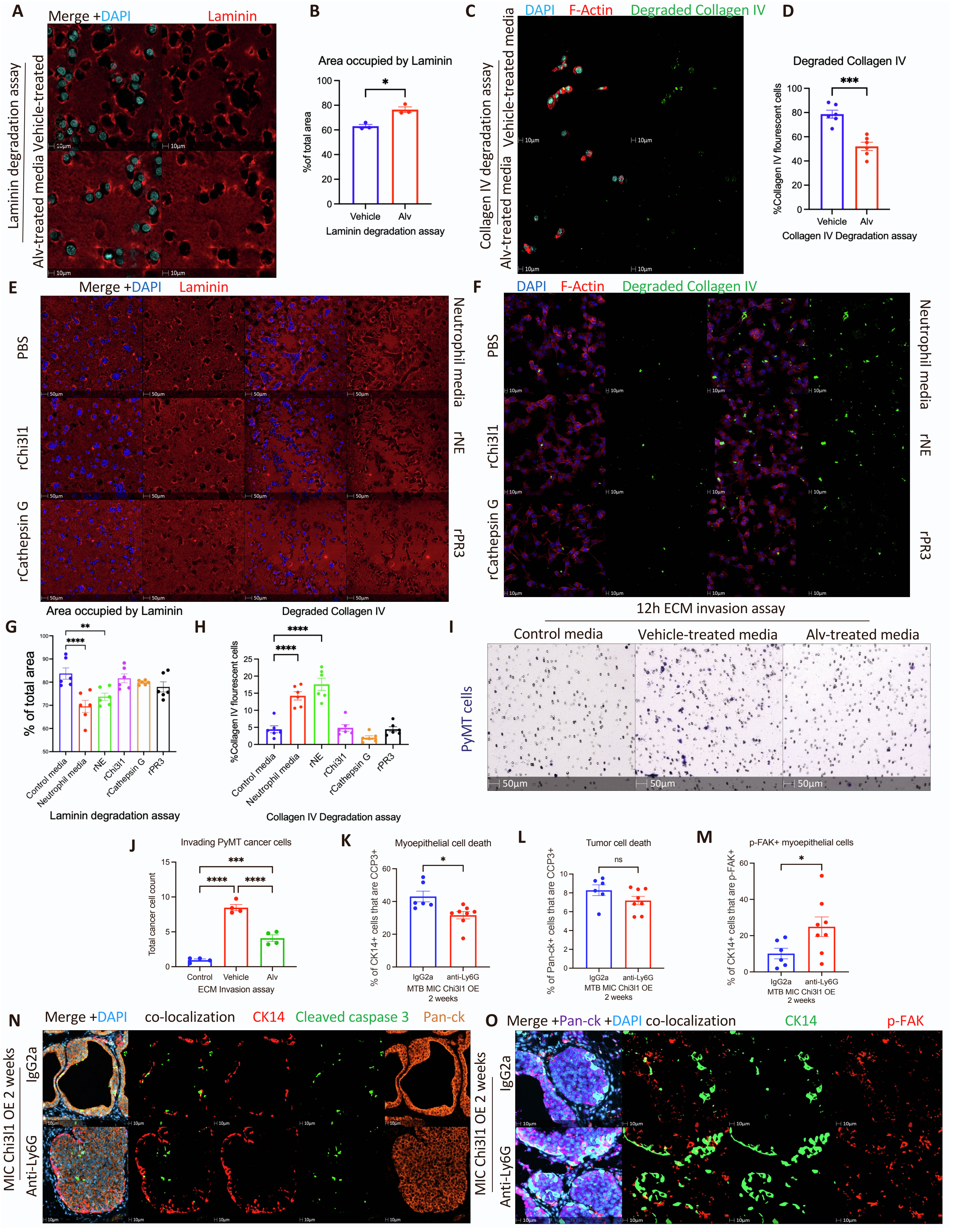

A
Merge +DAPI
Laminin
Laminin degradation assay
Vehicle-treated
Alv-treated media
B
Area occupied by Laminin
%of total area
Vehicle
Alv
Laminin degradation assay
C
DAPI F-Actin Degraded Collagen IV
Collagen IV degradation assay
Vehicle-treated media
Alv-treated media
D
Degraded Collagen IV
%Collagen IV flourescent cells
Vehicle
Alv
Collagen IV Degradation assay
E
Merge +DAPI Laminin
PBS
rChi3l1
rCathepsin G
Neutrophil media
rNE
rPR3
F
DAPI F-Actin Degraded Collagen IV
PBS
rChi3l1
rCathepsin G
Neutrophil media
rNE
rPR3
G
Area occupied by Laminin
% of total area
Control media
Neutrophil media
rNE
rChi3l1
rCathepsin G
rPR3
Laminin degradation assay
H
Degraded Collagen IV
%Collagen IV flourescent cells
Control media
Neutrophil media
rNE
rChi3l1
rCathepsin G
rPR3
Collagen IV Degradation assay
I
12h ECM invasion assay
Control media
Vehicle-treated media
Alv-treated media
PyMT cells
J
Invading PyMT cancer cells
Total cancer cell count
Control
Vehicle
Alv
ECM Invasion assay
K
Myoepithelial cell death
% of CK14+ cells that are CCP3+
IgG2a
anti-Ly6G
MTB MIC Chi3l1 OE
2 weeks
L
Tumor cell death
% of Pan-ck+ cells that are CCP3+
IgG2a
anti-Ly6G
MTB MIC Chi3l1 OE
2 weeks
M
p-FAK+ myoepithelial cells
% of CK14+ cells that are p-FAK+
IgG2a
anti-Ly6G
MTB MIC Chi3l1 OE
2 weeks
N
Merge +DAPI co-localization CK14 Cleaved caspase 3 Pan-ck
MIC Chi3l1 OE 2 weeks
IgG2a
Anti-Ly6G
O
Merge +Pan-ck +DAPI co-localization CK14 p-FAK
MIC Chi3l1 OE 2 weeks
IgG2a
Anti-Ly6G

Supplementary Figure 11: NE mediates ECM degradation and myoepithelial cell anoikis. Related to Figure 4 and 5.
(A) Staining for laminin and DAPI on PyMT+ cells cultured on synthetic EMC in neutrophil-conditioned media treated with vehicle or Alvelestat. **(B)** Quantification of area occupied by laminin after culture in neutrophil-conditioned media (*n* = 3 technical replicates per condition). Represented as represented as percent of total analyzed area. Derived from the staining shown in A. **(C)** PyMT+ cells cultured on fluorescently-labeled collagen IV ECM in neutrophil conditioned media treated with vehicle or Alvelestat. Collagen IV would fluoresce green when degraded. **(D)** Quantification of degraded collagen IV as a percentage of total cells after culture in neutrophil-conditioned media (*n* = 6 technical replicates per condition). Derived from the staining shown in C. **(E)** Staining for laminin and DAPI on PyMT+ cells cultured on synthetic EMC in control media supplemented with PBS or supplemented with recombinant NE (rNE), recombinant cathepsin G, recombinant proteinase 3 (PR3) or recombinant Chi3l1, or cultured in in neutrophil conditioned media. **(F)** PyMT+ cells cultured on fluorescently-labeled collagen IV ECM in control media supplemented with PBS or supplemented with rNE, recombinant cathepsin G, rPR3 or rChi3l1, or cultured in in neutrophil conditioned media. **(G)** Quantification of area occupied by laminin after culture in the presence of recombinant proteins (*n* = 6 technical replicates per condition). Represented as represented as percent of total analyzed area. Derived from the staining shown in E. **(H)** Quantification of degraded collagen IV as a percentage of total cells after culture in presence of recombinant proteins (*n* = 6 technical replicates per condition). Derived from the staining shown in F. **(I)** Representative images of invading PyMT+ cells when cultured on a layer of synthetic ECM with control media, neutrophil conditioned media treated with vehicle, or Alvelestat stained with crystal violet. **(J)** Quantification of total invading PyMT+ cancer cells cultured on a layer of synthetic ECM with control media, neutrophil conditioned media treated with vehicle or Alvelestat. *n* = 4 technical replicates for each condition. Derived from the staining shown in I. **(K)** Quantification of the percentage of CK14+ cleaved caspase 3+ cells cells divided by total CK14+ cells in IgG2a (*n* = 6) and anti-Ly6G (*n* = 8) treated Chi3l1 OE MIC mammary glands at 2 weeks post induction. Derived from the staining shown in N. **(L)** Quantification of the percentage of Pan-ck+ cleaved caspase 3+ cells cells within total Pan-ck+ cells in IgG2a (*n* = 6) and anti-Ly6G (*n* = 8) treated Chi3l1 OE MIC mammary glands at 2 weeks post induction. Derived from the staining shown in N. **(M)** Quantification of the percentage of CK14+ p-FAK+ cells cells divided by total CK14+ cells in IgG2a (*n* = 6) and anti-Ly6G (*n* = 8) treated Chi3l1 OE MIC mammary glands at 2 weeks post induction. Derived from the staining shown in O. **(N)** Fluorescent IHC staining of mammary tissue from Chi3l1 OE MIC mice treated with anti-Ly6G or IgG2a isotype control for 2 weeks, using antibodies against CK14, cleaved caspase 3 and pan-ck, counterstained with DAPI. **(O)** Fluorescent IHC staining of mammary tissue from Chi3l1 OE MIC mice treated with anti-Ly6G or IgG2a isotype control for 2 weeks, using antibodies against CK14, p-FAK and Pan-ck, counterstained with DAPI. Ns: not significant, *p<0.05, ***p<0.001 and ****p<0.0001 calculated by Student's T-test (For B,D), One-way anova with Dunnet's post hoc test (for F,H), One-way anova with Tuckey's post hoc test (For J) or using Mann-Whitney non-parametric test (For K-M). Data are presented as mean ± SEM. Each data point is an independent biological sample, *N* represents the number of independent biological replicates. Analysis was performed by the HALO (Indica lab) software. Quantification was performed on whole-slide scans of the entire mammary gland section.

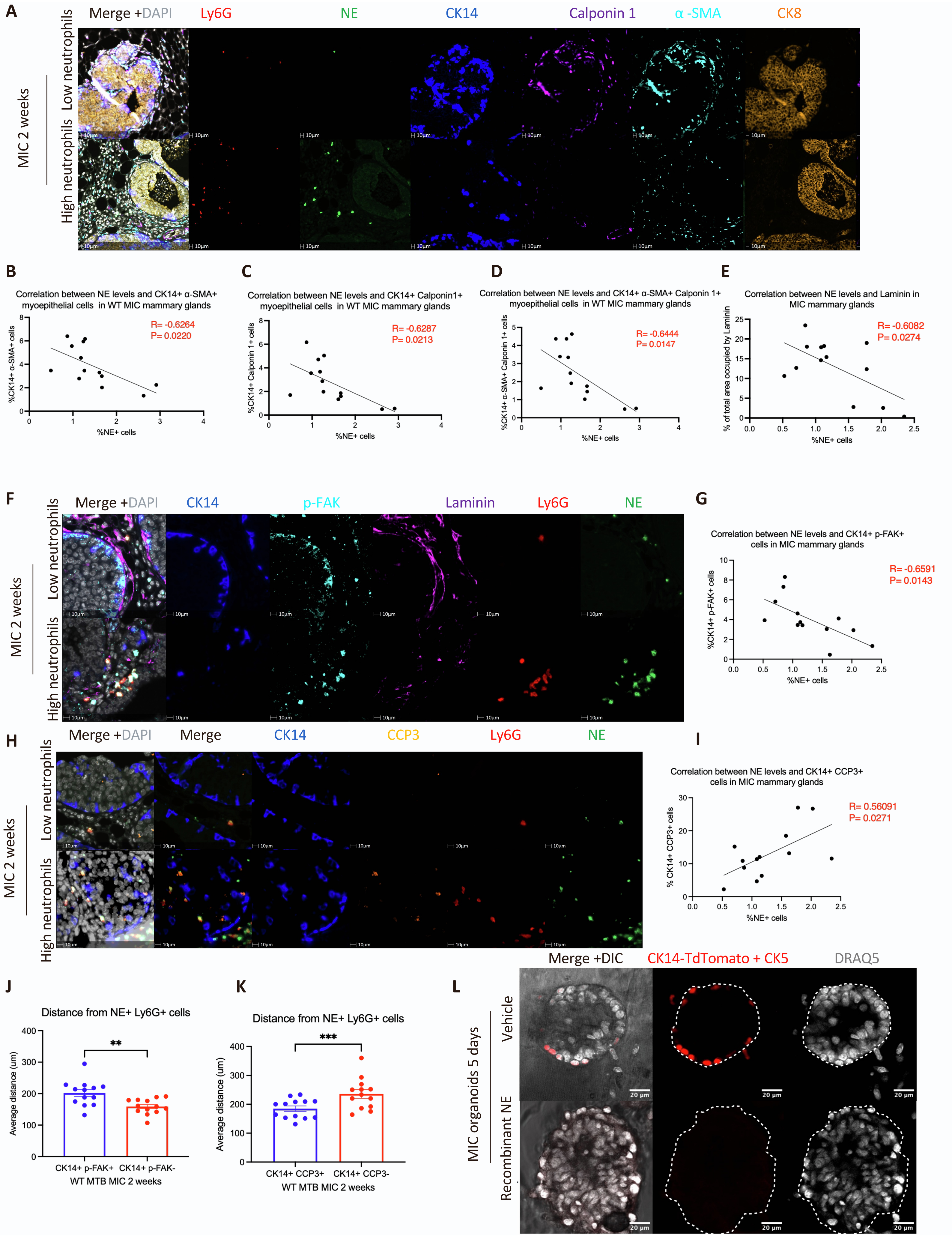

A
Merge +DAPI
Ly6G
NE
CK14
Calponin 1
α -SMA
CK8
MIC 2 weeks
Low neutrophils
High neutrophils
10μm
B
Correlation between NE levels and CK14+ α-SMA+ myoepithelial cells in WT MIC mammary glands
R= -0.6264
P= 0.0220
%CK14+ α-SMA+ cells
%NE+ cells
C
Correlation between NE levels and CK14+ Calponin1+ myoepithelial cells in WT MIC mammary glands
R= -0.6287
P= 0.0213
%CK14+ Calponin 1+ cells
%NE+ cells
D
Correlation between NE levels and CK14+ α-SMA+ Calponin 1+ myoepithelial cells in WT MIC mammary glands
R= -0.6444
P= 0.0147
%CK14+ α-SMA+ Calponin 1+ cells
%NE+ cells
E
Correlation between NE levels and Laminin in MIC mammary glands
R= -0.6082
P= 0.0274
% of total area occupied by Laminin
%NE+ cells
F
Merge +DAPI
CK14
p-FAK
Laminin
Ly6G
NE
MIC 2 weeks
Low neutrophils
High neutrophils
G
Correlation between NE levels and CK14+ p-FAK+ cells in MIC mammary glands
R= -0.6591
P= 0.0143
%CK14+ p-FAK+ cells
%NE+ cells
H
Merge +DAPI
Merge
CK14
CCP3
Ly6G
NE
MIC 2 weeks
Low neutrophils
High neutrophils
I
Correlation between NE levels and CK14+ CCP3+ cells in MIC mammary glands
R= 0.56091
P= 0.0271
% CK14+ CCP3+ cells
%NE+ cells
J
Distance from NE+ Ly6G+ cells
**
Average distance (um)
CK14+ p-FAK+
CK14+ p-FAK-
WT MTB MIC 2 weeks
K
Distance from NE+ Ly6G+ cells
***
Average distance (um)
CK14+ CCP3+
CK14+ CCP3-
WT MTB MIC 2 weeks
L
Merge +DIC
CK14-TdTomato + CK5
DRAQ5
MIC organoids 5 days
Vehicle
Recombinant NE
20 μm

Supplementary Figure 12: Spatial analysis of NE and myoepithelial markers in WT MIC mammary glands. Related to Figure 6.
(A) Fluorescent IHC staining of mammary tissue from WT MIC mice at 2 weeks post induction, using antibodies against CK14, Calponin 1, α - SMA, Ly6G, NE and CK8 counterstained with DAPI. **(B-D)** Pearson correlation between NE+ cells and the total CK14+ calponin 1, CK14+ α-SMA+ and CK14+ calponin 1+ α-SMA+ cells normalized to total analyzed area in WT MIC mammary glands at 2 weeks post induction (*n* = 13). Derived from the staining shown in A. **(E)** Pearson correlation between NE+ cells and the the area occupied by laminin in WT MIC mammary glands at 2 weeks post induction (*n* = 13). Derived from the staining shown in F. **(F)** Fluorescent IHC staining of mammary tissue from WT MIC mice at 2 weeks post induction, using antibodies against CK14, p-FAK, Laminin, Ly6G and NE counterstained with DAPI. **(G)** Pearson correlation between NE+ cells and the the area occupied by laminin or total CK14 p-Fak+ cells represented as percentage of total cells in WT MIC mammary glands at 2 weeks post induction (*n* = 13). Derived from the staining shown in F. **(H)** Fluorescent IHC staining of mammary tissue from WT MIC mice at 2 weeks post induction, using antibodies against CK14, Cleaved caspase 3, Ly6G and NE counterstained with DAPI. **(I)** Pearson correlation between NE+ cells and total CK14 CCP3+ cells represented as percentage of total cells (*n* = 13). Derived from the staining shown in H. **(J)** Quantification of the average distance of Ly6G+ NE+ cells to CK14+ p-FAK+ cells or CK14+ p-FAK- cells in WT MIC mice at 2 weeks post induction (*n* = 13). Derived from the staining shown in F. **(K)** Quantification of the average distance of Ly6G+ NE+ cells to CK14+ CCP3+ cells or CK14+ CCP3- cells in WT MIC mice at 2 weeks post induction (*n* = 13). Derived from the staining shown in H. **(L)** Fluorescent staining of organoids grown from MIC CK14-Tdtomato mammary glands induced in culture for 5 days and treated with vehicle or recombinant neutrophil elastase for 24 hours. In red, CK5 immunostaining and endogenous K14 fluorescence are visible and in grey DRAQ5 marks nuclei. White dotted line used to outline organoid seen by DIC. **p<0.01 and ****p<0.0001 calculated by paired Student's T-test. Data are presented as mean ± SEM. Pearson correlation was performed using two-tailed t test. Each data point is an independent biological sample, *N* represents the number of independent biological replicates. Analysis was performed by the HALO (Indica lab) software. Quantification was performed on whole-slide scans of the entire mammary gland section.

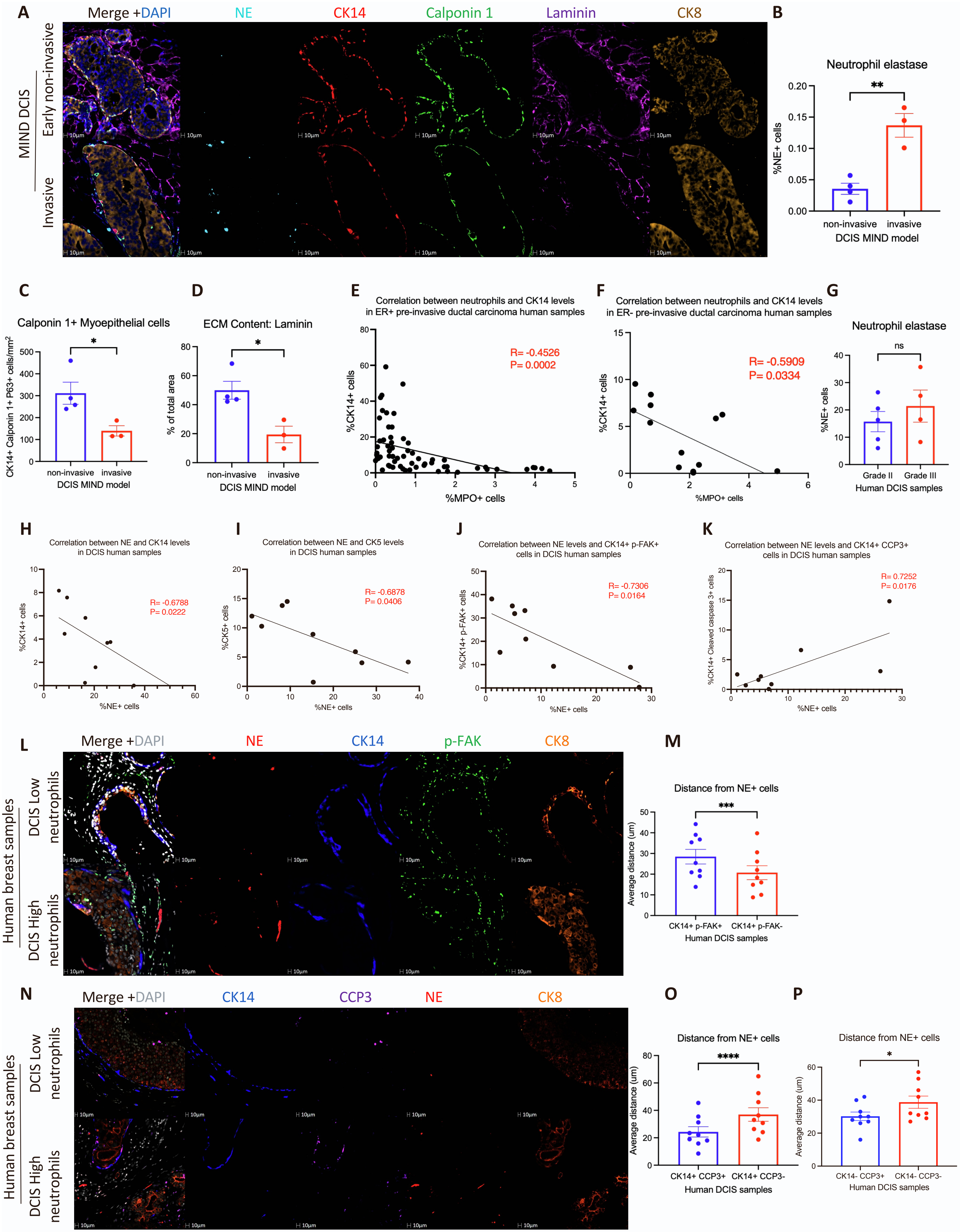
A
Merge +DAPI
NE
CK14
Calponin 1
Laminin
CK8
MIND DCIS
Early non-invasive
Invasive
10µm
B
Neutrophil elastase
%NE+ cells
non-invasive
invasive
DCIS MIND model
**
C
Calponin 1+ Myoepithelial cells
CK14+ Calponin 1+ P63+ cells/mm2
non-invasive
invasive
DCIS MIND model
*
D
ECM Content: Laminin
% of total area
non-invasive
invasive
DCIS MIND model
*
E
Correlation between neutrophils and CK14 levels
in ER+ pre-invasive ductal carcinoma human samples
%CK14+ cells
%MPO+ cells
R= -0.4526
P= 0.0002
F
Correlation between neutrophils and CK14 levels
in ER- pre-invasive ductal carcinoma human samples
%CK14+ cells
%MPO+ cells
R= -0.5909
P= 0.0334
G
Neutrophil elastase
%NE+ cells
ns
Grade II
Grade III
Human DCIS samples
H
Correlation between NE and CK14 levels
in DCIS human samples
%CK14+ cells
%NE+ cells
R= -0.6788
P= 0.0222
I
Correlation between NE and CK5 levels
in DCIS human samples
%CK5+ cells
%NE+ cells
R= -0.6878
P= 0.0406
J
Correlation between NE levels and CK14+ p-FAK+
cells in DCIS human samples
%CK14+ p-FAK+ cells
%NE+ cells
R= -0.7306
P= 0.0164
K
Correlation between NE levels and CK14+ CCP3+
cells in DCIS human samples
%CK14+ Cleaved caspase 3+ cells
%NE+ cells
R= 0.7252
P= 0.0176
L
Merge +DAPI
NE
CK14
p-FAK
CK8
Human breast samples
DCIS Low neutrophils
DCIS High neutrophils
M
Distance from NE+ cells
Average distance (um)
***
CK14+ p-FAK+
CK14+ p-FAK-
Human DCIS samples
N
Merge +DAPI
CK14
CCP3
NE
CK8
Human breast samples
DCIS Low neutrophils
DCIS High neutrophils
O
Distance from NE+ cells
Average distance (um)
****
CK14+ CCP3+
CK14+ CCP3-
Human DCIS samples
P
Distance from NE+ cells
Average distance (um)
*
CK14- CCP3+
CK14- CCP3-
Human DCIS samples

Supplementary Figure 13: Spatial analysis of NE and myoepithelial markers in human samples samples. Related to Figure 6.
(A) Fluorescent IHC staining of early DCIS and invasive human MIND DCIS using antibodies against NE, CK14, Calponin 1, laminin and CK8, counterstained with DAPI. **(B)** Quantification of total NE+ cells as a percentage of total cells in early DCIS ($n = 4$), and invasive ($n = 3$) MIND samples. Derived from the staining shown in in A. **(C)** Quantification of total CK14+ calponin 1+ cells normalized to total analyzed area in early DCIS ($n = 4$), and invasive ($n = 3$) MIND samples. Derived from the staining shown in A. **(D)** Quantification of area occupied by laminin early DCIS ($n = 4$), and invasive ($n = 3$) MIND samples. Represented as percent of mammary gland area. Derived from the staining shown in I A. **(E, F)** Pearson correlation between neutrophils (MPO+ cells) and the total CK14+ cells in human ER+ ($n = 62$) or ER- ($n = 13$) DCIS samples. Both represented as a percentage of total cells. Derived from the staining shown in in Fig. 6A. **(G)** Quantification of total NE+ cells as a percentage of total cells in grade II ($n = 5$) or grade III ($n = 4$) human DCIS samples. Derived from the staining shown in in Fig. 6E. **(H-K)** Pearson correlation between NE+ cells and total CK14+ cells, total CK5+ cells, CK14+ p-FAK+ cells or CK14+ CCP3+ cells in human DCIS samples ($n = 9$). All represented as a percentage of total cells. Derived from the staining shown in in 6E and S12L.**(L)** Fluorescent IHC staining of human DCIS samples using antibodies against NE, CK14, p-FAK, and CK8, counterstained with DAPI. Micrograph represents areas of high NE and low NE from different DCIS patient samples. **(M)** Quantification of the average distance of NE+ cells to CK14+ p-FAK+ cells or CK14+ p-FAK- cells in human DCIS samples ($n = 9$). Derived from the staining shown in in L. **(N)** Fluorescent IHC staining of human DCIS samples using antibodies against NE, CK14, CCP3, and CK8, counterstained with DAPI. Micrograph represents areas of high NE and low NE from different DCIS patient samples **(O)** Quantification of the average distance of NE+ cells to CK14+ CCP3+ cells or CK14+ CCP3- cells in human DCIS samples ($n = 9$). Derived from the staining shown in N. **(P)** Quantification of the average distance of NE+ cells to CK14- CCP3+ cells or CK14- CCP3- cells in human DCIS samples ($n = 9$). Derived from the staining shown in N. Ns: not significant, $^{*}p<0.05$, $^{**}p<0.01$, $^{***}p<0.001$ and $^{****}p<0.0001$ calculated by Student's T-test. Paired t-test was used for L and N. Pearson correlation was performed using two-tailed t test. Data are presented as mean ± SEM. Each data point is an independent biological sample, *N* represents the number of independent biological replicates. Analysis was performed by the HALO (Indica lab) software. Quantification was performed on whole-slide scans of the entire mammary gland section.

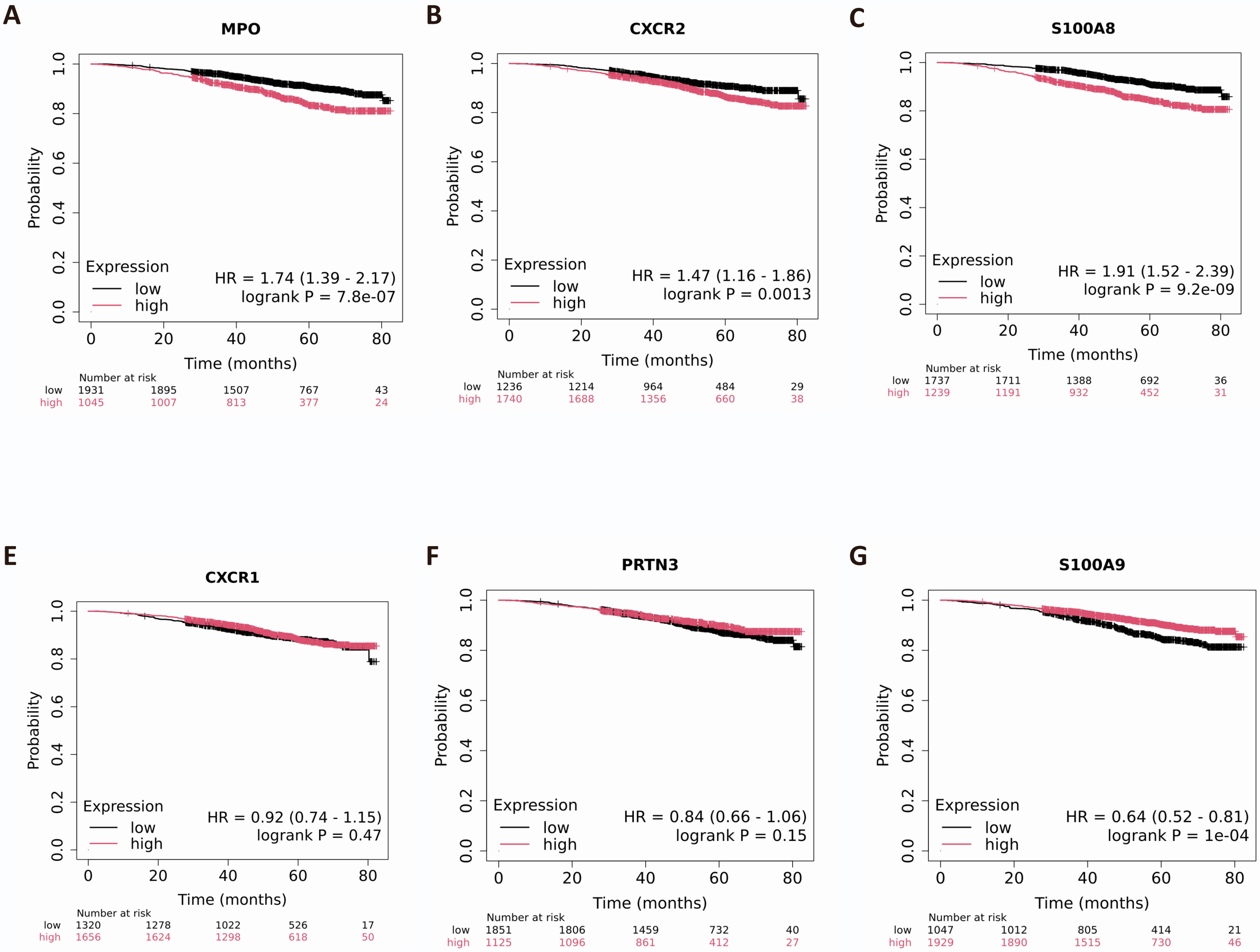
A
MPO
Probability
Expression
low
high
HR = 1.74 (1.39 - 2.17)
logrank P = 7.8e-07
Time (months)
Number at risk
low 1931 1895 1507 767 43
high 1045 1007 813 377 24
B
CXCR2
Probability
Expression
low
high
HR = 1.47 (1.16 - 1.86)
logrank P = 0.0013
Time (months)
Number at risk
low 1236 1214 964 484 29
high 1740 1688 1356 660 38
C
S100A8
Probability
Expression
low
high
HR = 1.91 (1.52 - 2.39)
logrank P = 9.2e-09
Time (months)
Number at risk
low 1737 1711 1388 692 36
high 1239 1191 932 452 31
E
CXCR1
Probability
Expression
low
high
HR = 0.92 (0.74 - 1.15)
logrank P = 0.47
Time (months)
Number at risk
low 1320 1278 1022 526 17
high 1656 1624 1298 618 50
F
PRTN3
Probability
Expression
low
high
HR = 0.84 (0.66 - 1.06)
logrank P = 0.15
Time (months)
Number at risk
low 1851 1806 1459 732 40
high 1125 1096 861 412 27
G
S100A9
Probability
Expression
low
high
HR = 0.64 (0.52 - 0.81)
logrank P = 1e-04
Time (months)
Number at risk
low 1047 1012 805 414 21
high 1929 1890 1515 730 46

Supplementary Figure 14: Clinical correlations of various neutrophil markers in breast cancer. Related to Figure 6. (A-C) Elevated levels of MPO, CXCR2 and S100A8 are associated with decreased overall survival in breast cancer patients of all subtypes at 5 years post diagnosis (*n* = 2976). **(E-G)** Elevated levels of CXCR1, PRTN3 and S100A9 are not associated with decreased overall survival in breast cancer patients of all subtypes at 5 years post diagnosis (*n* = 2976). Analyzed with log-rank test, p-value presented in the figure.

Variable	Non-metastatic (n= 21)	Metastatic (n= 12)
**Average Age**	70	68.4
**ER positive**	14 (66%)	7 (58%)
**PR positive**	11 (52%)	6 (50%)
**HER2 positive**	19 (90%)	11(92%)
**Grade II**	9 (43%)	6 (50%)
**Grade III**	12 (57%)	6 (50%)
**Tumor size**	20.5 mm	22.8mm

Supplementary Table 1: Clinicopathological characteristics of the breast cancer cohort used for metastatic versus non-metastatic analyses. Related to figure 6.
