# Supplementary material for: Neutrophil elastase-mediated myoepithelial loss is a therapeutic vulnerability in ductal carcinoma in situ progression
Source: Cell Rep Med. 2026 Sep 11;7(10):103050. doi: 10.1016/j.xcrm.2026.103050 (PMC13710378; doi:10.1016/j.xcrm.2026.103050)
Supplement: Supplementary file 2 [file mmc3.pdf]

Cell Reports
Medicine

Article

# Neutrophil elastase-mediated myoepithelial loss is a therapeutic vulnerability in ductal carcinoma *in situ* progression

## Graphical abstract

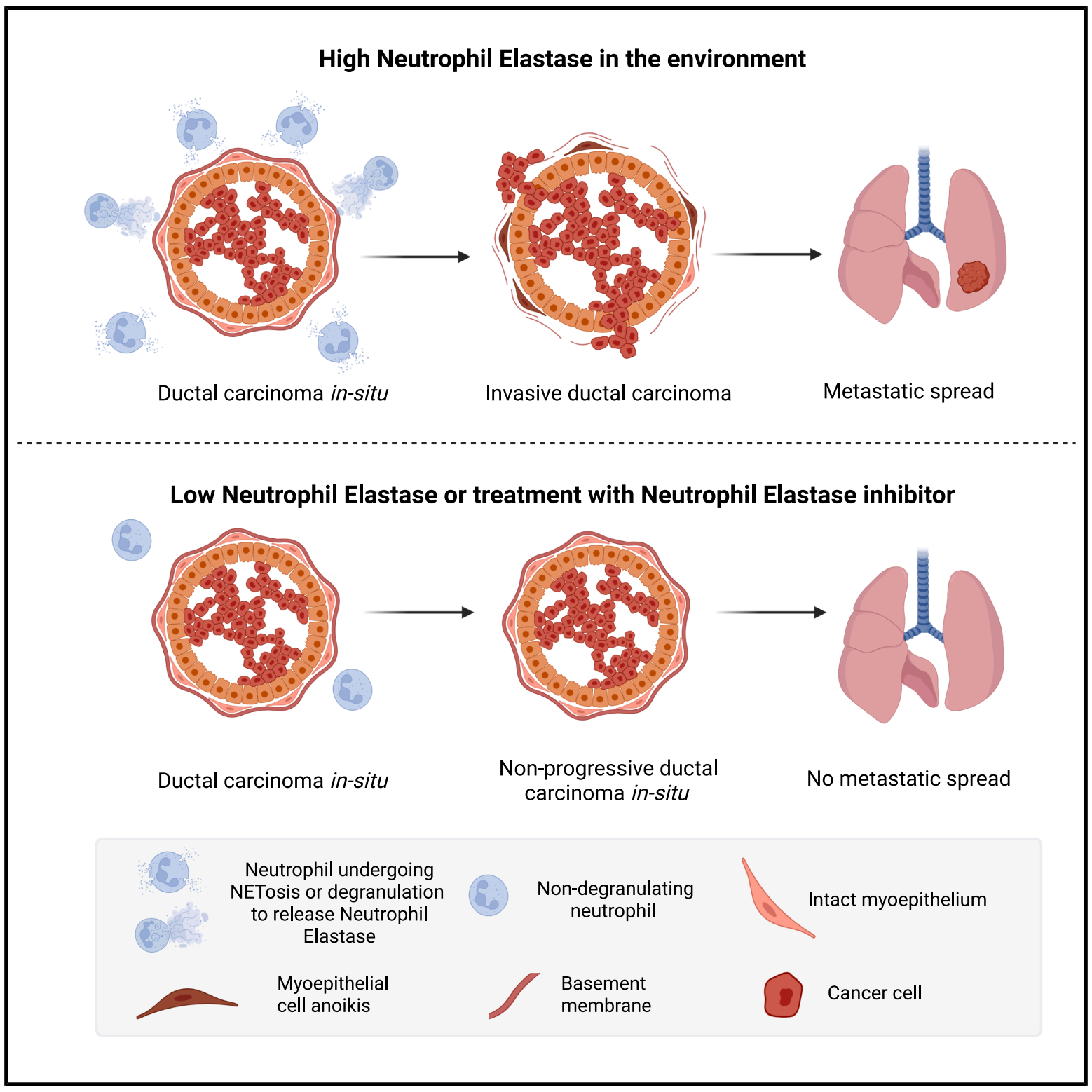


## Highlights

- Neutrophils drive the transition from ductal carcinoma *in situ* to invasive breast cancer
- Neutrophil elastase degrades basement membrane and induces myoepithelial anoikis
- Neutrophil elastase inhibition preserves the myoepithelium and limits metastasis
- Human ductal carcinoma *in situ* links neutrophil elastase to myoepithelial loss

## Authors

Tarek Taifour, Yu Gu, Adéline Massé, ..., Luke McCaffrey, Morag Park, William J. Muller

## Correspondence

william.muller@mcgill.ca

## In brief

Transition from ductal carcinoma *in situ* (DCIS) to invasive breast cancer remains a key step in disease progression that is poorly understood. Taifour et al. show that neutrophil elastase disrupts the myoepithelial barrier to promote invasive progression, identifying neutrophil elastase as a potential therapeutic target for high-risk DCIS.

Taifour et al., 2026, Cell Reports Medicine *7*, 103050
October 20, 2026 © 2026 The Authors. Published by Elsevier Inc.
https://doi.org/10.1016/j.xcrm.2026.103050

CellPress

Cell Reports Medicine

CellPress
OPEN ACCESS

Article

# Neutrophil elastase-mediated myoepithelial loss is a therapeutic vulnerability in ductal carcinoma *in situ* progression

Tarek Taifour,[1,2] Yu Gu,[2,3] Adéline Massé,[2,3] Xiwei Meng,[2,3] Clarisse Dane Echavez,[2,3] Dongmei Zuo,[2] Virginie Sanguin-Gendreau,[2] Anne Blouin,[2] Adda-Lee Graham-Paquin,[2,3] Miranda Addie,[2,4] Emilie Solymoss,[1,2] Bin Xiao,[2] Hailey Proud,[2,3] Sherif Samer Attalla,[2,3] Alice Jisoo Nam,[2,3] Vasilios Papavasiliou,[2] Hellen Kuasne,[2] Peter M. Siegel,[1,2,3] Khandan Keyomarsi,[5] Luke McCaffrey,[1,2,3] Morag Park,[1,2,3] and William J. Muller[1,2,3,6,*]

[1]McGill University, Division of Experimental Medicine, Department of Medicine, Faculty of Medicine, Montreal, QC H4A 3J1, Canada
[2]Rosalind and Morris Goodman Cancer Institute, Montreal, QC H3A 1A3, Canada
[3]McGill University, Department of Biochemistry, Faculty of Medicine, Montreal, QC H3A 1A3, Canada
[4]Department of Surgery, Division of Surgical and Interventional Sciences, McGill University, Montreal, QC H3G 1A4, Canada
[5]Department of Experimental Radiation Oncology, University of Texas MD Anderson Cancer Center, Houston, TX 77030, USA
[6]Lead contact
*Correspondence: william.muller@mcgill.ca
https://doi.org/10.1016/j.xcrm.2026.103050

## SUMMARY

Progression from ductal carcinoma *in situ* (DCIS) to invasive ductal carcinoma (IDC) marks a critical step in breast cancer progression, yet the mechanisms governing this transition remain poorly defined. Here, we identify neutrophils as a key immunological driver of DCIS progression. Across two independent breast cancer mouse models, neutrophil depletion or genetic deletion of their tumor-derived chemoattractant chitinase-3 like 1 (Chi3l1) preserves the myoepithelial barrier, halts DCIS-to-IDC transition, and suppresses lung metastasis. Mechanistically, tumor-infiltrating neutrophils release neutrophil elastase (NE) that degrades the basement membrane extracellular matrix, leading to erosion of the myoepithelial layer by anoikis. Pharmacological NE inhibition restores myoepithelial integrity and blocks DCIS progression and metastasis. Consistently, elevated NE levels in human breast cancer samples are associated with myoepithelial disruption, DCIS progression, and metastasis. Together, these findings identify neutrophils as key mediators of myoepithelial barrier disruption and highlight NE as a potential therapeutic target for high-risk DCIS.

## INTRODUCTION

The transition from ductal carcinoma *in situ* (DCIS) to invasive ductal carcinoma (IDC) marks a pivotal event in breast cancer progression.[1] With widespread screening programs, early detection of non-invasive DCIS now accounts for approximately 20% of newly diagnosed breast cancers.[1,2] Approximately half of DCIS lesions progress to IDC, yet reliable biomarkers to identify high-risk lesions remain lacking.[1,2] DCIS is distinguished from IDC by an intact myoepithelial layer that functions as a physical and biochemical barrier to invasion.[3] Disruption of this layer is a prerequisite for invasive progression, yet the mechanisms governing myoepithelial breakdown remain poorly understood.[3] Understanding the mechanisms governing myoepithelial disruption may improve risk stratification and therapeutic intervention for early breast cancer.

Genomic and transcriptomic studies indicate that DCIS and matched IDC share most genetic alterations, suggesting that invasive progression may depend more on changes in the tumor microenvironment than on additional tumor-intrinsic mutations.[4–6] Thus, alterations in the tumor immune microenvironment (TIME) have emerged as potential drivers of DCIS progression.[7–12] Among immune components, neutrophils are increasingly recognized as critical mediators of immune suppression, metastatic progression, and poor patient outcomes across multiple cancer types.[13–17] Importantly, neutrophils release a diverse repertoire of granule-associated proteases capable of degrading extracellular matrix (ECM) and basement membrane components, positioning them as attractive candidates to regulate the integrity of the myoepithelial barrier.[18,19] Elevated neutrophil infiltration has been observed in DCIS.[20] However, whether neutrophils directly contribute to myoepithelial layer disruption and invasive transition during DCIS progression remains unknown.

In this study, we identify neutrophil-derived neutrophil elastase (NE) as a critical mediator of myoepithelial layer disruption and DCIS progression. Neutrophil depletion as well as genetic or pharmacological inhibition of NE across multiple breast cancer genetically engineered mouse models (GEMMs) preserved the myoepithelial barrier, blocked DCIS progression, and reduced lung metastases. Consistently, human DCIS lesions with elevated neutrophil infiltration or NE levels exhibited


© 2026 The Authors. Published by Elsevier Inc.
This is an open access article under the CC BY license (http://creativecommons.org/licenses/by/4.0/).

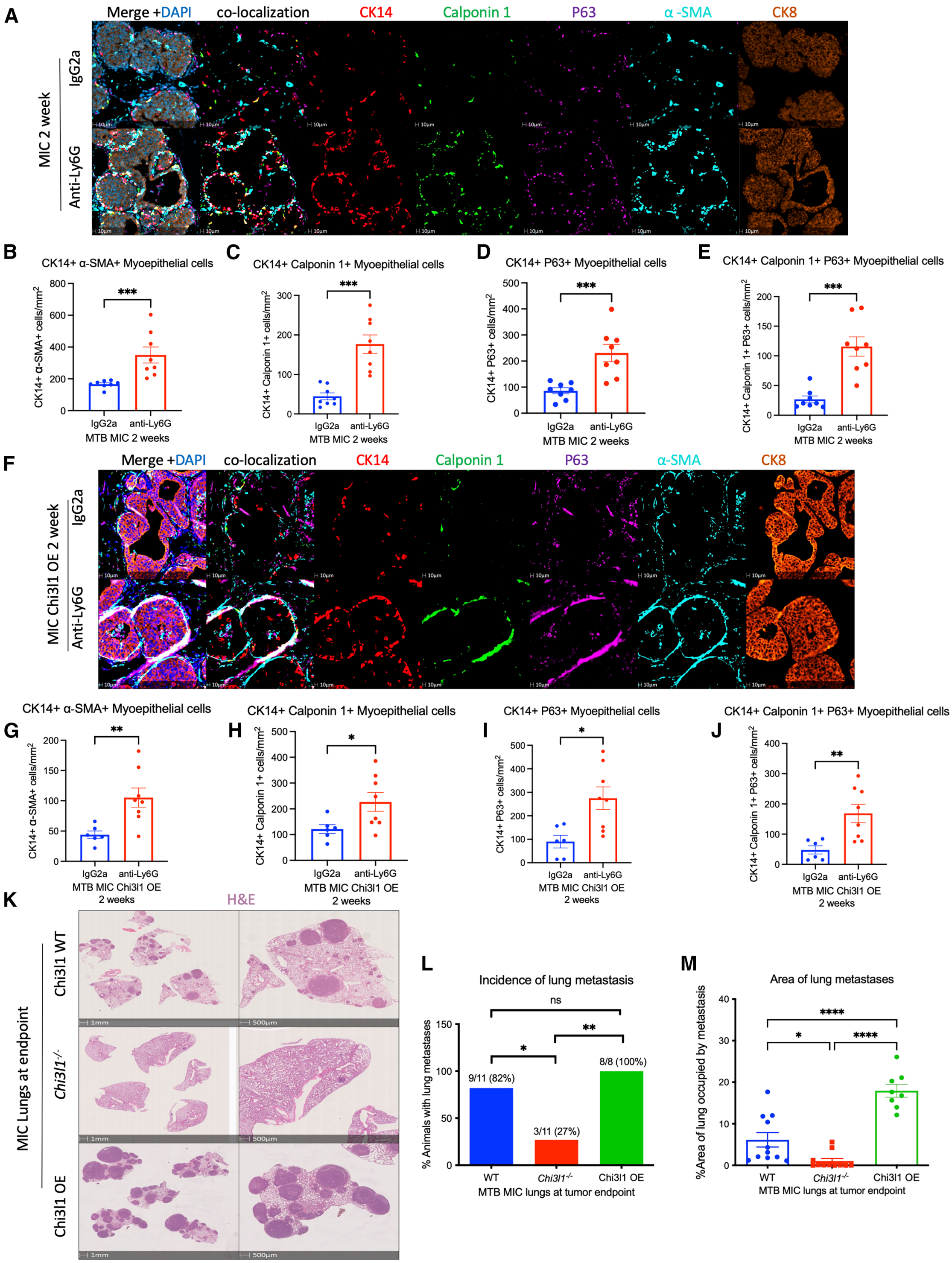


**Figure 1. Neutrophils promote DCIS progression**

(A) Fluorescent immunohistochemistry (IHC) staining of mammary tissue from WT MIC mice treated with anti-Ly6G or IgG2a isotype control for 2 weeks, using antibodies against CK14, α-SMA, calponin 1, P63, and CK8, counterstained with DAPI.

(B–E) Quantification of total CK14$^+$ α-SMA$^+$ (Cohen's d = −1.8016), CK14$^+$ calponin 1$^+$ (Cohen's d = −2.6470), CK14$^+$ P63$^+$ (Cohen's d = −2.0707), and CK14$^+$ calponin 1$^+$ P63$^+$ (Cohen's d = −2.5783) cells normalized to total analyzed area in IgG2a ($n$ = 8) and anti-Ly6G ($n$ = 8)-treated WT MIC mammary glands at 2 weeks post-induction. Derived from the staining shown in (A).

*(legend continued on next page)*

pronounced myoepithelial disruption. Mechanistically, NE released during neutrophil degranulation or NETosis degraded basement membrane ECM, inducing myoepithelial anoikis. Together, these findings establish neutrophils and NE as key drivers of myoepithelial layer loss and DCIS progression.

## RESULTS

### Neutrophils promote myoepithelial layer disruption

To investigate whether neutrophils contribute to myoepithelial layer disruption during breast cancer progression, we utilized a doxycycline-inducible GEMM co-expressing the oncogenic polyomavirus middle T antigen (PyMT) and Cre recombinase (herein referred to as MIC).[21] This model recapitulates the stepwise progression of luminal-B breast cancer, developing mammary hyperplasia within 1 week of induction, DCIS-like lesions by 2 weeks, and IDCs by 5–6 weeks, which subsequently metastasize to the lungs.[21–23] Using neutrophil depletion via an anti-Ly6G (lymphocyte antigen 6 complex locus G6D) antibody previously established and validated in this model,[24,25] we assessed myoepithelial integrity during early tumor progression. As described in these prior studies, 8-week-old MIC mice were treated with anti-Ly6G antibody starting at doxycycline induction and continuing for 2 weeks, which allowed the assessment of neutrophil function at the DCIS stage of disease progression.

Staining for various myoepithelial markers, including cytokeratin 14 (CK14), cytokeratin 5 (CK5), alpha-smooth muscle actin (α-SMA), calponin 1, and P63, revealed significant retention of the myoepithelial layer in anti-Ly6G-treated glands compared with IgG2a-treated controls (Figures 1A–1E; Figures S1A and S1B). Ductal lesions in neutrophil-depleted glands exhibited a continuous and structurally intact myoepithelial layer, whereas control lesions showed marked fragmentation and loss of myoepithelial marker expression (Figures 1A–1E; Figures S1A and S1B). Importantly, neutrophil depletion had no impact on cancer cell proliferation, as assessed through staining for proliferation markers Ki67 and proliferating cell nuclear antigen (PCNA) (Figures S1C–S1E), indicating that preservation of the myoepithelium was not driven by differences in cancer cell proliferation.

To validate these findings in a second independent GEMM representing a distinct breast cancer subtype, we depleted neutrophils in the C3(1)-TAg (C3Tag) GEMM (Figures S1F–S1H). This model develops aggressive basal-like mammary tumors driven by SV40 large T-antigen expression.[26,27] Consistent with our findings in the MIC GEMM, neutrophil depletion in the C3Tag GEMM led to a significant retention of myoepithelial content without altering cancer cell proliferation (Figures S1I–S1P). Overall, these findings demonstrate that neutrophils promote myoepithelial barrier disruption during DCIS progression in luminal-B and basal breast cancer GEMMs.

### Targeting neutrophil chemoattractants preserves myoepithelial integrity

We next asked whether tumor-intrinsic regulation of neutrophil recruitment similarly influences myoepithelial integrity. We previously demonstrated that the tumor-derived cytokine chitinase-3 like 1 (Chi3l1) promotes neutrophil recruitment to the TIME.[24,25] To assess whether Chi3l1-dependent neutrophil accumulation contributes to myoepithelial layer disruption, we crossed Chi3l1-deficient (*Chi3l1*$^{-/-}$) or mammary epithelial-specific Chi3l1-overexpressing (Chi3l1 OE) mice with the MIC model (Figures S2A–S2C). After tumor onset, these models exhibited no differences in disease-bearing survival or tumor volume outgrowth (Figures S2D and S2E).

Staining for neutrophil markers Ly6G, myeloperoxidase (MPO), and NE validated our prior findings. Chi3l1-deficient lesions exhibited reduced neutrophil numbers, whereas Chi3l1 OE glands exhibited a marked increase in neutrophil content relative to wild-type (WT) controls (Figures S2F–S2I). Importantly, no differences in neutrophil numbers were observed in age-matched non-MIC WT, Chi3l1-deficient, or Chi3l1 OE mammary glands (Figures S2J–S2L), indicating that differences in neutrophil levels in these models occur specifically in the context of oncogene-induced tumorigenesis. Thus, these models provide complementary genetic tools to modulate neutrophil levels and investigate their role in DCIS progression. Correspondingly, Chi3l1-deficient MIC lesions demonstrated robust retention of myoepithelial markers, while Chi3l1 OE MIC lesions displayed substantial loss of myoepithelial marker expression and structural integrity compared to controls (Figures S3A–S3H). Analysis of proliferation markers revealed no significant differences between the three groups (Figures S3I–S3K). To determine whether loss of the myoepithelial layer observed in Chi3l1 OE MIC glands was neutrophil dependent, we assessed myoepithelial content in Chi3l1 OE mammary glands following anti-Ly6G treatment as validated in our earlier study.[25] Anti-Ly6G treatment restored myoepithelial layer continuity in Chi3l1 OE MIC glands without impacting cancer cell proliferation, consistent with observations in WT MIC mice (Figures 1F–1J; Figures S3L, S3M, and S4A–S4C).

(F) Fluorescent IHC staining of mammary tissue from Chi3l1 OE MIC mice treated with anti-Ly6G or IgG2a isotype control for 2 weeks, using antibodies against CK14, α-SMA, calponin 1, P63, and CK8, counterstained with DAPI.

(G–J) Quantification of total CK14$^+$ α-SMA$^+$ (Cohen's d = −1.7167), CK14$^+$ calponin 1$^+$ (Cohen's d = −1.2640), CK14$^+$ P63$^+$ (Cohen's d = −1.6381), and CK14$^+$ calponin 1$^+$ P63$^+$ (Cohen's d = −1.7400) cells normalized to total analyzed area in IgG2a ($n$ = 6) and anti-Ly6G ($n$ = 8)-treated Chi3l1 OE MIC mammary glands at 2 weeks post-induction. Derived from the staining shown in (F).

(K) Representative H&E staining of WT, Chi3l1$^{-/-}$, and Chi3l1 OE MIC lungs at mammary tumor endpoint.

(L) Percentage of WT, Chi3l1$^{-/-}$, and Chi3l1 OE MIC mice with pulmonary metastases.

(M) Quantification of total pulmonary metastatic area in WT ($n$ = 11), Chi3l1$^{-/-}$ ($n$ = 11), and Chi3l1 OE ($n$ = 8) MIC lungs at mammary tumor endpoint (average of 59 days since tumor onset for WT, 57 days for Chi3l1$^{-/-}$, and 60 days for Chi3l1 OE) (Eta squared; η2 = 0.73). Represented as percent of total lung area.

*$p < 0.05$, **$p < 0.01$, ***$p < 0.001$, and ****$p < 0.0001$ calculated by Mann-Whitney non-parametric test (for B–E and G–J), Fisher's exact test (for L), or one-way ANOVA with Tukey's post hoc test (for M). Data are presented as mean ± SEM. Each data point is an independent biological sample, and $N$ represents the number of independent biological replicates. Analysis was performed by the HALO (Indica Labs) software. Quantification was performed on whole-slide scans of the entire mammary gland section. Scale bars: 10 (A and F) and 500 μm (K).

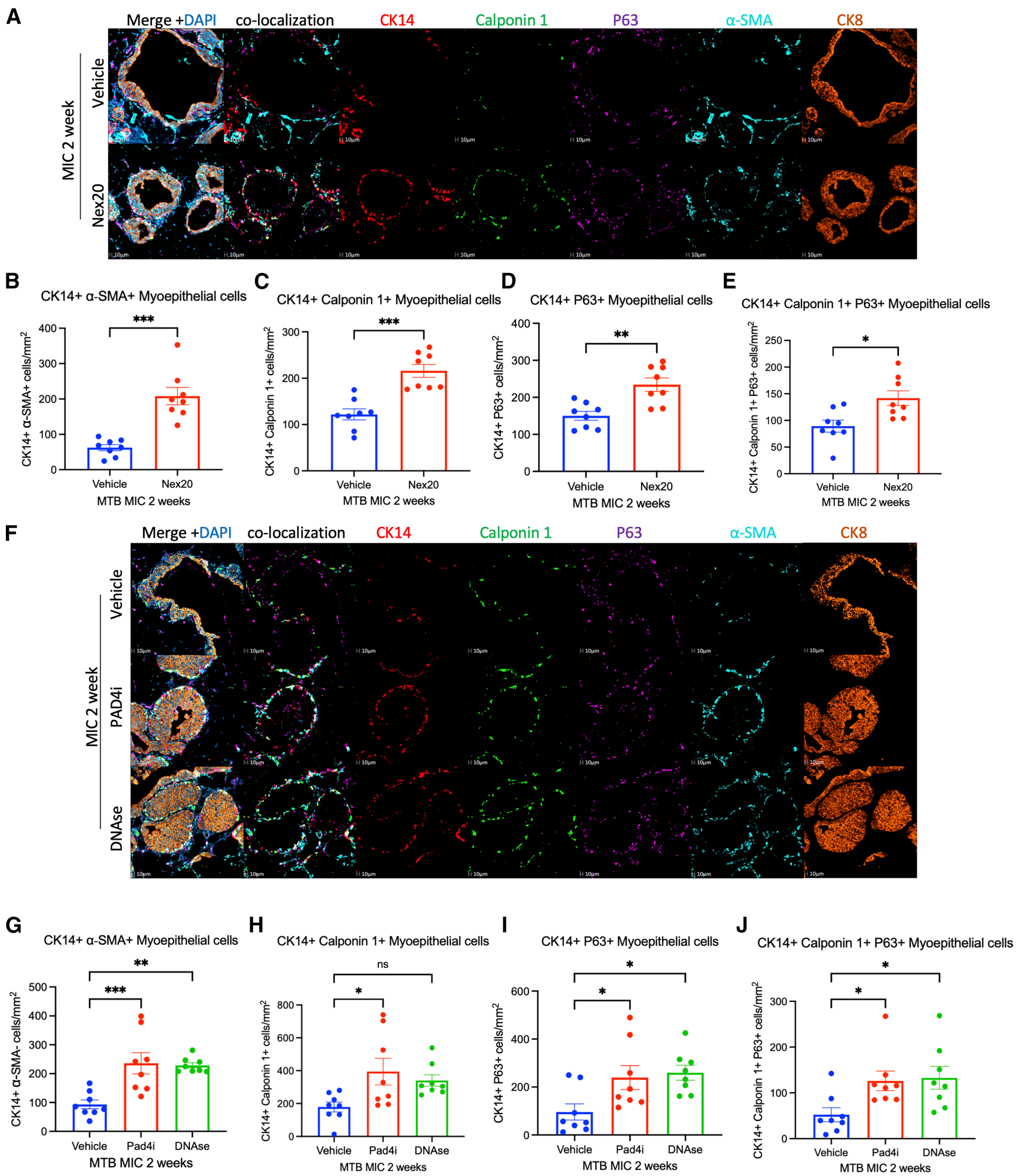


**Figure 2. Targeting neutrophil degranulation or NETosis inhibits myoepithelial layer dissolution**

(A) Fluorescent IHC staining of mammary glands from vehicle or Nex20-treated WT MIC mice at 2 weeks post-induction using antibodies against CK14, α-SMA, calponin 1, P63, and CK8, counterstained with DAPI.

(B–E) Quantification of total $CK14^+$ $\alpha$-$SMA^+$ (Cohen's d = −2.8082), $CK14^+$ calponin $1^+$ (Cohen's d = −2.5726), $CK14^+$ $P63^+$ (Cohen's d = −1.9333), and $CK14^+$ calponin $1^+$ $P63^+$ (Cohen's d = −1.4729) cells normalized to total analyzed area in vehicle ($n = 8$) and Nex20-treated ($n = 8$) MIC mammary glands at 2 weeks post-induction. Derived from the staining shown in (A).

(F) Fluorescent IHC staining of mammary glands from vehicle, Pad4i, or DNase-treated WT MIC mice at 2 weeks post-induction using antibodies against CK14, α-SMA, calponin 1, P63, and CK8, counterstained with DAPI.

(G–J) Quantification of total $CK14^+$ $\alpha$-$SMA^+$ (eta squared: $\eta2 = 0.52$), $CK14^+$ calponin $1^+$ (Eta squared: $\eta2 = 0.29$), $CK14^+$ $P63^+$ (Eta squared; $\eta2 = 0.33$), and $CK14^+$ calponin $1^+$ $P63^+$ (Eta squared; $\eta2 = 0.3$) cells normalized to total analyzed area in vehicle ($n = 8$), Pad4i ($n = 8$), and DNase-treated ($n = 8$) MIC mammary glands at 2 weeks post-induction. Derived from the staining shown in (F).

*(legend continued on next page)*

These phenotypes persisted at tumor endpoint; Chi3l1-deficient tumors retained significantly higher myoepithelial content, while Chi3l1 OE tumors exhibited myoepithelial loss (Figures S4D–S4I), indicating that neutrophil-driven loss of myoepithelium remains consistent throughout tumor progression. Consistent with the protective barrier function of the myoepithelium against tumor invasion and metastasis, Chi3l1-deficient mice developed significantly fewer and smaller lung metastases, whereas Chi3l1 overexpression increased both metastatic incidence and burden compared to WT controls (Figures 1K–1M). Cancer cell proliferation remained unchanged at mammary tumor endpoint (Figures S4J–S4L), indicating that the metastatic phenotype was independent of cancer cell proliferation. Together, these data demonstrate that Chi3l1-mediated neutrophil recruitment promotes myoepithelial barrier disruption and metastatic progression.

To further validate our findings in a basal GEMM, Chi3l1-deficient mice were crossed with the C3Tag GEMM (Figures S5A and S5B).[26,27] Like the MIC model, Chi3l1 ablation in the C3Tag GEMM had no impact on disease-bearing survival, tumor outgrowth, or proliferation (Figures S5C–S5G). Consistent with the role of Chi3l1 in neutrophil recruitment, Chi3l1-deficient C3Tag tumors exhibited significantly reduced neutrophil infiltration compared with Chi3l1-proficient controls (Figures S5H–S5K). Notably, Chi3l1-deficient C3Tag tumors retained robust expression of myoepithelial markers, including α-SMA, calponin 1, and P63, compared to Chi3l1-proficient tumors (Figures S5L–S5P). Because C3Tag tumors exhibit a basal phenotype, CK14 was expressed by both tumor and myoepithelial cells, making it unsuitable for quantifying the myoepithelial compartment in end-stage tumors. These findings demonstrate that neutrophil-associated myoepithelial layer disruption is observed across genetically and molecularly distinct models of breast cancer.

## Neutrophil degranulation and NETosis promote myoepithelial layer disruption

Neutrophils exert effector functions through the release of proteases and granular contents via degranulation and through neutrophil extracellular traps (NETosis). To define which neutrophil function contributes to myoepithelial layer disruption, we selectively inhibited neutrophil degranulation or NETosis in the MIC GEMM and assessed myoepithelial integrity.

To inhibit neutrophil degranulation, MIC mice were treated with Nexinhib20 (Nex20), a selective inhibitor of degranulation.[28] While Nex20 treatment did not impact overall neutrophil levels in the mammary gland (Figures S6A and S6B), it significantly reduced serum MPO and NE levels measured by ELISA (enzyme-linked immunosorbent assay), confirming effective inhibition of neutrophil degranulation (Figures S6C and S6D). Importantly, Nex20 treatment did not impact the levels of citrullinated H3 (CitH3), a selective NETosis marker, confirming the specificity of degranulation blockade (Figures S6E–S6I).

Nex20-treated glands exhibited significant retention of myoepithelial markers and preservation of myoepithelial architecture compared to vehicle-treated controls (Figures 2A–2E). Similar protection of the myoepithelium was observed in Chi3l1 OE MIC mice treated with Nex20 (Figures S6J–S6O), demonstrating that degranulation-dependent myoepithelial disruption occurred even in the context of elevated neutrophil infiltration. Together, these data indicate that neutrophil degranulation is a key contributor to myoepithelial barrier loss.

NETosis represents a second neutrophil effector mechanism implicated in tumor progression.[24,29] We had previously treated WT MIC mice with a peptidyl arginine deiminase 4 inhibitor (Pad4i, GSK484) to block NET formation, or DNase-1 to disrupt pre-existing NETs.[24] In both settings, staining revealed a significant retention of myoepithelial markers compared to vehicle-treated glands (Figures 2F–2J). Collectively, these findings demonstrate that both neutrophil degranulation and NETosis independently contribute to myoepithelial layer disruption.

## NE promotes myoepithelial layer disruption

Because pharmacological inhibition of either neutrophil degranulation or NETosis preserved the myoepithelial layer, we hypothesized that a shared neutrophil-derived effector mediates myoepithelial disruption. NE, a serine protease released during both degranulation and NETosis, is a strong candidate mediator given its ability to remodel the TIME and degrade ECM components.[30–32] Although elevated NE levels are associated with poor breast cancer patient outcomes,[33] its role in DCIS progression has not been defined.

To functionally assess the role of NE in DCIS progression, we treated WT MIC mice with alvelestat (Alv), a selective NE inhibitor.[34] Effective NE inhibition was confirmed by evaluating the levels of NE substrates, thrombospondin-1 (TSP1) and elastin, which are degraded by NE. Indeed, alvelestat-treated mammary glands exhibited significantly increased TSP1 and elastin levels (Figures S7A–S7D). Importantly, alvelestat treatment did not impact overall neutrophil levels or cancer cell proliferation in the mammary gland (Figures S7E–S7I), indicating that its effects were not due to changes in neutrophil recruitment or proliferation.

Furthermore, alvelestat-treated glands exhibited marked retention of myoepithelial markers and preservation of myoepithelial architecture compared to vehicle-treated controls (Figures 3A–3E; Figures S7J and S7K). These findings were also confirmed through fluorescent-activated cell sorting (FACS) where alvelestat treatment had no impact on overall neutrophil levels but significantly increased EPCAM$^{-}$CD49f$^{+}$CK5$^{+}$CK14$^{+}$ myoepithelial cells (Figures S7L–S7O). Similar protection of the myoepithelial layer was observed in Chi3l1 OE MIC mice treated with alvelestat, demonstrating that NE inhibition counteracted myoepithelial disruption even in the setting of elevated neutrophil infiltration (Figures S8A–S8F). To assess whether this phenotype was shared across GEMMs, WT C3Tag mice were treated with alvelestat at

Ns, not significant, *$p < 0.05$, **$p < 0.01$, ***$p < 0.001$, and ****$p < 0.0001$ calculated Mann-Whitney non-parametric test (for B–E) or one-way ANOVA with Dunnet's post hoc test (for G–J). Data are presented as mean ± SEM. Each data point is an independent biological sample, and *N* represents the number of independent biological replicates. Analysis was performed by the HALO (Indica Labs) software. Quantification was performed on whole-slide scans of the entire mammary gland section. Scale bars: 10 μm.

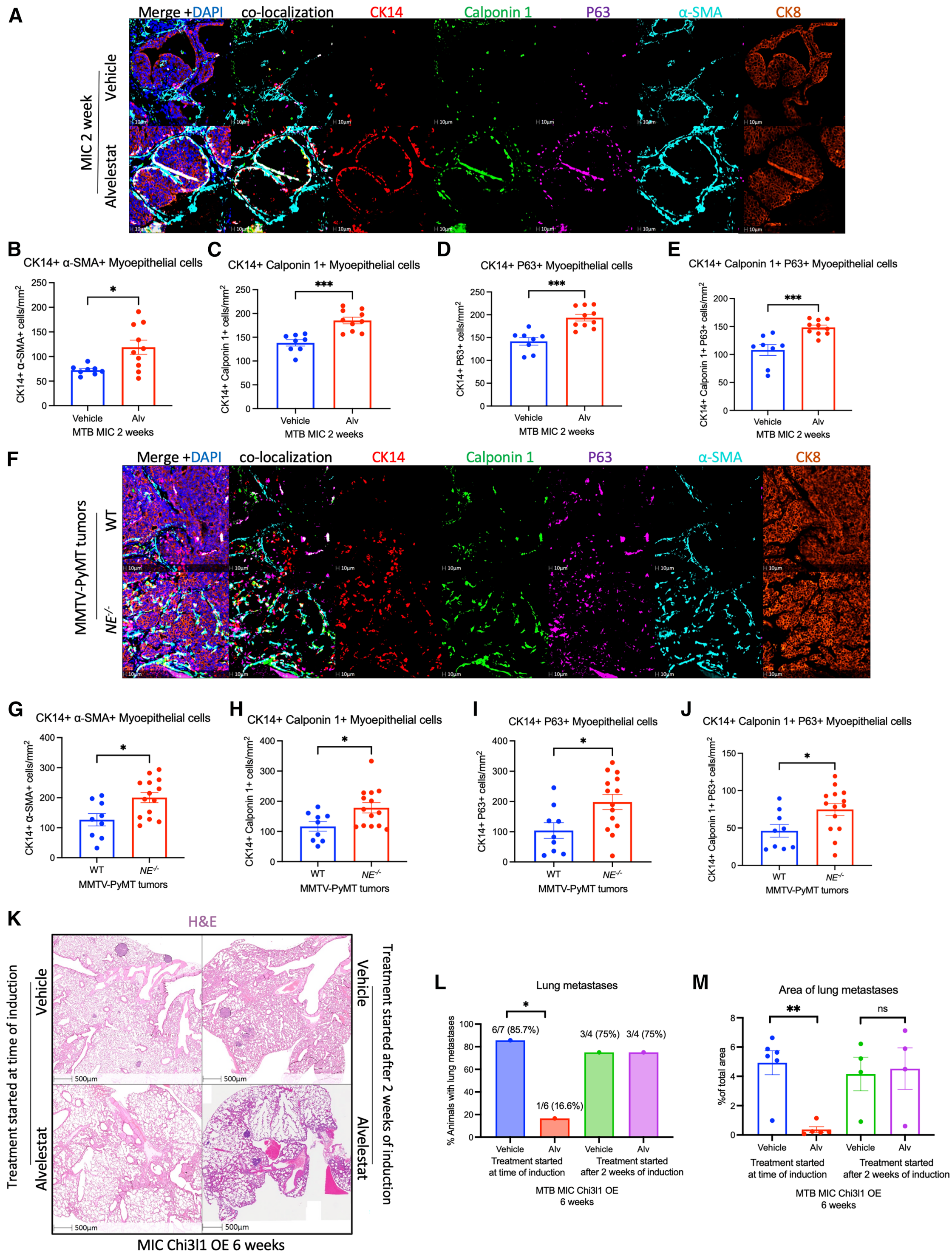


**Figure 3. Targeting NE inhibits DCIS progression**

(A) Fluorescent IHC staining of mammary glands from vehicle or alvelestat-treated WT MIC mice at 2 weeks post-induction using antibodies against CK14, calponin 1, P63 α-SMA, and CK8 counterstained with DAPI.

(B–E) Quantification of total $CK14^{+}$ $\alpha\text{-}SMA^{+}$ (Cohen's d = −1.3403), $CK14^{+}$ calponin $1^{+}$ (Cohen's d = −2.2712), $CK14^{+}$ $P63^{+}$ (Cohen's d = −2.2540), and $CK14^{+}$ calponin $1^{+}$ $P63^{+}$ (Cohen's d = −1.9551) cells normalized to total analyzed area in vehicle (*n* = 8) and alvelestat-treated (*n* = 10) MIC mammary glands at 2 weeks post-induction. Derived from the staining shown in (A).

*(legend continued on next page)*

the DCIS stage. Consistently, alvelestat-treated C3Tag mammary glands exhibited significantly increased myoepithelial content compared with vehicle-treated controls (Figures S8G–S8J).

Complementing our pharmacological studies, we evaluated myoepithelial content in breast tumors derived from NE-deficient (*NE*$^{-/-}$) MMTV-PyMT mice.[35] While NE deficiency does not alter primary tumor onset or growth, NE-deficient mice exhibit a marked reduction in lung metastases.[35] Consistent with our alvelestat-treated mice, staining revealed that NE-deficient tumors retained significantly higher levels of myoepithelial markers, indicating preserved myoepithelial integrity compared to NE-proficient counterparts (Figures 3F–3J). Together, these pharmacologic and genetic approaches identify NE as a critical neutrophil-derived effector driving myoepithelial layer disruption.

We had previously demonstrated that targeting NETosis or neutrophil degranulation inhibits lung metastatic progression.[25] Given the role of the myoepithelium in restraining invasion and metastatic dissemination, we investigated whether targeting NE would similarly suppress metastasis. Thus, we extended alvelestat treatment in Chi3l1 OE MIC mice to 6 weeks, a time point at which these mice develop lung metastases.[25] NE inhibition significantly reduced the incidence and burden of lung metastases without impacting mammary tumor onset (Figures 3K–3M; Figures S9A and S9B). To determine whether this anti-metastatic effect was associated with the preservation of the myoepithelial barrier rather than a direct impact on metastatic outgrowth, we performed a second intervention study in which alvelestat treatment was initiated 2 weeks after doxycycline induction, a stage at which myoepithelial disruption had already occurred (Figure S3) and continued until week 6. In contrast to early intervention, delayed NE inhibition failed to reduce metastatic incidence or burden (Figures 3K–3M; Figure S9A). These findings argue that NE activity contributed to early metastatic dissemination and that NE inhibition is less effective once myoepithelial barrier disruption had already occurred.

Consistent with this interpretation, NE inhibition had no impact on anti-tumor immune responses. Staining revealed no differences in total CD8$^{+}$ cytotoxic T cells, CD4$^{+}$ T helper cells, or T cell activation status between vehicle- and alvelestat-treated glands (Figures S9C–S9H), indicating that the metastatic phenotype was not due to changes in the TIME. To further investigate whether NE contributed to tumor progression independently of its effects on the myoepithelial barrier, we treated tumor-bearing MIC mice with alvelestat from the time of palpable tumor onset until humane endpoint. Under these conditions, where the myoepithelial layer is already disrupted and invasive disease is established, NE inhibition had no effect on primary tumor growth, metastatic incidence, or burden (Figures S9I–S9M). Furthermore, we previously demonstrated that blood and metastatic lesions of WT and Chi3l1 OE mice exhibited comparable levels of neutrophil and NE$^{+}$ cell infiltration, indicating that the increased metastatic burden in Chi3l1 OE mice is not associated with increased neutrophil accumulation or NE expression at the metastatic site.[25]

Together, these findings indicate that NE exerts its greatest effects during early disease progression and are consistent with preservation of myoepithelial integrity as major mechanisms underlying the anti-metastatic effects of NE inhibition in this model, rather than direct suppression of established tumors or metastatic lesions.

## NE-mediated ECM degradation induces anoikis of myoepithelial cells

We next sought to determine whether NE-mediated myoepithelial disruption was driven by loss of myoepithelial cells or by alterations in myoepithelial differentiation state. Studies have shown that progression from DCIS to IDC is accompanied by progressive myoepithelial de-differentiation, characterized by the sequential loss of differentiation markers including P63 and calponin 1.[36,37] To investigate whether neutrophils regulate myoepithelial differentiation in our model, we re-analyzed our immunofluorescence data and quantified total CK14$^{+}$ myoepithelial cells together with partially differentiated CK14$^{+}$ P63$^{-}$ and CK14$^{+}$ calponin 1$^{-}$ populations as a percentage of total CK14$^{+}$ cells. Consistent with our previous findings, neutrophil depletion or inhibition of neutrophil degranulation significantly increased the abundance of total CK14$^{+}$ myoepithelial cells (Figure S10A). However, the proportion of CK14$^{+}$ P63$^{-}$ and CK14$^{+}$ calponin 1$^{-}$ cells relative to total CK14$^{+}$ cells remained unchanged between treatment groups (Figures S10A–S10F). These data demonstrate that neutrophils drive DCIS progression in our models by promoting myoepithelial cell loss rather than inducing myoepithelial de-differentiation.

Myoepithelial cells exist in a highly specialized physical niche, where their survival and function depend on direct adhesion to

(F) Fluorescent IHC staining of WT and NE$^{-/-}$ MMTV-PyMT end-stage mammary tumors using antibodies against CK14, calponin 1, P63, α-SMA, and CK8 counterstained with DAPI.

(G–J) Quantification of total CK14$^{+}$ α-SMA$^{+}$ (Cohen's d = −1.1790), CK14$^{+}$ calponin 1$^{+}$ (Cohen's d = −1.0529), CK14$^{+}$ P63$^{+}$ (Cohen's d = −1.0780), and CK14$^{+}$ calponin 1$^{+}$ P63$^{+}$ (Cohen's d = −1.0049) cells normalized to total analyzed area in WT ($n$ = 9) and NE$^{-/-}$ ($n$ = 14) MMTV-PyMT mammary tumors. Derived from the staining shown in (F).

(K) Representative H&E staining of Chi3l1 OE MIC lungs treated with vehicle or alvelestat for 6 weeks. On the left side are mice in which treatment was started from the time of induction (prior to myoepithelial disruption) and on the right are mice in which treatment was started after 2 weeks of induction (after the myoepithelial layer was disrupted).

(L) Percentage of Chi3l1 OE MIC mice with pulmonary metastases after treatment with vehicle or alvelestat for 6 weeks.

(M) Quantification of total pulmonary metastatic area in Chi3l1 OE MIC lungs after treatment with vehicle ($n$ = 7) or alvelestat ($n$ = 6) from the time of induction (Cohen's d = 2.5843) or treated with vehicle ($n$ = 4) or alvelestat ($n$ = 4) after 2 weeks of induction. Both experiments were completed at 6 weeks of induction. Represented as percent of total lung area.

*$p < 0.05$, **$p < 0.01$, ***$p < 0.001$, and ****$p < 0.0001$ calculated by Mann-Whitney non-parametric test, Fisher's exact test (for L). Data are presented as mean ± SEM. Each data point is an independent biological sample, and $N$ represents the number of independent biological replicates. Analysis was performed by the HALO (Indica Labs) software. Quantification was performed on whole-slide scans of the entire mammary gland section. Scale bars: 10 (A and F) and 500 μm (K).

ECM components.[38] Engagement of β1-integrins with laminin and collagen IV activates focal adhesion kinase (FAK) signaling,[39] which is essential for myoepithelial survival and resistance to anoikis. Because NE is a serine protease known to degrade laminin and collagen IV, we hypothesized that NE-mediated ECM degradation disrupts integrin-dependent survival signaling, leading to myoepithelial cell death by anoikis.

To assess whether neutrophils regulate basement membrane integrity *in vivo*, we examined ECM composition in neutrophil-depleted mammary glands by staining for laminin and collagen IV. Anti-Ly6G-treated glands exhibited a significant increase in ECM components, as measured by the area occupied by laminin or collagen IV, compared to IgG2a-treated controls (Figures 4A–4C). Similar preservation of ECM components was observed following NE inhibition in both WT and Chi3l1 OE MIC mice (Figures 4D–4I).

To independently validate the role of NE in ECM degradation, we performed *in vitro* ECM degradation assays. $PyMT^{+}$ tumor cells were cultured for 12 h on a synthetic ECM layer in conditioned media derived from murine neutrophils treated with alvelestat or vehicle control. Staining revealed significantly increased laminin coverage when tumor cells were cultured in alvelestat-treated neutrophil media compared to vehicle-treated media, indicating loss of laminin degradation after inhibiting NE (Figures S11A and S11B). In parallel, $PyMT^{+}$ cells were cultured on fluorescently labeled collagen IV matrices in conditioned media derived from murine neutrophils treated with alvelestat or vehicle control. This matrix utilizes quenched probes that only fluoresce green when collagen IV is cleaved. Exposure to vehicle-treated neutrophil-conditioned media resulted in marked fluorescence, indicative of collagen IV degradation, whereas alvelestat-treated neutrophil media caused a significant reduction in fluorescence, demonstrating impaired collagen IV degradation (Figures S11C and S11D).

As neutrophils release a variety of factors that remodel the ECM, we sought to validate their role in ECM degradation. Laminin and collagen IV degradation assays were repeated in the presence of either recombinant NE, recombinant Chi3l1, recombinant proteinase 3 (PR3), or recombinant cathepsin G. Consistent with our prior results, recombinant NE caused significant degradation of laminin and collagen IV, like the effects of neutrophil-conditioned media (Figures S11E–S11H). Recombinant cathepsin G and PR3 had no detectable effect on laminin or collagen IV coverage (Figures S11E–S11H), indicating that NE is the primary protease responsible for ECM degradation under these experimental conditions. Recombinant Chi3l1 also had no impact on the ECM, confirming that the phenotypes observed in Chi3l1 OE mice were driven by neutrophils and not due to the effects of Chi3l1 on the ECM (Figures S11E–S11H). To independently confirm the role of NE in ECM rewiring and cancer cell invasion, we performed an *in vitro* transwell invasion assay, culturing $PyMT^{+}$ cells on a synthetic ECM in the presence of conditioned neutrophil medium treated with vehicle or alvelestat. While neutrophil-conditioned medium stimulated cancer cell invasion, this effect was reduced in media treated with alvelestat (Figures S11I and S11J). Collectively, these *in vivo* and *in vitro* data establish NE as a key mediator of basement membrane ECM degradation.

We next evaluated whether ECM degradation leads to myoepithelial cell anoikis. To assess this, we performed staining for cleaved caspase-3 (CCP3), a marker of cell death, and p-FAK (phosphorylated focal adhesion kinase), a readout of β1-integrin-ECM engagement. Both markers were combined with CK14 to assess their levels specifically in the myoepithelial compartment. In neutrophil-depleted glands, we observed a significant reduction in $CCP3^{+}$ myoepithelial cells compared with controls, accompanied by a marked increase in p-$FAK^{+}$ myoepithelial cells, without a change in cancer cell death (Figures S11K–S11O). These findings indicate that neutrophil depletion preserved integrin-mediated survival signaling and protected myoepithelial cells from anoikis.

We next assessed CCP3 and p-FAK expression in alvelestat-treated WT and Chi3l1 OE MIC mammary glands. In both models, NE inhibition caused a significant decrease in $CCP3^{+}$ myoepithelial cells and a concomitant increase in p-$FAK^{+}$ myoepithelial cells compared to vehicle-treated controls, while tumor cell death remained unchanged (Figures 5A–5J). These data demonstrate that NE-mediated ECM degradation disrupts integrin-dependent survival signaling, leading to myoepithelial cell anoikis.

To further validate these findings, we stained for myoepithelial and neutrophil markers across a larger number of MIC mammary glands. Consistent with our model, NE levels were inversely correlated with the abundance of myoepithelial cells (Figures S12A–S12D). Importantly, NE levels were negatively correlated with laminin content and the number of p-$FAK^{+}CK14^{+}$ myoepithelial cells and positively correlated with $CCP3^{+}$ myoepithelial cells (Figures S12E–S12I), which suggested an association between NE accumulation, loss of adhesion signaling, and induction of myoepithelial cell death. Spatial distance analysis further demonstrated that $NE^{+}$ cells were preferentially localized near p-$FAK^{-}$ myoepithelial cells and near $CCP3^{+}$ myoepithelial cells (Figures S12J and S12K).

To validate our findings in an isolated *in vitro* system, we generated organoids from CK14-TdTomato-MIC mammary glands, in which myoepithelial cells are fluorescently labeled with TdTomato.[40] Organoids were embedded in an ECM-rich three-dimensional matrix that preserves mammary duct-like architecture and myoepithelial organization. Treatment with recombinant NE resulted in significant loss of $CK14^{+}$ myoepithelial cells after 24 h of treatment, whereas PBS-treated control organoids maintained an intact myoepithelial layer (Figures 5K and 5L; Figure S12L). These data demonstrate that NE is sufficient to drive myoepithelial cell loss independently of systemic factors and further support a direct role for NE in disrupting myoepithelial integrity.

Collectively, our *in vivo* and *in vitro* analyses define a mechanism through which neutrophil-derived NE degrades basement membrane ECM, compromises integrin-mediated survival signaling, and induces myoepithelial cell death through anoikis.

### DCIS patient samples with elevated NE exhibit myoepithelial barrier disruption

To validate our findings in a human model of DCIS progression, we analyzed mammary glands from the MCF7-DCIS mouse

CellPress
OPEN ACCESS

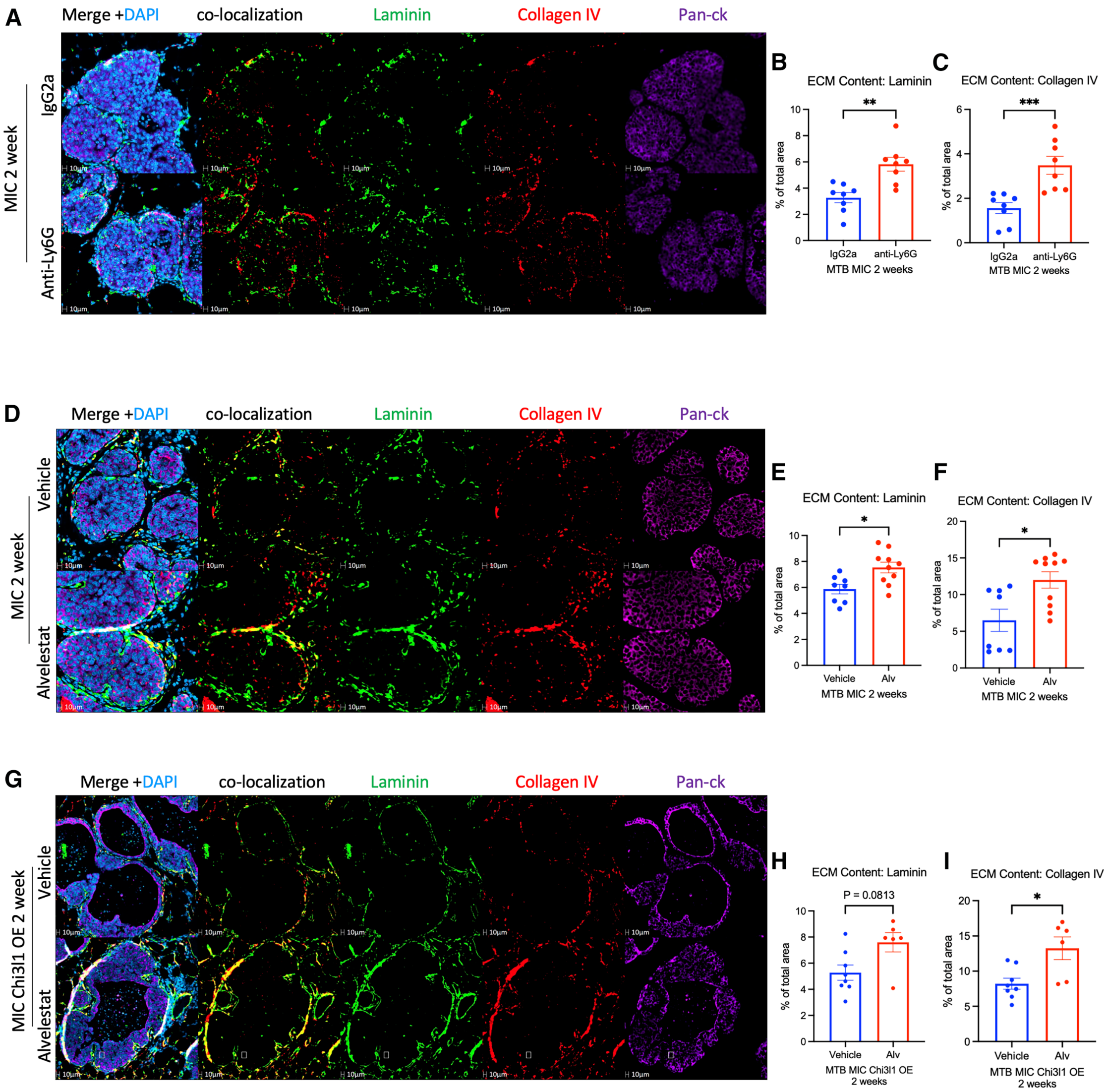


**Figure 4. Targeting NE prevents ECM degradation**

(A) Fluorescent IHC staining of mammary tissue from WT MIC mice treated with anti-Ly6G or IgG2a isotype control for 2 weeks, using antibodies against laminin, collagen IV, and Pan-ck, counterstained with DAPI.

(B and C) Quantification of area occupied by laminin (Cohen's d = −1.9433) and collagen IV (Cohen's d = −2.0424) in IgG2a ($n$ = 8) and anti-Ly6G ($n$ = 8)-treated WT MIC mammary glands. Represented as percent of mammary gland area. Derived from the staining shown in (A).

(D) Fluorescent IHC staining of mammary tissue from WT MIC mice treated with vehicle or alvelestat for 2 weeks, using antibodies against laminin, collagen IV, and Pan-ck, counterstained with DAPI (blue).

(E and F) Quantification of area occupied by laminin (Cohen's d = −1.3939) and collagen IV (Cohen's d = −1.4118) in vehicle ($n$ = 8) and alvelestat ($n$ = 10)-treated WT MIC mammary glands. Represented as percent of mammary gland area. Derived from the staining shown in (D).

(G) Fluorescent IHC staining of mammary tissue from Chi3l1 OE MIC mice treated with vehicle or alvelestat for 2 weeks, using antibodies against laminin, collagen IV, and Pan-ck, counterstained with DAPI.

(H and I) Quantification of area occupied by laminin (Cohen's d = −1.3560) and collagen IV (Cohen's d = −1.6446) in vehicle ($n$ = 8) and alvelestat ($n$ = 6)-treated Chi3l1 OE MIC mammary glands. All data represented as percent of mammary gland area. Derived from the staining shown in (G).

*$p < 0.05$ and **$p < 0.01$ calculated by Mann-Whitney non-parametric test. Data are presented as mean ± SEM. Each data point is an independent biological sample, and $N$ represents the number of independent biological replicates. Analysis was performed by the HALO (Indica Labs) software. Quantification was performed on whole-slide scans of the entire mammary gland section. Scale bars: 10 μm.

mammary intraductal (MIND)[41,42] model containing either non-invasive DCIS lesions or lesions that had progressed to invasive disease. Staining revealed a marked increase in $NE^+$ cells within invasive samples compared to non-invasive DCIS lesions (Figures S13A and S13B). Consistent with our findings in GEMMs, progression to invasive disease was accompanied by a profound loss of myoepithelium and laminin coverage (Figures S13A–S13D). These findings extend our observations

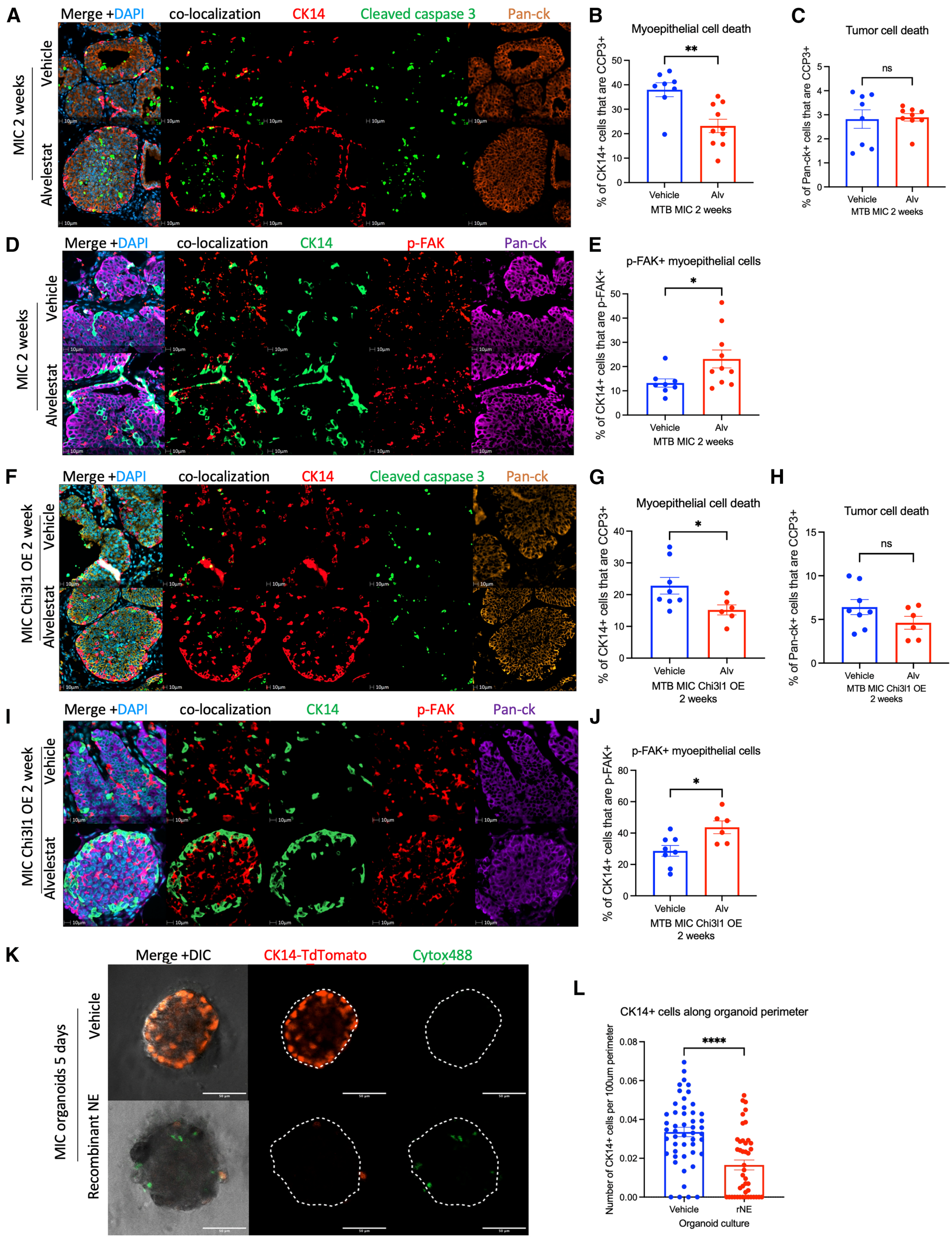


**Figure 5. Targeting NE prevents myoepithelial layer anoikis**

(A) Fluorescent IHC staining of mammary tissue from WT MIC mice treated with vehicle or alvelestat for 2 weeks, using antibodies against CK14, cleaved caspase-3, and Pan-ck, counterstained with DAPI.

(B) Quantification of the percentage of $CK14^{+}$ cleaved caspase-$3^{+}$ cells within total $CK14^{+}$ cells in vehicle (*n* = 8) and alvelestat (*n* = 10)-treated WT MIC mammary glands (Cohen's d = 1.7575). Derived from the staining shown in (A).

(C) Quantification of the percentage of Pan-$ck^{+}$ cleaved caspase-$3^{+}$ cells within total Pan-$ck^{+}$ cells in vehicle (*n* = 8) and alvelestat (*n* = 10)-treated WT MIC mammary glands (Cohen's d = −0.0604). Derived from the staining shown in (A).

*(legend continued on next page)*

to an independent human DCIS progression model and demonstrate that invasive transition is associated with increased NE accumulation, ECM degradation, and myoepithelial loss.

To assess the clinical relevance of our findings, we next examined neutrophil infiltration and myoepithelial integrity in human breast cancer. Staining for MPO as a neutrophil marker and CK14 was performed on breast cancer tissue microarrays encompassing normal breast tissue, DCIS, and IDC (Figure 6A). Consistent with prior reports,[20] DCIS samples exhibited significantly higher neutrophil infiltration compared to normal breast tissue (Figures 6A and 6B). Notably, neutrophil abundance did not differ between DCIS and IDC samples, indicating that neutrophil accumulation occurs early during disease progression (Figures 6A and 6B). Importantly, quantitative analysis revealed an inverse correlation between neutrophil abundance and myoepithelial content within DCIS lesions (Figures 6A–6C). DCIS samples with elevated neutrophil infiltration exhibited marked disruption of the myoepithelium compared with neutrophil-low lesions (Figures 6A–6C). Importantly, this inverse correlation was observed in both estrogen receptor $(ER)^+$ and $ER^-$ DCIS lesions, indicating that the relationship is unlikely to be driven solely by ER status (Figures S13E and S13F).

Given our experimental data identifying NE as a mediator of myoepithelial disruption, we next stained an independent cohort of DCIS samples for NE and myoepithelial markers. Consistent with our mechanistic findings, elevated NE expression strongly correlated with reduced myoepithelial levels and compromised myoepithelial architecture in DCIS lesions (Figures 6D–6F). Importantly, NE levels did not differ between grade II and grade III DCIS samples, indicating that the phenotype was independent of tumor grade (Figure S13G). To determine whether the relationship between NE accumulation, loss of focal adhesion signaling, and myoepithelial cell death was also evident in human disease, we stained for CK14, p-FAK, CCP3, and NE in human DCIS specimens. Consistent with our findings in GEMMs, regions exhibiting elevated NE levels displayed reduced numbers of p-FAK$^+$ myoepithelial cells and increased CCP3$^+$ myoepithelial cells (Figures S13H–S13O). Quantitative analysis revealed a significant negative correlation between NE abundance and p-FAK$^+$ myoepithelial cells and a positive correlation between NE abundance and apoptotic CCP3$^+$ myoepithelial cells (Figures S13H–S13O). Spatial analysis further demonstrated that NE$^+$ cells were preferentially localized near p-FAK$^-$ and CCP3$^+$ myoepithelial cells (Figures S13M and S13O). Interestingly, NE$^+$ cells were also localized near regions of non-myoepithelial cell death (Figure S13P), suggesting that NE-associated activity may extend beyond myoepithelial disruption and contribute to localized tissue damage. Together, these findings provide independent validation in human DCIS that local NE accumulation is associated with loss of focal adhesion signaling and induction of myoepithelial cell death, supporting a conserved mechanism of NE-mediated myoepithelial barrier disruption during breast cancer progression.

To correlate our findings in the clinical setting, we analyzed independent patient cohorts and publicly available transcriptomic datasets. Immunofluorescence staining of primary breast cancer specimens revealed significantly higher NE levels in tumors from patients who subsequently developed metastatic disease compared with tumors from patients who remained metastasis-free (Figures 6G and 6H). To extend these findings to pre-invasive disease, we interrogated a publicly available DCIS progression dataset[43] and observed a trend toward increased *NE* expression in DCIS lesions that subsequently progressed to invasive disease compared with non-progressing lesions, although this difference did not reach statistical significance (Figure 6I). Given the low abundance and spatially restricted nature of neutrophils in DCIS lesions, bulk transcriptomic analyses may underestimate localized NE activity within the tumor microenvironment.

Finally, Kaplan-Meier survival analyses demonstrated that elevated *NE* expression was associated with poorer survival outcomes in breast cancer patients (Figure 6J). To determine whether this association reflected a broader neutrophil abundance signature, we examined the prognostic associations of

(D) Fluorescent IHC staining of mammary tissue from WT MIC mice treated with vehicle or alvelestat for 2 weeks, using antibodies against CK14, p-FAK, and Pan-ck, counterstained with DAPI.

(E) Quantification of the percentage of CK14$^+$ p-FAK$^+$ cells within total CK14$^+$ cells in vehicle ($n$ = 8) and alvelestat ($n$ = 10)-treated WT MIC mammary glands (Cohen's d = −1.0545). Derived from the staining shown in (D).

(F) Fluorescent IHC staining of mammary tissue from Chi3l1 OE MIC mice treated with vehicle or alvelestat for 2 weeks, using antibodies against CK14, cleaved caspase-3, and Pan-ck, counterstained with DAPI.

(G) Quantification of the percentage of CK14$^+$ cleaved caspase-3$^+$ cells within total CK14$^+$ cells in vehicle ($n$ = 8) and alvelestat ($n$ = 6)-treated Chi3l1 OE MIC mammary glands (Cohen's d = 1.2349). Derived from the staining shown in (F).

(H) Quantification of the percentage of Pan-ck$^+$ cleaved caspase-3$^+$ cells within total Pan-ck$^+$ cells in vehicle ($n$ = 8) and alvelestat ($n$ = 6)-treated Chi3l1 OE MIC mammary glands (Cohen's d = 0.8193). Derived from the staining shown in (F).

(I) Fluorescent IHC staining of mammary tissue from Chi3l1 OE MIC mice treated with vehicle or alvelestat for 2 weeks, using antibodies against CK14, p-FAK, and Pan-ck, counterstained with DAPI.

(J) Quantification of the percentage of CK14$^+$ p-FAK$^+$ cells within total CK14$^+$ cells in vehicle ($n$ = 8) and alvelestat ($n$ = 6)-treated Chi3l1 OE MIC mammary glands (Cohen's d = −1.5316). Derived from the staining shown in (I).

(K) Live fluorescent visualization of organoids grown from MIC Ck14-TdTomato mammary glands induced in culture for 5 days and treated with vehicle or recombinant neutrophil elastase for 24 h. Red indicates endogenous K14-driven TdTomato. Green indicates Incucyte Cytotox Dye, which marks dead cells. The white dotted line outlines the organoid seen by DIC.

(L) Quantification of total CK14$^+$ cells per 100 μm perimeter in NE ($n$ = 43 organoids, technical replicate) or vehicle-treated ($n$ = 49 technical replicate) organoids (Cohen's d = 1.0088). Results are pooled from 3 technical replicates. Derived from the staining shown in (K).

Ns, not significant, $^*p < 0.05$, $^{**}p < 0.01$, $^{***}p < 0.001$, and $^{****}p < 0.0001$ calculated by Mann-Whitney non-parametric test. Data are presented as mean ± SEM. Each data point is an independent biological sample, and $N$ represents the number of independent biological replicates. Analysis was performed by the HALO (Indica Labs) software. Quantification was performed on whole-slide scans of the entire mammary gland section. Scale bars: 10 (A, D, F, and I) and 50 μm (K).

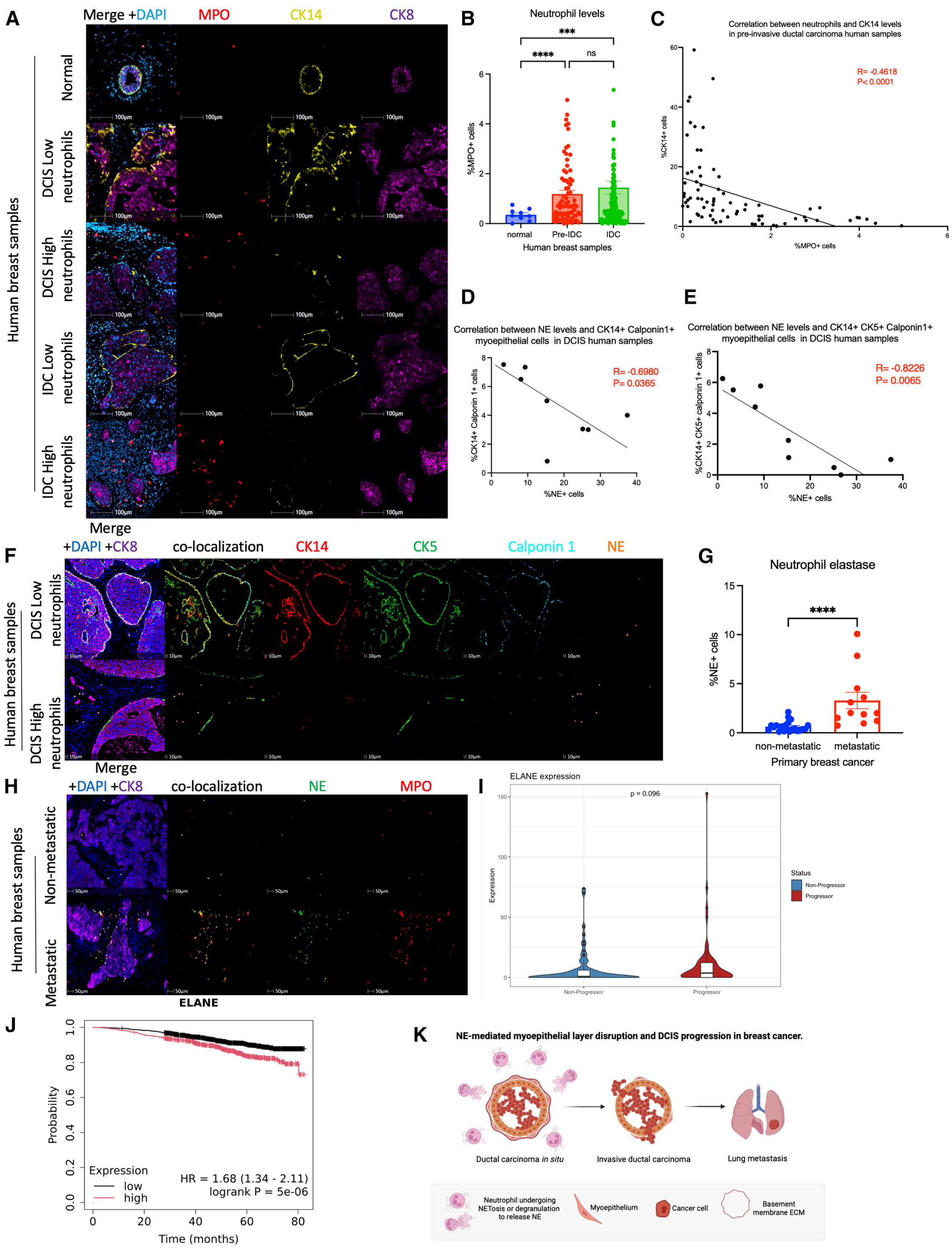


**Figure 6. NE correlates with myoepithelial layer loss in human DCIS samples**

(A) Fluorescent IHC staining of human normal breast tissue, pre-invasive ductal carcinoma, and invasive ductal carcinoma samples using antibodies against MPO, CK14, and CK8, counterstained with DAPI. Pre-IDC samples include hyperplasia, atypia, and DCIS. Micrograph represents areas of high neutrophils and low neutrophils from different DCIS or IDC patient samples.

(B) Quantification of total $MPO^{+}$ cells as a percentage of total cells in normal ($n$ = 9), DCIS ($n$ = 75), and IDC ($n$ = 207) human patient samples. Derived from the staining shown in (A).

*(legend continued on next page)*

additional neutrophil-associated markers. While some markers, including *MPO*, *CXCR2*, and *S100A8*, were associated with poorer overall survival, *S100A9* was associated with improved survival, whereas *CXCR1* and *PRTN1* did not show significant associations (Figures S14A–S14G). These divergent associations suggest that the prognostic relationship observed for *NE* is not simply attributable to overall neutrophil abundance and may instead reflect distinct neutrophil functional states or programs.

Collectively, analyses of human DCIS samples support our *in vivo* findings and suggest a potential association between neutrophil-derived NE and myoepithelial barrier disruption during DCIS progression. Mechanistically, NE promotes ECM degradation, which leads to myoepithelial cell death through anoikis and breast cancer progression (Figure 6K).

## DISCUSSION

DCIS is a commonly diagnosed early stage of breast cancer that is marked by the presence of an intact myoepithelial layer.[1,3] This layer acts as a physical and biochemical barrier that restricts tumor cell invasion.[3,11] Loss of myoepithelial integrity is among the strongest histopathological predictors of invasion in DCIS and poor outcomes.[36,44,45] Yet the cellular and molecular mechanisms driving this process remain poorly understood, limiting our ability to identify patients at risk of progression and develop mechanism-based preventive strategies. Using complementary GEMMs and human DCIS samples, we identified neutrophil-derived NE as a key mediator of myoepithelial layer disruption and DCIS progression.

Myoepithelial disruption is likely multifactorial, involving altered differentiation, stromal remodeling, and inflammatory signaling.[9,10,46,47] Previous studies have associated loss of differentiation markers P63, calponin, and α-SMA with microinvasion.[36,37] Our findings complemented these models by identifying a distinct mechanism whereby neutrophils promoted physical loss of myoepithelial cells through protease-mediated ECM degradation and anoikis. Consistent with this interpretation, targeting neutrophils or NE increased total myoepithelial abundance without altering the proportion of differentiated myoepithelial subsets. These findings support a stepwise model in which localized NE degrades the ECM, disrupts β1-integrin signaling, induces myoepithelial anoikis, and facilitates invasive transition.

Emerging evidence argues that tumor-extrinsic factors within the TIME play a decisive role in determining DCIS behavior.[48,49] Multiple stromal populations, including macrophages, lymphocytes, and fibroblasts, have been implicated in invasive progression through effects on epithelial behavior, stromal remodeling, and ECM organization.[9,20,46,50,51] Immune infiltration patterns have been associated with aggressive DCIS and increased risk of progression.[12,46,50,52] However, the mechanisms through which immune cells promote myoepithelial barrier disruption have remained poorly defined. Our findings identify neutrophils as active mediators of this process through localized release of NE. Future studies defining the spatial and temporal contributions of other immune populations will further clarify how the TIME regulates invasive progression.

NE is a neutrophil-derived serine protease that modulates the TIME, degrades ECM components, and is associated with poor prognosis.[32,53] While neutrophils have been observed in DCIS patient samples,[20] their functional contribution to myoepithelial disruption has remained largely unexplored. Here, using complementary genetic, pharmacological, and clinical samples, we identified NE as a critical mediator of myoepithelial disruption and invasive progression. Given the availability of selective NE inhibitors with established safety profiles in clinical trials,[54,55] these findings provide a rationale for investigating whether NE inhibition may have utility in early-stage DCIS.

Elevated neutrophil abundance is associated with poor patient outcomes and immunosuppression across multiple cancer types.[13,17] However, direct therapeutic depletion of neutrophils is not clinically feasible because of their essential role in host defense. Targeting tumor-enriched neutrophil-derived effector molecules, such as NE, or upstream tumor-derived chemoattractants that regulate neutrophil recruitment may represent

(C) Pearson correlation between neutrophils (MPO$^+$ cells) and the total CK14$^+$ cells in human DCIS samples ($n$ = 75). Both presented as a percentage of total cells. Derived from the staining shown in (A).

(D and E) Pearson correlation between NE$^+$ cells and total CK14$^+$ calponin 1$^+$ cells or CK14$^+$ CK5$^+$ calponin 1$^+$ cells in human DCIS samples ($n$ = 9). All presented as a percentage of total cells. Derived from the staining shown in (F).

(F) Fluorescent IHC staining of human DCIS samples using antibodies against NE, CK14, CK5, calponin 1, and CK8, counterstained with DAPI. Micrograph represents areas of high NE and low NE from different DCIS patient samples.

(G) Quantification of total NE$^+$ cells as a percentage of total cells in non-metastatic ($n$ = 21) or metastatic ($n$ = 12) breast cancer samples (Cohen's d = −1.4931). Derived from the staining shown in (H).

(H) Fluorescent IHC staining of human non-metastatic or metastatic breast cancer samples using antibodies against NE, MPO, and CK8, counterstained with DAPI.

(I) Expression levels of *ELANE* (NE) in human DCIS samples that progressed to invasive disease compared to DCIS samples that did not progress, analyzed using the Wilcoxon rank-sum test.

(J) Elevated levels of *NE* are associated with decreased overall survival in breast cancer patients of all subtypes at 5 years post-diagnosis ($n$ = 2,976).

(K) Summary of the proposed model for NE-mediated myoepithelial layer disruption and DCIS progression. Neutrophils release NE during NETosis or degranulation, which degrades the ECM, leading to myoepithelial cell death by anoikis. This marks the transition to invasive disease that can metastasize to distant organs.

Created with BioRender.com. Ns, not significant, $^{**}p < 0.01$, $^{***}p < 0.001$, and $^{****}p < 0.0001$ calculated by Brown-Forsythe and Welch ANOVA tests with Dunnett's T3 multiple comparison post hoc test (for B) or Mann-Whitney non-parametric test (for G). Data are presented as mean ± SEM. Pearson correlation was performed using two-tailed $t$ test. Each data point is an independent biological sample, and $N$ represents the number of independent biological replicates. Analysis was performed by the HALO (Indica Labs) software. Quantification was performed on whole-slide scans of the entire mammary gland section. Scale bars: 100 (A) and 10 μm (F andH).

more selective therapeutic strategies. Importantly, neutrophils release numerous proteases and collagenases capable of degrading the ECM, which may also contribute to myoepithelial disruption. Defining the relative contribution of these proteases during DCIS progression may uncover additional therapeutic opportunities and improve our understanding of neutrophil biology in early breast cancer.

Although neutrophils were relatively sparse within mammary lesions, their biological impact is determined by localized protease release rather than absolute abundance. Unlike many immune populations that act primarily through cell-cell interactions, neutrophils release highly potent proteases capable of sustained ECM degradation following degranulation.[13,56] Furthermore, neutrophils are notoriously difficult to capture using conventional transcriptomic approaches due to their short half-life and susceptibility to processing-induced loss.[57–59] Our findings indicate that the spatial localization of neutrophils may be more informative than their overall abundance. $NE^+$ neutrophils were observed in both intratumoral and peritumoral regions but preferentially localized near sites of myoepithelial disruption, supporting a model in which localized protease release directly compromises barrier integrity. Despite their relatively low abundance, targeting neutrophils or NE preserved myoepithelial integrity and suppressed disease progression. Future studies should determine whether spatial analysis of NE-positive cells in DCIS biopsy samples can improve risk stratification in DCIS.

While Chi3l1 was used in the present study primarily as a genetic platform to modulate neutrophil recruitment, these findings should be viewed within the broader biology of the Chi3l1 pathway. We previously demonstrated that STAT3 (signal transducer and activator of transcription 3) is a direct transcriptional regulator of Chi3l1, with chromatin immunoprecipitation confirming STAT3 binding to the Chi3l1 promoter and genetic Stat3 ablation markedly reducing Chi3l1 expression and neutrophil recruitment.[24,25] Furthermore, Chi3l1 deficiency broadly remodels the TIME by enhancing T cell infiltration and activation, a phenotype that was phenocopied by neutrophil depletion, indicating that these immunologic effects are largely neutrophil dependent.[24] Interestingly, NE inhibition preserved myoepithelial integrity without altering T cell abundance or activation, indicating that neutrophils promote breast cancer progression through at least two distinct mechanisms: an NE-dependent pathway driving ECM degradation and myoepithelial disruption and an NE-independent pathway regulating immune remodeling. Defining the mediators responsible for this second program represents an important area for future investigation.

In summary, our findings identify neutrophils and NE as key regulators of myoepithelial barrier integrity during DCIS progression. Neutrophil degranulation and NE release promote ECM degradation, impair $\beta$1-integrin signaling, and trigger myoepithelial anoikis, thereby facilitating invasive transition. These findings establish a mechanistic link between inflammation and myoepithelial layer disruption and support further investigation of neutrophil-derived proteases during early breast cancer progression.

### Limitations of the study

Several limitations of this preclinical study should be considered. Although our data identified NE as a mediator of myoepithelial disruption, neutrophils release numerous additional proteases and inflammatory mediators that may also contribute.[53,60] Future studies should define the relative contribution of these pathways, determine the temporal window during which neutrophil activity irreversibly compromises myoepithelial integrity, and investigate how Chi3l1 regulates neutrophil activation, mobilization from the bone marrow, and NE release.

An additional limitation relates to the clinical evaluation of NE as a biomarker of DCIS progression. Although patient cohorts supported an association between elevated NE and disease progression, larger clinically annotated cohorts incorporating spatial proteomics, multiplex imaging, circulating biomarkers, and long-term follow-up will be required to establish the prognostic utility of NE. Functional validation in additional human DCIS models will further strengthen translational relevance. Addressing these questions will clarify the biological and potential clinical significance of NE during DCIS progression.

## RESOURCE AVAILABILITY

### Lead contact

Further information and requests for resources and reagents should be directed to and will be fulfilled by the lead contact, William J. Muller (william.muller@mcgill.ca).

### Materials availability

All unique reagents or mouse models used in this study may be made available upon request from the lead contact with a completed materials transfer agreement.

### Data and code availability

- This paper analyzes existing, publicly available data, accessible at the Gene Expression Omnibus GSE281303.[43] All data reported in this paper will be shared by the lead contact upon request.
- This paper does not report original code.
- Any additional information required to reanalyze the data reported in this paper is available from the lead contact upon request.

## ACKNOWLEDGMENTS

We thank the Histology, Transgenic, Flow Cytometry, Bioinformatics, Microscopy, and Animal Husbandry Core Facilities at the Goodman Cancer Institute, McGill University, and Mitra Cowen from the McGill Integrated Core for Animal Modeling. T.T. is supported by Vanier Canada Graduate Scholarship (CIHR; CGV-192738). Y.G. is supported by CIHR Canada Graduate Scholarship–Doctoral. A.M. is supported by the Donner Foundation Studentship Award (Goodman Cancer Institute, McGill University), the Rolande and Marcel Gosselin Graduate Fellowship, and the Carole Epstein Fellowship in Health (Faculty of Medicine, McGill University). X.M. is supported by a Fonds de Recherche du Québec–Santé Master's Research Scholarship. C.D.E. is supported by a Canada Graduate Scholarship–Master's. E.S. is supported by a Cancer Research Society Doctoral Research Award. The Breast Cancer Functional Genomics Group (BCFGG) Biobank at the Goodman Cancer Institute/MUHC is supported by the Réseau de Recherche en Cancer of the Fonds de Recherche du Québec–Santé and the Quebec Breast Cancer Foundation and is certified by the Canadian Tumor Repository Network (CTRNet). W.J.M. is a Distinguished James McGill Professor and Canada Research Chair in Molecular Oncology. This work was supported by the Terry Fox Research Institute Program Project Grant (P.M.S. and W.J.M.); the CIHR Foundation (148373), Bridge (PLL-185690) and Project (PJT-190027) grants; the Canadian Cancer Society Impact Grant; and the Cancer Research Society (1281449).

CellPress
OPEN ACCESS

## AUTHOR CONTRIBUTIONS

Conceptualization, T.T., Y.G., and W.J.M.; investigation, T.T., Y.G., A.M., X.M., C.D.E., D.Z., V.S.-G., A.B., A.-L.G.-P., M.A., B.X., E.S., H.P., S.S.A., and A.J.N.; analysis, T.T., Y.G., A.M., X.M., C.D.E., V.S.-G., A.B., A.-L.G.-P., and L.M.; resources, M.A., H.K., P.M.S., K.K., L.M., and M.P.; writing – original draft, T.T. and W.J.M.; review and editing, T.T., Y.G., A.M., X.M., C.D.E., D.Z., E.S., B.X., H.P., S.S.A., H.K., P.M.S., K.K., M.P., and W.J.M.; supervision, W.J.M.; and funding acquisition, W.J.M.

## DECLARATION OF INTERESTS

At the time of publication, S.S.A. is an employee of Genentech, a member of Roche Group.

## STAR★METHODS

Detailed methods are provided in the online version of this paper and include the following:

- KEY RESOURCES TABLE
- EXPERIMENTAL MODEL AND STUDY PARTICIPANT DETAILS
  - Animals-mouse models
  - Human participants
  - Primary cell culture and cell lines
- METHOD DETAILS
  - Pharmacological treatments
  - Mammary intraductal engraftment
  - Human bioinformatic analysis
  - RNA FISH
  - Protein extraction and immunoblots
  - Multiplex fluorescent immunohistochemistry
  - FACS analysis
  - Enzyme-linked immunosorbent assay
  - *In vitro* laminin degradation assay
  - *In vitro* collagen IV degradation assay
  - Trans-well invasion assay
  - Organoid culture and NE treatment
- QUANTIFICATION AND STATISTICAL ANALYSIS

## SUPPLEMENTAL INFORMATION

Supplemental information can be found online at https://doi.org/10.1016/j.xcrm.2026.103050.

Received: February 9, 2026
Revised: June 29, 2026
Accepted: August 19, 2026
Published: September 11, 2026

## REFERENCES

1. Groen, E.J., Elshof, L.E., Visser, L.L., Rutgers, E.J.T., Winter-Warnars, H.A.O., Lips, E.H., and Wesseling, J. (2017). Finding the balance between over- and under-treatment of ductal carcinoma in situ (DCIS). Breast *31*, 274–283. https://doi.org/10.1016/j.breast.2016.09.001.
2. Barrio, A.V., and Van Zee, K.J. (2017). Controversies in the Treatment of Ductal Carcinoma in Situ. Annu. Rev. Med. *68*, 197–211. https://doi.org/10.1146/annurev-med-050715-104920.
3. Pandey, P.R., Saidou, J., and Watabe, K. (2010). Role of myoepithelial cells in breast tumor progression. Front. Biosci. *15*, 226–236. https://doi.org/10.2741/3617.
4. Pareja, F., Brown, D.N., Lee, J.Y., Da Cruz Paula, A., Selenica, P., Bi, R., Geyer, F.C., Gazzo, A., da Silva, E.M., Vahdatinia, M., et al. (2020). Whole-Exome Sequencing Analysis of the Progression from Non-Low-Grade Ductal Carcinoma In Situ to Invasive Ductal Carcinoma. Clin. Cancer Res. *26*, 3682–3693. https://doi.org/10.1158/1078-0432.Ccr-19-2563.
5. Pang, J.M.B., Savas, P., Fellowes, A.P., Mir Arnau, G., Kader, T., Vedururu, R., Hewitt, C., Takano, E.A., Byrne, D.J., Choong, D.Y., et al. (2017). Breast ductal carcinoma in situ carry mutational driver events representative of invasive breast cancer. Mod. Pathol. *30*, 952–963. https://doi.org/10.1038/modpathol.2017.21.
6. Sanati, S. (2019). Morphologic and Molecular Features of Breast Ductal Carcinoma in Situ. Am. J. Pathol. *189*, 946–955. https://doi.org/10.1016/j.ajpath.2018.07.031.
7. Wang, J., Li, B., Luo, M., Huang, J., Zhang, K., Zheng, S., Zhang, S., and Zhou, J. (2024). Progression from ductal carcinoma in situ to invasive breast cancer: molecular features and clinical significance. Signal Transduct. Targeted Ther. *9*, 83. https://doi.org/10.1038/s41392-024-01779-3.
8. Gibson, S.V., Roozitalab, R.M., Allen, M.D., Jones, J.L., Carter, E.P., and Grose, R.P. (2023). Everybody needs good neighbours: the progressive DCIS microenvironment. Trends Cancer *9*, 326–338. https://doi.org/10.1016/j.trecan.2023.01.002.
9. Linde, N., Casanova-Acebes, M., Sosa, M.S., Mortha, A., Rahman, A., Farias, E., Harper, K., Tardio, E., Reyes Torres, I., Jones, J., et al. (2018). Macrophages orchestrate breast cancer early dissemination and metastasis. Nat. Commun. *9*, 21. https://doi.org/10.1038/s41467-017-02481-5.
10. Ye, J., Baer, J.M., Faget, D.V., Morikis, V.A., Ren, Q., Melam, A., Delgado, A.P., Luo, X., Bagchi, S.M., Belle, J.I., et al. (2024). Senescent CAFs Mediate Immunosuppression and Drive Breast Cancer Progression. Cancer Discov. *14*, 1302–1323. https://doi.org/10.1158/2159-8290.Cd-23-0426.
11. Hu, M., Yao, J., Carroll, D.K., Weremowicz, S., Chen, H., Carrasco, D., Richardson, A., Violette, S., Nikolskaya, T., Nikolsky, Y., et al. (2008). Regulation of in situ to invasive breast carcinoma transition. Cancer Cell *13*, 394–406. https://doi.org/10.1016/j.ccr.2008.03.007.
12. Risom, T., Glass, D.R., Averbukh, I., Liu, C.C., Baranski, A., Kagel, A., McCaffrey, E.F., Greenwald, N.F., Rivero-Gutiérrez, B., Strand, S.H., et al. (2022). Transition to invasive breast cancer is associated with progressive changes in the structure and composition of tumor stroma. Cell *185*, 299–310.e18. https://doi.org/10.1016/j.cell.2021.12.023.
13. Xiong, S., Dong, L., and Cheng, L. (2021). Neutrophils in cancer carcinogenesis and metastasis. J. Hematol. Oncol. *14*, 173. https://doi.org/10.1186/s13045-021-01187-y.
14. Cools-Lartigue, J., Spicer, J., McDonald, B., Gowing, S., Chow, S., Giannias, B., Bourdeau, F., Kubes, P., and Ferri, L. (2013). Neutrophil extracellular traps sequester circulating tumor cells and promote metastasis. J. Clin. Investig. *123*, 3446–3458. https://doi.org/10.1172/JCI67484.
15. Hajizadeh, F., Aghebati Maleki, L., Alexander, M., Mikhailova, M.V., Masjedi, A., Ahmadpour, M., Hashemi, V., and Jadidi-Niaragh, F. (2021). Tumor-associated neutrophils as new players in immunosuppressive process of the tumor microenvironment in breast cancer. Life Sci. *264*, 118699. https://doi.org/10.1016/j.lfs.2020.118699.
16. Geng, S.-K., Fu, S.-M., Ma, S.-H., Fu, Y.-P., and Zhang, H.-W. (2021). Tumor infiltrating neutrophil might play a major role in predicting the clinical outcome of breast cancer patients treated with neoadjuvant chemotherapy. BMC Cancer *21*, 68. https://doi.org/10.1186/s12885-021-07789-6.
17. Zakurdaev, E.I., Bagateliya, Z.A., Titov, K.S., Elkhouli, E., Chizhikov, N.P., and Kharina, D.V. (2025). The Role of Tumor-Associated Neutrophils in Early Luminal HER2-Negative Breast Cancer Progression. Asian Pac. J. Cancer Prev. APJCP *26*, 207–213. https://doi.org/10.31557/APJCP.2025.26.1.207.
18. Zhang, H., Qin, Y., Wei, H., and Yu, C.-Y. (2026). Neutrophils: A Double-Edged Sword for Cancer Immunotherapy. ACS Nano Med. *1*, 31–53. https://doi.org/10.1021/acsnanomed.5c00003.

19. Uribe-Querol, E., and Rosales, C. (2015). Neutrophils in Cancer: Two Sides of the Same Coin. J. Immunol. Res. *2015*, 983698. https://doi.org/10.1155/2015/983698.

20. Gil Del Alcazar, C.R., Huh, S.J., Ekram, M.B., Trinh, A., Liu, L.L., Beca, F., Zi, X., Kwak, M., Bergholtz, H., Su, Y., et al. (2017). Immune Escape in Breast Cancer During In Situ to Invasive Carcinoma Transition. Cancer Discov. *7*, 1098–1115. https://doi.org/10.1158/2159-8290.Cd-17-0222.

21. Rao, T., Ranger, J.J., Smith, H.W., Lam, S.H., Chodosh, L., and Muller, W.J. (2014). Inducible and coupled expression of the polyomavirus middle T antigen and Cre recombinase in transgenic mice: an in vivo model for synthetic viability in mammary tumour progression. Breast Cancer Res. *16*, R11–R14. https://doi.org/10.1186/bcr3603.

22. Attalla, S., Taifour, T., Bui, T., and Muller, W. (2021). Insights from transgenic mouse models of PyMT-induced breast cancer: recapitulating human breast cancer progression in vivo. Oncogene *40*, 475–491. https://doi.org/10.1038/s41388-020-01560-0.

23. Lin, E.Y., Jones, J.G., Li, P., Zhu, L., Whitney, K.D., Muller, W.J., and Pollard, J.W. (2003). Progression to malignancy in the polyoma middle T oncoprotein mouse breast cancer model provides a reliable model for human diseases. Am. J. Pathol. *163*, 2113–2126. https://doi.org/10.1016/s0002-9440(10)63568-7.

24. Taifour, T., Attalla, S.S., Zuo, D., Gu, Y., Sanguin-Gendreau, V., Proud, H., Solymoss, E., Bui, T., Kuasne, H., Papavasiliou, V., et al. (2023). The tumor-derived cytokine Chi3l1 induces neutrophil extracellular traps that promote T cell exclusion in triple-negative breast cancer. Immunity *56*, 2755–2772.e8. https://doi.org/10.1016/j.immuni.2023.11.002.

25. Taifour, T., Massé, A., Gu, Y., Sanguin-Gendreau, V., Zuo, D., Xiao, B., Solymoss, E., Shen, Y., Proud, H., Attalla, S.S., et al. (2026). The CHI3L1-Neutrophil Axis Drives Immune Suppression and Breast Cancer Metastatic Dissemination. JCI Insight *11*, e199307. https://doi.org/10.1172/jci.insight.199307.

26. Maroulakou, I.G., Anver, M., Garrett, L., and Green, J.E. (1994). Prostate and mammary adenocarcinoma in transgenic mice carrying a rat C3(1) simian virus 40 large tumor antigen fusion gene. Proc. Natl. Acad. Sci. USA *91*, 11236–11240. https://doi.org/10.1073/pnas.91.23.11236.

27. Usary, J., Darr, D.B., Pfefferle, A.D., and Perou, C.M. (2016). Overview of Genetically Engineered Mouse Models of Distinct Breast Cancer Subtypes. Curr. Protoc. Pharmacol. *72*, 14.38.1-14.38.11. https://doi.org/10.1002/0471141755.ph1438s72.

28. Johnson, J.L., Ramadass, M., He, J., Brown, S.J., Zhang, J., Abgaryan, L., Biris, N., Gavathiotis, E., Rosen, H., and Catz, S.D. (2016). Identification of Neutrophil Exocytosis Inhibitors (Nexinhibs), Small Molecule Inhibitors of Neutrophil Exocytosis and Inflammation: DRUGGABILITY OF THE SMALL GTPase Rab27a. J. Biol. Chem. *291*, 25965–25982. https://doi.org/10.1074/jbc.M116.741884.

29. De Meo, M.L., and Spicer, J.D. (2022). The Role of Neutrophil Extracellular Traps in Cancer Progression and Metastasis (Elsevier), pp. 101595.

30. Zeng, W., Song, Y., Wang, R., He, R., and Wang, T. (2023). Neutrophil elastase: From mechanisms to therapeutic potential. J. Pharm. Anal. *13*, 355–366. https://doi.org/10.1016/j.jpha.2022.12.003.

31. Jia, W., Mao, Y., Luo, Q., Wu, J., and Guan, Q. (2024). Targeting neutrophil elastase is a promising direction for future cancer treatment. Discov. Oncol. *15*, 167. https://doi.org/10.1007/s12672-024-01010-3.

32. Fujie, K., Shinguh, Y., Inamura, N., Yasumitsu, R., Okamoto, M., and Okuhara, M. (1999). Release of neutrophil elastase and its role in tissue injury in acute inflammation: effect of the elastase inhibitor, FR134043. Eur. J. Pharmacol. *374*, 117–125. https://doi.org/10.1016/S0014-2999(99)00268-X.

33. Foekens, J.A., Ries, C., Look, M.P., Gippner-Steppert, C., Klijn, J.G.M., and Jochum, M. (2003). The prognostic value of polymorphonuclear leukocyte elastase in patients with primary breast cancer. Cancer Res. *63*, 337–341.

34. Stevens, T., Ekholm, K., Gränse, M., Lindahl, M., Kozma, V., Jungar, C., Ottosson, T., Falk-Håkansson, H., Churg, A., Wright, J.L., et al. (2011). AZD9668: pharmacological characterization of a novel oral inhibitor of neutrophil elastase. J. Pharmacol. Exp. Therapeut. *339*, 313–320. https://doi.org/10.1124/jpet.111.182139.

35. Lulla, A.R., Akli, S., Karakas, C., Caruso, J.A., Warma, L.D., Fowlkes, N.W., Rao, X., Wang, J., Hunt, K.K., Watowich, S.S., and Keyomarsi, K. (2024). Neutrophil Elastase Remodels Mammary Tumors to Facilitate Lung Metastasis. Mol. Cancer Therapeut. *23*, 492–506. https://doi.org/10.1158/1535-7163.Mct-23-0414.

36. Russell, T.D., Jindal, S., Agunbiade, S., Gao, D., Troxell, M., Borges, V.F., and Schedin, P. (2015). Myoepithelial cell differentiation markers in ductal carcinoma in situ progression. Am. J. Pathol. *185*, 3076–3089. https://doi.org/10.1016/j.ajpath.2015.07.004.

37. Mitchell, E., Jindal, S., Chan, T., Narasimhan, J., Sivagnanam, S., Gray, E., Chang, Y.H., Weinmann, S., and Schedin, P. (2020). Loss of myoepithelial calponin-1 characterizes high-risk ductal carcinoma in situ cases, which are further stratified by T cell composition. Mol. Carcinog. *59*, 701–712. https://doi.org/10.1002/mc.23171.

38. Adriance, M.C., Inman, J.L., Petersen, O.W., and Bissell, M.J. (2005). Myoepithelial cells: good fences make good neighbors. Breast Cancer Res. *7*, 190–197. https://doi.org/10.1186/bcr1286.

39. Pang, X., He, X., Qiu, Z., Zhang, H., Xie, R., Liu, Z., Gu, Y., Zhao, N., Xiang, Q., and Cui, Y. (2023). Targeting integrin pathways: mechanisms and advances in therapy. Signal Transduct. Targeted Ther. *8*, 1. https://doi.org/10.1038/s41392-022-01259-6.

40. Vallmajo-Martin, Q., Ma, Z., Srinivasan, S., Murali, D., Dravis, C., Mukund, K., Subramaniam, S., Wahl, G.M., and Lytle, N.K. (2024). The molecular chronology of mammary epithelial cell fate switching. Preprint at bioRxiv. https://doi.org/10.1101/2024.10.08.617155.

41. Sflomos, G., Dormoy, V., Metsalu, T., Jeitziner, R., Battista, L., Scabia, V., Raffoul, W., Delaloye, J.-F., Treboux, A., Fiche, M., et al. (2016). A Preclinical Model for ERα-Positive Breast Cancer Points to the Epithelial Microenvironment as Determinant of Luminal Phenotype and Hormone Response. Cancer Cell *29*, 407–422. https://doi.org/10.1016/j.ccell.2016.02.002.

42. Hutten, S.J., de Bruijn, R., Lutz, C., Badoux, M., Eijkman, T., Chao, X., Ciwinska, M., Sheinman, M., Messal, H., Herencia-Ropero, A., et al. (2023). A living biobank of patient-derived ductal carcinoma in situ mouse-intraductal xenografts identifies risk factors for invasive progression. Cancer Cell *41*, 986–1002.e9. https://doi.org/10.1016/j.ccell.2023.04.002.

43. Debeljak, M., Cho, S., Downs, B.M., Considine, M., Avin-McKelvey, B., Wang, Y., Perez, P.N., Grizzle, W.E., Hoadley, K.A., Lynch, C.F., et al. (2024). Multimodal genome-wide survey of progressing and non-progressing breast ductal carcinoma in-situ. Breast Cancer Res. *26*, 178. https://doi.org/10.1186/s13058-024-01927-1.

44. Toussaint, J., Durbecq, V., Altintas, S., Doriath, V., Rouas, G., Paesmans, M., Bedard, P., Haibe-Kains, B., Tjalma, W.A., Larsimont, D., et al. (2010). Low CD10 mRNA expression identifies high-risk ductal carcinoma in situ (DCIS). PLoS One *5*, e12100. https://doi.org/10.1371/journal.pone.0012100.

45. Hsiao, Y.H., Tsai, H.D., Chou, M.C., and Man, Y.G. (2011). The myoepithelial cell layer may serve as a potential trigger factor for different outcomes of stage-matched invasive lobular and ductal breast cancers. Int. J. Biol. Sci. *7*, 147–153. https://doi.org/10.7150/ijbs.7.147.

46. Bui, T.M., Jimenez, E.R., Li, Z., Foidart, P., Puleo, J., Yan, P., Jhaveri, A., Yang, L., Nishida, J., Seehawer, M., et al. (2026). Identification of cycling regulatory T cell precursors as conductors of immune escape during breast carcinoma progression. Cancer Cell *44*, 1179–1200.e14. https://doi.org/10.1016/j.ccell.2026.03.015.

47. Göransson, S., Chen, S., Olofsson, H., Larsson, O., and Strömblad, S. (2023). An extracellular matrix stiffness-induced breast cancer cell transcriptome resembles the transition from ductal carcinoma in situ (DCIS) to invasive ductal carcinoma (IDC). Biochem. Biophys. Res. Commun. *654*, 73–79. https://doi.org/10.1016/j.bbrc.2023.03.001.

48. Trinh, A., Gil Del Alcazar, C.R., Shukla, S.A., Chin, K., Chang, Y.H., Thibault, G., Eng, J., Jovanović, B., Aldaz, C.M., Park, S.Y., et al. (2021). Genomic Alterations during the In Situ to Invasive Ductal Breast Carcinoma Transition Shaped by the Immune System. Mol. Cancer Res. *19*, 623–635. https://doi.org/10.1158/1541-7786.Mcr-20-0949.

49. Gil Del Alcazar, C.R., Trinh, A., Alečković, M., Rojas Jimenez, E., Harper, N.W., Oliphant, M.U.J., Xie, S., Krop, E.D., Lulseged, B., Murphy, K.C., et al. (2022). Insights into Immune Escape During Tumor Evolution and Response to Immunotherapy Using a Rat Model of Breast Cancer. Cancer Immunol. Res. *10*, 680–697. https://doi.org/10.1158/2326-6066.Cir-21-0804.

50. Campbell, M.J., Baehner, F., O'Meara, T., Ojukwu, E., Han, B., Mukhtar, R., Tandon, V., Endicott, M., Zhu, Z., Wong, J., et al. (2017). Characterizing the immune microenvironment in high-risk ductal carcinoma in situ of the breast. Breast Cancer Res. Treat. *161*, 17–28. https://doi.org/10.1007/s10549-016-4036-0.

51. Darvishian, F., Wu, Y., Ozerdem, U., Chun, J., Adams, S., Guth, A., Axelrod, D., Shapiro, R., Troxel, A.B., Schnabel, F., and Roses, D. (2022). Macrophage density is an adverse prognosticator for ipsilateral recurrence in ductal carcinoma in situ. Breast *64*, 35–40. https://doi.org/10.1016/j.breast.2022.04.004.

52. Nelson, A.C., Machado, H.L., and Schwertfeger, K.L. (2018). Breaking through to the Other Side: Microenvironment Contributions to DCIS Initiation and Progression. J. Mammary Gland Biol. Neoplasia *23*, 207–221. https://doi.org/10.1007/s10911-018-9409-z.

53. Sheng, Y., Peng, W., Huang, Y., Cheng, L., Meng, Y., Kwantwi, L.B., Yang, J., Xu, J., Xiao, H., Kzhyshkowska, J., and Wu, Q. (2023). Tumor-activated neutrophils promote metastasis in breast cancer via the G-CSF-RLN2-MMP-9 axis. J. Leukoc. Biol. *113*, 383–399. https://doi.org/10.1093/jleuko/qiad004.

54. Wells, J.M., Titlestad, I.L., Tanash, H., Turner, A.M., Chapman, K.R., Hatipoğlu, U.Ş., Goldklang, M.P., D'Armiento, J.M., Pirozzi, C.S., Drummond, M.B., et al. (2025). Two randomised controlled phase 2 studies of the oral neutrophil elastase inhibitor alvelestat in alpha-1 antitrypsin deficiency. Eur. Respir. J. *66*, 2501019. https://doi.org/10.1183/13993003.01019-2025.

55. Vogelmeier, C., Aquino, T.O., O'Brien, C.D., Perrett, J., and Gunawardena, K.A. (2012). A randomised, placebo-controlled, dose-finding study of AZD9668, an oral inhibitor of neutrophil elastase, in patients with chronic obstructive pulmonary disease treated with tiotropium. COPD J. Chronic Obstr. Pulm. Dis. *9*, 111–120. https://doi.org/10.3109/15412555.2011.641803.

56. Quail, D.F., Amulic, B., Aziz, M., Barnes, B.J., Eruslanov, E., Fridlender, Z.G., Goodridge, H.S., Granot, Z., Hidalgo, A., Huttenlocher, A., et al. (2022). Neutrophil phenotypes and functions in cancer: A consensus statement. J. Exp. Med. *219*, e20220011. https://doi.org/10.1084/jem.20220011.

57. Fetit, R., McLaren, A.S., White, M., Mills, M.L., Falconer, J., Cortes-Lavaud, X., Gilroy, K., Lannagan, T.R.M., Ridgway, R.A., Nixon, C., et al. (2024). Characterizing Neutrophil Subtypes in Cancer Using scRNA Sequencing Demonstrates the Importance of IL1β/CXCR2 Axis in Generation of Metastasis-specific Neutrophils. Cancer Res. Commun. *4*, 588–606. https://doi.org/10.1158/2767-9764.Crc-23-0319.

58. Hatje, K., Schneider, K., Danilin, S., Koechl, F., Giroud, N., Juglair, L., Marbach, D., Knuckles, P., Bergauer, T., Metruccio, M., et al. (2025). Comparison of single-cell RNA-seq methods to enable transcriptome profiling of neutrophils in clinical samples. Cell Rep. Methods *5*, 101173. https://doi.org/10.1016/j.crmeth.2025.101173.

59. Garratt, L.W. (2021). Current Understanding of the Neutrophil Transcriptome in Health and Disease. Cells *10*, 2406. https://doi.org/10.3390/cells10092406.

60. Pham, C.T.N. (2006). Neutrophil serine proteases: specific regulators of inflammation. Nat. Rev. Immunol. *6*, 541–550. https://doi.org/10.1038/nri1841.

61. Huck, L., Pontier, S.M., Zuo, D.M., and Muller, W.J. (2010). beta1-integrin is dispensable for the induction of ErbB2 mammary tumors but plays a critical role in the metastatic phase of tumor progression. Proc. Natl. Acad. Sci. USA *107*, 15559–15564. https://doi.org/10.1073/pnas.1003034107.

62. Hsu, B.E., Tabariès, S., Johnson, R.M., Andrzejewski, S., Senecal, J., Lehuédé, C., Annis, M.G., Ma, E.H., Völs, S., Ramsay, L., et al. (2019). Immature Low-Density Neutrophils Exhibit Metabolic Flexibility that Facilitates Breast Cancer Liver Metastasis. Cell Rep. *27*, 3902–3915.e6. https://doi.org/10.1016/j.celrep.2019.05.091.

63. Gunther, E.J., Belka, G.K., Wertheim, G.B.W., Wang, J., Hartman, J.L., Boxer, R.B., and Chodosh, L.A. (2002). A novel doxycycline-inducible system for the transgenic analysis of mammary gland biology. FASEB J. *16*, 283–292. https://doi.org/10.1096/fj.01-0551com.

64. Fahad Ullah, M. (2019). Breast Cancer: Current Perspectives on the Disease Status. Adv. Exp. Med. Biol. *1152*, 51–64. https://doi.org/10.1007/978-3-030-20301-6_4.

65. Ly, D., Forman, D., Ferlay, J., Brinton, L.A., and Cook, M.B. (2013). An international comparison of male and female breast cancer incidence rates. Int. J. Cancer *132*, 1918–1926. https://doi.org/10.1002/ijc.27841.

66. Tabariès, S., Dong, Z., Annis, M.G., Omeroglu, A., Pepin, F., Ouellet, V., Russo, C., Hassanain, M., Metrakos, P., Diaz, Z., et al. (2011). Claudin-2 is selectively enriched in and promotes the formation of breast cancer liver metastases through engagement of integrin complexes. Oncogene *30*, 1318–1328. https://doi.org/10.1038/onc.2010.518.

67. Gu, Y., Taifour, T., Bui, T., Zuo, D., Pacis, A., Poirier, A., Attalla, S., Fortier, A.-M., Sanguin-Gendreau, V., Pan, T.-C., et al. (2024). Osteopontin is a therapeutic target that drives breast cancer recurrence. Nat. Commun. *15*, 9174. https://doi.org/10.1038/s41467-024-53023-9.

68. Gu, Y., Zuo, D., Hu, Q.X., Sanguin-Gendreau, V., Pacis, A., Guiot, M.C., Chang, A.C.C., Taifour, T., Ling, C., Lee, A.V., et al. (2026). Activating mutations in ESR1 contribute to an immunosuppressive breast tumor microenvironment by dampening cytokine secretion. JCI Insight *11*, e199927. https://doi.org/10.1172/jci.insight.199927.

## STAR★METHODS

### KEY RESOURCES TABLE

| REAGENT or RESOURCE | SOURCE | IDENTIFIER |
|---|---|---|
| Antibodies | | |
| Rat IgG2a, 2A3 | Bio X cell | Cat# BE0089; RRID: AB_1107769 |
| Anti-Ly6G, 1A8 | Bio X cell | Cat# BE0075-1; RRID: AB_1107721 |
| Chi3l1 | Invitrogen | Cat# PA5-81356; RRID: AB_2788571 |
| CitH3 (western blot) | Abcam | Cat# Ab5103; RRID: AB_304752 |
| TSP1 | Cell Signaling Technology | Cat# 37879S |
| β -actin | Sigma Aldrich | Cat# A5441; RRID: AB_476744 |
| Vinculin | Millipore | Cat# MAB3574; RRID: AB_2304338 |
| α-tubulin | Cell signaling | Cat# 3873; RRID: AB_1904178 |
| CK14 | Ventana | Cat# 760-4805; RRID: AB_2935819 |
| CK5 | Biolegend | Cat# 905504; RRID: AB_3095835 |
| Calponin 1 | Cell Signaling Technology | Cat# 17819; RRID: AB_2798789 |
| P63 | Santa Cruz | Cat# sc25268; RRID: AB_628092 |
| α-SMA | DAKO | Cat# M0851; RRID: AB_2313736 |
| Ly6G | Cell Signaling Technology | Cat#87048; RRID: AB_2909808 |
| MPO | Abcam | Cat# ab208670; RRID: AB_2864724 |
| CitH3 (Staining) | Cell Signaling Technology | Cat# 26111 |
| Mouse NE | Cell Signaling Technology | Cat# 90120; RRID: AB_3096401 |
| Human NE | Cell Signaling Technology | Cat# 28162 |
| Laminin | Abcam | Cat# ab11575; RRID: AB_298179 |
| Collagen IV | Abcam | Cat# ab6586; RRID: AB_305584 |
| Elastin | BIOS USA | Cat# bs-1756R; RRID: AB_10856940 |
| Cleaved caspase 3 | Cell Signaling Technology | Cat# 9661; RRID:AB_2341188 |
| *p*-FAK | Invitrogen | Cat# 44-624G |
| CK8 | Biolegend | Cat# 904804; RRID: AB_2616821 |
| CD3 | Abcam | Cat# ab16669; RRID: AB_443425 |
| CD8 | Cell Signaling Technology | Cat# 98941; RRID: AB_2756376 |
| CD4 | Cell Signaling Technology | Cat# 25229; RRID: AB_2798898 |
| PD1 | Cell Signaling Technology | Cat# 84651; RRID: AB_2800041 |
| Granzyme B | Cell Signaling Technology | Cat# 44153; RRID: AB_2857976 |
| Ki67 | Cell Signaling Technology | Cat# 12202; RRID: AB_2620142 |
| PCNA | Cell Signaling Technology | Cat# 2586; RRID: AB_2160343 |
| Pan-ck | Ventana | Cat# 760-2595; RRID: AB_2941938 |
| CD45 | BD Bioscience | Cat# 564225; RRID: AB_2716861 |
| CD11b | Biolegend | Cat# 101201; RRID: AB_312784 |
| Ly6G | Biolegend | Cat# 127606; RRID: AB_1236494 |
| F4/80 | Biolegend | Cat# 123114; RRID: AB_893478 |
| Epcam | Biolegend | Cat# 118233; RRID: AB_2632775 |
| CD49f | Biolegend | Cat# 313622; RRID: AB_2561705 |
| CK5 (FACS) | Santa Cruz | Cat# sc-32721; RRID: AB_627852 |
| CK14 (FACS) | Santa Cruz | Cat# sc-53253; RRID: AB_2134820 |
| Viability dye eFluorYM 506 | ThermoFisher/eBioscience | Cat# 65-0866-14 |
| ImmPRESS HRP Anti-Mouse IgG Polymer detection kit | Vector Laboratories | Cat# VECTMP745250 |

(*Continued on next page*)

| *Continued* | | |
|---|---|---|
| REAGENT or RESOURCE | SOURCE | IDENTIFIER |
| ImmPRESS HRP Anti-Rabbit IgG Polymer detection kit | Vector Laboratories | Cat# VECTMP740150 |
| Goat anti-rabbit secondary antibody Alexa Fluor 555 | Invitrogen | Cat# A21428 |
| Alexa Fluor plus 555 phalloidin | Invitrogen | Cat# A30106 |
| Biological samples | | |
| Fetal Bovine serum (FBS) | Wisent Inc. | Cat#080-150 |
| Bovine pituitary extract | Hammond Cell Tech | Cat#1078-NZ |
| Breast hyperplasia tissue array | Tissuearray.com (USBiomax) | Cat#BR806 |
| Breast cancer with matched breast tissue array | Tissuearray.com (USBiomax) | Cat# BR251 |
| Advanced stage breast cancer and normal breast tissue array | Tissuearray.com (USBiomax) | Cat#BR1191 |
| Multiple breast disease with NAT breast tissue array | Tissuearray.com (USBiomax) | Cat#1503 |
| Breast cancer tissue array with adjacent normal breast tissue | Tissuearray.com (USBiomax) | Cat#BR8011 |
| Breast tumor tissue array | Tissuearray.com (USBiomax) | Cat#20811 |
| Mid-advanced stage breast cancer and normal tissue array | Tissuearray.com (USBiomax) | Cat#8013 |
| Breast cancer tissue array (mostly ductal carcinoma *in situ*, DCIS) | Tissuearray.com (USBiomax) | Cat#301 |
| Chemicals, peptides, and recombinant proteins | | |
| doxycycline | Wisent Inc. | Cat# 450-185 |
| GSK484 PAD4i | Medchem express | Cat# HY-100514 |
| DNAse I | Roche | Cat#4536282001 |
| Nexinhib20 | Medchem express | Cat# HY-125792 |
| Alvelestat | Medchem express | Cat# HY-15651 |
| Hydrosortisone | Sigma | Cat#H4001 |
| Recombinant human EGF | Wisent Inc. | Cat#511-110-CU |
| Recombinant human insulin | Wisent Inc. | Cat#511-016-UG |
| Recombinant mouse Chi3l1 | R&D | Cat#2649-CH-050 |
| Recombinant mouse Cathpesin G | Biomatik | Cat#RPU51859 |
| Recombinant mouse PR3 | Biomatik | Cat#RPU43519 |
| Recombinant mouse NE | R&D | Cat#4517-SE |
| Cultrex Growth Factor Basement Membrane Extract | R&D | Cat# 3433-010-01 |
| DQ collagen, type IV from human placenta, fluorescein conjugate | Invitrogen | Cat# D12052 |
| Geltrex | Geltrex | Cat#A1413202 |
| Critical commercial assays | R&D system | Cat# 4517-SE |
| Incucyte Cytotox 488 reagent | Sartorius | Cat#4633 |
| DRAQ5 (Thermofisher#) | Thermofisher | Cat#62251 |
| Tyramide signal amplification substrates_ OPAL480 | Akoya biosciences | Cat#OP-001000 |
| Tyramide signal amplification substrates_ OPAL520 | Akoya biosciences | Cat# OP-001001 |
| Tyramide signal amplification substrates_ OPAL570 | Akoya biosciences | Cat#OP-001003 |
| Tyramide signal amplification substrates_ OPAL620 | Akoya biosciences | Cat#OP-001004 |

(*Continued on next page*)

| *Continued* | | |
|---|---|---|
| REAGENT or RESOURCE | SOURCE | IDENTIFIER |
| Tyramide signal amplification substrates_ OPAL690 | Akoya biosciences | Cat#OP-001006 |
| Tyramide signal amplification substrates_ OPAL780 | Akoya biosciences | Cat#OP-001008 |
| RNAscope 2.5 HD Assay-RED kit | Advanced Cell Diagnostics | Cat#322360 |
| 10× Casein Solution-Blocking buffer | Vector Laboratories | Cat#SP-5020-250 |
| Antigen Unmasking Solution, Citrate-Based PH6 | Vector Laboratories | Cat#H-3300-250 |
| Antigen Unmasking Solution, Tris-Based PH9 | Vector Laboratories | Cat#H-3301-250 |
| BD Cytofix/Cytoperm fixation/permeabilization | BD Bioscience | Cat#554714 |
| NE ELISA | R&D | Cat# DY4517-05 |
| MPO ELISA | R&D | Cat# DY3667 |
| MycoAlert Kit | Lonza | Cat# LT07-118 |
| Deposited data | | |
| Published human DCIS RNAseq | Debeljak et al.[43] | GEO: GSE281303 |
| Experimental models: Cell lines | | |
| Mouse: MMTV-PyMT | Huck et al.[61] | N/A |
| Mouse: Primary neutrophils | Hsu et al.[62] | N/A |
| Mouse organoids: MIC CK14-TdTomato | This paper | N/A |
| Mouse; 4T1 | Hsu et al.[62] | N/A |
| Human: MCF-7 | ATCC | Cat# HTB-22 |
| Experimental models: Organisms/strains | | |
| Mouse: MMTV-rtTA (MTB) | Gunther et al.[63] | N/A |
| Mouse: TetON-MIC | Rao et al.[21] | N/A |
| Mouse: Chi3l1$^{-/-}$ | Taifour et al.[24,25] | N/A |
| Mouse: Chi3l1 OE | Taifour et al.[24,25] | N/A |
| Mouse: C3Tag | Jackson lab | Cat#013591 |
| Mouse: CK14-TdTomato | Vallmajo-Martin et al.[40] | N/A |
| Mouse: NOD-scid IL2Rgammanull (NSG) | Jackson lab | Cat#005557 |
| Mouse: Balb/c | Jackson lab | Cat#000651 |
| Oligonucleotides | | |
| Chi3l1 RNAscope probe | Advanced Cell Diagnostics | Cat#449621 |
| Software and algorithms | | |
| HALO | Indica lab | https://www.indicalab.com/halo/ |
| Prism | Graphpad | https://www.graphpad.com/scientific-software/prism/ |
| Li-Cor Odyssey 3.0 | Li-Cor | https://www.licor.com/bio/ |
| Image Studio software | Li-Cor | https://www.licor.com/bio/image-studio/ |
| Flowjo 10.6.2 | Treestar | https://www.flowjo.com/ |
| BioRender | BioRender | https://www.BioRender.com |
| Kaplan Meier plotter | Kaplan Meier plotter | https://kmplot.com/analysis/ |

## EXPERIMENTAL MODEL AND STUDY PARTICIPANT DETAILS

### Animals-mouse models

All procedures and experiments described in this study were performed according to the guidelines of the Canadian Council of Animal Care and approved by the Animal Care Committee of McGill University (Animal Use Protocol #MCGL5518). All animals were maintained in the Goodman Cancer Institute's SPF facility. The MTB, MIC, germline *Chi3l1*$^{-/-}$, Chi3l1 OE and MMTV-PyMT *NE*$^{-/-}$ strains have been characterized and described previously.[21,24,25,35,63] The MTB strain was generously provided by Dr. Lewis Chodosh.[63] Briefly, the MTB transgene (MMTV-rtTA) utilizes the mammary gland specific promoter (MMTV) to drive expression of reverse tetracycline transactivator (rtTA) specifically in the mammary epithelial cells. The MIC transgene couples expression of

the PyMT oncogene and Cre recombinase through an Internal Ribosomal Entry Site (IRES) element under the control of the TetO promoter.[21,22] Doxycycline induction thus leads to expression of the PyMT oncogene as well as Cre recombinase in the mammary epithelium (Figure S2A). PyMT tumors are driven by activation of PI3K/AKT, Src and MAPK signaling pathways.[22] In the Chi3l1 OE model, doxycycline induction leads to expression of Chi3l1 specifically from mammary epithelial cells. The MTB MIC *Chi3l1*$^{-/-}$ as well as MTB MIC Chi3l1 OE crosses were described previously.[24,25] Chi3l1 ablation or OE was confirmed through RNA FISH. The C3Tag GEMM was purchased from Jackson lab (Strain # 013591) and has been characterized previously.[26] MTB MIC mice were crossed with CK14-TdTomato mice to generate organoid culture system where the CK14 myoepithelial layer fluoresces in red. The CK14-TdTomato was described previously[40] and it was generously provided by Dr. Geoffrey M. Wahl. Formalin fixed paraffin-embedded (FFPE) tissue from MMTV-PyMT *NE*$^{-/-}$ breast tumors were provided by Dr. Khandan Keyomarsi.[35]

All GEMM experimental animals were maintained on a uniform FVB/N background. BALB/c mice used for the 4T1 experiments were obtained from Jackson Laboratory (Cat. #000651). All experiments utilized adult virgin female mice. Adult MIC-based experimental animals were induced with doxycycline (2 mg/mL, Wisent, cat# 450–185) in their drinking water at 8 weeks of age. Mice on doxycycline were monitored for tumor formation through weekly physical palpations and caliper measurements. Mice were sacrificed at pre-determined timepoints (2 or 6-week post induction) or when they reached the ethical humane endpoint defined by the animal care guidelines as a single tumor of 2.5 $cm^3$ or multiple tumors per mouse totaling 6.0 $cm^3$. Each animal experiment was performed using an independent cohort of mice with its own corresponding control group. Animals and tissue samples were not reused across experiments, and all comparisons were made only between experimental groups and their matched litter-mate controls within the same cohort. C3Tag mice were monitored for tumor formation and sacrificed at tumor endpoint or at a predetermined time point (16 weeks of age), when their mammary glands develop DCIS lesions.

Breast cancer is the most common type of cancer affecting women worldwide.[64] Less than 1% of cases occur among men.[65] Because women are at a greater risk of developing breast cancer, we use female GEMMs in our research to accurately reflect the epidemiology of the disease. Thus, all experiments described in this study used female mice. Accordingly, the findings may not be generalizable to male breast cancer.

### Human participants

DCIS and IDC patient samples were collected at the time of biopsy or surgery at the McGill University Health Center (MUHC) after informed written consent, in accordance with institutional ethics approval (MUHC REB protocols SDR-99–780 and SDR-00–966). All DCIS samples were from female patients who were therapy-naïve at the time of surgery (mean age of 82). DCIS diagnoses were confirmed by a pathologist, and only cases classified as pure DCIS with negative lymph node status at diagnosis were included in the study. All DCIS samples (*n=9*) were clinically documented to be ER + PR + grade II or III. FFPE tissue sections were used for immunofluorescent analyses. All DCIS samples were included and they were not allocated into different experimental groups.

A retrospective clinical cohort consisting of metastatic (*n=12*) and non-metastatic (*n=21*) breast cancer samples was used to assess relative levels of NE. The cohort consisted predominantly of HER2-positive grade II–III invasive ductal carcinomas. Clinicopathological characteristics, including age, hormone receptor status, HER2 status, histological grade, and tumor size, were similar between metastatic and non-metastatic groups (Table S1). Samples were split into two groups: metastatic vs. non-metastatic based on clinical outcome data. For both the DCIS and metastasis cohorts: race, ethnicity, and ancestry were not systematically collected and therefore could not be evaluated. Because male breast cancer cases are rare,[65] all human tissue samples were derived from female patients. Therefore, the potential influence of sex on the observed findings could not be evaluated, and the findings may not be generalizable to male breast cancer.

Human normal (*n=9*), DCIS (*n=75*) and IDC (*n= 207*) breast tissue microarrays (BR806, BR251, BR1191, BR1503, BR8011, BR20811, BR8013 and BR301) were purchased from TissueArray.Com (formerly US Biomax Inc). The TMA included samples from female individuals with normal breast tissue, DCIS, or IDC. Samples were allocated to groups according to the diagnosis provided by the supplier. Patient information and diagnosis are available at https://www.tissuearray.com/

### Primary cell culture and cell lines

The PyMT+ cell line was established from MMTV-PyMT primary mammary tumors as described previously.[61] Cells were maintained in DMEM (Wisent, 219–010) media that contained 10% Fetal Bovine Serum (FBS), 5 ng/mL Epidermal Growth Factor (EGF), 35 μg/mL bovine pituitary extract, 5 μg/mL Insulin, 1 μg/mL Hydrocortisone, 5% penicillin/streptomycin and 5% amphotericin. The cells were incubated in a 5% CO2, 37°C incubator at all times. PyMT-derived cell lines were generated in-house from PyMT mammary tumors, and their identity was confirmed based on the expected PyMT genotype and phenotype. All cells were confirmed to be mycoplasma-free using the MycoAlert Kit (Lonza, LT07-118).

In order to extract murine neutrophils, 50K cells of the liver metastatic 4T1 derivative (4T1-2776) were injected into the mammary fat pad of 7-week-old female Balb/c mice (Jackson lab, Cat#000651). The cell line was previously characterized[66] and generously provided by Dr. Peter Siegel. The cells exhibited the expected 4T1 cellular and tumor phenotype and were confirmed to be mycoplasma-free using the MycoAlert Kit (Lonza, LT07-118). 3 weeks post-injection, the blood was collected by cardiac puncture and extraction of primary mouse low density neutrophils (LDNs) was performed as previously described.[62] LDNs were cultured at 37°C, 5% CO2 in RPMI medium (Wisent, 350–000-CL) supplemented with 3% FBS, 5% penicillin/streptomycin and 5% amphotericin. In order to generate neutrophil conditioned media, freshly extracted LDNs were cultured in the presence of 10 uM Alvelestat

(HY-15651, Medchem express) or vehicle control (10% DMSO, 75% PBS, 15% Tween-80) at 37°C, 5% CO2 for 10 h. Conditioned media was then collected, centrifuged to remove cell debris and stored at -80°C until used for laminin and collagen IV degradation assays.

The human breast cancer MCF-7 cell line (ATCC, cat# HTB-22) was cultured in DMEM supplemented with 10% FBS and was used for the MIND model experiment. MCF7 cells were obtained from ATCC and authenticated by the supplier. All cells were confirmed to be mycoplasma free using the MycoAlert Kit (Lonza, LT07-118).

## METHOD DETAILS

### Pharmacological treatments

Neutrophil depletion in WT MTB MIC and Chi3l1 OE MTB MIC mice through anti-Ly6G treatment was described previously.[24,25] FFPE mammary gland tissue from these experiments was used in this study. Neutrophil depletion was previously confirmed through fluorescent activated cell sorting as well as immunofluorescent staining.[24,25] Similarly, Pad4i (GSK484, HY-100514, Medchem express) and DNAse 1 (4536282001, Roche) treatments in WT MTB MIC mice has been described and validated previously.[24]

In order to deplete neutrophils in WT C3Tag mice, mice were randomized to receive 400 µg of purified anti-Ly-6G rat antibody (1A8, BioXcell #BE0075-1) or IgG2a rat isotype control (2A3, Bio X cell #BE0089) twice per week via IP injections. Treatments started when mice reached 12 weeks of age and continued for 4 weeks (16 weeks of age). Neutrophil depletion was confirmed through FACS sorting on the blood for circulating neutrophils as well as through immunofluorescent staining on the mammary glands.

In order to inhibit neutrophil degranulation, cohorts of MTB MIC Chi3l1 OE mice were randomized to receive 30mg/kg Nexinhib20 (HY-125792, Medchem express) via intraperitoneal (IP) injections. Nexinhib20 was dissolved in PBS, DMSO Polyethylene glycol and tween-80. Treatment took place daily starting 5 days before doxycycline induction and continued for 2 weeks post induction. Control mice were given mock (Vehicle) injections at the same time and for the same duration. Immunofluorescent staining and ELISA analyses were undertaken to validate successful inhibition of degranulation without impacting overall neutrophil recruitment.

In order to inhibit NE, cohorts of WT MTB MIC and MTB MIC Chi3l1 OE mice were randomized to receive 50mg/kg of Alvelestat (HY-15651, Medchem express) via IP injections. Alvelestat was dissolved in PBS, DMSO, Polyethylene glycol and tween-80. Treatment took place daily starting 5 days before doxycycline induction and continued for 2 or 6 weeks post induction. Control mice were given mock (Vehicle) injections at the same time and for the same duration. In the second set of experiments, cohorts of MTB MIC Chi3l1 OE were induced for 2 weeks before starting daily IP injections with 50mg/kg of Alvelestat until 6 weeks of induction. Immunofluorescent staining and immunoblots for direct NE-degradation targets was performed to confirm successful inhibition of NE. Similarly, to inhibit NE in C3Tag mice, Cohorts of WT C3Tag mice were randomized to receive 50mg/kg of Alvelestat (HY-15651, Medchem express) or vehicle control via IP injections. Treatments started when mice reached 12 weeks of age and continued daily for 4 weeks (16 weeks of age). Control mice were given mock (Vehicle) injections at the same time and for the same duration.

In order to study the role of NE in established mammary tumors, cohorts of WT MTB MIC mice were induced with doxycycline and monitored for tumor formation (average tumor onset at 35 days post-induction). Once tumors were palpable (measured at 250mm$^3$), the mice were randomized to receive 50mg/kg of Alvelestat or vehicle control. Treatment took place daily until reaching humane endpoint (approximately 60 days post tumor onset). The mice were then sacrificed and the lungs were analyzed for metastatic burden.

### Mammary intraductal engraftment

The human breast cancer MCF-7 cell line (ATCC, cat# HTB-22) was cultured in DMEM supplemented with 10% FBS. 5 × 104 cells were suspended in 10 µL of PBS with 2 µL of trypan blue and injected intraductally into 4th mammary gland of 8- to 12-week-old NOD-scid IL2Rgammanull (NSG) female mice (Jackson lab, Cat# 005557) Mammary glands were collected at 4, 8, or 12 weeks post-injection and fixed overnight in Carnoy's Fixative. Tissues were embedded in paraffin and sectioned at 4 µm. Sections were first stained with H&E for initial tumor staging, then confirmed by immunofluorescent staining. Tissues with cancer cells confined to the ductal tree were classified as DCIS, as confirmed by the presence of myoepithelial cells on immunofluorescence (CK5+). Tissues with stromal infiltration of cancer cells were classified as invasive. All lesions were of high nuclear grade (prominent pleomorphism and nucleoli; large nuclei (>2.5 times the size of a normal ductal epithelial cell)).

### Human bioinformatic analysis

Kaplan–Meier curves depicting overall survival based on *ELANE* mRNA expression in breast cancer patients were generated using the web-based tool Kaplan Meier plotter (https://kmplot.com/analysis/). The analysis was for 2976 breast cancer patient sample, stratified according to median *ELANE* expression.

For *ELANE* expression in DCIS datasets, the GSE281303 RNAseq publicly available dataset[43] was interrogated for *ELANE* expression in DCIS progressors vs. non-progressors and analyzed using Wilcoxon rank-sum test.

### RNA FISH

RNAscope *in situ* hybridization was performed on paraffin-embedded mammary gland sections using RNAscope 2.5 HD Assay-RED kit (ACD, Cat#322360) according to the manufacturer's protocol. The following probes were used: mouse *Chi3l1* (Cat# 449621). This protocol was followed with fluorescent IHC for pan-ck as described below.

### Protein extraction and immunoblots

Flash-frozen mammary gland or breast tumor pieces were crushed in liquid nitrogen using a mortar and a pestle. RIPA buffer (10 mM Tris at PH 8, 1 mM EDTA, 0.5 mM EGTA, 1% Triton X-100, 0.1% sodium deoxycholate, 0.1% SDS, 140 mM NaCl) with protease inhibitor cocktail (1 μg/mL aprotinin, 1 μg/mL leupeptin, 1 mM sodium orthovanadate and 1 μg/mL phenylmethylsulfonyl fluoride) was added to lyse the cells. The samples were left to rotate at 4°C for a minimum of 60 min. Lysates were centrifuged at 13000 RPM for 10 min at 4°C and the supernatant was collected.

Immunoblot was performed as previously described[67,68] using the following antibodies: Chi3l1 (1/1000, Invitrogen PA5-81356), CitH3 (1/1000, Abcam Ab5103), TSP1 (1/1000, Cell Signaling 37879S), β-actin (1/5000, Sigma Aldrich A5441), Vinculin (1/2000, Millipore MAB3574) and α-tubulin (1/1000, Cell signaling 3873). The membranes were imaged using the Odyssey CL-C imaging system. Image Studio software (LICOR Biosciences) was used to analyze the images. Each target protein was normalized to its appropriate loading control on the same membrane.

### Multiplex fluorescent immunohistochemistry

FFPE tissue sections were stained as previously described.[24] Briefly, the slides were deparaffinized in 3 successive 3-min incubations with xylene followed by dehydration in decreasing concentrations of ethanol. Target retrieval was performed in Citrate buffer (Ph 6, Vector Laboratories #H-3300–250) or EDTA Buffer (Ph 9, Vector Laboratories #H-3301–250) using a pressure cooker for 10 min. In order to block endogenous H2O2, the samples were incubated in 3% hydrogen peroxide for 10 min before blocking with casein-based buffer (Vector Laboratories, #SP-5020) for 5 min. Primary antibody was added for 30 min at room temperature. After 3 washes with tris-buffered saline with tween 20 (TBST), ImmPRESS HRP Polymer secondary antibodies (Vector Laboratories; anti-mouse VECTMP745250 or anti-rabbit VECTMP740150) was added for 30 min at room temperature. 3 additional washes with TBST were performed before adding the fluorophore. Opal working solution containing tyramide signal amplification substrates (OPAL 480: OP-001000, OPAL 520: OP-001001, OPAL 570: OP-001003, OPAL 620: OP-001004, OPAL 690: OP-001006, or OPAL 780; OP-001008 Akoya biosciences) was added and incubated for 10 min at room temperature. Slides were subjected to another round of target retrieval and the protocol was repeated up to 4 colors/antibodies per section. After the last round of staining, DAPI was added and the samples mounted for imaging.

For all staining experiments, the entire mammary gland or breast tumor tissue sections were scanned using the AxioScan Z1 digital slide scanner (Carl Zeiss). For quantifications, we used the HALO (Indica Lab) system sing one of the following algorithms: 'Fluorescent intensity' or 'Area Quantification FL v3.0.1'. The entire tissue section was analyzed for all staining experiments. No ROIs were manually selected; instead, the full tissue area was included in every analysis to avoid sampling bias. All samples were analyzed using identical image acquisition and analysis settings within the same analysis batch. Each sample represents an independent biological replicate. Quantification of immune cell populations is presented as the percentage of total cells within the analyzed region, ECM measurements are presented as the percentage of total analyzed area, and myoepithelial cell measurements are presented as cell density (number of positive cells normalized to the analyzed tissue area). The specific quantification method used for each analysis is indicated in the corresponding figure legend. Representative images throughout the manuscript are cropped, higher-magnification regions selected from the whole-slide scans to allow visualization of the relevant cellular and tissue features at publication resolution. Image selection was performed solely for visualization purposes and had no influence on the quantitative analysis, which was performed on the complete scanned section.

For ECM analyses, positive staining was quantified using the 'Area Quantification FL v3.0.1' module in HALO (Indica lab). A supervised, trainable algorithm was optimized to specifically detect fluorophore-associated signals corresponding to individual ECM markers and to calculate area fraction (positive staining as a percentage of total tissue area). Analysis of lung metastatic area was performed using HALO's 'Classifier function' which is teachable algorithm that can distinguish metastatic lesions from surrounding normal lung tissue. The percent of total lung area that is occupied by lung metastatic lesions was quantified. Distance analysis was performed using HALO's 'nearest neighbor' algorithm, which measures the distance from each cell in a target population (e.g., NE + cells) to the nearest neighbor cell of a second specified population (e.g., CK14+ *p*-FAK+ cells) and then averages these distances across the entire dataset.

The following primary antibodies were used: mouse and human CK14 (1/10, Ventana 760–4805), CK5 (1/500, Biolegend 905504), Calponin 1, (1/100, Cell Signaling 17819), P63 (1/300, Santa Cruz sc25268), α-SMA (1/400, DAKO M0851), Ly6G (1/100, Cell signaling 87048), MPO (1/500, Abcam ab208670), CitH3 (1/100, Cell signaling 26111), mouse NE (1/200, Cell signaling 90120), Human NE (1/200, Cell signaling 28162), Laminin (1/200, Abcam ab11575), Collagen IV (1/100, Abcam ab6586), Elastin (1/200, BIOS USA bs-1756R), Cleaved caspase 3 (1/200, Cell signaling 9661), *p*-FAK (1/200, Invitrogen, 44-624G), Ck8 (1/100, Biolegend 904804), CD3 (1/200, Abcam ab16669), CD8 (1/200, Cell signaling 98941), CD4 (1/50, Cell signaling 25229), PD1 (1/200, Cell signaling 84651), Granzyme B (1/200, Cell signaling 44153), Ki67 (1/200, Cell signaling 12202), PCNA (1/400, Cell signaling 2586) and Pan-ck (1/10, Ventana 760–2595). All antibodies were diluted in 2% BSA TBST.

### FACS analysis

Mammary glands from MTB MIC mice treated with Alvelestat or vehicle control for 2 weeks were excised and the lymph nodes were removed. The mammary glands were then processed as described previously.[24,25] BD Cytofix/Cytoperm fixation/permeabilization kit (BD Bioscience, #554714) was used according to manufacturer's protocol. The following antibodies were used: Viability was

assessed using fixable viability dye eFluorYM 506 (ThermoFisher/eBioscience 65–0866–14). Viable cells were then analyzed using the following antibodies: CD45 (BD Bioscience, 564225), CD11b (Biolegend, 101201), F4/80 (Biolegend, 123114), Ly6G (Biolegend, 127606), Epcam (Biolegend, 118233), CD49f (Biolegend, 313622), CK5 (Santa Cruz, sc-32721 PE), and CK14 (Santa Cruz, sc-53253AF488). Flow cytometry was done in the BD LSR Fortessa 4 lasers (405/488/561/633 nm) flow cytometer and the results were analyzed using Flowjo 10.6.2 (Treestar).

### Enzyme-linked immunosorbent assay

Cardiac puncture was performed to collect blood from Nex20-treated mice at 2-week post induction. The blood was incubated at room temperature for 1 h to allow it to clot before being centrifuged for 20 min at 1000xg at 4°C. The supernatant (serum) was collected and used for enzyme-linked immunosorbent assay ELISA at a 1 in 50 dilution. ELISA for NE (R&D, Cat# DY4517-05) or MPO (R&D, Cat# DY3667) was performed following the manufacturer's protocol. Each experiment was performed in duplicates and repeated twice.

### *In vitro* laminin degradation assay

To test for laminin degradation on synthetic ECM, Cultrex Growth Factor Basement Membrane Extract (R&D, Cat# 3433–010–01). Was used to coat a 96-well glass bottom microscopy plate (Thermofisher Scientific, Nunc, Cat#165305). The plate was incubated at 37°C in 5% $CO_2$ for 30 min to solidify. PyMT+ cells were plated at $1 \times 10^4$ cells per well with neutrophil conditioned media treated with Alvelestat or Vehicle control. The plates were incubated at 37°C in 5% $CO_2$ for 12 h. After washing with PBS, the cells were fixed using 2% paraformaldehyde. Cells were then permeabilized with PBS containing 0.5% Triton X-100 for 10 min at room temperature, followed by blocking using PBS with 0.2% Triton X-100 and 0.05% Tween 20 (IF buffer) and 2% BSA for 30 min at room temperature. Primary anti-laminin antibody (1/200, Abcam Ab11575) was added and incubated overnight at 4°C. The cells were washed three times before adding Goat anti-rabbit secondary antibody Alexa Fluor 555 (Invitrogen A21428) for 40 min at room temperature. After three additional washes with IF buffer, DAPI was added for 5 min at room temperature. The cells were then incubated in PBS and imaged using Zeiss AxioObserver fully motorized inverted confocal microscope LSM 710 with a 10× objective. Each experiment was performed in triplicates and repeated twice.

This assay was also repeated in neutrophil conditioned medium or media supplemented with PBS control, 1 μg/mL of recombinant mouse neutrophil elastase (R&D system, 4517-SE), 1 μg/mL of recombinant mouse cathepsin G (Biomatik, RPU51859), 2 μg/mL of recombinant mouse proteinase 3 (Biomatik, RPU43519) or 200ng/mL of recombinant murine Chi3l1(R&D systems, 2649-CH). All recombinant proteins were dissolved in PBS. The assay was ran in triplicates and repeated twice.

### *In vitro* collagen IV degradation assay

To test for laminin degradation on synthetic ECM, DQ collagen, type IV from human placenta, fluorescein conjugate (Invitrogen, D12052) was used to coat a 96-well glass bottom microscopy plate (Thermofisher Scientific, Nunc, Cat#165305). DQ Collagen was prepared at a 1mg/mL concentration in distilled water. The coating was dissolved by heating it up to 50°C with agitation. The stock was then diluted to a working concentration of 25 μg/mL in 0.1% unconjugated gelatin. PyMT+ cancer cells were culture in neutrophil conditioned media treated with Alvelestat or vehicle control and were incubated at 37°C in 5% $CO_2$ for 12 h. After washing with PBS, the cells were fixed using 2% paraformaldehyde. Cells were then permeabilized with PBS containing 0.5% Triton X-100 for 10 min at room temperature, followed by blocking using PBS with 0.2% Triton X-100 and 0.05% Tween 20 (IF buffer) and 2% BSA for 30 min at room temperature. At that point, degraded collagen would fluoresce green under the microscope. For Actin staining, Alexa Fluor plus 555 phalloidin was used (1/500 Invitrogen A30106) and incubated for 45 min at room temperature. After three additional washes with IF buffer, DAPI was added for 5 min at room temperature. The cells were then incubated in PBS and imaged using Zeiss AxioObserver fully motorized inverted confocal microscope LSM 710 with a 10× objective. The experiment was performed in triplicates and repeated twice.

The assay was also repeated in the presence of recombinant NE, recombinant cathepsin G, recombinant Proteinase 3 or recombinant Chi3l1 as described for laminin degradation assay.

### Trans-well invasion assay

PyMT cancer cell invasion assay through synthetic ECM was performed as described previously.[25] PyMT cancer cells were placed on top of the inserts in the appropriate conditioned media, and the assay was performed as previously described.[25] Following 12 h of incubation at 37°C, 5% CO2, the filters were removed, and the non-migrating cells were removed from the top of the membrane. The cells that invaded through the membrane were fixed in 10% neutral buffered formalin for 30 min and filters were counterstained with crystal violet. Image acquisition of stained cells was performed with an EVOS M7000 Invitrogen microscope (objective 10×). Cells were manually counted on HALO. The assay was repeated twice.

### Organoid culture and NE treatment

Mammary glands (3rd, 4th and 5th) were isolated from MIC CK14-Tdtomato[40] adult mice, minced with scissors and digested in a pre-warmed digestion media (DMEM/F12 supplemented with 5 μg/mL insulin, 600 U/mL Nystatin, 100 U/mL Penicillin/Streptomycin, 2 mg/mL Collagenase A, 0.00625% Trypsin EDTA) for 30 min with rotation. Following a 5-min 1000rpm spin at room temperature,

CellPress
OPEN ACCESS

cells were rinsed in DMEM/F12 and spun for 10 min, at 1000rpm spin at room temperature. Epithelial clumps were purified through a series of five quick spins (1500rpm, 15 s) and washes (PBS 5%FBS). Following this, organoids were resuspended in 100% Geltrex (gibco#A1413202) and plated in domes above a 100% Geltrex pad in ibidi plates (ibidi#80826) and grown with Mammosphere media (EpiCult Mouse media (STEMCELL#05610) supplemented with 5 μg/mL insulin, 100 U/mL Penicillin/Streptomycin, 0.02 μg/mL mEGF (R&D systems#2028-EG-200), 1 μg/mL hydrocortisone, 4% KnockOut serum (gibco#10828010)) with doxycycline (3mg/mL). These were allowed to recover and reorganize into organoids over a 72-h period and were then removed from their Geltrex domes with prewarmed cell recovery solution (Corning#354253). Organoids were then moved to rolling culture (in mammosphere media) where they were treated with either vehicle (PBS) or neutrophil elastase (50mM) for 24 h. Prior to imaging, organoids were stained with Incucyte Cytotox 488 reagent (Sartorius#4633) at 1:2000 dilution to visualize dying cells and DRAQ5 (Thermofisher# 62251) to visualize nuclei. Organoids were carefully moved to a clean ibidi plate and imaged using the LSM-910 confocal laser scanning microscope, equipped with a 20×/NA = 0.40 long distance objective lens. Following imaging of live organoids, organoids were fixed in 4% PFA for 20 min at room temperature and immunostained using antibodies against cytokeratin 5 (Biolegend #905901) at 1/200 dilution. Analysis was performed using ImageJ v1.54p. Organoid perimeter was measured manually by tracing the outline of the organoid structures in the DIC channel. All tdTomato positive cells that came in contact with the organoid perimeter were counted. Statistical analysis on this data was done using JMP Student Edition 19. For each condition, $n = 3$ mice were used to generate 10–15 organoids per mouse.

## QUANTIFICATION AND STATISTICAL ANALYSIS

All statistical analyses were performed using Prism software (GraphPad, San Diego, CA). The appropriate statistical test was used for different experiments and is indicated in the figure legend. One-way ANOVA with Tukey's or Dunnett's post-hoc tests for multiple comparisons were performed as appropriate. Mann-Whitney non-parametric test, two-tailed paired or un-paired Student's *t* test were used to compare two groups. Effect sizes were determined using Cohen's d for two-group comparisons or Eta squared for multiple comparison, the values are indicated in the figure legends. For all figures, the data is plotted as average ±standard error of the mean (SEM). Each data point is an independent biological sample. The results of the statistical tests can be found within the figures and figure legends. $p$ values less than 0.05 were considered statistically significant. Sample size ($n$) is indicated in the figure legend. ($n$) represents independent biological replicates (separate mice or human breast cancer samples). Sample sizes were determined using a combination of *a priori* power calculations (power = 0.8, $\alpha = 0.05$) for key genetic mouse cross experiments and estimates derived from pilot studies and prior published work. For *in vivo* drug treatment experiments, sample sizes were based on prior literature and pilot experiments using the same model systems. No samples were excluded.

Cell Reports Medicine, Volume *7*

## Supplemental information

# Neutrophil elastase-mediated myoepithelial loss is a therapeutic vulnerability in ductal carcinoma *in situ* progression

**Tarek Taifour, Yu Gu, Adéline Massé, Xiwei Meng, Clarisse Dane Echavez, Dongmei Zuo, Virginie Sanguin-Gendreau, Anne Blouin, Adda-Lee Graham-Paquin, Miranda Addie, Emilie Solymoss, Bin Xiao, Hailey Proud, Sherif Samer Attalla, Alice Jisoo Nam, Vasilios Papavasiliou, Hellen Kuasne, Peter M. Siegel, Khandan Keyomarsi, Luke McCaffrey, Morag Park, and William J. Muller**

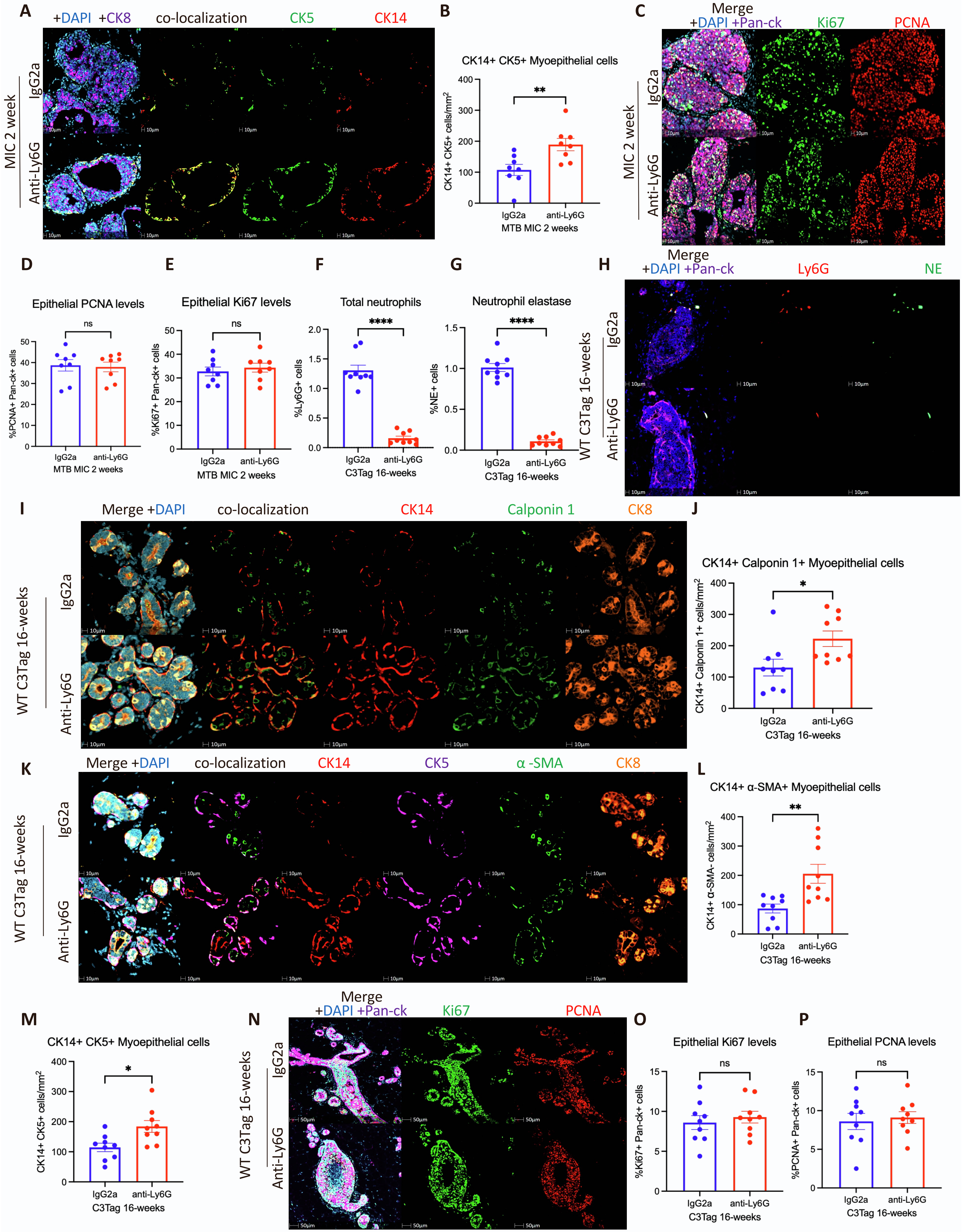

A
+DAPI +CK8
co-localization
CK5
CK14
MIC 2 week
IgG2a
Anti-Ly6G
B
CK14+ CK5+ Myoepithelial cells
CK14+ CK5+ cells/mm2
IgG2a
anti-Ly6G
MTB MIC 2 weeks
C
Merge
+DAPI +Pan-ck
Ki67
PCNA
D
Epithelial PCNA levels
%PCNA+ Pan-ck+ cells
MTB MIC 2 weeks
E
Epithelial Ki67 levels
%Ki67+ Pan-ck+ cells
MTB MIC 2 weeks
F
Total neutrophils
%Ly6G+ cells
C3Tag 16-weeks
G
Neutrophil elastase
%NE+ cells
C3Tag 16-weeks
H
Merge
+DAPI +Pan-ck
Ly6G
NE
WT C3Tag 16-weeks
I
Merge +DAPI
co-localization
CK14
Calponin 1
CK8
J
CK14+ Calponin 1+ Myoepithelial cells
CK14+ Calponin 1+ cells/mm2
C3Tag 16-weeks
K
Merge +DAPI
co-localization
CK14
CK5
α -SMA
CK8
L
CK14+ α-SMA+ Myoepithelial cells
CK14+ α-SMA- cells/mm2
C3Tag 16-weeks
M
CK14+ CK5+ Myoepithelial cells
CK14+ CK5+ cells/mm2
C3Tag 16-weeks
N
Merge
+DAPI +Pan-ck
Ki67
PCNA
WT C3Tag 16-weeks
O
Epithelial Ki67 levels
%Ki67+ Pan-ck+ cells
C3Tag 16-weeks
P
Epithelial PCNA levels
%PCNA+ Pan-ck+ cells
C3Tag 16-weeks

**Supplementary Figure 1: Neutrophils depletion increases CK5 myoepithelial marker without Impacting cancer proliferation. Related to Figure 1.**

**(A)** Fluorescent IHC staining of mammary tissue from WT MIC mice treated with anti-Ly6G or IgG2a isotype control for 2 weeks, using antibodies against CK14, CK5 and CK8, counterstained with DAPI. **(B)** Quantification of total CK14+ CK5+ cells normalized to total analyzed area in IgG2a ($n = 8$) and anti-Ly6G ($n = 8$) treated WT MIC mammary glands at 2 weeks post induction. Derived from the staining shown in A. **(C)** Fluorescent IHC staining of mammary tissue from WT MIC mice treated with anti-Ly6G or IgG2a isotype control for 2 weeks, using antibodies against Ki67, PCNA and Pan-ck, counterstained with DAPI. **(D,E)** Quantification of total Pan-ck+ Ki67+ and Pan-ck+ PCNA+ cells as a percentage of total cells in IgG2a ($n = 8$) and anti-Ly6G ($n = 8$) treated WT MIC mammary glands at 2 weeks post induction. Derived from the staining shown in C. **(F,G)** Quantification of total Ly6G+ and NE+ cells as a percentage of total cells in IgG2a ($n = 9$), and anti-Ly6G ($n = 9$) treated WT C3Tag mammary glands at 16-weeks. Derived from the staining shown in H. **(H)** Fluorescent IHC staining of IgG2a and anti-Ly6G-treated WT C3Tag at 16-weeks using antibodies against Ly6G, NE, and Pan-ck, counterstained with DAPI. **(I)** Fluorescent IHC staining of IgG2a and anti-Ly6G-treated WT C3Tag at 16-weeks using antibodies against CK14, Calponin 1, and CK8, counterstained with DAPI. **(J)** Quantification of total CK14+ calponin 1+ cells normalized to total analyzed area in IgG2a ($n = 9$), and anti-Ly6G ($n = 9$) treated WT C3Tag mammary glands at 16-weeks. Derived from the staining shown in I. **(K)** Fluorescent IHC staining of IgG2a and anti-Ly6G-treated WT C3Tag at 16-weeks using antibodies against CK14, α-SMA, CK5 and CK8, counterstained with DAPI. **(L,M)** Quantification of total CK14+ α-SMA+ and CK14+ CK5+ cells normalized to total analyzed area in IgG2a ($n = 9$), and anti-Ly6G ($n = 9$) treated WT C3Tag mammary glands at 16-weeks. Derived from the staining shown in K. **(N)** Fluorescent IHC staining of IgG2a and anti-Ly6G-treated WT C3Tag at 16-weeks using antibodies against Ki67, PCNA, and Pan-ck counterstained with DAPI. **(O,P)** Quantification of total Pan-ck+ Ki67+ and Pan-ck+ PCNA+ cells as a percentage of total cells in IgG2a ($n = 9$), and anti-Ly6G ($n = 9$) treated WT C3Tag mammary glands at 16-weeks. Derived from the staining shown in N. Ns: not significant, $^*p<0.05$, $^{**}p<0.01$, and $^{****}p<0.0001$ calculated by Mann-Whitney non-parametric test. Data are presented as mean ± SEM. Each data point is an independent biological sample, *N* represents the number of independent biological replicates. Analysis was performed by the HALO (Indica lab) software. Quantification was performed on whole-slide scans of the entire mammary gland section.

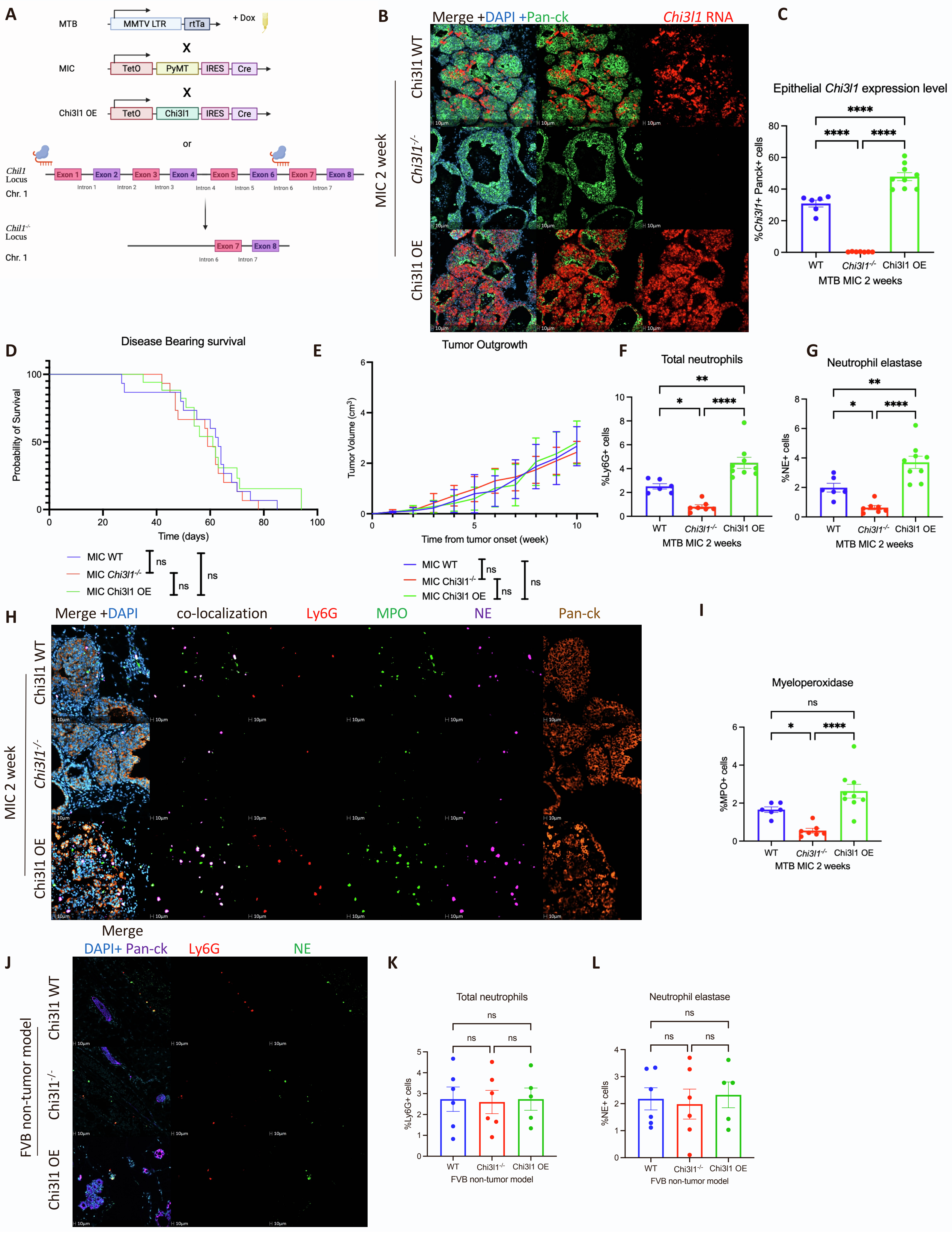

A
MTB
MMTV LTR
rtTa
+ Dox
X
MIC
TetO
PyMT
IRES
Cre
X
Chi3l1 OE
TetO
Chi3l1
IRES
Cre
or
Chil1 Locus
Chr. 1
Exon 1
Exon 2
Exon 3
Exon 4
Exon 5
Exon 6
Exon 7
Exon 8
Intron 1
Intron 2
Intron 3
Intron 4
Intron 5
Intron 6
Intron 7
Chil1-/- Locus
Chr. 1
Exon 7
Exon 8
Intron 6
Intron 7
B
Merge +DAPI +Pan-ck
Chi3l1 RNA
MIC 2 week
Chi3l1 WT
Chi3l1-/-
Chi3l1 OE
C
Epithelial Chi3l1 expression level
%Chi3l1+ Panck+ cells
****
****
****
WT
Chi3l1-/-
Chi3l1 OE
MTB MIC 2 weeks
D
Disease Bearing survival
Probability of Survival
Time (days)
MIC WT
MIC Chi3l1-/-
MIC Chi3l1 OE
ns
ns
ns
E
Tumor Outgrowth
Tumor Volume (cm3)
Time from tumor onset (week)
MIC WT
MIC Chi3l1-/-
MIC Chi3l1 OE
ns
ns
ns
F
Total neutrophils
%Ly6G+ cells
**
*
****
WT
Chi3l1-/-
Chi3l1 OE
MTB MIC 2 weeks
G
Neutrophil elastase
%NE+ cells
**
*
****
WT
Chi3l1-/-
Chi3l1 OE
MTB MIC 2 weeks
H
Merge +DAPI
co-localization
Ly6G
MPO
NE
Pan-ck
MIC 2 week
Chi3l1 WT
Chi3l1-/-
Chi3l1 OE
I
Myeloperoxidase
%MPO+ cells
ns
*
****
WT
Chi3l1-/-
Chi3l1 OE
MTB MIC 2 weeks
J
Merge
DAPI+ Pan-ck
Ly6G
NE
FVB non-tumor model
Chi3l1 WT
Chi3l1-/-
Chi3l1 OE
K
Total neutrophils
%Ly6G+ cells
ns
ns
ns
WT
Chi3l1-/-
Chi3l1 OE
FVB non-tumor model
L
Neutrophil elastase
%NE+ cells
ns
ns
ns
WT
Chi3l1-/-
Chi3l1 OE
FVB non-tumor model

**Supplementary Figure 2: Characterization of the *Chi3l1*$^{-/-}$ and Chi3l1 OE MIC GEMMs. Related to Figure 1.**
**(A)** Schematic representation of the MTB MIC *Chi3l1*$^{-/-}$ and Chi3l1 OE GEMMs used in this study, created with BioRender.com. **(B)** RNA FISH against *Chi3l1* combined with fluorescent IHC staining for Pan-ck, and DAPI on mammary tissue from WT, *Chi3l1*$^{-/-}$ and Chi3l1 OE MIC mammary glands at 2 weeks post induction. **(C)** Quantification of *Chi3l1*+Pan-ck+ cells as a percentage of total cells in WT (*n* = 6), *Chi3l1*$^{-/-}$ (*n* = 7), and Chi3l1 OE (*n* = 9) MIC mammary glands at 2 weeks post induction. Derived from the staining shown in B. **(D)** Disease-bearing survival of WT (*n* = 14), *Chi3l1*$^{-/-}$ (*n* = 15), and Chi3l1 OE (*n* = 17) MIC mammary tumor-bearing mice measured from day of measurable tumor (onset) to humane endpoint. Statistical analysis by Mantel-Cox test. **(E)** Primary tumors growth in WT (*n* = 14), *Chi3l1*$^{-/-}$ (*n* = 15), and Chi3l1 OE (*n* = 17) MIC mammary tumor-bearing mice. Tumors were measured and tracked weekly by a caliper. **(F,G)** Quantification of total Ly6G+ and NE+ cells as a percentage of total cells in WT (*n* = 6), *Chi3l1*$^{-/-}$ (*n* = 7), and Chi3l1 OE (*n* = 9) MIC mammary glands at 2 weeks post induction. Derived from the staining shown in H. **(H)** Fluorescent IHC staining of WT, *Chi3l1*$^{-/-}$, and Chi3l1 OE MIC mammary glands at 2 weeks post induction using antibodies against Ly6G, MPO, NE, and Pan-ck, counterstained with DAPI. **(I)** Quantification of total MPO+ cells as a percentage of total cells in WT (*n* = 6), *Chi3l1*$^{-/-}$ (*n* = 7), and Chi3l1 OE (*n* = 9) MIC mammary glands at 2 weeks post induction. Derived from the staining shown in H. **(J)** Fluorescent IHC staining of WT, *Chi3l1*$^{-/-}$, and Chi3l1 OE (non-MIC, non-tumor) mammary glands using antibodies against Ly6G, NE, and Pan-ck, counterstained with DAPI. **(K,L)** Quantification of total Ly6G+ and NE+ cells as a percentage of total cells in WT (*n* = 6), *Chi3l1*$^{-/-}$ (*n* = 6), and Chi3l1 OE (*n* = 5) (non-MIC, non-tumor) mammary glands. Derived from the staining shown in J. Ns: not significant, *$p<0.05$, **$p<0.01$, and ****$p<0.0001$ calculated by One-Way Anova with Tukey's post-hoc test. Data are presented as mean ± SEM. Each data point is an independent biological sample, *N* represents the number of independent biological replicates. Analysis was performed by the HALO (Indica lab) software. Quantification was performed on whole-slide scans of the entire mammary gland section.

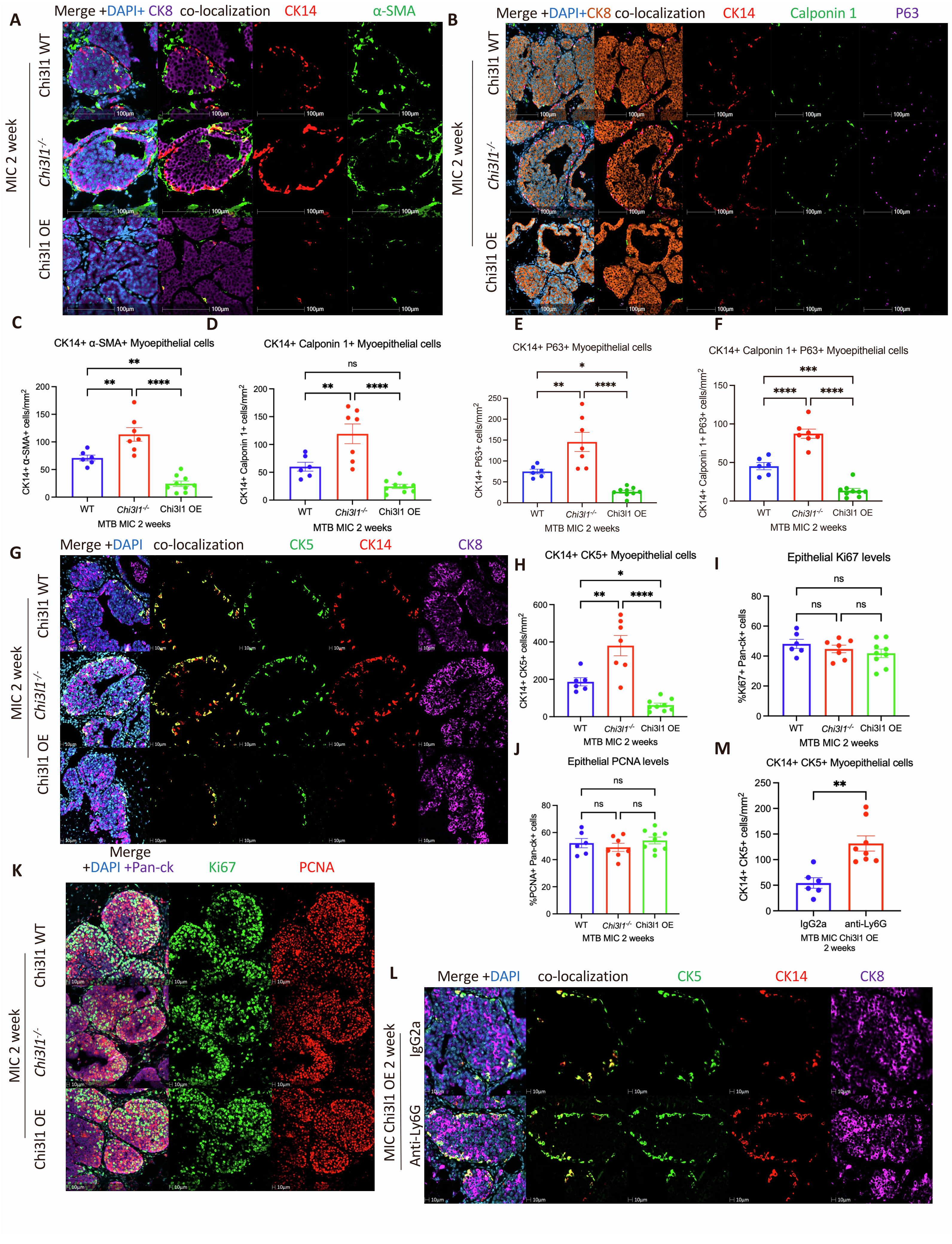

A
Merge +DAPI+ CK8 co-localization CK14 α-SMA
MIC 2 week
Chi3l1 WT
Chi3l1-/-
Chi3l1 OE
100μm
B
Merge +DAPI+CK8 co-localization CK14 Calponin 1 P63
C
CK14+ α-SMA+ Myoepithelial cells
CK14+ α-SMA+ cells/mm2
WT Chi3l1-/- Chi3l1 OE
MTB MIC 2 weeks
D
CK14+ Calponin 1+ Myoepithelial cells
CK14+ Calponin 1+ cells/mm2
E
CK14+ P63+ Myoepithelial cells
CK14+ P63+ cells/mm2
F
CK14+ Calponin 1+ P63+ Myoepithelial cells
CK14+ Calponin 1+ P63+ cells/mm2
G
Merge +DAPI co-localization CK5 CK14 CK8
H
CK14+ CK5+ Myoepithelial cells
CK14+ CK5+ cells/mm2
I
Epithelial Ki67 levels
%Ki67+ Pan-ck+ cells
J
Epithelial PCNA levels
%PCNA+ Pan-ck+ cells
M
CK14+ CK5+ Myoepithelial cells
CK14+ CK5+ cells/mm2
IgG2a anti-Ly6G
MTB MIC Chi3l1 OE
2 weeks
K
Merge
+DAPI +Pan-ck Ki67 PCNA
L
Merge +DAPI co-localization CK5 CK14 CK8
MIC Chi3l1 OE 2 week
IgG2a
Anti-Ly6G
10μm

**Supplementary Figure 3: Genetic ablation of Chi3l1 prevents myoepithelial layer dissolution, whereas Chi3l1 overexpression accelerates it. Related to Figure 1.**

**(A)** Fluorescent IHC staining of WT, *Chi3l1*$^{-/-}$ and Chi3l1 OE MIC mammary glands at 2 weeks post-induction using antibodies against CK14, α-SMA, and CK8, counterstained with DAPI. **(B)** Fluorescent IHC staining of WT, *Chi3l1*$^{-/-}$ and Chi3l1 OE MIC mammary glands at 2 weeks post induction using antibodies against CK14, calponin 1, P63, and CK8, counterstained with DAPI. **(C-F)** Quantification of total CK14+ α-SMA+, CK14+ calponin 1+, CK14+ P63+ and CK14+ calponin 1+ P63+ cells normalized to total analyzed area in WT (*n* = 6), *Chi3l1*$^{-/-}$ (*n* = 7), and Chi3l1 OE (*n* = 9) MIC mammary glands at 2 weeks post induction. Derived from the staining shown in A,B. **(G)** Fluorescent IHC staining of WT, *Chi3l1*$^{-/-}$ and Chi3l1 OE MIC mammary glands at 2 weeks post-induction using antibodies against CK14, CK5, and CK8, counterstained with DAPI. **(H)** Quantification of total CK14+ CK5+ cells normalized to total analyzed area in WT (*n* = 6), *Chi3l1*$^{-/-}$ (*n* = 7), and Chi3l1 OE (*n* = 9) MIC mammary glands at 2 weeks post induction. Derived from the staining shown in G. **(I,J)** Quantification of total Pan-ck+ Ki67+ and Pan-ck+ PCNA+ cells as a percentage of total cells in WT (*n* = 6), *Chi3l1*$^{-/-}$ (*n* = 7), and Chi3l1 OE (*n* = 9) MIC mammary glands at 2 weeks post induction. Derived from the staining shown in K. **(K)** Fluorescent IHC staining of WT, *Chi3l1*$^{-/-}$ and Chi3l1 OE MIC mammary glands at 2 weeks post induction using antibodies against PCNA, Ki67, and Pan-ck, counterstained with DAPI. **(L)** Fluorescent IHC staining of mammary tissue from Chi3l1 OE MIC mice treated with anti-Ly6G or IgG2a isotype control for 2 weeks, using antibodies against CK14, CK5, and CK8, counterstained with DAPI. **(M)** Quantification of total CK14+ CK5+ cells normalized to total analyzed area in IgG2a (*n* = 6) and anti-Ly6G (*n* = 8) treated Chi3l1 OE MIC mammary glands at 2 weeks post induction. Derived from the staining shown in L. Ns: not significant, *p<0.05, **p<0.01, ***p<0.001 and ****p<0.0001 calculated by One-Way Anova with Tukey's post-hoc test. Mann-Whitney non-parametric test was used for M. Data are presented as mean ± SEM. Each data point is an independent biological sample, *N* represents the number of independent biological replicates. Analysis was performed by the HALO (Indica lab) software. Quantification was performed on whole-slide scans of the entire mammary gland section.

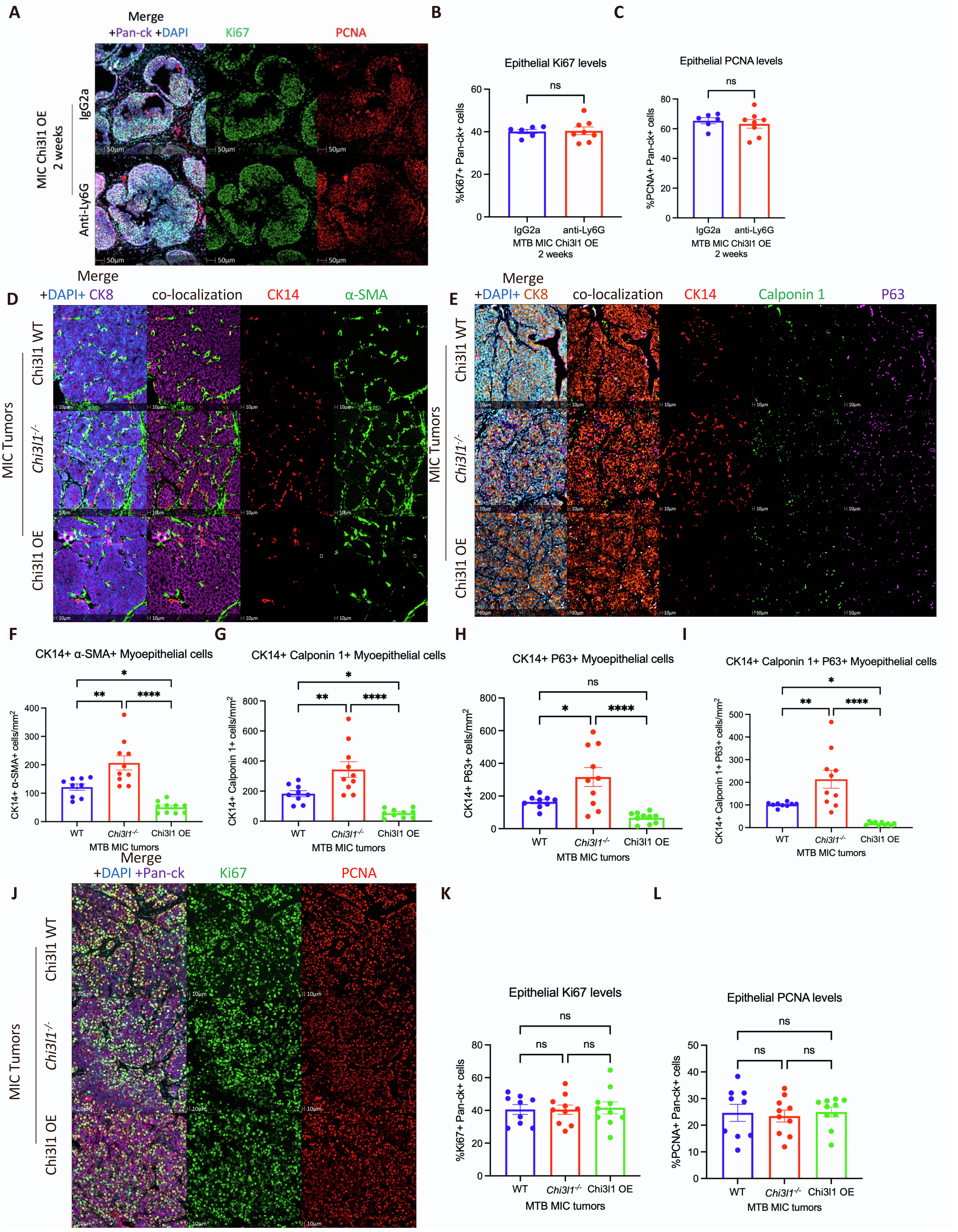
A
Merge
+Pan-ck +DAPI
Ki67
PCNA
MIC Chi3l1 OE 2 weeks
IgG2a
Anti-Ly6G
B
Epithelial Ki67 levels
%Ki67+ Pan-ck+ cells
IgG2a
anti-Ly6G
MTB MIC Chi3l1 OE 2 weeks
C
Epithelial PCNA levels
%PCNA+ Pan-ck+ cells
D
Merge +DAPI+ CK8
co-localization
CK14
α-SMA
MIC Tumors
Chi3l1 WT
Chi3l1-/-
Chi3l1 OE
E
Merge +DAPI+ CK8
co-localization
CK14
Calponin 1
P63
F
CK14+ α-SMA+ Myoepithelial cells
CK14+ α-SMA+ cells/mm2
WT
Chi3l1-/-
Chi3l1 OE
MTB MIC tumors
G
CK14+ Calponin 1+ Myoepithelial cells
CK14+ Calponin 1+ cells/mm2
H
CK14+ P63+ Myoepithelial cells
CK14+ P63+ cells/mm2
I
CK14+ Calponin 1+ P63+ Myoepithelial cells
CK14+ Calponin 1+ P63+ cells/mm2
J
Merge +DAPI +Pan-ck
Ki67
PCNA
K
Epithelial Ki67 levels
%Ki67+ Pan-ck+ cells
L
Epithelial PCNA levels
%PCNA+ Pan-ck+ cells

**Supplementary Figure 4: Chi3l1 ablation or overexpression does not impact on cancer cell proliferation. Related to Figure 1.**
**(A)**. Fluorescent IHC staining of mammary tissue from Chi3l1 OE MIC mice treated with anti-Ly6G or IgG2a isotype control for 2 weeks, using antibodies against PCNA, Ki67, and Pan-ck, counterstained with DAPI. **(B,C)** Quantification of total Pan-ck+ Ki67+ and Pan-ck+ PCNA+ cells as a percentage of total cells in IgG2a (*n* = 6) and anti-Ly6G (*n* = 8) treated Chi3l1 OE MIC mammary glands at 2 weeks post induction**.** Derived from the staining shown in A. **(D)** Fluorescent IHC staining of WT, $Chi3l1^{-/-}$ and Chi3l1 OE MIC end-stage mammary tumors using antibodies against CK14, α-SMA, and CK8, counterstained with DAPI. **(E)** Fluorescent IHC staining of WT, $Chi3l1^{-/-}$ and Chi3l1 OE MIC end-stage mammary tumors using antibodies against CK14, calponin 1, P63, and CK8, counterstained with DAPI. **(F-I)** Quantification of total CK14+ α-SMA+, CK14+ calponin 1+, CK14+ P63+ and CK14+ calponin 1+ P63+ cells normalized to total analyzed area in WT (*n* = 9), $Chi3l1^{-/-}$ (*n* = 10), and Chi3l1 OE (*n* = 10) MIC mammary tumors. Derived from the staining shown in D,E. **(J)** Fluorescent IHC staining of WT, $Chi3l1^{-/-}$ and Chi3l1 OE MIC end-stage mammary tumors using antibodies against PCNA, Ki67, and Pan-ck, counterstained with DAPI. **(K,L)** Quantification of total Pan-ck+ Ki67+ and Pan-ck+ PCNA+ cells as a percentage of total cells in WT (*n* = 9), $Chi3l1^{-/-}$ (*n* = 10), and Chi3l1 OE (*n* = 10) MIC mammary tumors. Derived from the staining shown in J. Ns: not significant, *p<0.05, **p<0.01 and ****p<0.0001 calculated by One-Way Anova with Tukey's post-hoc test. Mann-Whitney non-parametric test was used for B,C. Data are presented as mean ± SEM. Each data point is an independent biological sample, *N* represents the number of independent biological replicates. Analysis was performed by the HALO (Indica lab) software. Quantification was performed on whole-slide scans of the entire mammary gland section.

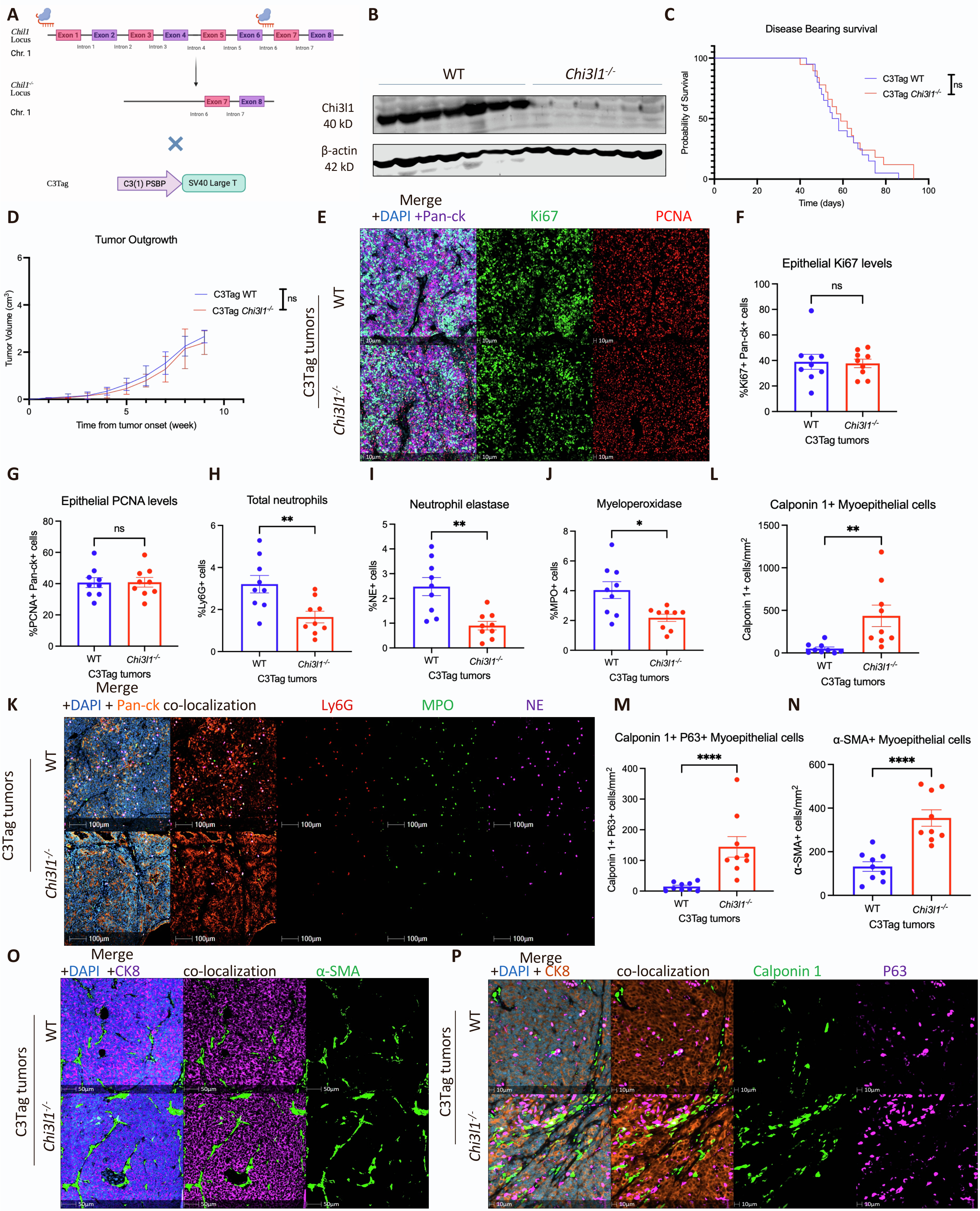

A
Chil1 Locus
Chr. 1
Exon 1
Exon 2
Exon 3
Exon 4
Exon 5
Exon 6
Exon 7
Exon 8
Intron 1
Intron 2
Intron 3
Intron 4
Intron 5
Intron 6
Intron 7
Chil1-/- Locus
Chr. 1
C3Tag
C3(1) PSBP
SV40 Large T
B
WT
Chi3l1-/-
Chi3l1
40 kD
β-actin
42 kD
C
Disease Bearing survival
Probability of Survival
Time (days)
C3Tag WT
C3Tag Chi3l1-/-
ns
D
Tumor Outgrowth
Tumor Volume (cm3)
Time from tumor onset (week)
C3Tag WT
C3Tag Chi3l1-/-
ns
E
Merge
+DAPI +Pan-ck
Ki67
PCNA
C3Tag tumors
WT
Chi3l1-/-
10µm
F
Epithelial Ki67 levels
%Ki67+ Pan-ck+ cells
ns
WT
Chi3l1-/-
C3Tag tumors
G
Epithelial PCNA levels
%PCNA+ Pan-ck+ cells
ns
WT
Chi3l1-/-
C3Tag tumors
H
Total neutrophils
%Ly6G+ cells
**
WT
Chi3l1-/-
C3Tag tumors
I
Neutrophil elastase
%NE+ cells
**
WT
Chi3l1-/-
C3Tag tumors
J
Myeloperoxidase
%MPO+ cells
*
WT
Chi3l1-/-
C3Tag tumors
L
Calponin 1+ Myoepithelial cells
Calponin 1+ cells/mm2
**
WT
Chi3l1-/-
C3Tag tumors
K
Merge
+DAPI + Pan-ck co-localization
Ly6G
MPO
NE
C3Tag tumors
WT
Chi3l1-/-
100µm
M
Calponin 1+ P63+ Myoepithelial cells
Calponin 1+ P63+ cells/mm2
****
WT
Chi3l1-/-
C3Tag tumors
N
α-SMA+ Myoepithelial cells
α-SMA+ cells/mm2
****
WT
Chi3l1-/-
C3Tag tumors
O
Merge
+DAPI +CK8
co-localization
α-SMA
C3Tag tumors
WT
Chi3l1-/-
50µm
P
Merge
+DAPI + CK8
co-localization
Calponin 1
P63
C3Tag tumors
WT
Chi3l1-/-
10µm

**Supplementary Figure 5: Characterization of Chi3l1-deficient C3Tag GEMM. Related to Figure 1.**
**(A)** Schematic representation of the C3Tag *Chi3l1*$^{-/-}$ GEMM used in this study, created with BioRender.com. **(B)** Immunoblots for Chi3l1 and β-actin on WT ($n$ = 7) and *Chi3l1*$^{-/-}$ ($n$ = 6) C3Tag mammary endpoint tumors. **(C)** Disease-bearing survival of WT ($n$ = 20) and *Chi3l1*$^{-/-}$ ($n$ = 19) C3Tag mammary tumor-bearing mice measured from day of measurable tumor (onset) to humane endpoint. Statistical analysis by Mantel–Cox test. **(D)** Primary tumors growth in WT ($n$ = 7) and *Chi3l1*$^{-/-}$ ($n$ = 10) C3Tag mammary tumor-bearing mice. Tumors were measured and tracked weekly by a caliper. **(E)** Fluorescent IHC staining of WT and *Chi3l1*$^{-/-}$ C3Tag end-stage mammary tumors, using antibodies against Ki67, PCNA and Pan-ck, counterstained with DAPI. **(F,G)** Quantification of total Pan-ck+ Ki67+ and Pan-ck+ PCNA+ cells as a percentage of total cells in WT ($n$ = 9) and *Chi3l1*$^{-/-}$ ($n$ = 9) C3Tag end-stage mammary tumors. Derived from the staining shown in E. **(H-J)** Quantification of total Ly6G+, NE+ and MPO+ cells as a percentage of total cells in WT ($n$ = 9) and *Chi3l1*$^{-/-}$ ($n$ = 9) C3Tag end-stage mammary tumors. Derived from the staining shown in K. **(K)** Fluorescent IHC staining of WT and *Chi3l1*$^{-/-}$ C3Tag end-stage mammary tumors using antibodies against Ly6G, MPO, NE and Pan-ck, counterstained with DAPI. **(L-N)** Quantification of total Calponin 1+, calponin 1+ P63+ and α-SMA+ cells normalized to total analyzed area in WT ($n$ = 9) and *Chi3l1*$^{-/-}$ ($n$ = 9) C3Tag end-stage mammary tumors. Derived from the staining shown in O,P. **(O)** Fluorescent IHC staining of WT and *Chi3l1*$^{-/-}$ C3Tag end-stage mammary tumors using antibodies against α-SMA, and CK8, counterstained with DAPI. **(P)** Fluorescent IHC staining of WT and *Chi3l1*$^{-/-}$ C3Tag end-stage mammary tumors using antibodies against calponin 1, P63 and CK8, counterstained with DAPI. Ns: not significant, *$p<0.05$, **$p<0.01$ and ***$p<0.001$ calculated by Mann-Whitney non-parametric test. Data are presented as mean ± SEM. Each data point is an independent biological sample, *N* represents the number of independent biological replicates. Analysis was performed by the HALO (Indica lab) software. Quantification was performed on whole-slide scans of the entire mammary gland section.

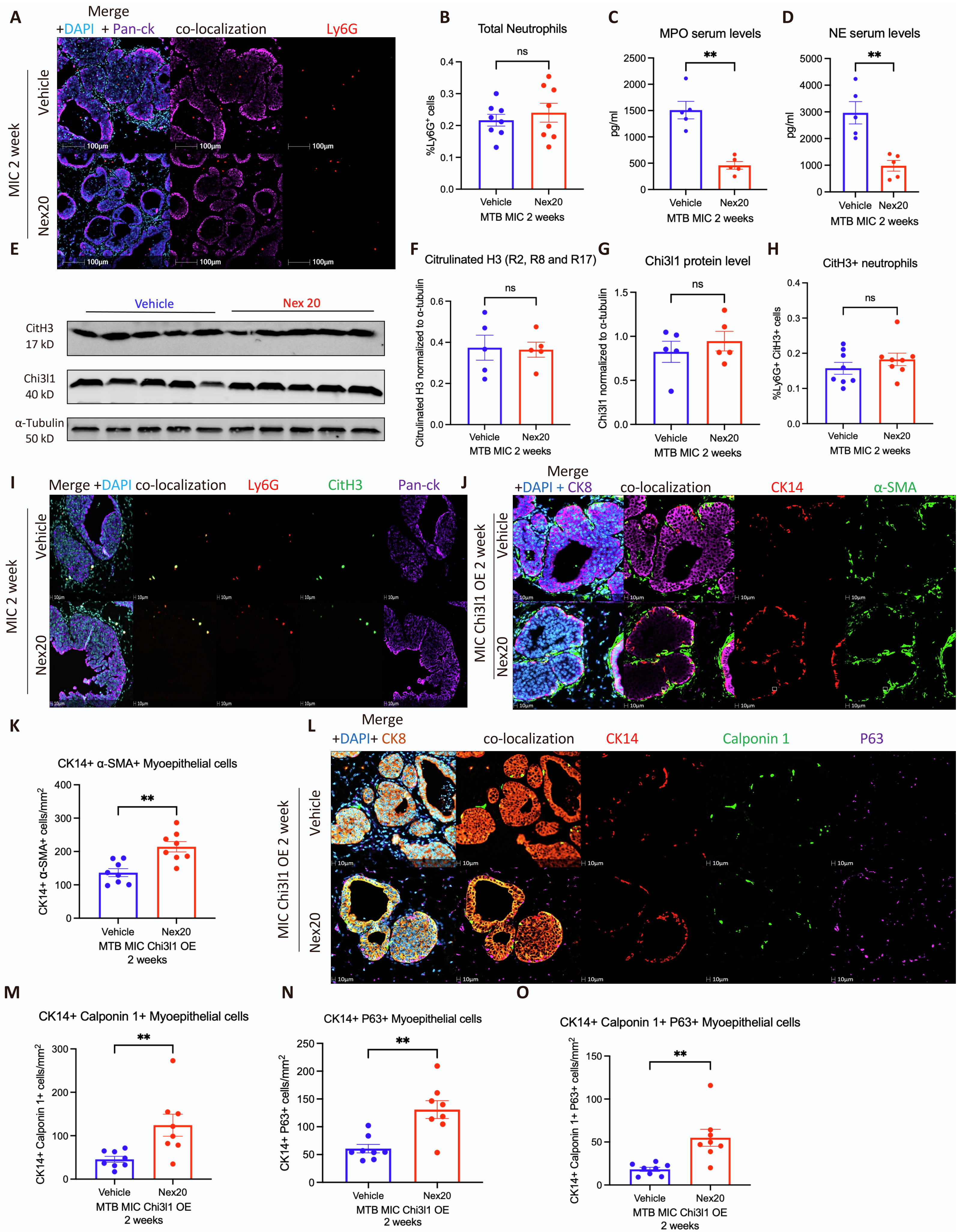

A
Merge
+DAPI + Pan-ck
co-localization
Ly6G
MIC 2 week
Vehicle
Nex20
100µm
B
Total Neutrophils
%Ly6G+ cells
ns
Vehicle
Nex20
MTB MIC 2 weeks
C
MPO serum levels
pg/ml
**
Vehicle
Nex20
MTB MIC 2 weeks
D
NE serum levels
pg/ml
**
Vehicle
Nex20
MTB MIC 2 weeks
E
Vehicle
Nex 20
CitH3
17 kD
Chi3l1
40 kD
α-Tubulin
50 kD
F
Citrulinated H3 (R2, R8 and R17)
Citulinated H3 normalized to α-tubulin
ns
Vehicle
Nex20
MTB MIC 2 weeks
G
Chi3l1 protein level
Chi3l1 normalized to α-tubulin
ns
Vehicle
Nex20
MTB MIC 2 weeks
H
CitH3+ neutrophils
%Ly6G+ CitH3+ cells
ns
Vehicle
Nex20
MTB MIC 2 weeks
I
Merge +DAPI
co-localization
Ly6G
CitH3
Pan-ck
MIC 2 week
Vehicle
Nex20
J
Merge
+DAPI + CK8
co-localization
CK14
α-SMA
MIC Chi3l1 OE 2 week
Vehicle
Nex20
K
CK14+ α-SMA+ Myoepithelial cells
CK14+ α-SMA+ cells/mm²
**
Vehicle
Nex20
MTB MIC Chi3l1 OE
2 weeks
L
Merge
+DAPI+ CK8
co-localization
CK14
Calponin 1
P63
MIC Chi3l1 OE 2 week
Vehicle
Nex20
M
CK14+ Calponin 1+ Myoepithelial cells
CK14+ Calponin 1+ cells/mm²
**
Vehicle
Nex20
MTB MIC Chi3l1 OE
2 weeks
N
CK14+ P63+ Myoepithelial cells
CK14+ P63+ cells/mm²
**
Vehicle
Nex20
MTB MIC Chi3l1 OE
2 weeks
O
CK14+ Calponin 1+ P63+ Myoepithelial cells
CK14+ Calponin 1+ P63+ cells/mm²
**
Vehicle
Nex20
MTB MIC Chi3l1 OE
2 weeks

**Supplementary Figure 6: Inhibiting neutrophil degranulation in Chi3l1 OE mice rescues myoepithelial layer loss. Related to Figure 2.** **(A)** Fluorescent IHC staining of mammary glands from vehicle or Nex20-treated WT MIC mice at 2 weeks post induction using antibodies against Ly6G, and Pan-ck, counterstained with DAPI. **(B)** Quantification of total Ly6G+ cells as a percentage of total cells in vehicle (*n* = 8), and Nex20-treated (*n* = 8) MIC mammary glands at 2 weeks post induction. Derived from the staining shown in A. **(C, D)** ELISA for serum MPO and NE from vehicle (*n* = 5) and Nex20 (*n* = 5) treated WT MIC mice at 2 weeks post-induction. **(E)** Immunoblots for CitH3, Chi3l1 and α-tubulin on vehicle (*n* = 5) and Nex20 (*n* = 5) treated WT MIC mammary glands at 2 weeks post-induction. Protein was extracted form flash frozen mammary gland and subjected to immunoblot analysis. **(F,G)** Quantification of CitH3 and Chi3l1 immunoblot normalized to α-tubulin. **(H)** Quantification of total Ly6G+ CitH3+ cells as a percentage of total cells in vehicle (*n* = 8), and Nex20-treated (*n* = 8) MIC mammary glands at 2 weeks post induction. Derived from the staining shown in I. **(I)** Fluorescent IHC staining of mammary glands from vehicle or Nex20-treated WT MIC mice at 2 weeks post induction using antibodies against Ly6G, CitH3 and Pan-ck, counterstained with DAPI. **(J)** Fluorescent IHC staining of mammary glands from vehicle or Nex20-treated Chi3l1 OE MIC mice at 2 weeks post induction using antibodies against CK14, α-SMA, and CK8, counterstained with DAPI. **(K)** Quantification of total CK14+ α-SMA+ cells normalized to total analyzed area in vehicle (*n* = 8) or Nex20-treated (*n* = 8) Chi3l1 OE MIC mammary glands at 2 weeks post induction. Derived from the staining shown in J. **(L)** Fluorescent IHC staining of of mammary glands from vehicle or Nex20-treated Chi3l1 OE MIC mice at 2 weeks post induction using antibodies against CK14, calponin 1, P63 and CK8, counterstained with DAPI (blue). **(M-O)** Quantification of total CK14+ α-SMA+, CK14+ calponin 1+, CK14+ P63+ and CK14+ calponin 1+ P63+ cells normalized to total analyzed area in vehicle (*n* = 8) or Nex20-treated (*n* = 8) Chi3l1 OE MIC mammary glands at 2 weeks post induction. Derived from the staining shown in L. Ns: not significant, *p<0.05 and **p<0.01 calculated by Mann-Whitney non-parametric test. Data are presented as mean ± SEM. Each data point is an independent biological sample, *N* represents the number of independent biological replicates. Analysis was performed by the HALO (Indica lab) software. Quantification was performed on whole-slide scans of the entire mammary gland section.

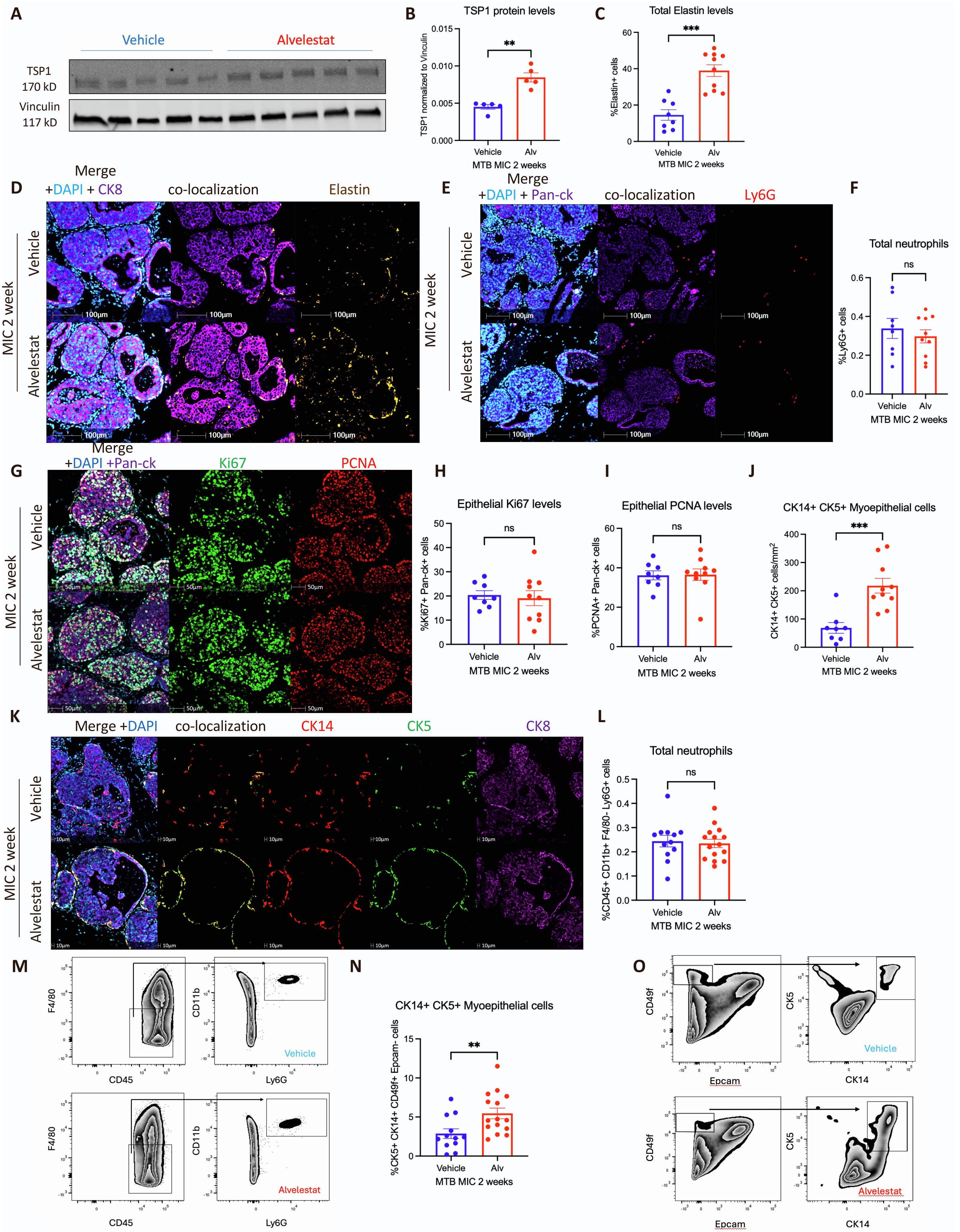
A
Vehicle
Alvelestat
TSP1
170 kD
Vinculin
117 kD
B
TSP1 protein levels
TSP1 normalized to Vinculin
**
Vehicle
Alv
MTB MIC 2 weeks
C
Total Elastin levels
%Elastin+ cells
***
Vehicle
Alv
MTB MIC 2 weeks
D
Merge
+DAPI + CK8
co-localization
Elastin
MIC 2 week
Vehicle
Alvelestat
100µm
E
Merge
+DAPI + Pan-ck
co-localization
Ly6G
MIC 2 week
Vehicle
Alvelestat
100µm
F
Total neutrophils
%Ly6G+ cells
ns
Vehicle
Alv
MTB MIC 2 weeks
G
Merge
+DAPI +Pan-ck
Ki67
PCNA
MIC 2 week
Vehicle
Alvelestat
50µm
H
Epithelial Ki67 levels
%Ki67+ Pan-ck+ cells
ns
Vehicle
Alv
MTB MIC 2 weeks
I
Epithelial PCNA levels
%PCNA+ Pan-ck+ cells
ns
Vehicle
Alv
MTB MIC 2 weeks
J
CK14+ CK5+ Myoepithelial cells
CK14+ CK5+ cells/mm2
***
Vehicle
Alv
MTB MIC 2 weeks
K
Merge +DAPI
co-localization
CK14
CK5
CK8
MIC 2 week
Vehicle
Alvelestat
10µm
L
Total neutrophils
%CD45+ CD11b+ F4/80- Ly6G+ cells
ns
Vehicle
Alv
MTB MIC 2 weeks
M
F4/80
CD45
CD11b
Ly6G
Vehicle
Alvelestat
N
CK14+ CK5+ Myoepithelial cells
%CK5+ CK14+ CD49f+ Epcam- cells
**
Vehicle
Alv
MTB MIC 2 weeks
O
CD49f
Epcam
CK5
CK14
Vehicle
Alvelestat

**Supplementary Figure 7: NE inhibition increases myoepithelial content without impacting neutrophil numbers or cancer cell proliferation. Related to Figure 3.**

**(A)** Immunoblots for TSP1 and vinculin on vehicle (*n* = 5) or Alvelestat (*n* = 5) treated WT MIC mammary glands at 2 weeks post-induction. **(B)** Quantification of TSP1 immunoblot normalized to vinculin. **(C)** Quantification of elastin+ cells as a percentage of total cells in vehicle (*n* = 8), and Alvelestat (*n* = 10) treated MIC mammary glands at 2 weeks post induction. Derived from the staining shown in D. **(D)** Fluorescent IHC staining of mammary glands from vehicle or Alvelestat-treated WT MIC mice at 2 weeks post induction using antibodies against elastin and CK8, counterstained with DAPI. **(E)** Fluorescent IHC staining of mammary glands from vehicle or Alvelestat-treated WT MIC mice at 2 weeks post induction using antibodies against Ly6G and Pan-ck, counterstained with DAPI. **(F)** Quantification of Ly6G+ cells as a percentage of total cells in vehicle (*n* = 8), and Alvelesta (*n* = 10) treated MIC mammary glands at 2 weeks post induction. Derived from the staining shown in E. **(G)** Fluorescent IHC staining of mammary glands from vehicle or Alvelestat-treated WT MIC mice at 2 weeks post induction using antibodies against Ki67, PCNA and Pan-ck, counterstained with DAPI. **(H,I)** Quantification of total Pan-ck+ Ki67+ and Pan-ck+ PCNA+ cells as a percentage of total cells in vehicle (*n* = 8), and Alvelestat (*n* = 10) treated MIC mammary glands at 2 weeks post induction. Derived from the staining shown in G. **(J)** Quantification of total CK14+ CK5+ cells normalized to total analyzed area in vehicle (*n* = 8), and Alvelestat (*n* = 10) treated MIC mammary glands at 2 weeks post induction. Derived from the staining shown in K. **(K)** Fluorescent IHC staining of mammary glands from vehicle or Alvelestat-treated WT MIC mice at 2 weeks post induction using antibodies against CK14, CK5 and CK8 counterstained with DAPI (blue). **(L)** Quantification of CD45+ Cd11b+ F4/80- Ly6G cells as percent of total live cells in vehicle (*n* = 12), and Alvelestat (*n* = 15) treated MIC mammary glands at 2 weeks post induction. **(M)** Representative FACS gating strategy of neutrophil populations in WT MIC mammary glands treated with Alvelestat- or vehicle control for 2 weeks. CD45%+ cells were sorted based on expression F4/80 (macrophage marker) and Cd11b (myeloid marker). Neutrophils (Ly6G+) cells were gated out of the CD11b+ F4/80- cells**.** **(N)** Quantification of Epcam- CD49f+ CK14+ CK5+ cells as percent of total live cells in vehicle (*n* = 12), and Alvelestat (*n* = 15) treated MIC mammary glands at 2 weeks post induction. **(O)** Representative FACS gating strategy of myoepithelial populations in WT MIC mammary glands treated with Alvelestat- or vehicle control for 2 weeks. Briefly, viable cells were sorted based on expression of EPCAM (luminal marker) and CD49f (Myoepithelial basal marker). Myoepithelial cells (CK14+ CK5+) were gated out of EPCAM- CD49f+ population. Ns: not significant, **p<0.01, ***p<0.001 and ****p<0.0001 calculated by Mann-Whitney non-parametric test. Data are presented as mean ± SEM. Each data point is an independent biological sample, *N* represents the number of independent biological replicates. Analysis was performed by the HALO (Indica lab) software. Quantification was performed on whole-slide scans of the entire mammary gland section.

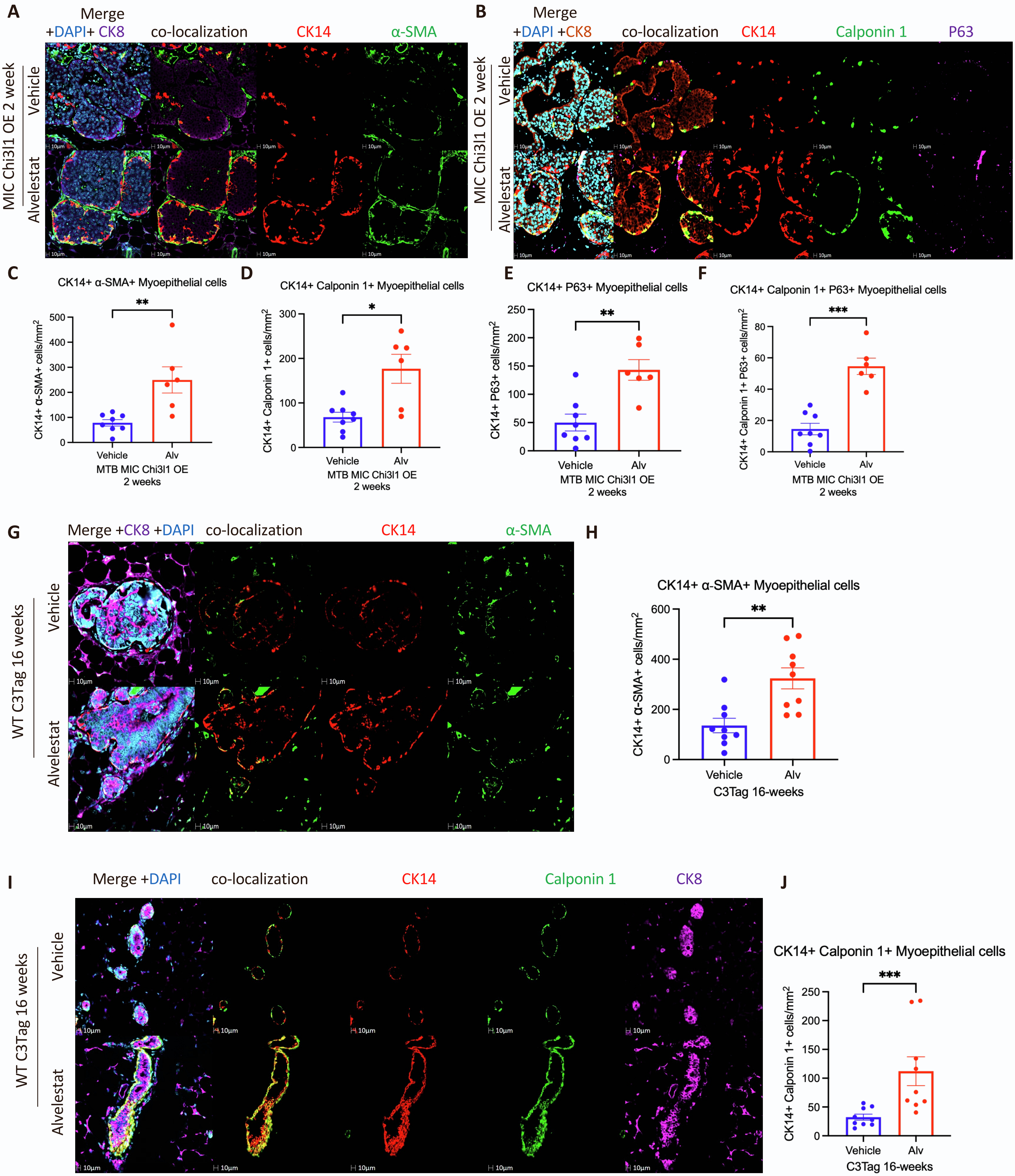

A
Merge +DAPI+ CK8
co-localization
CK14
α-SMA
MIC Chi3l1 OE 2 week
Vehicle
Alvelestat
B
Merge +DAPI +CK8
co-localization
CK14
Calponin 1
P63
MIC Chi3l1 OE 2 week
Vehicle
Alvelestat
C
CK14+ α-SMA+ Myoepithelial cells
CK14+ α-SMA+ cells/mm2
**
Vehicle
Alv
MTB MIC Chi3l1 OE 2 weeks
D
CK14+ Calponin 1+ Myoepithelial cells
CK14+ Calponin 1+ cells/mm2
*
Vehicle
Alv
MTB MIC Chi3l1 OE 2 weeks
E
CK14+ P63+ Myoepithelial cells
CK14+ P63+ cells/mm2
**
Vehicle
Alv
MTB MIC Chi3l1 OE 2 weeks
F
CK14+ Calponin 1+ P63+ Myoepithelial cells
CK14+ Calponin 1+ P63+ cells/mm2
***
Vehicle
Alv
MTB MIC Chi3l1 OE 2 weeks
G
Merge +CK8 +DAPI
co-localization
CK14
α-SMA
WT C3Tag 16 weeks
Vehicle
Alvelestat
H
CK14+ α-SMA+ Myoepithelial cells
CK14+ α-SMA+ cells/mm2
**
Vehicle
Alv
C3Tag 16-weeks
I
Merge +DAPI
co-localization
CK14
Calponin 1
CK8
WT C3Tag 16 weeks
Vehicle
Alvelestat
J
CK14+ Calponin 1+ Myoepithelial cells
CK14+ Calponin 1+ cells/mm2
***
Vehicle
Alv
C3Tag 16-weeks

**Supplementary Figure 8: Inhibiting NE in other GEMMs prevents myoepithelial layer dissolution. Related to Figure 3.**
**(A)** Fluorescent IHC staining of mammary glands from vehicle or Alvelestat-treated Chi3l1 OE MIC mice at 2 weeks post induction using antibodies against CK14, α-SMA, and CK8, counterstained with DAPI. **(B)** Fluorescent IHC staining of of mammary glands from vehicle or Alvelestat-treated Chi3l1 OE MIC mice at 2 weeks post induction using antibodies against CK14, calponin 1, P63 and CK8, counterstained with DAPI. **(C-F)** Quantification of total CK14+ α-SMA+, CK14+ calponin 1+, CK14+ P63+ and CK14+ calponin 1+ P63+ cells normalized to total analyzed area in vehicle (*n* = 8) or Alvelestat (*n* = 6) treated Chi3l1 OE MIC mammary glands at 2 weeks post induction. Derived from the staining shown in A,B. **(G)** Fluorescent IHC staining of mammary glands from vehicle or Alvelestat-treated WT C3Tag mice at 16 weeks using antibodies against CK14, α-SMA, and CK8, counterstained with DAPI. **(H)** Quantification of total CK14+ α-SMA+ cells normalized to total analyzed area in vehicle (*n* = 9) or Alvelestat (*n* = 9) treated WT C3Tag mammary glands at 16 weeks. Derived from the staining shown in G. **(I)** Fluorescent IHC staining of mammary glands from vehicle or Alvelestat-treated WT C3Tag mice at 16 weeks using antibodies against calponin 1, CK14 and CK8, counterstained with DAPI. **(J)** Quantification of total CK14+ calponin 1+ cells normalized to total analyzed area in vehicle (*n* = 9) or Alvelestat (*n* = 9) treated WT C3Tag mammary glands at 16 weeks. Derived from the staining shown in I. *p<0.05 and **p<0.01 calculated by Mann-Whitney non-parametric test. Data are presented as mean ± SEM. Each data point is an independent biological sample, *N* represents the number of independent biological replicates. Analysis was performed by the HALO (Indica lab) software. Quantification was performed on whole-slide scans of the entire mammary gland section.

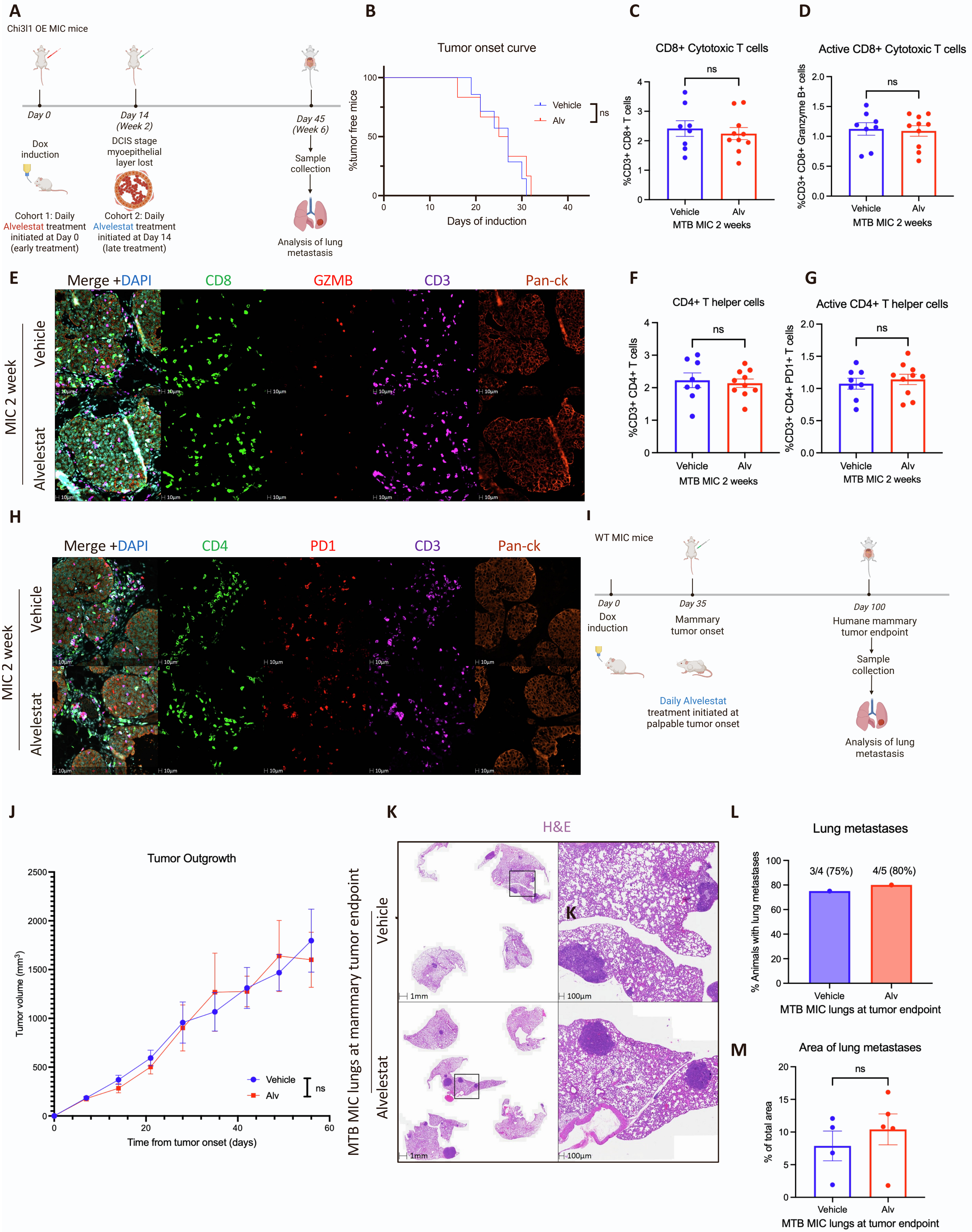
A
Chi3l1 OE MIC mice
Day 0
Day 14 (Week 2)
Day 45 (Week 6)
Dox induction
DCIS stage myoepithelial layer lost
Sample collection
Cohort 1: Daily Alvelestat treatment initiated at Day 0 (early treatment)
Cohort 2: Daily Alvelestat treatment initiated at Day 14 (late treatment)
Analysis of lung metastasis
B
Tumor onset curve
%tumor free mice
Days of induction
Vehicle
Alv
ns
C
CD8+ Cytotoxic T cells
%CD3+ CD8+ T cells
Vehicle
Alv
MTB MIC 2 weeks
D
Active CD8+ Cytotoxic T cells
%CD3+ CD8+ Granzyme B+ cells
E
Merge +DAPI
CD8
GZMB
CD3
Pan-ck
MIC 2 week
Vehicle
Alvelestat
F
CD4+ T helper cells
%CD3+ CD4+ T cells
G
Active CD4+ T helper cells
%CD3+ CD4+ PD1+ T cells
H
Merge +DAPI
CD4
PD1
CD3
Pan-ck
I
WT MIC mice
Day 0
Day 35
Day 100
Dox induction
Mammary tumor onset
Humane mammary tumor endpoint
Sample collection
Daily Alvelestat treatment initiated at palpable tumor onset
Analysis of lung metastasis
J
Tumor Outgrowth
Tumor volume (mm³)
Time from tumor onset (days)
K
H&E
MTB MIC lungs at mammary tumor endpoint
1mm
100µm
L
Lung metastases
% Animals with lung metastases
3/4 (75%)
4/5 (80%)
MTB MIC lungs at tumor endpoint
M
Area of lung metastases
% of total area

**Supplementary Figure 9: Inhibiting NE in established mammary tumor does not impact lung metastasis. Related to Figure 3.**
**(A)** Schematic representation of the experimental plan for early and delayed Alvelestat treatment in Chi3l1 OE MIC mice, created with BioRender.com. **(B)** Mammary tumor onset in Vehicle (*n* = 7) or Alvelestat (*n* = 6)-treated Chi3l1 OE MIC mice tracked by weekly physical palpation. Treatment started at doxycycline induction and continued daily for 6 weeks. Analysis by Log-rank test. **(C,D)** Quantification of total CD3+ CD8+, CD3+ CD8+ GzmB+ cells as a percentage of total cells in vehicle (*n* = 8), and Alvelestat (*n* = 10) treated MIC mammary glands at 2 weeks post induction. Derived from the staining shown in E. **(E)** Fluorescent IHC staining of mammary glands from vehicle or Alvelestat-treated Chi3l1 OE MIC mice at 2 weeks post induction using antibodies against GzmB, CD8, CD3 and Pan-ck, counterstained with DAPI. **(F,G)** Quantification of total CD3+CD4+ and CD3+ CD4+ PD1+ cells as a percentage of total cells in vehicle (*n* = 8), and Alvelestat (*n* = 10) treated MIC mammary glands at 2 weeks post induction. Derived from the staining shown in H. **(H)** Fluorescent IHC staining of mammary glands from vehicle or Alvelestat-treated Chi3l1 OE MIC mice at 2 weeks post induction using antibodies against PD1, CD4, CD3 and Pan-ck, counterstained with DAPI. **(I)** Schematic representation of the experimental plan for Alvelestat treatment in tumor-bearing WT MIC mice, created with BioRender.com. **(J)** Primary tumors growth in Vehicle (*n* = 4), and Alvelestat-treated (*n* = 5) WT MIC mammary tumor-bearing mice. Tumors were measured and tracked by a caliper. **(K)** Representative H&E staining of WT MIC lungs at mammary tumor endpoint after treatment with vehicle or Alvelestat from tumor onset and until humane end point. **(L)** Percentage of WT MIC mice with pulmonary metastases after treatment with vehicle (*n* = 4) or Alvelestat (*n* = 5) from tumor onset and until humane end point. **(M)** Quantification of total pulmonary metastatic area in WT MIC lungs at mammary tumor endpoint after treatment with vehicle (*n* = 4) or Alvelestat (*n* = 5) from tumor onset and until humane end point. Represented as percent of total lung area. Ns: Not significant; *p<0.05, **p<0.01, ***p<0.001 and ****p<0.0001 calculated by Mann-Whitney non-parametric test. Data are presented as mean ± SEM. Each data point is an independent biological sample, *N* represents the number of independent biological replicates. Analysis was performed by the HALO (Indica lab) software. Quantification was performed on whole-slide scans of the entire mammary gland section.

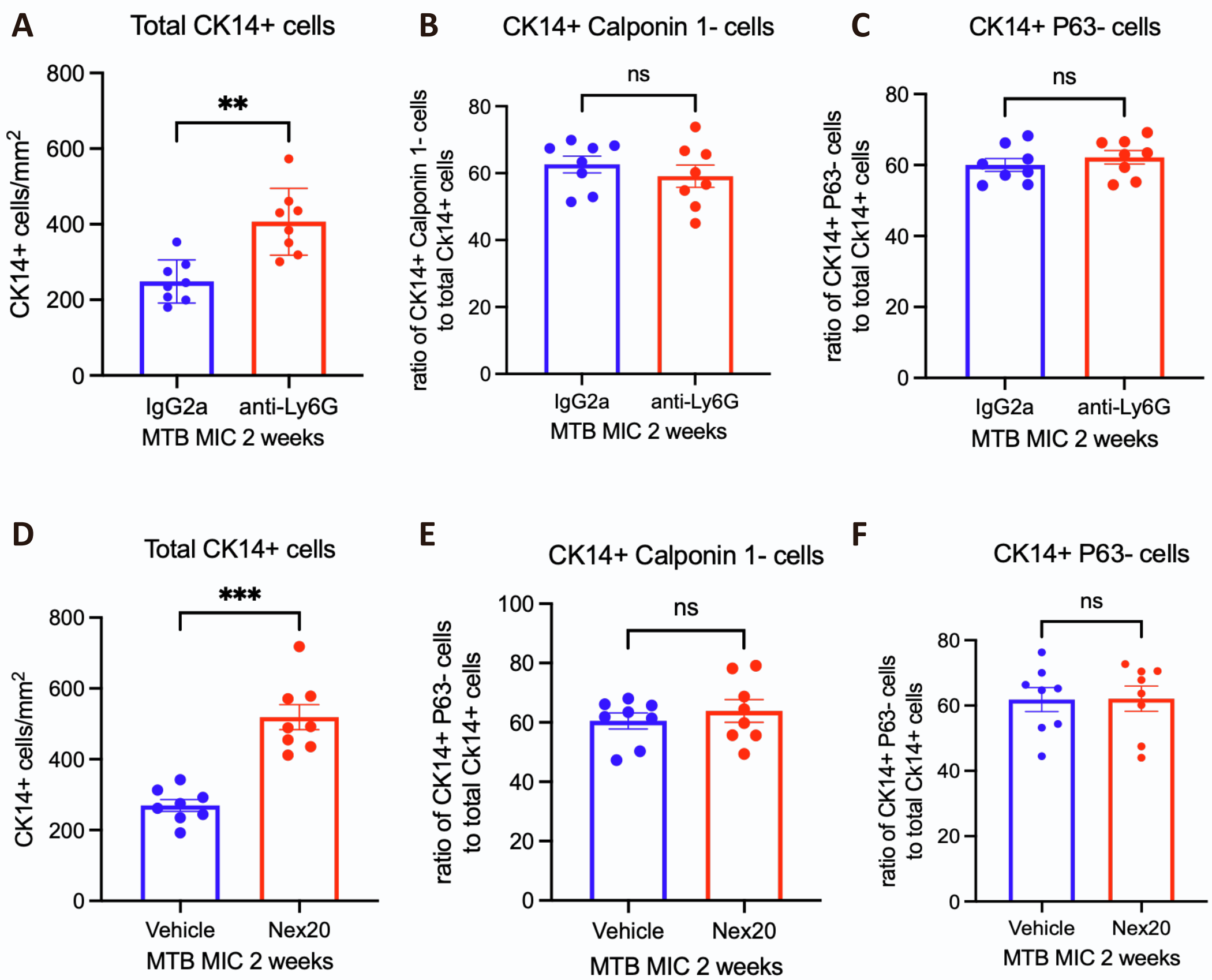
A
Total CK14+ cells
CK14+ cells/mm²
800
600
400
200
0
**
IgG2a
anti-Ly6G
MTB MIC 2 weeks
B
CK14+ Calponin 1- cells
ratio of CK14+ Calponin 1- cells to total Ck14+ cells
80
60
40
20
0
ns
IgG2a
anti-Ly6G
MTB MIC 2 weeks
C
CK14+ P63- cells
ratio of CK14+ P63- cells to total Ck14+ cells
80
60
40
20
0
ns
IgG2a
anti-Ly6G
MTB MIC 2 weeks
D
Total CK14+ cells
CK14+ cells/mm²
800
600
400
200
0
***
Vehicle
Nex20
MTB MIC 2 weeks
E
CK14+ Calponin 1- cells
ratio of CK14+ P63- cells to total Ck14+ cells
100
80
60
40
20
0
ns
Vehicle
Nex20
MTB MIC 2 weeks
F
CK14+ P63- cells
ratio of CK14+ P63- cells to total Ck14+ cells
80
60
40
20
0
ns
Vehicle
Nex20
MTB MIC 2 weeks

**Supplementary Figure 10: Targeting neutrophils and their effector function does not affect myoepithelial cell differentiation. Related to Figure 3.**

**(A-C)** Quantification of total CK14+ and the percentage of CK14+ calponin 1-, CK14+ P63- within total CK14+ in IgG2a (*n* = 8) and anti-Ly6G (*n* = 8) treated WT MIC mammary glands at 2 weeks post induction. Analyzed from staining performed in Figure 1A. **(D-E)** Quantification of total CK14+, and the percentage of CK14+ calponin 1-, CK14+ P63- within total CK14+ in vehicle (*n* = 8), and Nex20-treated (*n* = 8) MIC mammary glands at 2 weeks post induction. Analyzed from staining performed in Figure 2A. Ns: Not significant; **p<0.01 and ***p<0.001 calculated by Mann-Whitney non-parametric test. Data are presented as mean ± SEM. Each data point is an independent biological sample, *N* represents the number of independent biological replicates. Analysis was performed by the HALO (Indica lab) software. Quantification was performed on whole-slide scans of the entire mammary gland section.

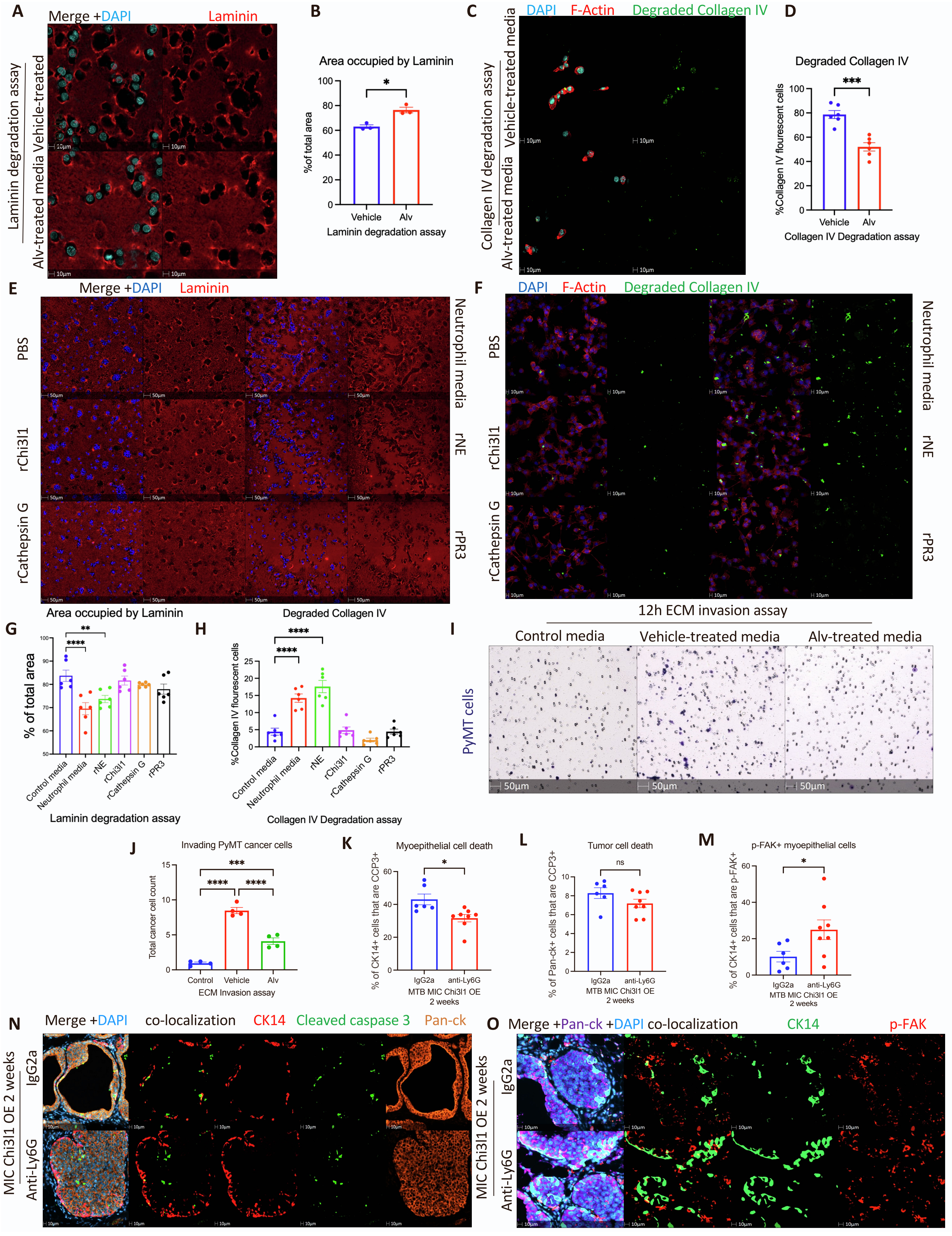

A
Merge +DAPI
Laminin
Laminin degradation assay
Vehicle-treated
Alv-treated media
B
Area occupied by Laminin
%of total area
Vehicle
Alv
Laminin degradation assay
C
DAPI F-Actin Degraded Collagen IV
Collagen IV degradation assay
Vehicle-treated media
Alv-treated media
D
Degraded Collagen IV
%Collagen IV flourescent cells
Vehicle
Alv
Collagen IV Degradation assay
E
Merge +DAPI Laminin
PBS
rChi3l1
rCathepsin G
Neutrophil media
rNE
rPR3
F
DAPI F-Actin Degraded Collagen IV
PBS
rChi3l1
rCathepsin G
Neutrophil media
rNE
rPR3
G
Area occupied by Laminin
% of total area
Control media
Neutrophil media
rNE
rChi3l1
rCathepsin G
rPR3
Laminin degradation assay
H
Degraded Collagen IV
%Collagen IV flourescent cells
Control media
Neutrophil media
rNE
rChi3l1
rCathepsin G
rPR3
Collagen IV Degradation assay
I
12h ECM invasion assay
Control media
Vehicle-treated media
Alv-treated media
PyMT cells
50µm
J
Invading PyMT cancer cells
Total cancer cell count
Control
Vehicle
Alv
ECM Invasion assay
K
Myoepithelial cell death
% of CK14+ cells that are CCP3+
IgG2a
anti-Ly6G
MTB MIC Chi3l1 OE
2 weeks
L
Tumor cell death
% of Pan-ck+ cells that are CCP3+
ns
IgG2a
anti-Ly6G
MTB MIC Chi3l1 OE
2 weeks
M
p-FAK+ myoepithelial cells
% of CK14+ cells that are p-FAK+
IgG2a
anti-Ly6G
MTB MIC Chi3l1 OE
2 weeks
N
Merge +DAPI co-localization CK14 Cleaved caspase 3 Pan-ck
MIC Chi3l1 OE 2 weeks
IgG2a
Anti-Ly6G
O
Merge +Pan-ck +DAPI co-localization CK14 p-FAK
MIC Chi3l1 OE 2 weeks
IgG2a
Anti-Ly6G

**Supplementary Figure 11: NE mediates ECM degradation and myoepithelial cell anoikis. Related to Figure 4 and 5.**
**(A)** Staining for laminin and DAPI on PyMT+ cells cultured on synthetic EMC in neutrophil-conditioned media treated with vehicle or Alvelestat. **(B)** Quantification of area occupied by laminin after culture in neutrophil-conditioned media (*n* = 3 technical replicates per condition). Represented as represented as percent of total analyzed area. Derived from the staining shown in A. **(C)** PyMT+ cells cultured on fluorescently-labeled collagen IV ECM in neutrophil conditioned media treated with vehicle or Alvelestat. Collagen IV would fluoresce green when degraded. **(D)** Quantification of degraded collagen IV as a percentage of total cells after culture in neutrophil-conditioned media (*n* = 6 technical replicates per condition). Derived from the staining shown in C. **(E)** Staining for laminin and DAPI on PyMT+ cells cultured on synthetic EMC in control media supplemented with PBS or supplemented with recombinant NE (rNE), recombinant cathepsin G, recombinant proteinase 3 (PR3) or recombinant Chi3l1, or cultured in in neutrophil conditioned media. **(F)** PyMT+ cells cultured on fluorescently-labeled collagen IV ECM in control media supplemented with PBS or supplemented with rNE, recombinant cathepsin G, rPR3 or rChi3l1, or cultured in in neutrophil conditioned media. **(G)** Quantification of area occupied by laminin after culture in the presence of recombinant proteins (*n* = 6 technical replicates per condition). Represented as represented as percent of total analyzed area. Derived from the staining shown in E. **(H)** Quantification of degraded collagen IV as a percentage of total cells after culture in presence of recombinant proteins (*n* = 6 technical replicates per condition). Derived from the staining shown in F. **(I)** Representative images of invading PyMT+ cells when cultured on a layer of synthetic ECM with control media, neutrophil conditioned media treated with vehicle, or Alvelestat stained with crystal violet. **(J)** Quantification of total invading PyMT+ cancer cells cultured on a layer of synthetic ECM with control media, neutrophil conditioned media treated with vehicle or Alvelestat. *n* = 4 technical replicates for each condition. Derived from the staining shown in I. **(K)** Quantification of the percentage of CK14+ cleaved caspase 3+ cells cells divided by total CK14+ cells in IgG2a (*n* = 6) and anti-Ly6G (*n* = 8) treated Chi3l1 OE MIC mammary glands at 2 weeks post induction. Derived from the staining shown in N. **(L)** Quantification of the percentage of Pan-ck+ cleaved caspase 3+ cells cells within total Pan-ck+ cells in IgG2a (*n* = 6) and anti-Ly6G (*n* = 8) treated Chi3l1 OE MIC mammary glands at 2 weeks post induction. Derived from the staining shown in N. **(M)** Quantification of the percentage of CK14+ p-FAK+ cells cells divided by total CK14+ cells in IgG2a (*n* = 6) and anti-Ly6G (*n* = 8) treated Chi3l1 OE MIC mammary glands at 2 weeks post induction. Derived from the staining shown in O. **(N)** Fluorescent IHC staining of mammary tissue from Chi3l1 OE MIC mice treated with anti-Ly6G or IgG2a isotype control for 2 weeks, using antibodies against CK14, cleaved caspase 3 and pan-ck, counterstained with DAPI. **(O)** Fluorescent IHC staining of mammary tissue from Chi3l1 OE MIC mice treated with anti-Ly6G or IgG2a isotype control for 2 weeks, using antibodies against CK14, p-FAK and Pan-ck, counterstained with DAPI. Ns: not significant, *p<0.05, ***p<0.001 and ****p<0.0001 calculated by Student's T-test (For B,D), One-way anova with Dunnet's post hoc test (for F,H), One-way anova with Tuckey's post hoc test (For J) or using Mann-Whitney non-parametric test (For K-M). Data are presented as mean ± SEM. Each data point is an independent biological sample, *N* represents the number of independent biological replicates. Analysis was performed by the HALO (Indica lab) software. Quantification was performed on whole-slide scans of the entire mammary gland section.

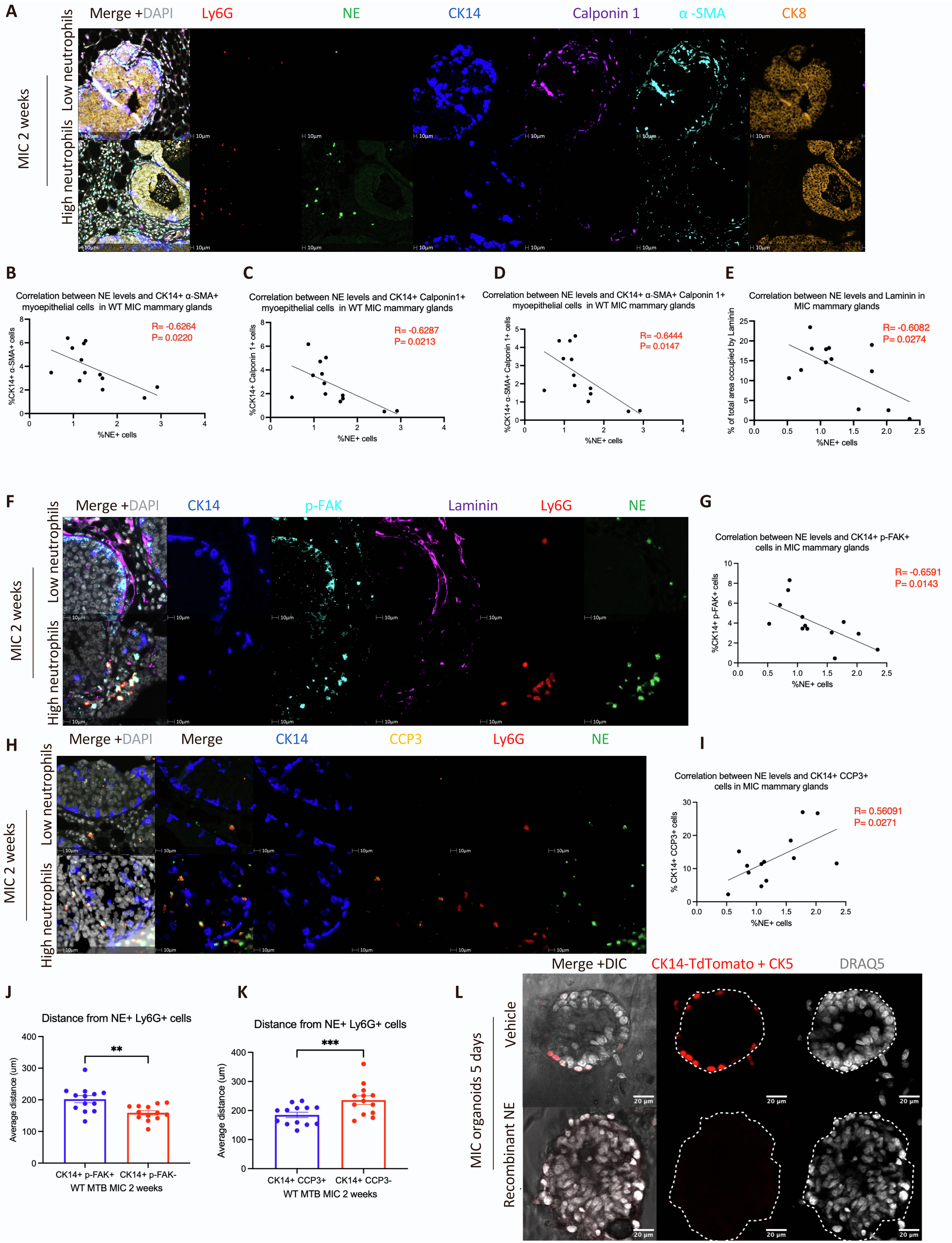
A
MIC 2 weeks
Low neutrophils
High neutrophils
Merge +DAPI
Ly6G
NE
CK14
Calponin 1
α -SMA
CK8
10μm
B
Correlation between NE levels and CK14+ α-SMA+ myoepithelial cells in WT MIC mammary glands
R= -0.6264
P= 0.0220
%CK14+ α-SMA+ cells
%NE+ cells
C
Correlation between NE levels and CK14+ Calponin1+ myoepithelial cells in WT MIC mammary glands
R= -0.6287
P= 0.0213
%CK14+ Calponin 1+ cells
%NE+ cells
D
Correlation between NE levels and CK14+ α-SMA+ Calponin 1+ myoepithelial cells in WT MIC mammary glands
R= -0.6444
P= 0.0147
%CK14+ α-SMA+ Calponin 1+ cells
%NE+ cells
E
Correlation between NE levels and Laminin in MIC mammary glands
R= -0.6082
P= 0.0274
% of total area occupied by Laminin
%NE+ cells
F
MIC 2 weeks
Low neutrophils
High neutrophils
Merge +DAPI
CK14
p-FAK
Laminin
Ly6G
NE
10μm
G
Correlation between NE levels and CK14+ p-FAK+ cells in MIC mammary glands
R= -0.6591
P= 0.0143
%CK14+ p-FAK+ cells
%NE+ cells
H
MIC 2 weeks
Low neutrophils
High neutrophils
Merge +DAPI
Merge
CK14
CCP3
Ly6G
NE
10μm
I
Correlation between NE levels and CK14+ CCP3+ cells in MIC mammary glands
R= 0.56091
P= 0.0271
% CK14+ CCP3+ cells
%NE+ cells
J
Distance from NE+ Ly6G+ cells
**
Average distance (um)
CK14+ p-FAK+
CK14+ p-FAK-
WT MTB MIC 2 weeks
K
Distance from NE+ Ly6G+ cells
***
Average distance (um)
CK14+ CCP3+
CK14+ CCP3-
WT MTB MIC 2 weeks
L
MIC organoids 5 days
Vehicle
Recombinant NE
Merge +DIC
CK14-TdTomato + CK5
DRAQ5
20 μm

**Supplementary Figure 12: Spatial analysis of NE and myoepithelial markers in WT MIC mammary glands. Related to Figure 6.**
**(A)** Fluorescent IHC staining of mammary tissue from WT MIC mice at 2 weeks post induction, using antibodies against CK14, Calponin 1, α -SMA, Ly6G, NE and CK8 counterstained with DAPI. **(B-D)** Pearson correlation between NE+ cells and the total CK14+ calponin 1, CK14+ α-SMA+ and CK14+ calponin 1+ α-SMA+ cells normalized to total analyzed area in WT MIC mammary glands at 2 weeks post induction (*n* = 13). Derived from the staining shown in A. **(E)** Pearson correlation between NE+ cells and the the area occupied by laminin in WT MIC mammary glands at 2 weeks post induction (*n* = 13). Derived from the staining shown in F. **(F)** Fluorescent IHC staining of mammary tissue from WT MIC mice at 2 weeks post induction, using antibodies against CK14, p-FAK, Laminin, Ly6G and NE counterstained with DAPI. **(G)** Pearson correlation between NE+ cells and the the area occupied by laminin or total CK14 p-Fak+ cells represented as percentage of total cells in WT MIC mammary glands at 2 weeks post induction (*n* = 13). Derived from the staining shown in F. **(H)** Fluorescent IHC staining of mammary tissue from WT MIC mice at 2 weeks post induction, using antibodies against CK14, Cleaved caspase 3, Ly6G and NE counterstained with DAPI. **(I)** Pearson correlation between NE+ cells and total CK14 CCP3+ cells represented as percentage of total cells (*n* = 13). Derived from the staining shown in H. **(J)** Quantification of the average distance of Ly6G+ NE+ cells to CK14+ p-FAK+ cells or CK14+ p-FAK- cells in WT MIC mice at 2 weeks post induction (*n* = 13). Derived from the staining shown in F. **(K)** Quantification of the average distance of Ly6G+ NE+ cells to CK14+ CCP3+ cells or CK14+ CCP3- cells in WT MIC mice at 2 weeks post induction (*n* = 13). Derived from the staining shown in H. **(L)** Fluorescent staining of organoids grown from MIC CK14-Tdtomato mammary glands induced in culture for 5 days and treated with vehicle or recombinant neutrophil elastase for 24 hours. In red, CK5 immunostaining and endogenous K14 fluorescence are visible and in grey DRAQ5 marks nuclei. White dotted line used to outline organoid seen by DIC. **p<0.01 and ****p<0.0001 calculated by paired Student's T-test. Data are presented as mean ± SEM. Pearson correlation was performed using two-tailed t test. Each data point is an independent biological sample, *N* represents the number of independent biological replicates. Analysis was performed by the HALO (Indica lab) software. Quantification was performed on whole-slide scans of the entire mammary gland section.

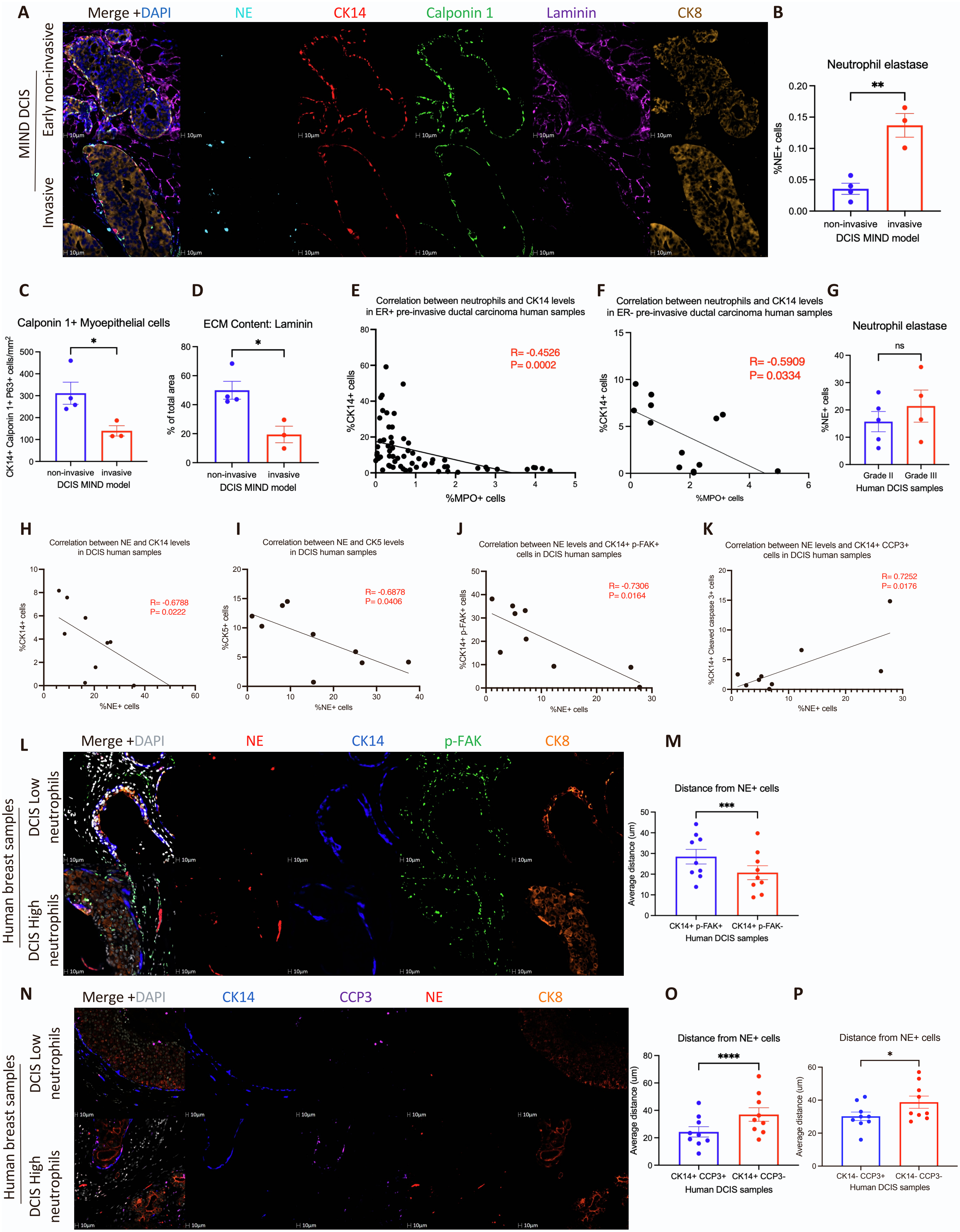
A
Merge +DAPI
NE
CK14
Calponin 1
Laminin
CK8
MIND DCIS
Early non-invasive
Invasive
B
Neutrophil elastase
%NE+ cells
non-invasive
invasive
DCIS MIND model
C
Calponin 1+ Myoepithelial cells
CK14+ Calponin 1+ P63+ cells/mm2
non-invasive
invasive
DCIS MIND model
D
ECM Content: Laminin
% of total area
non-invasive
invasive
DCIS MIND model
E
Correlation between neutrophils and CK14 levels in ER+ pre-invasive ductal carcinoma human samples
R= -0.4526
P= 0.0002
%CK14+ cells
%MPO+ cells
F
Correlation between neutrophils and CK14 levels in ER- pre-invasive ductal carcinoma human samples
R= -0.5909
P= 0.0334
%CK14+ cells
%MPO+ cells
G
Neutrophil elastase
ns
%NE+ cells
Grade II
Grade III
Human DCIS samples
H
Correlation between NE and CK14 levels in DCIS human samples
R= -0.6788
P= 0.0222
%CK14+ cells
%NE+ cells
I
Correlation between NE and CK5 levels in DCIS human samples
R= -0.6878
P= 0.0406
%CK5+ cells
%NE+ cells
J
Correlation between NE levels and CK14+ p-FAK+ cells in DCIS human samples
R= -0.7306
P= 0.0164
%CK14+ p-FAK+ cells
%NE+ cells
K
Correlation between NE levels and CK14+ CCP3+ cells in DCIS human samples
R= 0.7252
P= 0.0176
%CK14+ Cleaved caspase 3+ cells
%NE+ cells
L
Merge +DAPI
NE
CK14
p-FAK
CK8
Human breast samples
DCIS Low neutrophils
DCIS High neutrophils
M
Distance from NE+ cells
Average distance (um)
CK14+ p-FAK+
CK14+ p-FAK-
Human DCIS samples
N
Merge +DAPI
CK14
CCP3
NE
CK8
Human breast samples
DCIS Low neutrophils
DCIS High neutrophils
O
Distance from NE+ cells
Average distance (um)
CK14+ CCP3+
CK14+ CCP3-
Human DCIS samples
P
Distance from NE+ cells
Average distance (um)
CK14- CCP3+
CK14- CCP3-
Human DCIS samples

**Supplementary Figure 13: Spatial analysis of NE and myoepithelial markers in human samples samples. Related to Figure 6.**
**(A)** Fluorescent IHC staining of early DCIS and invasive human MIND DCIS using antibodies against NE, CK14, Calponin 1, laminin and CK8, counterstained with DAPI. **(B)** Quantification of total NE+ cells as a percentage of total cells in early DCIS (*n* = 4), and invasive (*n* = 3) MIND samples. Derived from the staining shown in in A. **(C)** Quantification of total CK14+ calponin 1+ cells normalized to total analyzed area in early DCIS (*n* = 4), and invasive (*n* = 3) MIND samples. Derived from the staining shown in A. **(D)** Quantification of area occupied by laminin early DCIS (*n* = 4), and invasive (*n* = 3) MIND samples. Represented as percent of mammary gland area. Derived from the staining shown in I A. **(E, F)** Pearson correlation between neutrophils (MPO+ cells) and the total CK14+ cells in human ER+ (*n* = 62) or ER- (*n* = 13) DCIS samples. Both represented as a percentage of total cells. Derived from the staining shown in in Fig. 6A. **(G)** Quantification of total NE+ cells as a percentage of total cells in grade II (*n* = 5) or grade III (*n* = 4) human DCIS samples. Derived from the staining shown in in Fig. 6E. **(H-K)** Pearson correlation between NE+ cells and total CK14+ cells, total CK5+ cells, CK14+ p-FAK+ cells or CK14+ CCP3+ cells in human DCIS samples (*n* = 9). All represented as a percentage of total cells. Derived from the staining shown in in 6E and S12L.**(L)** Fluorescent IHC staining of human DCIS samples using antibodies against NE, CK14, p-FAK, and CK8, counterstained with DAPI. Micrograph represents areas of high NE and low NE from different DCIS patient samples. **(M)** Quantification of the average distance of NE+ cells to CK14+ p-FAK+ cells or CK14+ p-FAK- cells in human DCIS samples (*n* = 9). Derived from the staining shown in in L. **(N)** Fluorescent IHC staining of human DCIS samples using antibodies against NE, CK14, CCP3, and CK8, counterstained with DAPI. Micrograph represents areas of high NE and low NE from different DCIS patient samples **(O)** Quantification of the average distance of NE+ cells to CK14+ CCP3+ cells or CK14+ CCP3- cells in human DCIS samples (*n* = 9). Derived from the staining shown in N. **(P)** Quantification of the average distance of NE+ cells to CK14- CCP3+ cells or CK14- CCP3- cells in human DCIS samples (*n* = 9). Derived from the staining shown in N. Ns: not significant, *p<0.05, **p<0.01, ***p<0.001 and ****p<0.0001 calculated by Student's T-test. Paired t-test was used for L and N. Pearson correlation was performed using two-tailed t test. Data are presented as mean ± SEM. Each data point is an independent biological sample, *N* represents the number of independent biological replicates. Analysis was performed by the HALO (Indica lab) software. Quantification was performed on whole-slide scans of the entire mammary gland section.

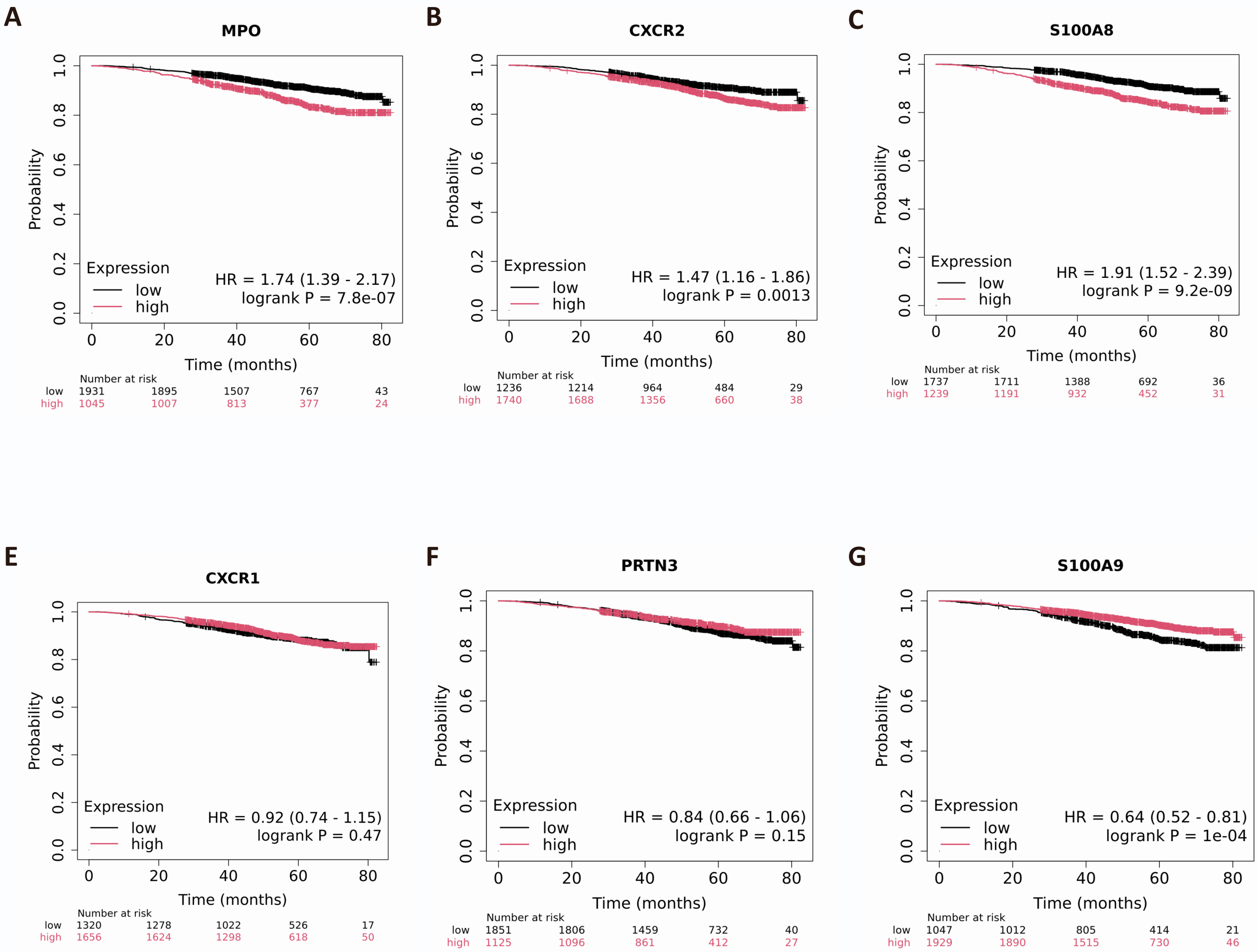
A
MPO
Probability
Expression
low
high
HR = 1.74 (1.39 - 2.17)
logrank P = 7.8e-07
Time (months)
Number at risk
low 1931 1895 1507 767 43
high 1045 1007 813 377 24
B
CXCR2
Probability
Expression
low
high
HR = 1.47 (1.16 - 1.86)
logrank P = 0.0013
Time (months)
Number at risk
low 1236 1214 964 484 29
high 1740 1688 1356 660 38
C
S100A8
Probability
Expression
low
high
HR = 1.91 (1.52 - 2.39)
logrank P = 9.2e-09
Time (months)
Number at risk
low 1737 1711 1388 692 36
high 1239 1191 932 452 31
E
CXCR1
Probability
Expression
low
high
HR = 0.92 (0.74 - 1.15)
logrank P = 0.47
Time (months)
Number at risk
low 1320 1278 1022 526 17
high 1656 1624 1298 618 50
F
PRTN3
Probability
Expression
low
high
HR = 0.84 (0.66 - 1.06)
logrank P = 0.15
Time (months)
Number at risk
low 1851 1806 1459 732 40
high 1125 1096 861 412 27
G
S100A9
Probability
Expression
low
high
HR = 0.64 (0.52 - 0.81)
logrank P = 1e-04
Time (months)
Number at risk
low 1047 1012 805 414 21
high 1929 1890 1515 730 46

**Supplementary Figure 14: Clinical correlations of various neutrophil markers in breast cancer. Related to Figure 6. (A-C)** Elevated levels of MPO, CXCR2 and S100A8 are associated with decreased overall survival in breast cancer patients of all subtypes at 5 years post diagnosis (*n* = 2976). **(E-G)** Elevated levels of CXCR1, PRTN3 and S100A9 are not associated with decreased overall survival in breast cancer patients of all subtypes at 5 years post diagnosis (*n* = 2976). Analyzed with log-rank test, p-value presented in the figure.

| Variable | Non-metastatic (n= 21) | Metastatic (n= 12) |
|---|---|---|
| **Average Age** | 70 | 68.4 |
| **ER positive** | 14 (66%) | 7 (58%) |
| **PR positive** | 11 (52%) | 6 (50%) |
| **HER2 positive** | 19 (90%) | 11(92%) |
| **Grade II** | 9 (43%) | 6 (50%) |
| **Grade III** | 12 (57%) | 6 (50%) |
| **Tumor size** | 20.5 mm | 22.8mm |

**Supplementary Table 1: Clinicopathological characteristics of the breast cancer cohort used for metastatic versus non-metastatic analyses. Related to figure 6.**
